# Supplementary material for: Schizophrenia-associated methylomic variation: molecular signatures of disease and polygenic risk burden across multiple brain regions
Source: Hum Mol Genet. 2016 Dec 22;26(1):210–25. doi: 10.1093/hmg/ddw373 (PMC5351932; doi:10.1093/hmg/ddw373)
Supplement: Supplementary Data [file ddw373_Supp.zip › Main Text Markup.docx]

**Schizophrenia-associated methylomic variation: molecular signatures of disease and polygenic risk burden across multiple brain regions**

Joana Viana^1^, Eilis Hannon^1^, Emma Dempster^1^, Ruth Pidsley^2^, Ruby Macdonald^1^, Olivia Knox^1^, Helen Spiers^3^, Claire Troakes^3^, Safa Al-Saraj^3^, Gustavo Turecki^4^, Leonard C Schalkwyk^5^, Jonathan Mill^1,3,*^

^1^ University of Exeter Medical School, University of Exeter, Exeter, EX2 5DW, UK.

^2^ Garvan Institute of Medical Research, Sydney 2010, NSW, Australia.

^3^ Institute of Psychiatry, Psychology & Neuroscience, King’s College London, London, SE5 8AF, UK.

^4^ Douglas Mental Health Institute, McGill University, Montreal H4H 1R3, QC, Canada.

^5^ School of Biological Sciences, University of Essex, Colchester CO4 3SQ, UK.

* Corresponding author: Jonathan Mill, University of Exeter Medical School, RILD Building, Royal Devon & Exeter Hospital, Barrack Road, Exeter. EX2 5DW. UK.

E-mail: j.mill@exeter.ac.uk

**ABSTRACT**

Genetic association studies provide evidence for a substantial polygenic component to schizophrenia, although the neurobiological mechanisms underlying the disorder remain largely undefined. Building on recent studies supporting a role for developmentally regulated epigenetic variation in the molecular etiology of schizophrenia, this study aimed to identify epigenetic variation associated with both a diagnosis of schizophrenia and elevated polygenic risk burden for the disease across multiple brain regions. Genome-wide DNA methylation was quantified in 262 post-mortem brain samples, representing tissue from four brain regions (prefrontal cortex, striatum, hippocampus and cerebellum) from 41 schizophrenia patients and 47 controls. We identified multiple disease-associated and polygenic risk score-associated differentially methylated positions and regions, which are not enriched in genomic regions identified in genetic studies of schizophrenia and do not reflect direct genetic effects on DNA methylation. Our study represents the first analysis of epigenetic variation associated with schizophrenia across multiple brain regions and highlights the utility of polygenic risk scores for identifying molecular pathways associated with etiological variation in complex disease.

**INTRODUCTION**

Schizophrenia is a severe neurodevelopmental disorder, characterized by episodic psychosis and altered cognitive function([1](#_ENREF_1)), that contributes significantly to the global burden of disease([2](#_ENREF_2)). Twin and family studies have highlighted a notable heritable component to schizophrenia([3](#_ENREF_3)), however the role of genetic variation in the etiology of the disorder is complex. Rare, highly penetrant inherited and *de novo* mutations have been implicated in some cases of schizophrenia([4-7](#_ENREF_4)), however, susceptibility is predominantly attributed to the action of common genetic variants of low penetrance. Recently, a large-scale genome-wide association study (GWAS) identified 108 independent genomic loci exhibiting genome-wide significant association with schizophrenia, which provided convincing evidence for a substantial polygenic component to etiology within signals falling below genome-wide levels of significance([8](#_ENREF_8)). Despite these advances in understanding the genetic epidemiology of schizophrenia, little is known about the mechanisms by which schizophrenia risk variants mediate disease susceptibility in the brain([9](#_ENREF_9), [10](#_ENREF_10)).

Improved understanding about the biology of the genome has led to increased interest in the role of non-DNA sequence-based variation in the etiology of neurodevelopmental phenotypes, including schizophrenia. Epigenetic processes have been hypothesized to mediate associations between genetic risk burden, environmental risk exposure and phenotype. Furthermore, a growing number of studies provide evidence for the dysregulation of epigenetic mechanisms in complex psychiatric disorders([9](#_ENREF_9), [11-13](#_ENREF_11)). To date, such studies have primarily focused on DNA methylation at CpG dinucleotides, as this is the best characterized and most stable epigenetic modification. DNA methylation influences gene expression via physical disruption of transcription factor binding and through attraction of methyl-binding proteins that initiate chromatin compaction and gene silencing. Of note, previous studies characterizing schizophrenia-associated methylomic variation have been limited by small sample number or the assessment of a single brain region([14-19](#_ENREF_14)).

This study represents the first attempt to systematically examine the association of genome-wide methylomic variation with schizophrenia and schizophrenia polygenic risk burden, across multiple brain regions, using post-mortem tissue obtained from two independent cohorts of schizophrenia patients and controls.

**RESULTS**

*Overview of experimental strategy*

We quantified genome-wide patterns of DNA methylation in 262 post-mortem samples derived from four brain regions dissected from 88 individuals (41 schizophrenia and 47 non-psychiatric controls) obtained from two independent brain banks, using the Illumina Infinium HumanMethylation450 BeadChip (450K array) (Illumina Inc., San Diego, CA, USA) (see **Materials and Methods**). In total, data from 76 prefrontal cortex (PFC; n = 38 schizophrenia patients and 38 controls), 82 striatum (STR; n = 37 schizophrenia patients and 45 controls), 27 hippocampus (HC; n = 14 schizophrenia patients and 13 controls) and 77 cerebellum (CER; n = 37 schizophrenia patients and 40 controls) samples passed stringent quality control (QC) metrics and were used for analysis (**Table 1** and **Supplementary Figure 1**). For post-mortem brain regions available from both brain banks (PFC, STR and CER), a meta-analysis approach was used to combine data from both sources. Our initial analyses focused on identifying differentially methylated positions (DMPs) and differentially methylated regions (DMRs) associated with disease status. Analyses were first performed independently for each brain region, and we subsequently employed a multi-level model to identify consistent DNA methylation markers of schizophrenia present across multiple brain regions. We subsequently calculated a schizophrenia polygenic risk score (PRS) for Caucasian samples (**Supplementary Table 1**) to identify DMPs and DMRs associated with polygenic risk burden. A schematic overview of the study is given in **Supplementary Figure 2** with more detailed experimental procedures described in the **Material and Methods** section.

*Methylomic differences between schizophrenia cases and controls – differentially methylated positions and regions*

No global differences in DNA methylation - estimated by averaging across all probes on the array included in our analysis - were identified between schizophrenia patients and controls in any of the four brain regions (PFC: schizophrenia (SZ) = 48.43%, controls (CTR) = 48.57%, *=* 0.51; STR: SZ = 49.20%, CTR = 49.16%, *P =* 0.12; HC: SZ = 48.44%, CTR = 48.38%, *P =* 0.05; CER: SZ = 47.25%, CTR = 47.27%, *P =* 0.89). Furthermore, the estimated “DNA methylation age” for each sample – calculated using an epigenetic clock based on DNA methylation values ([20](#_ENREF_20), [21](#_ENREF_21)) – was strongly correlated with actual chronological age in each brain region (PFC: ρ= 0.94, *P* < 2.20E-16; STR: ρ = 0.93, *P* < 2.20E-16; HC: ρ = 0.94, *P* = 1.30E-13; CER: ρ = 0.86, *P* = 2.20E-16) (**Supplementary Figure 3**), with no evidence for accelerated “epigenetic aging” in affected individuals (PFC: *P =* 0.16, STR: *P =* 0.73, HC: *P =* 0.73, CER: *P =* 0.30). Taken together, these data indicate that schizophrenia is not associated with any systemic methylomic differences across the probes included on the Illumina 450K array in the brain regions tested in this study.

In contrast, we find significant evidence for schizophrenia-associated variation at specific loci across the genome in each brain region. Our first analyses focused on identifying DNA methylation differences between schizophrenia cases and non-psychiatric controls. The fifty top-ranked schizophrenia-associated DMPs in each brain region are listed in **Supplementary Tables 2-5** and **Supplementary Figures 4-7,** with 12 DMPs passing a highly stringent significance threshold (*P* < 1.66E-07, see **Materials & Methods**) (**Table 2**, **Figure 1** and **Supplementary Figure 8**). Results for all probes included in the final QC’d dataset can be downloaded from <http://epigenetics.essex.ac.uk/schizobrain/>. Although the specific list of top-ranked DMPs identified in each tissue is relatively distinct, many DMPs are characterized by consistent effects across brain regions (**Supplementary Figures 4-7**), and for DMPs identified in each of the four individual brain regions, schizophrenia-associated DNA methylation differences are significantly positively correlated with those at the same probes in the other three brain regions (correlations for: PFC DMPs = 0.43 (STR), 0.31 (HC), 0.61 (CER); STR SMPs = 0.77 (PFC), 0.65 (HC), 0.74 (CER); HC DMPs = 0.63 (PFC), 0.74 (HC), 0.54 (CER); and CER DMPs = 0.64 (PFC), 0.36 (STR), 0.46 (HC)) (**Supplementary Figure 9**).

Quantile-quantile (Q-Q) plots for the *P*-values of the analyses in each tissue are shown in **Supplementary Figure 10A-D** highlighting some evidence of *P-*value inflation (PFC λ = 1.18, STR λ = 1.02, HC λ = 1.13, CER λ = 1.23) in several of the brain regions; such inflation is not unusual in epigenome-wide association study (EWAS) analyses and standard genomic control methods – widely used in GWAS – are not suitable for EWAS data ([26](#_ENREF_26)). Because it is likely that unmeasured factors beyond the variables included in our analysis model (i.e. age, sex, and estimated neuronal proportion) confound our case-control analysis of methylomic variation associated with schizophrenia, we therefore investigated the impact of additional surrogate variables capturing variation in DNA methylation on the association statistics for schizophrenia-associated DMPs. We compared the regression coefficients from our initial analysis model to sequential models iteratively including up to 10 principal components (PCs) derived from the DNA methylation data, observing a strong positive correlation for schizophrenia-associated DNA methylation differences between analyses (**Supplementary Figures 11-14**). This sensitivity analysis implies that although additional confounders potentially exist in our dataset, the identified schizophrenia-associated DMPs are relatively robust to the major PCs associated with methylomic variance.

We next used *comb-p*([27](#_ENREF_27)) (see **Materials and Methods**) to identify spatially correlated regions of differential DNA methylation significantly associated with schizophrenia (Šidák-corrected *P* < 0.05, number of consecutive probes ≥ 2) in each of the four brain regions (PFC: 13 DMRs spanning an average of 7 probes and 279bp; STR: 2 DMRs spanning an average of 8 probes and 328bp; HC: 1 DMRs spanning an average of 7 probes and 260bp; CER: 10 DMRs spanning an average of 5 probes and 246bp) (**Figure 2** and **Table 3**). ([28](#_ENREF_28))([29](#_ENREF_29))Many DMRs are again characterized by consistent schizophrenia-associated differences in DNA methylation across multiple brain regions (**Figure 2**). Of note, a DMR spanning four probes within the *RPH3AL* gene on chromosome 17, which encodes a protein that plays a direct regulatory role in calcium-ion-dependent exocytosis, is consistently hypomethylated in schizophrenia patients across all four brain regions (PFC: median DNA methylation difference = -8.03%, median *P =* 8.27E-04; STR: median DNA methylation difference = -5.32%, median *P =* 7.14E-03; HC: median DNA methylation difference = -7.84%, median *P =* 1.78E-02; CER: median DNA methylation difference = -10.24, median *P =* 3.86E-03) (**Figure2** and **Supplementary Figures 15-16**). ([30](#_ENREF_30))([31](#_ENREF_31))We subsequently confirmed schizophrenia-associated hypomethylation across the same region using *Bumphunter* ([32](#_ENREF_32)) - an alternative DMR analysis approach – in the PFC (*P =* 4.85E-05), STR (*P =* 2.00E-04) and CER (*P* = 6.01E-06). To validate the differences identified across this DMR using the Illumina 450K array we employed bisulfite-pyrosequencing to quantify DNA methylation across an amplicon spanning three CpG sites (including cg11940040 and two adjacent CpG sites not on the 450K array) in the PFC (n = 35 schizophrenia and 36 controls) and STR (n = 36 schizophrenia and 41 controls) samples. All three sites were significantly hypomethylated in schizophrenic patients compared to controls in both brain regions (**Supplementary Table 6** and **Supplementary Figures 15-16**), with DNA methylation differences reflecting those identified using the 450K array (PFC: average DNA methylation difference = -8.68%, *P =* 1.72E-03; STR: average DNA methylation difference = -5.33%, *P =* 1.41E-02).

*Consistent methylomic markers of schizophrenia across brain regions*

We next employed a multi-level model to further explore consistent schizophrenia-associated differences across multiple brain regions (see **Material and Methods**). As reported in previous analyses of epigenetic variation in the human brain ([33-35](#_ENREF_33)), our data show that at a global level the patterns of DNA methylation in the CER are very distinct to the other three brain regions included in this study (**Supplementary Figure 17**); for this reason we excluded the CER from our multi-region model and focused on identifying consistent signals across the PFC, STR and HC. Of note, there is inflation in the distribution of *P*-values in the multi-region case-control analysis (λ = 1.43, **Supplementary Figure 9E**); although our model is designed to control for the non-independence of brain regions from the same individual, it is possible that combining datasets has resulted in some residual inflation. Compared to other published EWAS analyses, however, this inflation is relatively modest and we do not identify an excessively large number of DMPs passing our stringent family-wise significance threshold. **Supplementary Table 7** and **Supplementary Figure 18** lists the fifty top-ranked cross-region DMPs, with significant cross-region DMRs listed in **Supplementary Table 8** and **Supplementary Figure 19**; in total we identified 22 DMRs spanning an average of 6 probes and 317bp. The multi-region model DMPs provide further support for several loci identified in our previous study of schizophrenia prefrontal cortex ([17](#_ENREF_17)) including *GSDMD* (cg26173173: *P =* 4.28E-05), *RASA3* (cg24803255: *P =* 1.51E-04), *PPFIA1* (cg08171022: *P =* 1.19E-02) and *MYT1L* (cg00236305: *P =* 4.62E-04) (**Supplementary Table 9**), suggesting that DNA methylation differences at these loci are consistently-associated with schizophrenia across the three regions. Of note, the top-ranked cross-region DMRs include a highly-significant signal spanning 11 CpG sites (Šidák-corrected *P =* 8.90E-11) annotated to *WNT5A*([36](#_ENREF_36)), in addition to regions annotated several loci identified in the analyses of the different brain regions such as *GBP4* (Šidák-corrected *P =* 0.01), *PRDM9* (Šidák-corrected *P =* 0.04) and *RPH3AL* (Šidák-corrected *P =* 1.23E-05).

*Methylomic variation associated with schizophrenia polygenic risk score (PRS)*

A recent large-scale GWAS of schizophrenia identified 128 independent associations spanning 108 genomic regions in a meta-analysis of over 80,000 samples([8](#_ENREF_8)). 5006, 5058, 5066 and 4951 Illumina 450K array probes included in our PFC, STR, HC and CER analyses, respectively, are located within these broad genomic regions. Although a number of probes within these regions were nominally associated with schizophrenia (see **Supplementary Table 10** for all DMPs with *P* < 1.00E-03), we found no overall enrichment of DMPs in any of the analyses performed (Fisher’s exact test: PFC *P =* 0.20, STR *P =* 0.27, HC *P =* 0.59, CER *P =* 0.55, multi-region model *P =*0.04) at a Bonferroni corrected *P*-value for the number of tests performed (*P* < 1.25x10^-2^).

Beyond the specific genome-wide significant loci identified in GWAS, an individual’s accumulated genetic burden can be quantified to define an overall PRS – i.e. the sum of trait-associated alleles across many genetic loci, weighted by effect sizes estimated by GWAS analyses ([8](#_ENREF_8), [37](#_ENREF_37)). We next explored if an increased burden of polygenic variants associated with schizophrenia was itself associated with variation in DNA methylation in the brain. Each sample was genotyped and SNP data was imputed using the latest data release from the 1,000 Genomes project, and a PRS for each sample was generated using data from the recent schizophrenia GWAS([8](#_ENREF_8)) (see **Materials and Methods**). None of the samples used in this study were included in the PGC GWAS analysis of schizophrenia, and thus did not contribute to defining the variants included in the PRS. To avoid population stratification effects, ethnicity was determined using data from HapMap Phase 3 (see **Materials and Methods**) and non-Caucasian samples (n = 10) were excluded from subsequent PRS-based analyses. Despite the relatively small sample size (see **Supplementary Table 1** for an overview of samples), schizophrenia patients (n = 34) were characterized by a significantly higher PRS than controls (n = 40) (*P =* 4.42E-03) (**Figure 3A**). For Caucasian samples, we repeated our case-control study with and without the inclusion of PRS as a covariate. For the top-ranked DMPs associated with schizophrenia (presented above), there was a highly-significant correlation of both schizophrenia-associated DNA methylation difference and *P*-value across all four brain regions (correlations ranging from 0.80 to 1.00 for all comparisons, except for the HC (0.56-1.00)), indicating that polygenic risk burden is not impacting greatly on the schizophrenia-associated differences identified.

We next employed a linear model, controlling for age, sex, and neuronal estimates (except in the CER, as described in **Materials and Methods**) to identify methylomic variation associated with the schizophrenia PRS. Q-Q plots for the *P*-values of the analyses in each tissue are shown in **Supplementary Figure 20**, again providing evidence of some *P-*value inflation (PFC λ = 0.96, STR λ = 1.10, HC λ = 1.17, CER λ = 1.26) in some brain regions. The 15 PRS-associated DMPs passing our stringent significance threshold listed in **Table 4** and the fifty top-ranked PRS-associated DMPs in each brain region presented in **Supplementary Figures 21-24** and **Supplementary Tables 11-14**. Results for all probes included in the analysis of PRS can be downloaded from <http://epigenetics.essex.ac.uk/schizobrain/>. The top-ranked PRS-associated DMP is cg20640266 (annotated to the zinc-finger gene *ZNF618*), at which an increased polygenic burden was associated with elevated DNA methylation in the CER (*P =* 6.50E-7) (**Figure 3B**). Although the specific top-ranked PRS-associated loci in each tissue are distinct, effect sizes at PRS-associated DMPs are significantly correlated across brain regions (**Supplementary Table 15** and **Supplementary Figure 25**), with the exception of the HC where the low number of samples (n = 23) means we are probably underpowered to detect robust effects. Furthermore, although there is no direct overlap between the top-ranked schizophrenia-associated and PRS-associated DMPs, the effect sizes at the top-ranked PRS-associated probes are significantly correlated with those at the same sites in the case-control analysis, and vice versa, across all brain regions (**Supplementary Figures 26-29**). We used identified spatially correlated regions of differential DNA methylation significantly associated with polygenic burden for schizophrenia (Šidák-corrected *P* < 0.05, number of consecutive probes ≥ 2). PRS-associated DMRs in each of the four brain regions are listed in **Table 5** and **Figure 4** (PFC: 6 DMRs spanning an average of 6 probes and 291bp; STR: 4 DMRs spanning an average of 5 probes and 237bp; CER: 10 DMRs spanning an average of 6 probes and 340bp; no DMRs were identified in the HC).

We next employed a multi-level model to identify consistent PRS-associated DMPs (**Supplementary Figure 30** and **Supplementary Table 16**) and DMRs (**Supplementary Figure 31** and **Supplementary Table 17** across PFC, STR and HC. Of note, the top-ranked PRS-associated DMP (cg04910228), at which PRS is negatively correlated with DNA methylation (estimate = -0.38%, *P =* 6.50E-07), is located within the *TSNAX-DISC1* locus on chromosome 1 (**Figure 3C**). A balanced translocation involving this gene that segregates with several major psychiatric disorders including schizophrenia has been intensively studied in a Scottish pedigree([5](#_ENREF_5)), although the involvement of this locus in the etiology of the disorder remains controversial and common genetic variation in this region was not identified in recent GWAS analyses([40](#_ENREF_40), [41](#_ENREF_41)). Our data suggest that an increased polygenic burden for schizophrenia may impact upon regulatory variation of the *DISC1* locus in the brain.

*Polygenic risk score-associated methylomic variation does not reflect direct genetic effects on DNA methylation*

Although one of the top-ranked PRS-associated DMPs was located within a GWAS-nominated genomic region - cg01682070 (annotated to *TAOK2* on chromosome 16), at which DNA methylation was positively correlated with PRS (*P =* 4.30E-08) - we found no significant enrichment of PRS-associated DMPs within GWAS-nominated schizophrenia-associated regions (Fisher’s exact test: PFC *P =* 0.83, STR *P =* 0.53, HC *P =* 0.24, CER *P =* 0.53). We next characterized methylation quantitative trait loci (mQTLs) associated with the 99,904 variants included in the PRS calculation, using linkage disequilibrium (LD)-pruned independent SNPs and a genome-wide mQTL significance threshold of *P <* 3.69E-13, as described in Hannon *et al*., 2016([34](#_ENREF_34)). Given the low number of samples from the HC, mQTL analyses were not performed for this brain region. In total we identified 255 associations between genetic variants and DNA methylation sites in the PFC, representing 198 unique SNPs (**Supplementary Table 18**), with 247 mQTL pairs (representing 201 independent SNPs) identified in the STR (**Supplementary Table 19**) and 282 mQTL pairs (representing 219 independent SNPs) identified in the CER (**Supplementary Table 20**). None of the top-ranked PRS-associated DMPs in any of the individual brain regions, in addition to those identified in the multi-region model, were significantly associated with any of the genetic variants included in the PRS calculation. Because it is possible that weaker-effect mQTLs may still underlie some of the PRS-associated epigenetic variation, we subsequently relaxed our mQTL significance threshold to *P* < 1.00E-10, again finding no overlap with PRS-associated DMPs (**Supplementary Figure 32**). Together, these data indicate that PRS-associated epigenetic variation does not directly result from genetic influences on DNA methylation in any of the brain regions tested.

**DISCUSSION**

In this study we quantified genome-wide patterns of DNA methylation in postmortem brain samples isolated from PFC, STR, HC and CER obtained from two independent cohorts of schizophrenia patients and controls. We identified numerous DMPs and DMRs associated with disease and polygenic risk burden; many of these loci were differentially methylated in individual brain regions although others showed consistent patterns across brain regions. Many of the DMPs and DMRs associated with increased genetic burden for schizophrenia are independent of the changes observed in the disease itself, and there is no evidence for direct genetic effects on DNA methylation (i.e. via mQTLs) for variants included in the PRS. Overall, our study represents the first analysis of epigenetic variation associated with schizophrenia across multiple brain regions and highlights that DNA methylation in the brain is robustly associated with the polygenic risk burden, independently of many of the changes observed in the disease itself.

Genes annotated to several of the schizophrenia-associated DMPs and DMRs have been previously implicated in the pathophysiology of schizophrenia or have relevant roles in brain function; such as *NCAM1* (which encodes a neural cell adhesion molecule with a well-established role in neurodevelopment and synaptic plasticity)([22](#_ENREF_22), [23](#_ENREF_23)), *SYNPO* (which encodes a actin-associated protein that is associated with postsynaptic densities and dendritic spines and differentially expressed in schizophrenia brain)([24](#_ENREF_24)), *GBP4* (that encodes a gene that has been found to be differentially expressed in schizophrenia patients)([25](#_ENREF_25)), *PRDM9* (which encodes a protein with histone H3K4 trimethyltransferase activity during meiosis that has been previously hypothesized to play a role in schizophrenia)([28](#_ENREF_28), [29](#_ENREF_29)) and *WNT5A* (an important neurodevelopmental locus)([36](#_ENREF_36)). Of note, a DMR spanning four probes within the *RPH3AL* gene (which encodes a protein that plays a direct regulatory role in calcium-ion-dependent exocytosis), was consistently hypomethylated in schizophrenia patients across all four brain regions. Interestingly, this DMR is also functionally annotated to the *DOC2B* gene using the Genomic Regions Enrichment of Annotation Tool (GREAT) ([30](#_ENREF_30)); this encodes a high-affinity Ca^2+^ sensor involved in spontaneous neurotransmitter release from synaptic vesicles ([31](#_ENREF_31)). ([38](#_ENREF_38))([39](#_ENREF_39))Most notably, the top-ranked probe associated with PRS in our multi-region model (i.e. across PFC, STR and HC) is located in the gene body of *DISC1*, a gene previously strongly linked to schizophrenia in a Scottish pedigree with a balanced translocation spanning the locus([5](#_ENREF_5)). Our data suggest that an increased PRS for schizophrenia may impact upon regulatory variation of the *DISC1* locus in the brain, implicating a potentially common pathway between polygenic and highly penetrant single locus etiologies that warrants further investigation.

Despite this being the first study to quantify DNA methylation across four different brain regions from schizophrenia patients and controls, this study has a number of important limitations. First, the number of samples assessed in this study is relatively low, especially for analyses involving the HC, which was only available from one of the two brain-bank cohorts. Despite this, we were able to identify a number of DMPs and DMRs passing our stringent significance thresholds in both the analyses of diagnosed schizophrenia and polygenic risk burden. Furthermore, although the magnitude of change at the differentially methylated loci was relatively small (i.e. involving a relative small proportion of cells in a given brain region), we were able to technically validate the Illumina 450K array data using bisulfite-pyrosequencing. Of note, given the relatively small number of individual donors, the PRS analyses were undertaken in both cases and controls, and therefore our study design is potentially confounded in a way that makes it not completely independent from the schizophrenia analysis. Despite this, we observed no direct overlap between the top-ranked schizophrenia-associated and PRS-associated DMPs although effect sizes were correlated.

Second, because epigenetic processes play an important role in defining cell-type-specific patterns of gene expression([42-44](#_ENREF_42)), the use of bulk tissue from each brain region is a potential confounder in DNA methylation studies([45](#_ENREF_45), [46](#_ENREF_46)). Despite our efforts to control for the effect of cell type diversity in DNA methylation quantification in our analyses using *in silico* approaches, this approach is not suitable to estimate the neuronal proportion in the cerebellum and cannot inform us about disease relevant DNA methylation changes specific to individual brain cell types. Third, there is increasing awareness of the importance of 5-hydroxymethyl cytosine (5-hmC) in the human brain([47](#_ENREF_47), [48](#_ENREF_48)), although this modification cannot be distinguished from DNA methylation using standard bisulfite-based approaches([49](#_ENREF_49)). It is therefore plausible that many of the differences identified in this study are confounded by modifications other than DNA methylation. To date, no study has evaluated the role of 5-hmC in schizophrenia or any other psychiatric disorder, although a recent paper from our group quantified levels of 5-hmC across the genome in human cortex and cerebellum ([49](#_ENREF_49)); of note, none of the significant DMPs identified in this study were characterized by detectable levels of this DNA modification.

Definitively distinguishing cause from effect in epigenetic epidemiology is difficult, especially for disorders like schizophrenia that manifest in inaccessible tissues such as the brain and are therefore particularly refractory to longitudinal study([46](#_ENREF_46)). However, our observation of consistent changes across multiple brain regions in two independent cohorts for many DMPs and DMRs suggests that the identified loci are potentially directly relevant to the schizophrenia pathogenesis. Furthermore, our identification of PRS-associated variation in DNA methylation potentially less confounded by medication intake and other disease-associated exposures that can influence case-control analyses. We tested if the PRS associations reflected the direct effects of genetic variation by testing whether the genetic variants used to derive the polygenic risk scores are mQTLs that influence DNA methylation at PRS-associated DMPs; our analyses suggest that the associations with schizophrenia polygenic burden are independent from genetic variation itself.

Unlike for GWAS, little work has been done to determine appropriate levels of significance in EWAS and a major issue in the field of epigenetic epidemiology is that no empirically-derived thresholds have been established that can be used consistently across studies (Mill & Heijmans, Nature Reviews Genetics, 2014). To establish a stringent multiple-testing significance threshold to identify schizophrenia-associated DMPs, we utilized data from a large Illumina 450K dataset (n = 675 individuals) generated as part of recent study from our group ([50](#_ENREF_50)). Briefly, we performed 5,000 EWAS permutations using a multiple linear regression model controlling for age, sex, smoking and cell composition, and used these to estimate the nominal *P*-value for 5% family-wise error (*P =* 1.66E-07). Although we believe this approach to be highly stringent, providing a significance threshold that can be used in subsequent EWAS analyses, it is important to note that the permutations were performed in an independent dataset.

Although we controlled for age, sex and derived neuronal composition in our analyses, it is plausible that other factors may be confounding our case-control analyses of schizophrenia, as highlighted by the inflated Q-Q plots observed for several of the analyses. For example, epidemiological data highlights a much higher rate of smoking in schizophrenia patients compared to unaffected controls([51](#_ENREF_51), [52](#_ENREF_52)). Although smoking has been shown to have striking effects on DNA methylation in blood([53](#_ENREF_53)), none of the robust smoking-associated DMPs identified in blood are amongst the schizophrenia DMPs identified in any of the four brain regions assessed in the current study. Although *P*-value inflation is a common feature of many DNA methylation datasets, standard genomic control methods – widely used in GWAS – are not suitable for EWAS data ([26](#_ENREF_26))). Therefore, we investigated the impact of including additional surrogate variables capturing variation in DNA methylation on the association statistics for schizophrenia-associated DMPs (**Supplementary Figures 11-14**), observing that the identified schizophrenia-associated DMPs are relatively robust to the major PCs associated with methylomic variance. Of course, the modest *P*-value inflation observed in this study does not necessarily result from residual confounding; it is plausible that there are multiple differentially methylated loci of small effect associated with schizophrenia, and that changes across genomic regions are coordinated. Finally, although the control samples used in this study were selected to be free of psychiatric morbidity, little additional information about these donors is available; given the nature of post-mortem tissue, for example, they will have died from a number of different causes. Although our study presents novel evidence for associations between schizophrenia diagnosis, schizophrenia polygenic burden and variable DNA methylation across different brain regions, further replication using larger sample sizes is essential to further support these results. Future studies should focus on understanding the transcriptional consequences of the observed associations, and testing whether these associations are causal or a consequence of disease and/or medication.

In summary, our data provide evidence for differences in DNA methylation across multiple brain regions in schizophrenia. We also identify evidence for differential DNA methylation associated with increased polygenic burden for schizophrenia, including in the vicinity of *DISC1*, a gene previously implicated in the disease by a highly penetrant balanced translocation. Of note, there is no enrichment of loci identified in a recent large GWAS of schizophrenia amongst DMPs for either schizophrenia or schizophrenia PRS identified in this study. Our study represents the first analysis of epigenetic variation associated with schizophrenia across multiple brain regions and highlights the utility of polygenic risk scores for identifying molecular pathways associated with etiological variation.

**MATERIALS AND METHODS**

*Post-mortem tissue samples*

Post-mortem PFC (Brodmann area 9), STR (putamen), HC, and CER samples from a total of 41 schizophrenia patients and 47 non-psychiatric control samples were obtained from the MRC London Neurodegenerative Diseases Brain Bank (LNDBB) (<http://www.kcl.ac.uk/ioppn/depts/bcn/Our-research/Neurodegeneration/brain-bank.aspx>) and the Douglas-Bell Canada Brain Bank (DBCBB), Montreal ([http://douglasbrainbank.ca/](http://douglasbrainbank.ca/%20)). LNDBB subjects were approached in life for written consent for brain banking, and all tissue donations were collected and stored following legal and ethical guidelines (NHS reference number 08/MRE09/38; LBBND brain bank HTA license number 12293). Schizophrenia patients were diagnosed by trained psychiatrists according to Diagnostic and Statistical Manual of Mental Disorders criteria. DBCBB samples were collected post-mortem following consent obtained with next of kin, according to tissue banking practices regulated by the Quebec Health Research Fund (<http://ethique.msss.gouv.qc.ca/>), and based on the OECD Guidelines on Human Biobanks and Genetic Research Databases (<http://www.oecd.org/science/biotech/44054609.pdf>). Psychiatric diagnoses were based on best-estimate diagnostic procedures, following SCID I diagnostic interviews conducted with informants, as described elsewhere([54](#_ENREF_54)). The current study was approved by the University of Exeter Medical School Research Ethics Committee (reference number 13/02/009). All samples were dissected by trained neuropathologists from each brain bank, snap-frozen and stored at -80°C.

*Methylomic profiling*

Genomic DNA was isolated using a standard phenol-chloroform extraction protocol and assessed for quality and purity using spectrophotometry. DNA (~500 ng) from each sample was treated with sodium bisulfite using the EZ-96 Gold DNA methylation kit (Zymo Research, Irvine, CA, USA). DNA methylation was quantified using the Illumina Infinium HumanMethylation450 BeadChip (Illumina, San Diego, CA, USA) scanned on an Illumina HiScan System (Illumina, San Diego, CA, USA). Samples were batched by tissue and brain-bank, and randomized with respect to diagnosis, sex and age throughout all experimental procedures. Illumina Genome Studio software was used to extract the raw signal intensities of each probe (without background correction or normalization). QC and normalization steps were performed separately for samples from each brain bank. Signal intensities for each probe were imported into R([55](#_ENREF_55)) using the *methylumi* and *minfi* packages([56](#_ENREF_56), [57](#_ENREF_57)). Multidimensional scaling plots of sex chromosome probes were used to check that the predicted sex corresponded with the reported sex for each individual. The ten bisulfite conversion control probes on the array were used to calculate the efficiency of the bisulfite conversion reaction. Comparison of 65 SNP probes on the array confirmed that matched tissues were sourced from the same individual. The 65 SNP probes, probes on sex chromosomes, cross-hybridizing probes([58](#_ENREF_58), [59](#_ENREF_59)) and probes containing a SNP with minor allele frequency > 5% within 10 bp of the single base extension position were excluded from analysis([58](#_ENREF_58)). The ‘pfilter’ function of the *wateRmelon* package([60](#_ENREF_60)) was used to filter data by beadcount and detection *P*-value. Samples with > 1% probes with a detection *P*-value > 0.01 were removed, along with probes with a detection *P*-value > 0.05 in at least 1% of samples and/or a beadcount < 3 in 5% of samples were also removed. The ‘dasen’ function in *wateRmelon* was used to normalize the data as previously described([60](#_ENREF_60)). The total number of CpG sites included and excluded for each brain region in the final dataset are presented in **Supplementary Table 21**. In total 5 PFC, 4 STR, 3 HC and 4 CER samples were excluded during these stringent QC procedures. The number of samples in the final dataset used in the analyses are presented in **Table 1**.

*Genotyping and derivation of schizophrenia polygenic risk scores*

Genomic DNA (200 ng) from each individual was used for genotyping on the Illumina Infinium HTS HumanOmniExpress-24 BeadChip v1-0 using an iScan Microarray Scanner (Illumina, San Diego, CA, USA), according to manufacturer’s instructions. Illumina GenomeStudio software was used for genotype calling and the data were exported as .ped and .map files. PLINK([61](#_ENREF_61)) was used to remove samples with > 5% missing data and SNPs with > 1% missing values, Hardy-Weinberg equilibrium *P* < 1.00E-03 or minor allele frequency of < 5%. Sample ethnicity was determined by merging the genotypes with data from HapMap Phase 3 (<http://www.sanger.ac.uk/resources/downloads/human/hapmap3.html>) and LD pruning the overlapping SNPs such that no pair of SNPs within 1500 bp had r^2^ > 0.20. GCTA software([62](#_ENREF_62)) was used to calculate principal components of the genetic data, which were visually inspected to ascertain ethnicity for each sample by comparison with the known ethnicities of the HapMap sample. Non-Caucasian samples (n = 10) were excluded from PRS analyses. For imputation, genotypes were recoded into .vcf files using PLINK1.9([63](#_ENREF_63)) and VCFtools([64](#_ENREF_64)) before uploading to the Michigan Imputation Server (<https://imputationserver.sph.umich.edu/start.html#!pages/home>), which uses SHAPEIT([65](#_ENREF_65)) to phase haplotypes, and Minimac3([66](#_ENREF_66)) with the most recent 1000 Genomes reference panel (phase 3, version 5) (<http://www.1000genomes.org/>). PRSs were calculated in PLINK([61](#_ENREF_61)) using the imputation dosages from 99940 variants and the score file downloaded from the Psychiatric Genomics Consortium (PGC) website (<https://www.med.unc.edu/pgc/downloads>) where GWAS results have been clumped, retaining the best association (identified by *P*-value) in each LD block.

*Identification of schizophrenia-associated differentially methylated positions and regions*

An overview of the samples included in our schizophrenia case-control analysis is given in **Table 1**. We estimated the proportion of neuronal cells for each sample using the CETS package in R([45](#_ENREF_45)). To identify DMPs in each brain region we used linear regression with the preprocessed and normalized methylation (β) values separately for samples from each brain bank using disease status, age, sex and neuronal proportion estimates as independent variables. Given the nature of the samples used in this study, information about medication, smoking status and other phenotypic information was not available and could not be included as covariates in analyses. Neuronal proportion estimates were not included as a variable for cerebellum samples because of the high proportion of non-NeuN-expressing neurons, which make CETS unsuitable for estimating cell composition. In our data, cerebellum neuronal estimates derived from CETS correlated significantly with age (ρ = 0.48, *P =* 1.26E-05) reflecting the previously reported age-associated variance in the ratio of NeuN-expressing and non-expressing cells in the cerebellum([45](#_ENREF_45)). For tissues collected from both brain banks (PFC, STR and CER) a fixed-effect meta-analysis on the adjusted mean β values computed with inverse variance weights was performed using the ‘metacont’ from the *meta* package in R([67](#_ENREF_67)). Only probes that survived QC and were common to both datasets were used in the meta-analysis (**Supplementary Table 23**). We employed a fixed-effects (rather than random-effects) meta-analysis because with only two sample cohorts contributing to the pooled effect size, the precision of the estimate of the between-studies variance is poor using a random-effects model. To identify DMRs, we identified spatially correlated *P*-values in our data using the Python module *comb-p*([27](#_ENREF_27)) to group spatially correlated DMPs (seed *P*-value < 1.00E-03, minimum of 2 probes) at a maximum distance of 300 bp in each brain tissue. DMR *P*-values were corrected for multiple testing using Šidák correction([68](#_ENREF_68)) which corrects the combined *P* for n_a_/n_r_ tests, where n_a_ is the total number of probes tested in the initial EWAS and n_r_ the number of probes in the given region. The Bioconductor package *Bumphunter* ([32](#_ENREF_32)) was used to confirm specific DMRs identified by *comb-p* with an alternative method*.* The probes common to the PFC, STR and HC analyses (411,449 probes) were tested for homogeneous DNA methylation effects associated with schizophrenia across the three brain regions using a mixed-effects model with sex, age, neuronal estimates and brain bank as fixed effects and individual and brain region as random effects.

*Identification of polygenic risk score-associated differentially methylated positions and regions*

Although the utility of PRS for exploring the molecular genomic mechanisms involved in disease pathogenesis is largely unexplored, PRS-associated epigenetic variation is potentially less affected by factors associated with the disease itself (e.g. medication exposure, stress, and smoking), which can confound case-control analyses. An overview of the samples included in our analysis of methylomic variation associated with polygenic burden for schizophrenia is given in **Supplementary Table 1**. To identify PRS-associated DMPs we performed a multiple linear regression for each cohort using the PRS, age, sex and neuronal proportion estimates as independent variables (except in the CER, where neuronal proportion estimates were not included, as described above). For each of the tissues collected from both brain banks (PFC, STR and CER), a fixed-effect meta-analysis based on the linear regression estimates and their standard errors was computed with inverse variance weights using the ‘metagen’ function from the *meta* package in R([67](#_ENREF_67)). Only probes that passed our QC metrics and were common to both cohorts in each brain region were used in the meta-analysis (**Supplementary Table 21**). To identify PRS-associated DMRs we used *comb-p*([27](#_ENREF_27)) as described above. To identify homogeneous DNA methylation effects associated with PRS across PFC, STR and HC data a mixed effects model was fitted as described above.

*Establishing multiple testing significance threshold for EWAS analysis*

To establish a stringent multiple-testing significance threshold to identify schizophrenia-associated DMPs, we utilized data from a large Illumina 450K dataset (n = 675 individuals) generated as part of recent study from our group ([50](#_ENREF_50)). The sample was randomly split into cases and controls 5,000 times, and for each permutation an EWAS was performed using a multiple linear regression model controlling for age, sex, smoking and cell composition, and the probe-level *P*-values recorded. The minimum or most significant *P*-value was identified for each permutation and the 5^th^ percentile across the permutations was used to estimate the nominal *P*-value for 5% family-wise error (*P =* 1.66E-07).

*Targeted validation using bisulfite-pyrosequencing*

Bisulfite-pyrosequencing was used to quantify DNA methylation across the chr17:154410-154672 region identified in our DMR analysis. The bisulfite-pyrosequencing assay was designed using the PyroMark Assay design software (Qiagen, Hilden, Germany), with bisulfite-PCR amplification performed in duplicate using the primers and assay conditions in **Supplementary Table 22**. Fully methylated control samples were included in all experiments. DNA methylation was quantified across amplicons using the Pyromark Q24 system (Qiagen) following the manufacturer's standard instructions and Pyromark Q24 CpG 2.0.6 software.

**ACKNOWLEDGEMENTS**

This work was supported by grants from the UK Medical Research Council (MRC) (grant number MR/K013807/1) and US National Institutes of Health (grant number AG036039) to JM. RP and HS were funded by MRC PhD studentships.

**CONFLICT OF INTEREST STATEMENT**

The authors declare that they have no conflict of interests.

**REFERENCES**

1 Burmeister, M., McInnis, M.G. and Zollner, S. (2008) Psychiatric genetics: progress amid controversy. *Nat Rev Genet*, **9**, 527-540.

2 World Health Organization. (2013) *The World Health Report 2013: Research for Universal Health Coverage*. World Health Organization.

3 Craddock, N., O'Donovan, M.C. and Owen, M.J. (2005) The genetics of schizophrenia and bipolar disorder: dissecting psychosis. *J Med Genet*, **42**, 193-204.

4 Xu, B., Roos, J.L., Dexheimer, P., Boone, B., Plummer, B., Levy, S., Gogos, J.A. and Karayiorgou, M. (2011) Exome sequencing supports a de novo mutational paradigm for schizophrenia. *Nat Genet*, **43**, 864-868.

5 St Clair, D., Blackwood, D., Muir, W., Carothers, A., Walker, M., Spowart, G., Gosden, C. and Evans, H.J. (1990) Association within a family of a balanced autosomal translocation with major mental illness. *Lancet*, **336**, 13-16.

6 Stefansson, H., Meyer-Lindenberg, A., Steinberg, S., Magnusdottir, B., Morgen, K., Arnarsdottir, S., Bjornsdottir, G., Walters, G.B., Jonsdottir, G.A., Doyle, O.M. *et al.* (2014) CNVs conferring risk of autism or schizophrenia affect cognition in controls. *Nature*, **505**, 361-366.

7 Purcell, S.M., Moran, J.L., Fromer, M., Ruderfer, D., Solovieff, N., Roussos, P., O'Dushlaine, C., Chambert, K., Bergen, S.E., Kahler, A. *et al.* (2014) A polygenic burden of rare disruptive mutations in schizophrenia. *Nature*, **506**, 185-190.

8 Schizophrenia Working Group of the Psychiatric Genomics, C. (2014) Biological insights from 108 schizophrenia-associated genetic loci. *Nature*, **511**, 421-427.

9 Fullard, J.F., Halene, T.B., Giambartolomei, C., Haroutunian, V., Akbarian, S. and Roussos, P. (2016) Understanding the genetic liability to schizophrenia through the neuroepigenome. *Schizophr Res*.

10 Psych, E.C., Akbarian, S., Liu, C., Knowles, J.A., Vaccarino, F.M., Farnham, P.J., Crawford, G.E., Jaffe, A.E., Pinto, D., Dracheva, S. *et al.* (2015) The PsychENCODE project. *Nat Neurosci*, **18**, 1707-1712.

11 Labrie, V., Pai, S. and Petronis, A. (2012) Epigenetics of major psychosis: progress, problems and perspectives. *Trends Genet*, **28**, 427-435.

12 Pidsley, R. and Mill, J. (2011) Epigenetic studies of psychosis: current findings, methodological approaches, and implications for postmortem research. *Biological Psychiatry*, **69**, 146-156.

13 Dempster, E., Viana, J., Pidsley, R. and Mill, J. (2013) Epigenetic studies of schizophrenia: progress, predicaments, and promises for the future. *Schizophrenia Bulletin*, **39**, 11-16.

14 Chen, C., Zhang, C., Cheng, L., Reilly, J.L., Bishop, J.R., Sweeney, J.A., Chen, H.Y., Gershon, E.S. and Liu, C. (2014) Correlation between DNA methylation and gene expression in the brains of patients with bipolar disorder and schizophrenia. *Bipolar. Disord.*, **16**, 790-799.

15 Numata, S., Ye, T., Herman, M. and Lipska, B.K. (2014) DNA methylation changes in the postmortem dorsolateral prefrontal cortex of patients with schizophrenia. *Front Genet*, **5**, 280.

16 Wockner, L.F., Noble, E.P., Lawford, B.R., Young, R.M., Morris, C.P., Whitehall, V.L. and Voisey, J. (2014) Genome-wide DNA methylation analysis of human brain tissue from schizophrenia patients. *Transl Psychiatry*, **4**, e339.

17 Pidsley, R., Viana, J., Hannon, E., Spiers, H., Troakes, C., Al-Saraj, S., Mechawar, N., Turecki, G., Schalkwyk, L.C., Bray, N.J. *et al.* (2014) Methylomic profiling of human brain tissue supports a neurodevelopmental origin for schizophrenia. *Genome Biology*, **15**, 483.

18 Jaffe, A.E., Gao, Y., Deep-Soboslay, A., Tao, R., Hyde, T.M., Weinberger, D.R. and Kleinman, J.E. (2016) Mapping DNA methylation across development, genotype and schizophrenia in the human frontal cortex. *Nature Neuroscience*, **19**, 40-47.

19 Ruzicka, W.B., Subburaju, S. and Benes, F.M. (2015) Circuit- and Diagnosis-Specific DNA Methylation Changes at gamma-Aminobutyric Acid-Related Genes in Postmortem Human Hippocampus in Schizophrenia and Bipolar Disorder. *JAMA Psychiatry*, **72**, 541-551.

20 Horvath, S. (2015) Erratum to: DNA methylation age of human tissues and cell types. *Genome Biol*, **16**, 96.

21 Horvath, S. (2013) DNA methylation age of human tissues and cell types. *Genome Biol*, **14**, R115.

22 Sunshine, J., Balak, K., Rutishauser, U. and Jacobson, M. (1987) Changes in neural cell adhesion molecule (NCAM) structure during vertebrate neural development. *Proc Natl Acad Sci U S A*, **84**, 5986-5990.

23 Ronn, L.C., Hartz, B.P. and Bock, E. (1998) The neural cell adhesion molecule (NCAM) in development and plasticity of the nervous system. *Exp Gerontol*, **33**, 853-864.

24 Focking, M., Lopez, L.M., English, J.A., Dicker, P., Wolff, A., Brindley, E., Wynne, K., Cagney, G. and Cotter, D.R. (2015) Proteomic and genomic evidence implicates the postsynaptic density in schizophrenia. *Mol Psychiatry*, **20**, 424-432.

25 Sanders, A.R., Goring, H.H., Duan, J., Drigalenko, E.I., Moy, W., Freda, J., He, D., Shi, J., Mgs and Gejman, P.V. (2013) Transcriptome study of differential expression in schizophrenia. *Hum Mol Genet*, **22**, 5001-5014.

26 van Iterson, M.M., van Zwet, E.W., Slagboom, P.E. and Heijmans, B.T. (2016) Controlling bias and inflation in epigenome- and transcriptome-wide association studies using the empirical null distribution. *bioRxiv*.

27 Pedersen, B.S., Schwartz, D.A., Yang, I.V. and Kechris, K.J. (2012) Comb-p: software for combining, analyzing, grouping and correcting spatially correlated P-values. *Bioinformatics*, **28**, 2986-2988.

28 Berg, I.L., Neumann, R., Lam, K.W., Sarbajna, S., Odenthal-Hesse, L., May, C.A. and Jeffreys, A.J. (2010) PRDM9 variation strongly influences recombination hot-spot activity and meiotic instability in humans. *Nat Genet*, **42**, 859-863.

29 Crow, T.J. (2011) 'The missing genes: what happened to the heritability of psychiatric disorders?'. *Mol Psychiatry*, **16**, 362-364.

30 McLean, C.Y., Bristor, D., Hiller, M., Clarke, S.L., Schaar, B.T., Lowe, C.B., Wenger, A.M. and Bejerano, G. (2010) GREAT improves functional interpretation of cis-regulatory regions. *Nat Biotechnol*, **28**, 495-501.

31 Groffen, A.J., Martens, S., Diez Arazola, R., Cornelisse, L.N., Lozovaya, N., de Jong, A.P., Goriounova, N.A., Habets, R.L., Takai, Y., Borst, J.G. *et al.* (2010) Doc2b is a high-affinity Ca2+ sensor for spontaneous neurotransmitter release. *Science*, **327**, 1614-1618.

32 Jaffe, A.E., Murakami, P., Lee, H., Leek, J.T., Fallin, M.D., Feinberg, A.P. and Irizarry, R.A. (2012) Bump hunting to identify differentially methylated regions in epigenetic epidemiology studies. *Int J Epidemiol*, **41**, 200-209.

33 Davies, M.N., Volta, M., Pidsley, R., Lunnon, K., Dixit, A., Lovestone, S., Coarfa, C., Harris, R.A., Milosavljevic, A., Troakes, C. *et al.* (2012) Functional annotation of the human brain methylome identifies tissue-specific epigenetic variation across brain and blood. *Genome Biol*, **13**, R43.

34 Hannon, E., Spiers, H., Viana, J., Pidsley, R., Burrage, J., Murphy, T.M., Troakes, C., Turecki, G., O'Donovan, M.C., Schalkwyk, L.C. *et al.* (2016) Methylation QTLs in the developing brain and their enrichment in schizophrenia risk loci. *Nat Neurosci*, **19**, 48-54.

35 Ladd-Acosta, C., Pevsner, J., Sabunciyan, S., Yolken, R.H., Webster, M.J., Dinkins, T., Callinan, P.A., Fan, J.B., Potash, J.B. and Feinberg, A.P. (2007) DNA methylation signatures within the human brain. *Am J Hum Genet*, **81**, 1304-1315.

36 Horigane, S., Ageta-Ishihara, N., Kamijo, S., Fujii, H., Okamura, M., Kinoshita, M., Takemoto-Kimura, S. and Bito, H. (2016) Facilitation of axon outgrowth via a Wnt5a-CaMKK-CaMKIalpha pathway during neuronal polarization. *Mol Brain*, **9**, 8.

37 International Schizophrenia, C., Purcell, S.M., Wray, N.R., Stone, J.L., Visscher, P.M., O'Donovan, M.C., Sullivan, P.F. and Sklar, P. (2009) Common polygenic variation contributes to risk of schizophrenia and bipolar disorder. *Nature*, **460**, 748-752.

38 Walters, J.T., Rujescu, D., Franke, B., Giegling, I., Vasquez, A.A., Hargreaves, A., Russo, G., Morris, D.W., Hoogman, M., Da Costa, A. *et al.* (2013) The role of the major histocompatibility complex region in cognition and brain structure: a schizophrenia GWAS follow-up. *Am J Psychiatry*, **170**, 877-885.

39 Gavin, D.P., Sharma, R.P., Chase, K.A., Matrisciano, F., Dong, E. and Guidotti, A. (2012) Growth arrest and DNA-damage-inducible, beta (GADD45b)-mediated DNA demethylation in major psychosis. *Neuropsychopharmacology*, **37**, 531-542.

40 Sullivan, P.F. (2013) Questions about DISC1 as a genetic risk factor for schizophrenia. *Mol Psychiatry*, **18**, 1050-1052.

41 Porteous, D.J., Thomson, P.A., Millar, J.K., Evans, K.L., Hennah, W., Soares, D.C., McCarthy, S., McCombie, W.R., Clapcote, S.J., Korth, C. *et al.* (2014) DISC1 as a genetic risk factor for schizophrenia and related major mental illness: response to Sullivan. *Mol Psychiatry*, **19**, 141-143.

42 Roadmap Epigenomics, C., Kundaje, A., Meuleman, W., Ernst, J., Bilenky, M., Yen, A., Heravi-Moussavi, A., Kheradpour, P., Zhang, Z., Wang, J. *et al.* (2015) Integrative analysis of 111 reference human epigenomes. *Nature*, **518**, 317-330.

43 Talens, R.P., Boomsma, D.I., Tobi, E.W., Kremer, D., Jukema, J.W., Willemsen, G., Putter, H., Slagboom, P.E. and Heijmans, B.T. (2010) Variation, patterns, and temporal stability of DNA methylation: considerations for epigenetic epidemiology. *FASEB J*, **24**, 3135-3144.

44 Varley, K.E., Gertz, J., Bowling, K.M., Parker, S.L., Reddy, T.E., Pauli-Behn, F., Cross, M.K., Williams, B.A., Stamatoyannopoulos, J.A., Crawford, G.E. *et al.* (2013) Dynamic DNA methylation across diverse human cell lines and tissues. *Genome Res*, **23**, 555-567.

45 Guintivano, J., Aryee, M.J. and Kaminsky, Z.A. (2013) A cell epigenotype specific model for the correction of brain cellular heterogeneity bias and its application to age, brain region and major depression. *Epigenetics*, **8**, 290-302.

46 Heijmans, B.T. and Mill, J. (2012) Commentary: The seven plagues of epigenetic epidemiology. *Int J Epidemiol*, **41**, 74-78.

47 Branco, M.R., Ficz, G. and Reik, W. (2012) Uncovering the role of 5-hydroxymethylcytosine in the epigenome. *Nat Rev Genet*, **13**, 7-13.

48 Kriaucionis, S. and Heintz, N. (2009) The nuclear DNA base 5-hydroxymethylcytosine is present in Purkinje neurons and the brain. *Science*, **324**, 929-930.

49 Lunnon, K., Hannon, E., Smith, R.G., Dempster, E., Wong, C., Burrage, J., Troakes, C., Al-Sarraj, S., Kepa, A., Schalkwyk, L. *et al.* (2016) Variation in 5-hydroxymethylcytosine across human cortex and cerebellum. *Genome Biol*, **17**, 27.

50 Hannon, E., Dempster, E., Viana, J., Burrage, J., Smith, A.R., Macdonald, R., St Clair, D., Mustard, C., Breen, G., Therman, S. *et al.* (2016) An integrated genetic-epigenetic analysis of schizophrenia: evidence for co-localization of genetic associations and differential DNA methylation. *Genome Biology*, **17**, 176.

51 Dalack, G.W., Healy, D.J. and Meador-Woodruff, J.H. (1998) Nicotine dependence in schizophrenia: clinical phenomena and laboratory findings. *Am J Psychiatry*, **155**, 1490-1501.

52 de Leon, J. and Diaz, F.J. (2005) A meta-analysis of worldwide studies demonstrates an association between schizophrenia and tobacco smoking behaviors. *Schizophr Res*, **76**, 135-157.

53 Elliott, H.R., Tillin, T., McArdle, W.L., Ho, K., Duggirala, A., Frayling, T.M., Davey Smith, G., Hughes, A.D., Chaturvedi, N. and Relton, C.L. (2014) Differences in smoking associated DNA methylation patterns in South Asians and Europeans. *Clin Epigenetics*, **6**, 4.

54 McGirr, A., Tousignant, M., Routhier, D., Pouliot, L., Chawky, N., Margolese, H.C. and Turecki, G. (2006) Risk factors for completed suicide in schizophrenia and other chronic psychotic disorders: a case-control study. *Schizophr Res*, **84**, 132-143.

55 R Core Team. (2015) R: A language and environment for statistical computing.

56 Aryee, M.J., Jaffe, A.E., Corrada-Bravo, H., Ladd-Acosta, C., Feinberg, A.P., Hansen, K.D. and Irizarry, R.A. (2014) Minfi: a flexible and comprehensive Bioconductor package for the analysis of Infinium DNA methylation microarrays. *Bioinformatics*, **30**, 1363-1369.

57 Davis, S., Du, P., Bilke, S., Triche, T. and Bootwalla, M. (2015) methylumi: Handle Illumina methylation data.

58 Chen, Y.A., Lemire, M., Choufani, S., Butcher, D.T., Grafodatskaya, D., Zanke, B.W., Gallinger, S., Hudson, T.J. and Weksberg, R. (2013) Discovery of cross-reactive probes and polymorphic CpGs in the Illumina Infinium HumanMethylation450 microarray. *Epigenetics*, **8**, 203-209.

59 Price, M.E., Cotton, A.M., Lam, L.L., Farre, P., Emberly, E., Brown, C.J., Robinson, W.P. and Kobor, M.S. (2013) Additional annotation enhances potential for biologically-relevant analysis of the Illumina Infinium HumanMethylation450 BeadChip array. *Epigenetics Chromatin*, **6**, 4.

60 Pidsley, R., CC, Y.W., Volta, M., Lunnon, K., Mill, J. and Schalkwyk, L.C. (2013) A data-driven approach to preprocessing Illumina 450K methylation array data. *BMC Genomics*, **14**, 293.

61 Purcell, S., Neale, B., Todd-Brown, K., Thomas, L., Ferreira, M.A., Bender, D., Maller, J., Sklar, P., de Bakker, P.I., Daly, M.J. *et al.* (2007) PLINK: a tool set for whole-genome association and population-based linkage analyses. *Am J Hum Genet*, **81**, 559-575.

62 Yang, J., Lee, S.H., Goddard, M.E. and Visscher, P.M. (2011) GCTA: a tool for genome-wide complex trait analysis. *Am J Hum Genet*, **88**, 76-82.

63 Chang, C.C., Chow, C.C., Tellier, L.C., Vattikuti, S., Purcell, S.M. and Lee, J.J. (2015) Second-generation PLINK: rising to the challenge of larger and richer datasets. *Gigascience*, **4**, 7.

64 Danecek, P., Auton, A., Abecasis, G., Albers, C.A., Banks, E., DePristo, M.A., Handsaker, R.E., Lunter, G., Marth, G.T., Sherry, S.T. *et al.* (2011) The variant call format and VCFtools. *Bioinformatics*, **27**, 2156-2158.

65 Delaneau, O., Marchini, J. and Zagury, J.F. (2012) A linear complexity phasing method for thousands of genomes. *Nat Methods*, **9**, 179-181.

66 Howie, B., Fuchsberger, C., Stephens, M., Marchini, J. and Abecasis, G.R. (2012) Fast and accurate genotype imputation in genome-wide association studies through pre-phasing. *Nat Genet*, **44**, 955-959.

67 Schwarzer, G. (2015) meta: General Package for Meta-Analysis.

68 Šidák, Z. (1967) Rectangular confidence region for the means of multivariate normal distributions. *Journal of the American Statistical Association*, **62**, 626-633.

**FIGURE LEGENDS**

**Figure 1. Forest plots showing the top-ranked schizophrenia-associated differentially methylated positions (DMPs).** Shown are data for 12 DMPs associated with schizophrenia at a highly stringent significance threshold (*P* < 1.66E-07) derived using permutations to estimate the nominal *P*-value for 5% family-wise error. Additional information on these DMPs is given in **Table 2**. Color depicts brain region in which the schizophrenia-association was identified: prefrontal cortex = blue, striatum = green, hippocampus = red, and cerebellum = yellow.

**Figure 2. Differentially methylated regions (DMRs) associated with schizophrenia.** Shown in chromosomal order are DMRs (Šidák-corrected *P* < 0.05, number of probes ≥ 2) associated with schizophrenia identified in any of the four tissues. Effect sizes for individual probes within each DMR are also shown for the other three brain regions (blue = hypomethylation, red = hypermethylation). Further details for individual DMRs are provided in **Table 3**.

**Figure 3. Increased polygenic burden for schizophrenia is associated with altered DNA methylation.** A) Schizophrenia samples included in our study scored significantly higher on a polygenic risk score derived from a recent large collaborative GWAS of schizophrenia. B) The top-ranked PRS-associated DMP was cg20640266 (annotated to *ZNF618*) in the cerebellum. C) The top-ranked multi-region PRS-associated DMP was cg04910228 (annotated to the *TSNAX-DISC1* locus).

**Figure 4. Differentially methylated regions (DMRs) associated with schizophrenia polygenic risk score (PRS).** Shown in chromosomal order are DMRs (Šidák-corrected *P* < 0.05, number of probes ≥ 2) associated with schizophrenia PRS identified in any of the four brain regions. Effect sizes for individual probes within each DMR are also shown for the other three brain regions (blue = hypomethylation, red = hypermethylation). Further details for individual DMRs are provided in **Table 5**.

**Table 1: Overview of samples included in the schizophrenia case versus control analysis.** LNDBB = MRC London Neurodegenerative Diseases Brain Bank, DBCBB = Douglas-Bell Canada Brain Bank.

|  |  |  | **N** | **Sex (male:female)** | **Age at death** | **Brain weight (g)** | **pH** |
| --- | --- | --- | --- | --- | --- | --- | --- |
| **LNDBB** | **Prefrontal cortex** | **schizophrenia** | 20 | 11:9 | 62.05 ± 15.87 | 1232.94 ± 129.22 | 6.64 ± 0.28 |
|  |  | **controls** | 23 | 17:6 | 62.04 ± 18.74 | 1368.48 ± 185.22 | 6.49 ± 0.33 |
|  |  | **total** | 43 | 28:15 | 62.05 ± 17.26 | 1310.88 ± 175.52 | 6.56 ± 0.31 |
|  |  | ***P*** | - | - | 1.00 | 0.01 | 0.13 |
|  | **Striatum** | **schizophrenia** | 21 | 11:10 | 61.76 ± 16.61 | 1227.44 ± 123.68 | 6.60 ± 0.30 |
|  |  | **controls** | 28 | 20:8 | 63.43 ± 18.16 | 1360.52 ± 184.59 | 6.46 ± 0.33 |
|  |  | **total** | 49 | 31:18 | 62.71 ± 17.36 | 1302.10 ± 172.37 | 6.53 ± 0.32 |
|  |  | ***P*** | - | - | 0.74 | 0.01 | 0.17 |
|  | **Hippocampus** | **schizophrenia** | 14 | 10:4 | 60.71 ± 15.93 | 1271.25 ± 139.22 | 6.63 ± 0.28 |
|  |  | **controls** | 13 | 11:2 | 61.92 ± 17.80 | 1415.27 ± 173.82 | 6.48 ± 0.41 |
|  |  | **total** | 27 | 21:6 | 61.30 ± 16.54 | 1340.13 ± 169.81 | 6.56 ± 0.34 |
|  |  | ***P*** | - | - | 0.85 | 0.04 | 0.31 |
|  | **Cerebellum** | **schizophrenia** | 21 | 11:10 | 61.76 ± 16.61 | 1227.44 ± 123.68 | 6.60 ± 0.30 |
|  |  | **controls** | 23 | 17:6 | 61.39 ± 19.25 | 1361.30 ± 185.03 | 6.46 ± 0.33 |
|  |  | **total** | 44 | 28:16 | 61.57 ± 17.83 | 1232.41 ± 172.79 | 6.60 ± 0.32 |
|  |  | ***P*** | - | - | 0.95 | 0.01 | 0.15 |
| **DBCBB** | **Prefrontal cortex** | **schizophrenia** | 18 | 15:3 | 45.50 ± 16.61 | 1431.78 ± 188.16 | 6.23 ± 0.22 |
|  |  | **controls** | 15 | 13:2 | 42.27 ± 14.80 | 1462.96 ± 175.17 | 6.12 ± 0.32 |
|  |  | **total** | 33 | 28:5 | 44.03 ± 15.65 | 1447.37 ± 179.32 | 6.18 ± 0.27 |
|  |  | ***P*** | - | - | 0.56 | 0.64 | 0.27 |
|  | **Striatum** | **schizophrenia** | 16 | 13:3 | 46.25 ± 17.10 | 1410.90 ± 193.35 | 6.21 ± 0.22 |
|  |  | **controls** | 17 | 14:3 | 45.65 ± 16.82 | 1438.02 ± 180.91 | 6.09 ± 0.31 |
|  |  | **total** | 33 | 27:6 | 45.94 ± 16.69 | 1426.27 ± 183.61 | 6.15 ± 0.27 |
|  |  | ***P*** | - | - | 0.92 | 0.70 | 0.23 |
|  | **Cerebellum** | **schizophrenia** | 16 | 14:2 | 44.56 ± 15.84 | 1404.62 ± 161.90 | 6.25 ± 0.22 |
|  |  | **controls** | 17 | 14:3 | 45.65 ± 16.82 | 1438.02 ± 180.91 | 6.09 ± 0.31 |
|  |  | **total** | 33 | 28:5 | 45.12 ± 16.10 | 1422.94 ± 170.58 | 6.17 ± 0.28 |
|  |  | ***P*** | - | - | 0.85 | 0.59 | 0.10 |

**Table 2. Top-ranked schizophrenia-associated differentially methylated positions (DMPs).** Shown are DMPs associated with schizophrenia at a highly stringent significance threshold (*P* < 1.66E-07) derived using permutations to estimate the nominal *P*-value for 5% family-wise error. Top-ranked schizophrenia-associated DMPs for each of the four brain regions profiled are presented in **Supplementary Tables 2-5**. Illumina and Genomic Regions Enrichment of Annotation Tool (GREAT)([30](#_ENREF_30)) annotation is listed for each DMP. LNDBB = London Neurodegenerative Disorders Brain Bank; DBCBB = Douglas-Bell Canada Brain Bank. Note hippocampus tissue was not available from the DBCBB.

| **Probe ID** | **Genomic position (hg19)** | **Illumina gene annotation** | **Genic region** | **GREAT annotation([30](#_ENREF_30))** | **Brain region** | **DNA methylation difference (%)** | ***P*** | **DNA methylation difference (%) LNDBB** | ***P*-value LNDBB** | **DNA methylation difference (%) DBCBB** | ***P*-value DBCBB** |
| --- | --- | --- | --- | --- | --- | --- | --- | --- | --- | --- | --- |
| cg08743050 | chr11:113113936 | *NCAM1* | Body | *TTC12; NCAM1* | Prefrontal cortex | -3.80 | 1.84E-08 | -1.80 | 0.15 | -4.63 | 8.39E-09 |
| cg14609448 | chr21:34896882 | *GART* | Body; 3'UTR | *DNAJC28; GART* | Cerebellum | 2.29 | 2.48E-08 | 1.37 | 0.02 | 3.11 | 3.60E-08 |
| cg08103144 | chr5:150028986 | *SYNPO* | Body | *MYOZ3; SYNPO* | Striatum | -3.17 | 3.64E-08 | 0.08 | 0.94 | -4.43 | 6.61E-11 |
| cg03847432 | chr7:43391524 | *HECW1* | Body | *STK17A; HECW1* | Striatum | 2.71 | 5.23E-08 | 1.55 | 0.04 | 3.69 | 4.76E-08 |
| cg22182016 | chr15:57998894 | *GRINL1A; GCOM1* | TSS200; Body | *POLR2M* | Striatum | -0.51 | 5.67E-08 | -0.27 | 0.14 | -0.59 | 5.41E-08 |
| cg01757160 | chr12:96588951 | *ELK3* | 5'UTR | *ELK3* | Cerebellum | 0.57 | 5.74E-08 | 0.58 | 5.13E-04 | 0.56 | 3.04e-05 |
| cg25361651 | chr20:29847402 | *DEFB115* | Body | *DEFB115; DEFB116* | Striatum | 4.43 | 6.52E-08 | 2.27 | 0.10 | 5.63 | 3.67E-08 |
| cg22221320 | chr1:89664340 | *GBP4* | Body | *GBP4* | Striatum | 6.93 | 7.88E-08 | 2.39 | 0.23 | 10.24 | 1.58E-09 |
| cg07751266 | chr16:67515323 | *ATP6V0D1* | TSS1500 | *ATP6V0D1* | Hippocampus | -1.35 | 1.27E-07 | -1.35 | 1.27E-07 | - | - |
| cg09757430 | chr13:28397122 | *-* | - | *PDX1; GSX1* | Cerebellum | -3.13 | 1.37E-07 | -2.38 | 0.02 | -3.54 | 1.65E-06 |
| cg05686445 | chr7:127636396 | *C7orf54; SND1* | TSS1500; Body | *LRRC4; SND1* | Prefrontal cortex | -3.65 | 1.63E-07 | -2.04 | 0.07 | -4.67 | 1.57E-07 |
| cg20751795 | chr19:58281019 | *ZNF586* | TSS200 | *ZNF586* | Cerebellum | 0.57 | 1.64E-07 | 0. 42 | 1.17E-06 | 0.13 | 0.03 |

**Table 23: Significant schizophrenia-associated differentially methylated regions (DMRs).** Shown in chromosomal order is the location of significant (Šidák-corrected *P* < 0.05) DMRs identified in each of the four brain regions, with the median *P-*value for DMR probes given for the other three brain regions (bold denotes median *P* < 0.05 and grey boxes denote regions that were not identified in that brain region). The ‘Gene’ column lists the combined Illumina and Genomic Regions Enrichment of Annotation Tool (GREAT) annotation([30](#_ENREF_30)).

| **Region** | **Gene(s)** | **Probes** | **N probes** | **Prefrontal cortex** | | **Striatum** | | **Hippocampus** | | **Cerebellum** | |
| --- | --- | --- | --- | --- | --- | --- | --- | --- | --- | --- | --- |
|  |  |  |  | **Median *P*** | **Šidák *P*** | **Median *P*** | **Šidák *P*** | **Median *P*** | **Šidák *P*** | **Median *P*** | **Šidák *P*** |
| chr1:25257505-25257814 | *RUNX3* | cg07996594; cg04221877; cg15014975; cg24019564; cg10993442; cg24842859; cg20695936; cg13106389; cg18087266; cg25882256; cg04250451; cg10013501 | 12 | 1.22E-02 | 1.02E-02 | 0.36 |  | 0.66 |  | 0.29 |  |
| chr1:89664260-89664583 | *GBP4* | cg23978657; cg02482460; cg22221320; cg21365602; cg20410995; cg14563196 | 6 | **8.36E-03** |  | 5.36E-04 | 3.16E-12 | 0.93 |  | 7.06E-02 |  |
| chr1:160336475-160336713 | *NHLH1* | cg18023842; cg08247612; cg13992678; cg00006397; cg00881010 | 5 | 0.24 |  | 0.62 |  | 0.42 |  | 8.00E-04 | 1.64E-05 |
| chr1:175162044-175162398 | *KIAA0040; TNN; TNR* | cg11908570; cg22626041; cg26563583; cg17839543; cg18880390; cg00099768; cg13857382; cg11973900; cg08873628; cg00321850 | 10 | 2.34E-02 | 7.62E-04 | 0.47 |  | 0.25 |  | 0.25 |  |
| chr2:241936844-241937035 | *SNED1* | cg16937168; cg03785076; cg21304158 | 3 | 0.45 |  | 0.74 |  | 0.89 |  | 5.16E-05 | 6.68E-05 |
| chr4:1202653-1202967 | *LOC100130872-SPON2; CTBP1* | cg14527262; cg04228083; cg17227257; cg16721321; cg26130533; cg14505741; cg11104416; cg18085660; cg11888738; cg01638225; cg21082272 | 11 | 3.61E-02 | 1.32E-02 | 0.41 |  | 0.66 |  | 0.72 |  |
| chr4:2627014-2627247 | *FAM193A; TNIP2; RNF4* | cg25790133; cg05083414; cg05949640; cg22980079; cg14549256; cg20163033; cg00097088 | 7 | 7.22E-03 | 2.93E-02 | 0.56 |  | 0.94 |  | 0.44 |  |
| chr5:23506738-23507031 | *PRDM9* | cg04362002; cg10589310 | 2 | 5.91E-02 |  | **3.76E-02** |  | 0.11 |  | 4.69E-06 | 3.65E-05 |
| chr5:23507450-23507657 |  | cg22054885; cg19837938; cg02444433; cg25472530; cg22079902; cg01667892 | 6 | 0.10 |  | 0.11 |  | 0.45 |  | 5.33E-04 | 5.49E-08 |
| chr6:30853948-30854234 | *DDR1* | cg16215084; cg25251478; cg26321999; cg00934322; cg07187855; cg24566261; cg09965419; cg17091577 | 8 | 2.28E-02 | 7.87E-03 | 0.75 |  | 0.73 |  | 0.21 |  |
| chr7:5535463-5535935 | *MIR589; TNRC18; FBXL18* | cg01942816; cg22108567; cg25343388; cg09286367; cg17419731; cg01024247; cg00966405; cg04155485 | 8 | 5.48E-03 | 3.44E-03 | 0.55 |  | 0.36 |  | 0.54 |  |
| chr8:145728203-145728631 | *GPT* | cg16587265; cg14476479; cg23793500; cg00280345; cg16582889; cg07658280; cg26572973; cg05241828; cg09957864; cg25600446; cg06110286; cg19352605 | 12 | 1.80E-03 | 5.53E-09 | 0.49 |  | 0.74 |  | 0.51 |  |
| chr10:124638756-124639111 | *FAM24B; LOC399815* | cg03804621; cg16299003; cg11218091; cg14708218; cg18195080; cg15252215 | 6 | **1.05E-02** |  | **4.88E-03** |  | 0.47 |  | 5.15E-03 | 4.71E-04 |
| chr10:131264786-131265074 | *MGMT* | cg26950715; cg02330106; cg12575438; cg02022136; cg23998405; cg01341123; cg25946389 | 7 | 0.13 |  | 0.54 |  | 0.83 |  | 1.01E-02 | 7.35E-03 |
| chr11:192897-193063 | *ODF3; SCGB1C1; LOC653486* | cg18793661; cg22280333; cg03960562; cg02378673; cg20297976 | 5 | 0.26 |  | 0.62 |  | 0.25 |  | 4.55E-04 | 2.08E-05 |
| chr11:34460298-34460558 | *CAT* | cg20731136; cg06027906; cg07768201; cg03720043; cg02109652; cg06908474; cg01847719 | 7 | 0.36 |  | **3.61E-02** |  | 1.00E-02 | 9.44E-04 | 0.21 |  |
| chr12:125145446-125145642 | *NCOR2; SCARB1* | cg12077664; cg27645498; cg19888509 | 3 | 2.00E-03 | 2.20E-02 | 0.56 |  | 0.59 |  | 0.18 |  |
| chr12:131452238-131452298 | *SFSWAP; GPR133* | cg24336338; cg03776878; cg23617848 | 3 | 0.80 |  | 0.23 |  | 0.59 |  | 1.20E-04 | 2.50E-03 |
| chr13:114202683-114202862 | *TFDP1; TMCO3* | cg16567723; cg24121069; cg11312353 | 3 | 6.38E-04 | 1.94E-02 | 0.12 |  | 0.74 |  | **9.18E-03** |  |
| chr16:2892518-2892913 | *PRSS30P; TMPRSS8* | cg07645761; cg00491180; cg01006802; cg27137258; cg10448227; cg10186456 | 6 | 7.18E-03 | 5.65E-03 | **3.91E-02** |  | 0.12 |  | **4.27E-03** | 1.62E-04 |
| chr17:154410-154672 | *DOC2B; RPH3AL* | cg08770870; cg11940040; cg10440639; cg23246911 | 4 | 8.27E-04 | 1.02E-04 | **7.14E-03** |  | **1.78E-02** |  | **3.86E-03** | 3.44E-02 |
| chr19:19639970-19640076 | *YJEFN3* | cg11244672; cg20098710 | 2 | 3.00E-04 | 4.49E-02 | 0.43 |  | 0.50 |  | 0.54 |  |
| chr19:57742112-57742445 | *AURKC* | cg25802888; cg19568003; cg18644286; cg26332114; cg19603903; cg23371413; cg06643849; cg25432232; cg22711741 | 9 | 0.23 |  | 1.34E-02 | 2.64E-03 | 0.86 |  | 0.27 |  |
| chr21:46898048-46898138 | *COL18A1; SLC19A1* | cg03208198; cg20383948 | 2 | 1.30E-04 | 2.31E-02 | 0.68 |  | 0.49 |  | 0.14 |  |

**Table 4: Top-ranked differentially methylated positions (DMPs) associated with schizophrenia polygenic score (PRS).** Shown are DMPs associated with the schizophrenia PRS at a highly stringent significance threshold (*P* < 1.66E-07) derived using permutations to estimate the nominal *P*-value for 5% family-wise error. Top-ranked PRS-associated DMPs for each of the four brain regions profiled are presented in **Supplementary Tables 12-15**. The methylation difference is measured per PRS unit. Illumina and Genomic Regions Enrichment of Annotation Tool (GREAT)([30](#_ENREF_30)) annotation is listed for each DMP. LNDBB = London Neurodegenerative Disorders Brain Bank; DBCBB = Douglas-Bell Canada Brain Bank.

| **Probe ID** | **Genomic position (hg19)** | **Illumina gene annotation** | **Gene region** | **GREAT annotation([30](#_ENREF_30))** | **Brain region** | **DNA methylation change (%)** | ***P*** | **DNA methylation change (%) LNDBB** | ***P*-value LNDBB** | **DNA methylation change (%) DBCBB** | ***P*-value**  **DBCBB** |
| --- | --- | --- | --- | --- | --- | --- | --- | --- | --- | --- | --- |
| cg18847009 | chr2:70175826 | *-* | - | *ASPRV1; MXD1* | Prefrontal cortex | -0.51 | 8.98E-08 | -0.50 | 7.14E-07 | -0.66 | 0.03 |
| cg26893445 | chr15:85924187 | *AKAP13* | 5'UTR | *AKAP13* | Striatum | 0.15 | 6.73E-08 | 0.16 | 6.80E-08 | 0.03 | 0.21 |
| cg12595281 | chr15:93633172 | *RGMA* | TSS1500 | *RGMA* | Striatum | 0.68 | 6.85E-08 | 0.82 | 5.36E-10 | 0.13 | 0.07 |
| cg20640266 | chr9:116811789 | *ZNF618* | Body | *AMBP; ZNF618* | Cerebellum | 0.60 | 1.62E-09 | 0.74 | 2.06E-06 | 0.32 | 0.02 |
| cg27150552 | chr7:48026856 | *SUNC1* | 3'UTR | *HUS1; SUN3* | Cerebellum | 0.34 | 1.30E-08 | 0.36 | 6.44E-09 | 0.09 | 0.68 |
| cg05209768 | chr2:164573665 | *FIGN* | Body | *KCNH7; FIGN* | Cerebellum | 0.70 | 1.55E-08 | 0.70 | 1.31E-07 | 0.70 | 0.04 |
| cg07793808 | chr12:122019006 | *KDM2B* | TSS200; TSS1500 | *KDM2B* | Cerebellum | -0.19 | 1.66E-08 | -0.21 | 2.94E-04 | -0.17 | 1.28E-05 |
| cg10218777 | chr3:133180261 | *BFSP2* | Body | *CDV3; BFSP2* | Cerebellum | 0.67 | 3.86E-08 | 0.65 | 4.24E-07 | 0.86 | 0.03 |
| cg01682070 | chr16:29996774 | *TAOK2* | Body | *HIRIP3; TAOK2* | Cerebellum | 0.32 | 4.20E-08 | 0.37 | 1.21E-08 | 0.11 | 0.38 |
| cg11786558 | chr17:2266589 | *SGSM2* | Body | *SGSM2; MNT* | Cerebellum | 0.69 | 4.35E-08 | 0.84 | 1.23E-06 | 0.52 | 4.73E-03 |
| cg26053083 | chr11:14995770 | *-* | - | *CALCA* | Cerebellum | -0.15 | 4.41E-08 | -0.15 | 2.53E-06 | -0.14 | 4.95E-03 |
| cg01022840 | chr14:71250264 | *MAP3K9* | Body | *MAP3K9; TTC9* | Cerebellum | 0.63 | 7.86E-08 | 0.66 | 7.10E-07 | 0.53 | 0.04 |
| cg08478539 | chr15:68640339 | *ITGA11* | Body | *FEM1B; ITGA11* | Cerebellum | 0.69 | 1.06E-07 | 0.80 | 3.06E-07 | 0.45 | 0.06 |
| cg23788334 | chr2:137181176 | *-* | - | *THSD7B; CXCR4* | Cerebellum | -0.13 | 1.15E-07 | -0.15 | 1.45E-05 | -0.11 | 1.56E-03 |
| cg16904520 | chr2:230590962 | *-* | - | *DNER; TRIP12* | Cerebellum | 0.26 | 1.25E-07 | 0.27 | 2.06E-06 | 0.22 | 0.02 |

**Table 5: Differentially methylated regions (DMRs) significantly associated with polygenic score (PRS) for schizophrenia.** Shown in chromosomal order is the location of significant (Šidák-corrected *P* < 0.05) DMRs identified in each of the four brain regions, with the median *P*-value for DMR probes given for the other three brain (bold denotes median *P* < 0.05 and grey boxes denote regions that were not identified in that brain region). The ‘gene’ column lists the combined Illumina and Genomic Regions Enrichment of Annotation Tool (GREAT) annotation ([30](#_ENREF_30)).

| **Region** | **Gene** | **Probes** | **N probes** | **Prefrontal cortex** | | **Cerebellum** | | **Striatum** | | **Hippocampus** | |
| --- | --- | --- | --- | --- | --- | --- | --- | --- | --- | --- | --- |
|  |  |  |  | **Median *P*** | **Šidák *P*** | **Median *P*** | **Šidák *P*** | **Median *P*** | **Šidák *P*** | **Median *P*** | **Šidák *P*** |
| chr1:84326547-84326857 | *TTLL7* | cg08882038; cg07807165; cg02531516; cg18116902; cg26347197; cg24955204; cg02483449 | 7 | 0.40 |  | 0.05 | 0.03 | 0.68 |  | 0.11 |  |
| chr1:168356537-168356674 | *TBX19; XCL2* | cg22695117; cg20678082; cg10555800; cg06122518 | 4 | **0.02** | 0.02 | 0.13 |  | 0.39 |  | 0.14 |  |
| chr2:3486706-3487165 | *ADI1; TRAPPC12* | cg14053828; cg15541040; cg15506890; cg08493051 | 4 | **0.03** |  | **2.82E-03** | 3.63E-04 | **0.03** |  | 0.84 |  |
| chr2:97405651-97405880 | *LMAN2L* | cg13915892; cg04771938; cg17340948; cg17526658; cg12930819; cg04918358; cg15007626 | 7 | 0.66 |  | **5.73E-03** | 9.07E-03 | 0.59 |  | 0.57 |  |
| chr4:74847646-74848017 | *PF4* | cg15158783; cg21043213; cg16072462; cg15398841; cg02530824; cg06834998; cg05509609; cg13126871 | 8 | **3.39E-03** | 2.10E-05 | 0.16 |  | **0.03** |  | **0.02** |  |
| chr5:102898463-102898730 | *NUDT12* | cg02976617; cg13665998; cg09166085; cg07655627 | 4 | **2.27E-03** | 0.01 | 0.08 |  | **3.91E-03** |  | 0.31 |  |
| chr5:493262-493614 | *SLC9A3; EXOC3* | cg19107578; cg25518170; cg20402284; cg25346936 | 4 | 0.76 |  | **1.17E-03** | 3.87E-04 | 0.67 |  | 0.72 |  |
| chr5:497397-497640 |  | cg22985016; cg16555556; cg14533753; cg00190355 | 4 | 0.49 |  | **5.41E-03** | 0.05 | 0.17 |  | 0.82 |  |
| chr6:30042919-30043419 | *RNF39* | cg12704854; cg11562284; cg02552311; cg03219282; cg24766429; cg23500724; cg10865856; cg24016627; cg23939808; cg12967914; cg00853042; cg23027574; cg05853632; cg22105332; cg19006429; cg27532187; cg01631162 | 17 | 0.06 | 2.99E-04 | 0.40 |  | 0.41 |  | 0.23 |  |
| chr6:33084549-33084841 | *HLA-DPB2; COL11A2* | cg03943025; cg08693832; cg27264993; cg21870640; cg08088295; cg17833071; cg23075555; cg13524302; cg02662362; cg24465429; cg24266485 | 11 | **0.03** | 0.04 | 0.11 |  | 0.19 |  | 0.23 |  |
| chr11:2907670-2907755 | *CDKN1C* | cg05090695; cg11744767; cg05559445; cg23225147 | 4 | **1.08E-03** | 1.93E-04 | 0.08 |  | 0.40 |  | 0.06 |  |
| chr12:75784617-75785098 | *GLIPR1L2* | cg14292619; cg00108944; cg23588049; cg12351126; cg02415057; cg07311024; cg02071292 | 7 | 0.66 |  | **1.27E-03** | 2.49E-07 | 0.55 |  | 0.19 |  |
| chr13:51417846-51418222 | *DLEU7; RNASEH2B; ST13P4* | cg20170533; cg08274637; cg13846270; cg27051129; cg10359157; cg20400592; cg05965387; cg17288288; cg03389701 | 9 | 0.50 |  | **8.88E-03** | 5.83E-04 | 0.31 |  | 0.28 |  |
| chr13:113540400-113540632 | *ATP11A; MCF2L* | cg17842918; cg11462099; cg26666292; cg11520003 | 4 | 0.53 |  | 0.17 |  | **2.57E-03** | 0.01 | 0.70 |  |
| chr16:66400320-66400600 | *CDH5* | cg08872742; cg02078525; cg00401972; cg00044665; cg16471830; cg22319147 | 6 | 0.68 |  | 0.56 |  | **6.43E-03** | 0.02 | 0.74 |  |
| chr19:2650727-2650864 | *GNG7; GADD45B* | cg03070741; cg01250212; cg27324541; cg10350536 | 4 | 0.43 |  | 0.65 |  | **3.07E-03** | 0.04 | 0.72 |  |
| chr19:11784514-11784956 | *ZNF833* | cg04598224; cg05950877; cg26772540; cg25394203; cg21771200; cg15209566; cg02274869 | 7 | 0.44 |  | **4.66E-03** | 1.81E-04 | 0.09 |  | 0.66 |  |
| chr19:17357315-17357642 | *NR2F6* | cg06108395; cg20981127; cg16749578; cg24057642 | 4 | 0.44 |  | **4.68E-04** | 2.12E-07 | 0.49 |  | 0.13 |  |
| chr19:47287964-47288264 | *SLC1A5; STRN4* | cg02711608; cg25607249; cg21766592; cg12165685; cg11645155; cg01406381 | 6 | 0.33 |  | 0.51 |  | **1.52E-03** | 7.62E-05 | 0.64 |  |
| chr20:30073399-30073577 | *NCRNA00028; HM1; REM1* | cg15537254; cg13159946; cg02991085; cg25502144; cg21846177 | 5 | 0.17 |  | **5.15E-04** | 2.06E-04 | 0.06 |  | 0.29 |  |
| chr20:32308081-32308344 | *PXMP4* | cg27194921; cg25092328; cg20588982; cg06231372; cg12297619; cg24270031 | 6 | **0.02** |  | **0.03** |  | **0.02** | 0.03 | 0.25 |  |
| chr22:38071534-38071678 | *LGALS1* | cg21064451; cg21737444; cg01264106; cg08835221; cg27619353; cg19853760 | 6 | 0.65 |  | **1.22E-03** | 2.30E-07 | 0.62 |  | 0.43 |  |

**ABBREVIATIONS**

| PFC | Prefrontal cortex |
| --- | --- |
| CER | Cerebellum |
| STR | Striatum |
| HC | Hippocampus |
| PRS | Polygenic risk score |
| DMP | Differentially methylated positon |
| DMR | Differentially methylated regions |
| GWAS | Genome-wide association study |
| LNDBB | MRC London Neurodegenerative Diseases Brain Bank |
| DBCBB | Douglas-Bell Canada Brain Bank |
| EWAS | Epigenome-wide association study |
| QC | Quality control |
| GREAT | Genomic Regions Enrichment of Annotation Tool |
| mQTLs | Methylation quantitative trait loci |
| MHC | Major histocompatibility complex |
|  |  |
|  |  |
|  |  |
|  |  |
|  |  |
|  |  |
|  |  |
|  |  |
