# Supplementary material for: Schizophrenia-associated methylomic variation: molecular signatures of disease and polygenic risk burden across multiple brain regions
Source: Hum Mol Genet. 2016 Dec 22;26(1):210–25. doi: 10.1093/hmg/ddw373 (PMC5351932; doi:10.1093/hmg/ddw373)
Supplement: Supplementary Data [file ddw373_Supp.zip › Supplementary Figures Final.docx]

**Supplementary Figures**

Viana et al

**Schizophrenia-associated methylomic variation: molecular signatures of disease and polygenic risk burden across multiple brain regions**

**
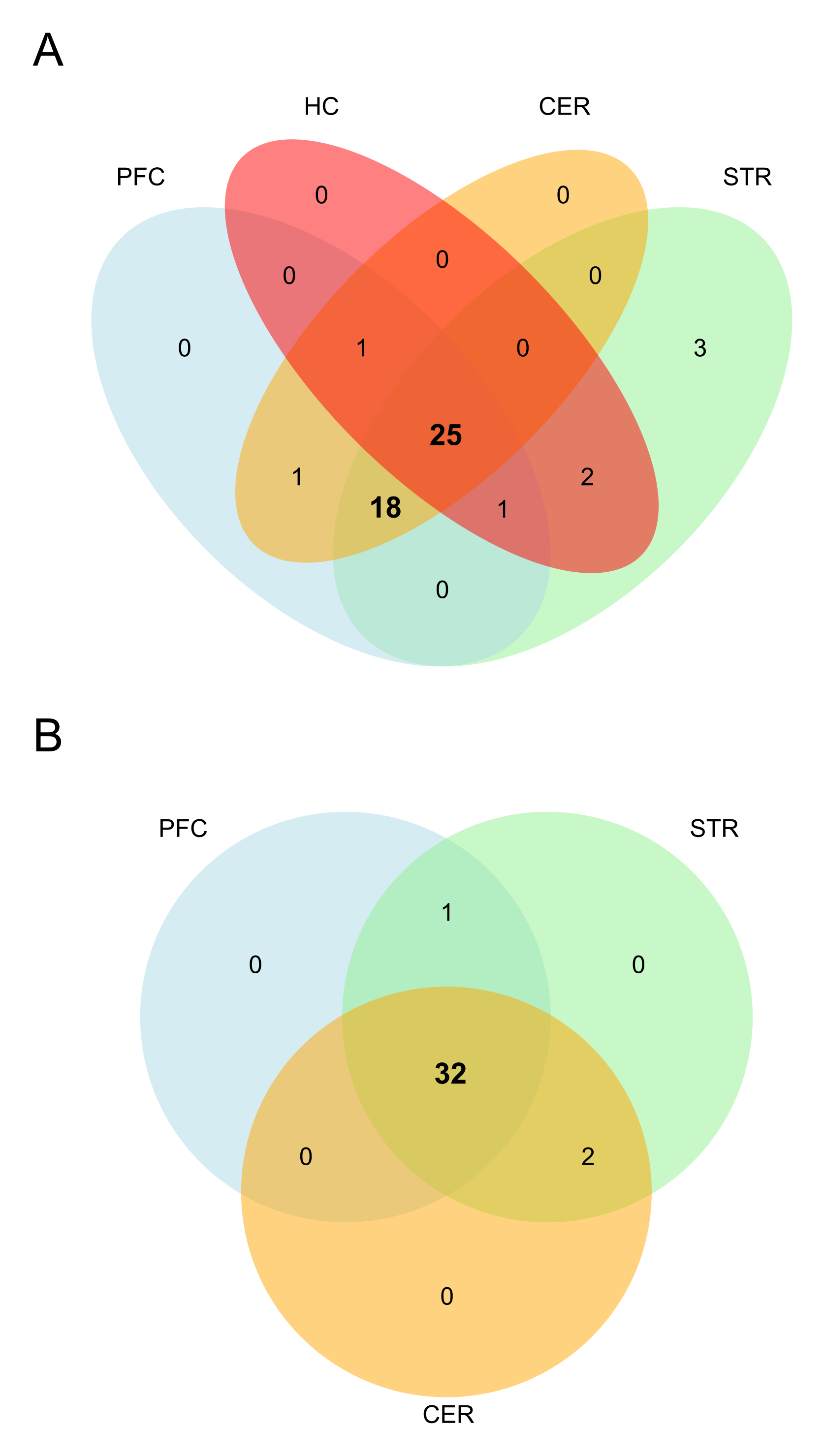
Supplementary Figure 1: An overview of the brain regions included in the final analysis dataset from each brain-bank.** Shown are details about tissues obtained from A) the MRC London Neurodegenerative Diseases Brain Bank (LNDBB) (<http://www.kcl.ac.uk/ioppn/depts/bcn/Our-research/Neurodegeneration/brain-bank.aspx>) and B) the Douglas-Bell Canada Brain Bank (DBCBB) ([http://douglasbrainbank.ca/](http://douglasbrainbank.ca/%20)). PFC = prefrontal cortex, STR = striatum, HC = hippocampus, CER = cerebellum.

**Supplementary Figure 2: Study design overview.** LNDBB = MRC London Neurodegenerative Diseases Brain Bank, DBCDD = the Douglas-Bell Canada Brain Bank, DMP = differentially methylated position, DMR = differentially methylated region.

**
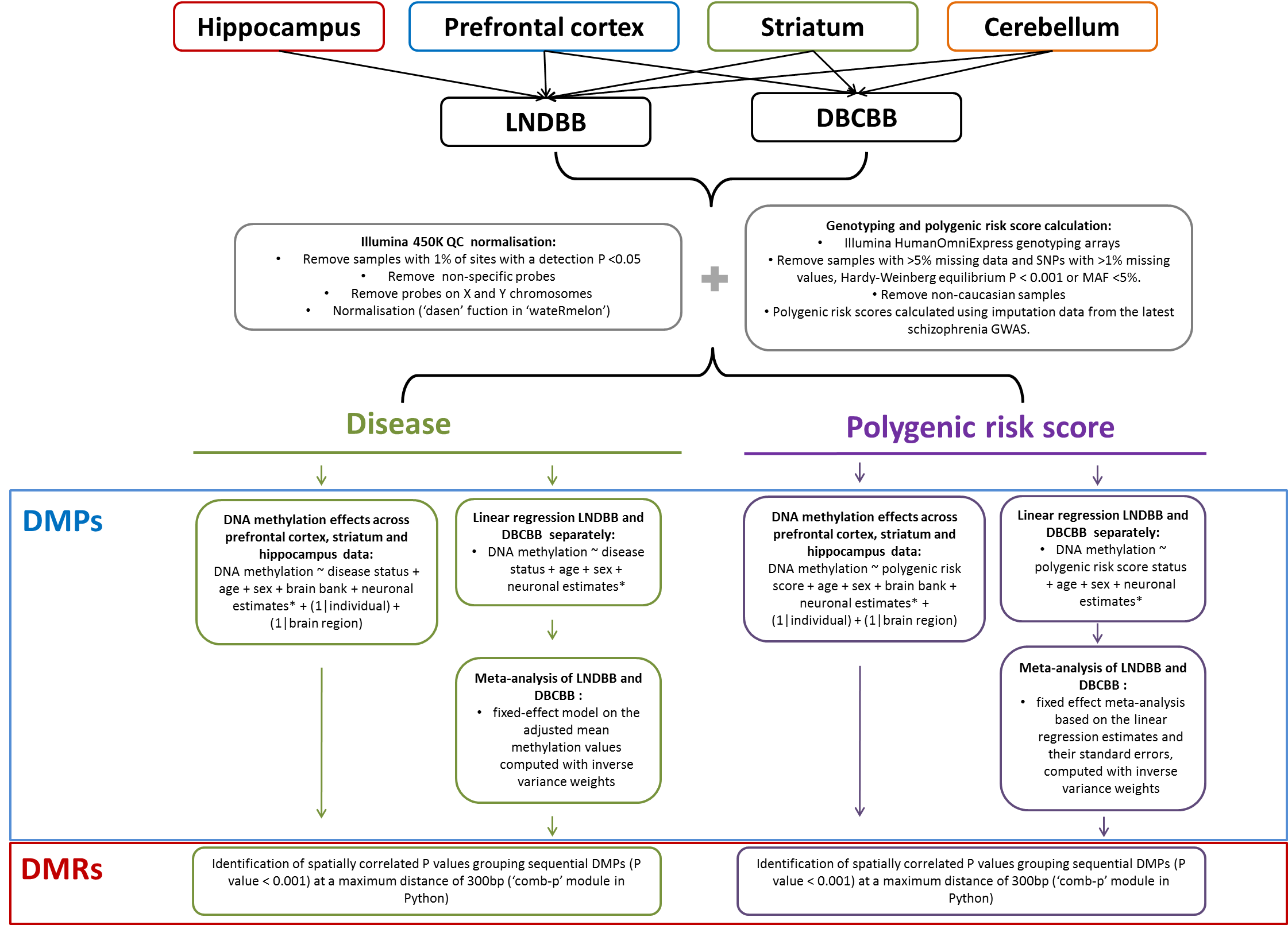
**

**Supplementary Figure3: The estimated “DNA methylation age” for each sample is strongly correlated with actual chronological age in each brain region.** Shown is the correlation between DNA methylation age (calculated using Horvath’s ‘epigenetic clock’ [^1^](#_ENREF_1)) and actual age for samples from both cohorts for A) prefrontal cortex, B) striatum, C) hippocampus, and D) cerebellum.

**Supplementary Figure 4: Heatmap showing the fifty top-ranked schizophrenia-associated differently methylated positions in the prefrontal cortex (PFC).** Shown for each probe is the DNA methylation difference between cases and controls, with the corresponding difference at the same probe for the three other brain regions (striatum (STR), hippocampus (HC) and cerebellum (CER)) dissected from the same individuals. Probes are ordered by *P-*value for hypomethylated (blue, top) and hypermethylated (red, bottom) loci within the PFC.

**Supplementary Figure 5: Heatmap showing the fifty top-ranked schizophrenia-associated differently methylated positions in the striatum (STR).** Shown for each probe is the DNA methylation difference between cases and controls, with the corresponding difference at the same probe for the three other brain regions (prefrontal cortex (PFC), hippocampus (HC) and cerebellum (CER)) dissected from the same individuals. Probes are ordered by *P-*value for hypomethylated (blue, top) and hypermethylated (red, bottom) loci within the STR.

**Supplementary Figure 6: Heatmap showing the fifty top-ranked schizophrenia-associated differently methylated positions in the hippocampus (HC).** Shown for each probe is the DNA methylation difference between cases and controls, with the corresponding difference at the same probe for the three other brain regions (prefrontal cortex (PFC), striatum (STR) and cerebellum (CER)) dissected from the same individuals. Probes are ordered by *P-*value for hypomethylated (blue, top) and hypermethylated (red, bottom) loci within the HC.

**Supplementary Figure 7: Heatmap showing the fifty top-ranked schizophrenia-associated differently methylated positions in the cerebellum (CER).** Shown for each probe is the DNA methylation difference between cases and controls, with the corresponding difference at the same probe for the three other brain regions (prefrontal cortex (PFC), striatum (STR) and hippocampus (HC)) dissected from the same individuals. Probes are ordered by *P-*value for hypomethylated (blue, top) and hypermethylated (red, bottom) loci within the CER.

**Supplementary Figure 8: Boxplots highlighting DNA methylation differences between cases and controls for the twelve significant schizophrenia-associated DMPs.** Shown are data for 12 DMPs associated with schizophrenia at a highly stringent significance threshold (*P* < 1.66E-07) derived using permutations to estimate the nominal *P*-value for 5% family-wise error. Additional information on these DMPs is given in **Table 2**. Color depicts brain region in which the schizophrenia-association was identified: prefrontal cortex = blue, striatum = green, hippocampus = red, and cerebellum = yellow.

**
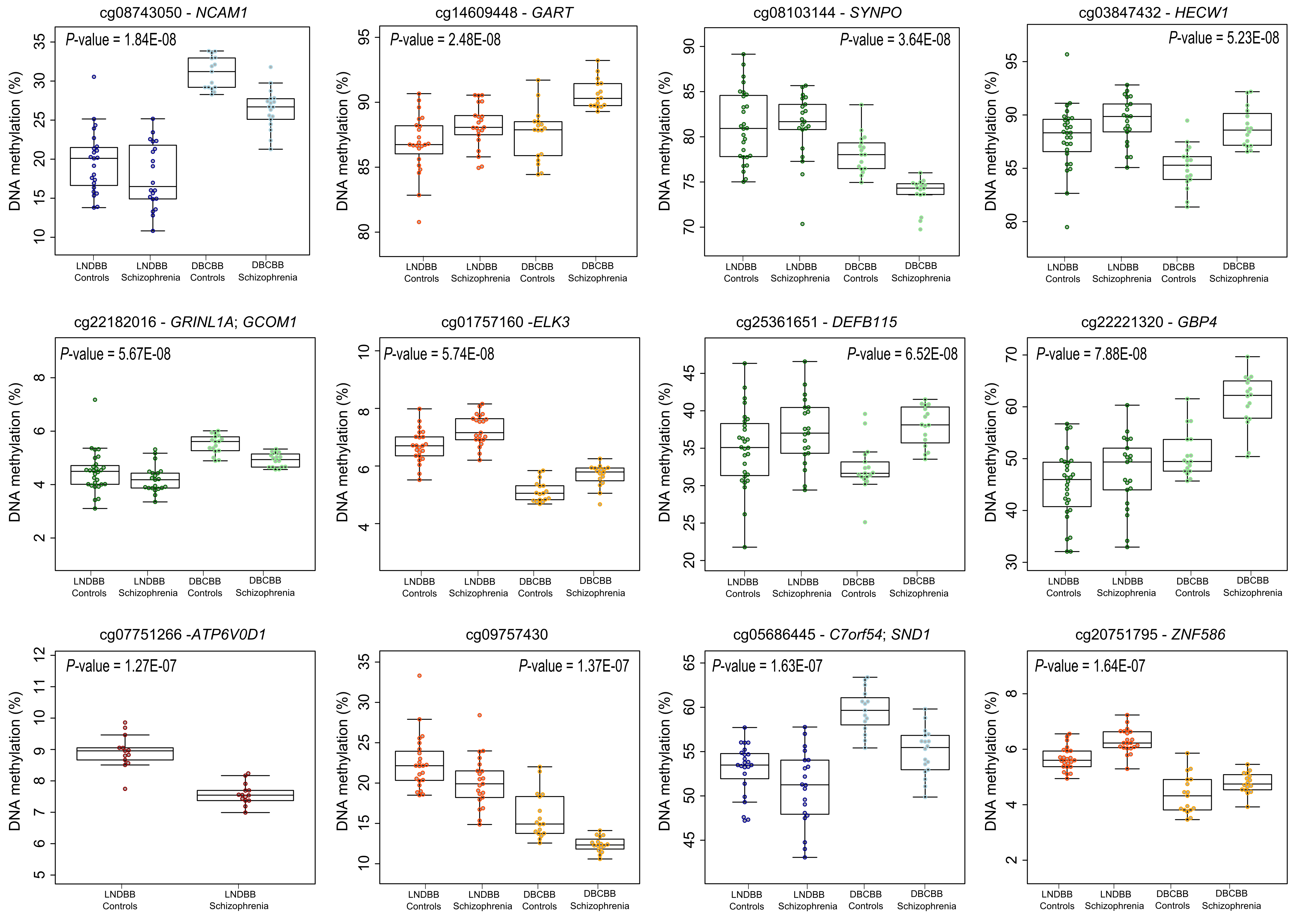
**

**Supplementary Figure 9: DNA methylation differences between schizophrenia cases and controls at top-ranked differently methylated positions (DMPs) identified in each individual brain region are significantly correlated with differences at the same sites in the other three brain regions.** Shown are the schizophrenia-associated differences observed across additional brain regions for DMPs identified in **A**) prefrontal cortex, **B**) striatum, **C**) hippocampus, and **D**) cerebellum.

**Supplementary Figure 10: Quantile-quantile plots for the case-control schizophrenia EWAS analyses.** Shown are the observed and expected distribution of *P*-values observed in the case-control analysis of A) prefrontal cortex (λ = 1.18), B) striatum (λ = 1.02), C) hippocampus (λ = 1.13), and D) cerebellum (λ = 1.23), and E) the multi-region model (λ = 1.43). Blue shading indicates 95% confidence intervals generated using permutations for each of the individual brain region analyses.

**
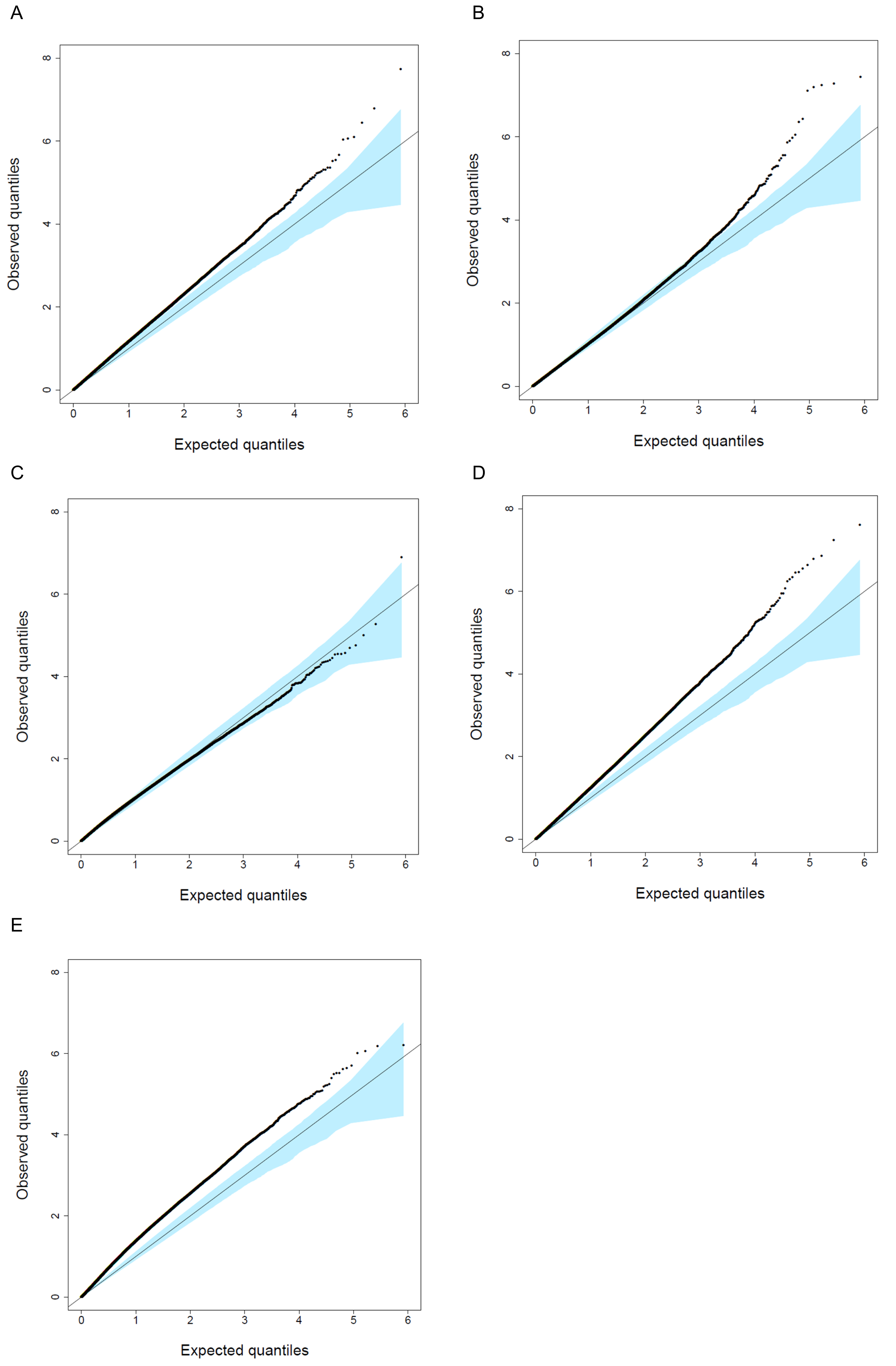
**

**Supplementary Figure 11: Schizophrenia-associated DNA methylation differences are robust to the addition of principal components (PC) capturing variation in DNA methylation data in the prefrontal cortex (PFC).** Shown is the correlation of DNA methylation differences for the fifty top-ranked PFC schizophrenia-associated DMPs (x-axis) with differences at the same positions in an EWAS iteratively adding 1 to 10 PCAs as independent co-variables (y-axis).

**
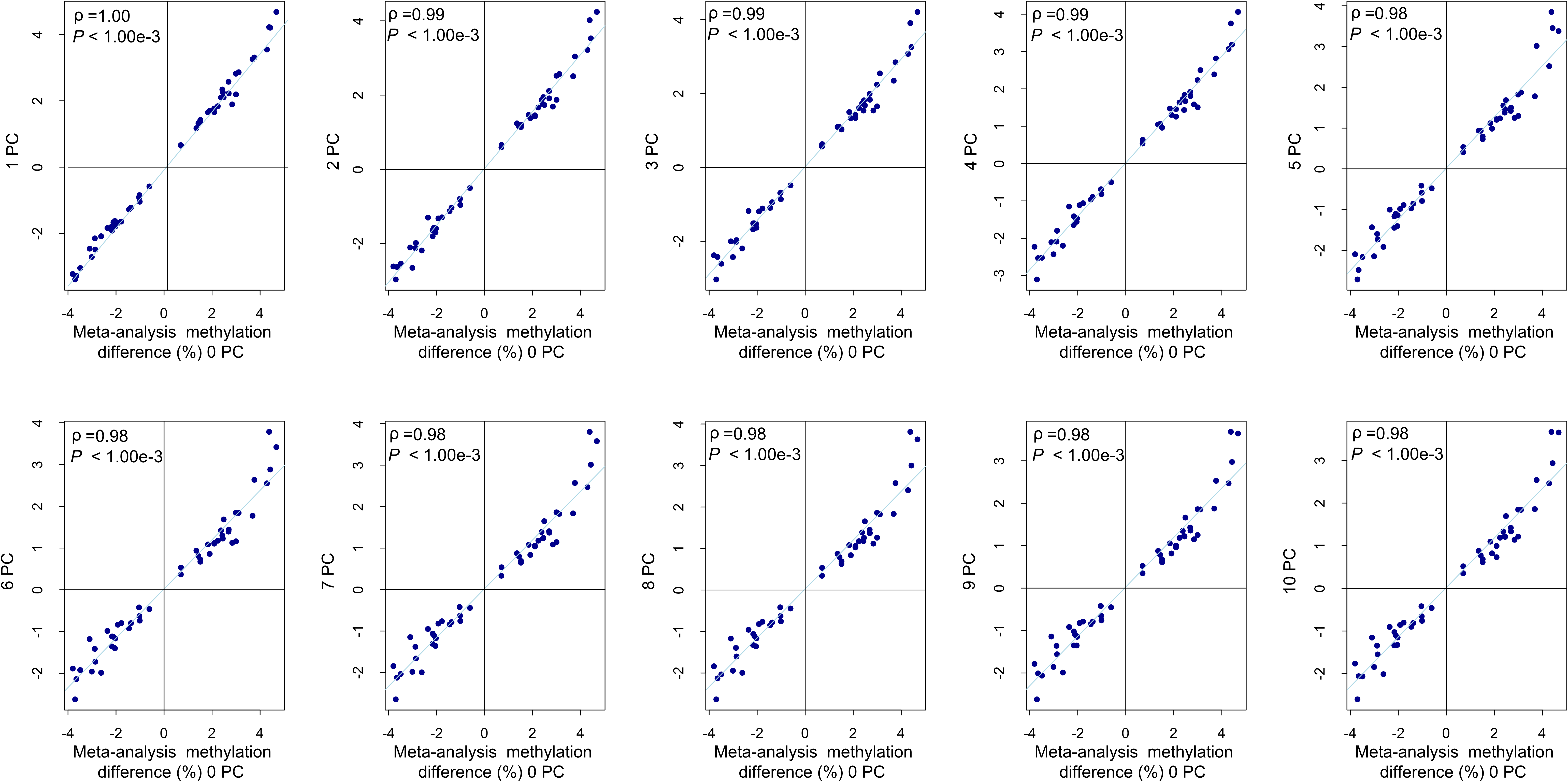
**

**Supplementary Figure 12: Schizophrenia-associated DNA methylation differences are robust to the addition of principal components (PC) capturing variation in DNA methylation data in the striatum (STR).** Shown is the correlation of DNA methylation differences for the fifty top-ranked STR schizophrenia-associated DMPs (x-axis) with differences at the same positions in an EWAS iteratively adding 1 to 10 PCAs as independent co-variables (y-axis).

**
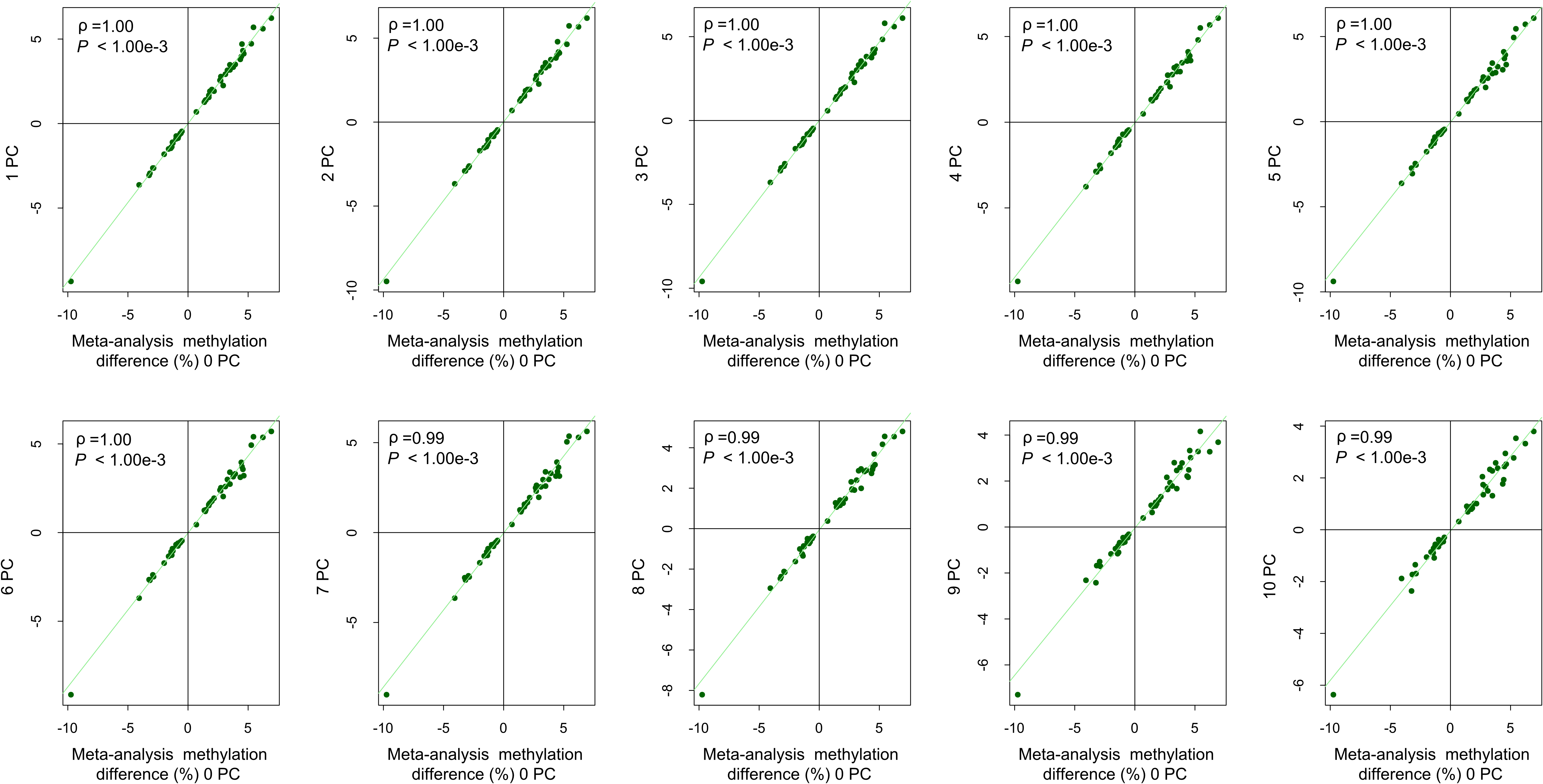
**

**Supplementary Figure 13: Schizophrenia-associated DNA methylation differences are robust to the addition of principal components (PC) capturing variation in DNA methylation data in the hippocampus (HC).** Shown is the correlation of DNA methylation differences for the fifty top-ranked HC schizophrenia-associated DMPs (x-axis) with differences at the same positions in an EWAS iteratively adding 1 to 10 PCAs as independent co-variables (y-axis).

**
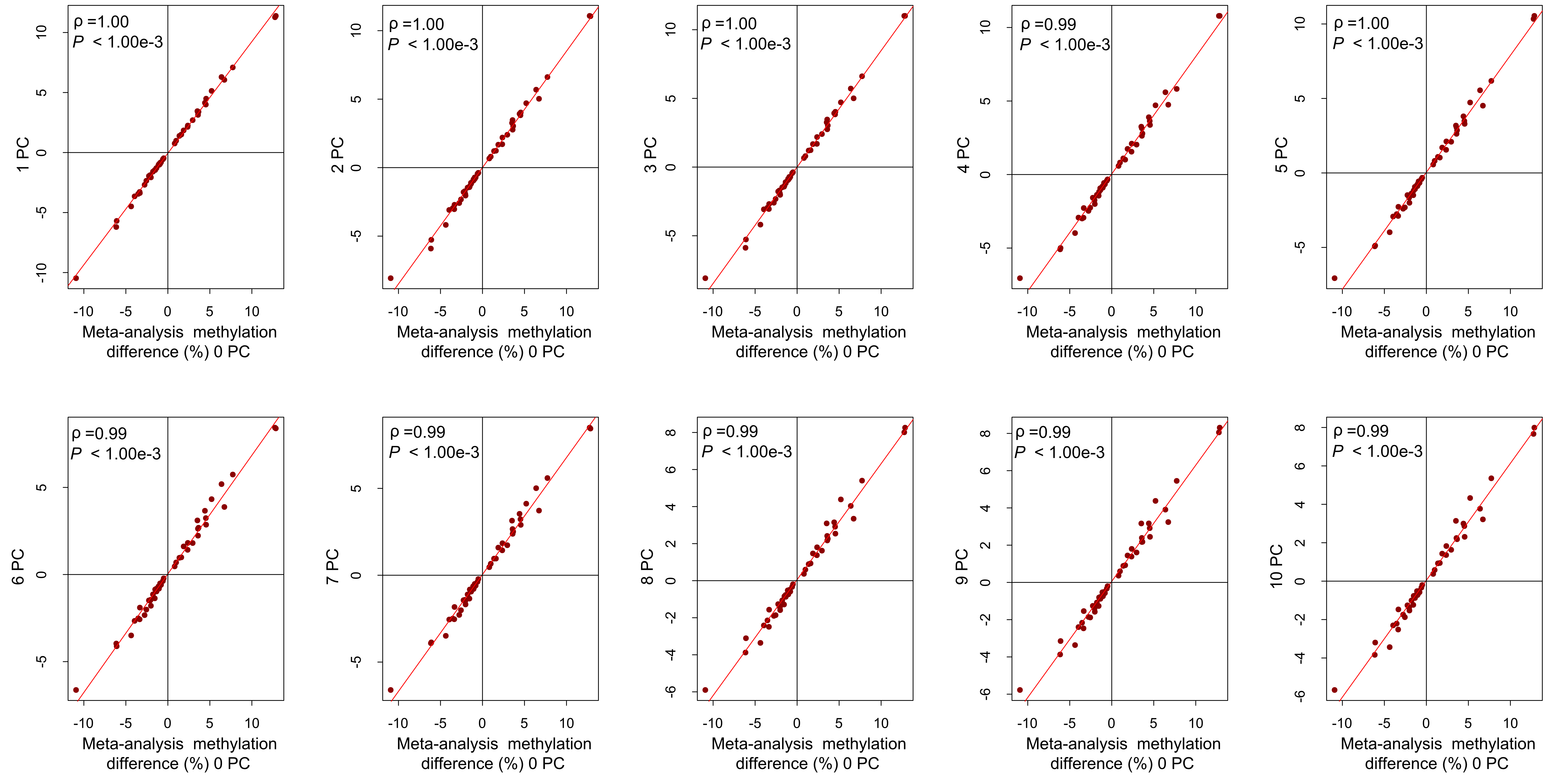
**

**Supplementary Figure 14: Schizophrenia-associated DNA methylation differences are robust to the addition of principal components (PCA) capturing variation in DNA methylation data in the cerebellum (CER).** Shown is the correlation of DNA methylation differences for the fifty top-ranked CER schizophrenia-associated DMPs (x-axis) with differences at the same positions in an EWAS iteratively adding 1 to 10 PCAs as independent co-variables (y-axis).

**
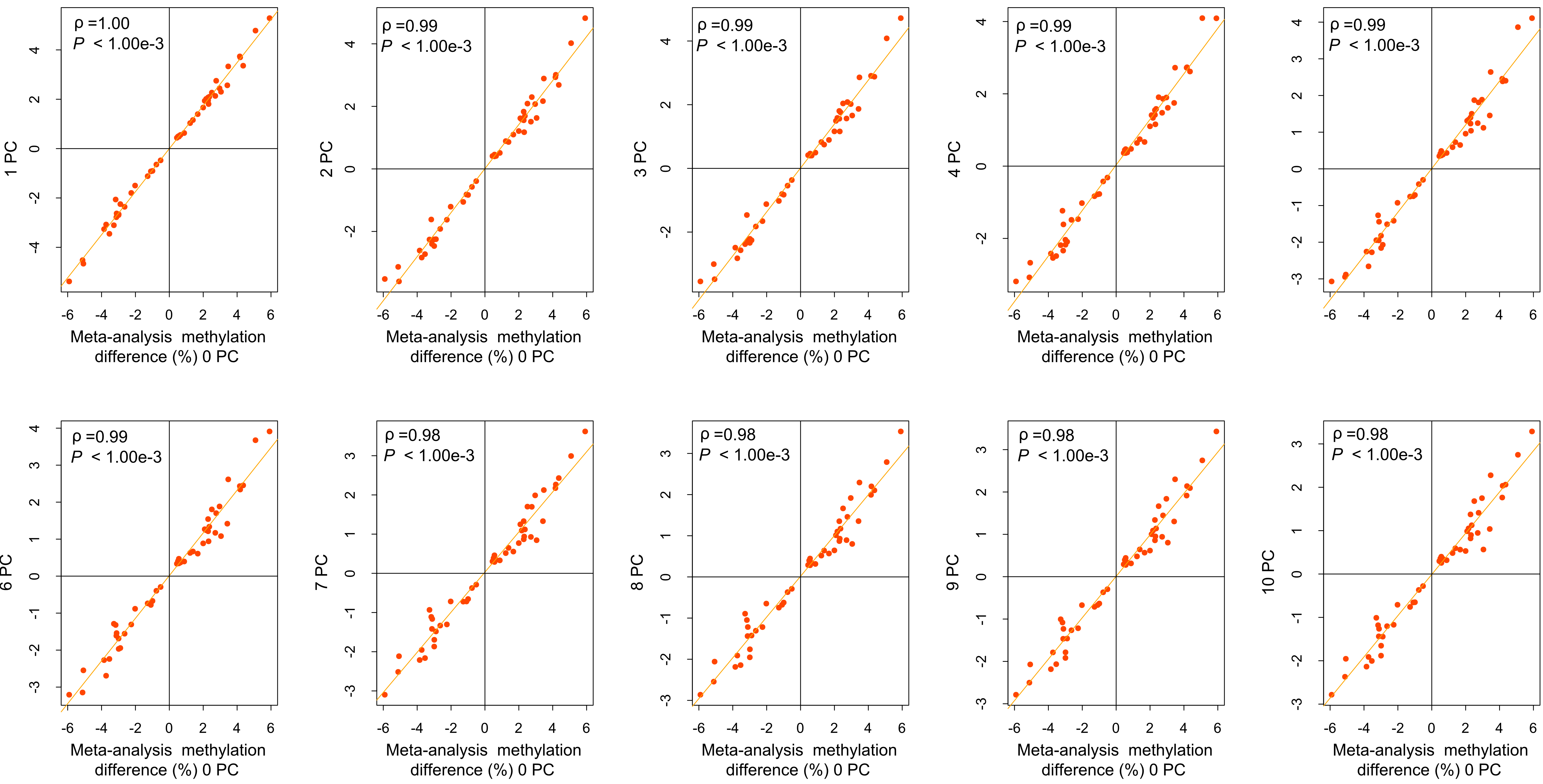
**

**Supplementary Figure 15: Validation of a highly-significant schizophrenia-associated hypomethylated region within the *RPH3AL* gene on chromosome 17 in the prefrontal cortex (PFC).** We validated the chr17:154410-154672 schizophrenia-associated region identified using the Illumina 450K array (**A** and **B**) in the PFC samples. We used bisulfite-PCR-pyrosequencing to validate schizophrenia-associated hypomethylation at cg11940040 and two adjacent CpG sites not present in the array (**C**).

**
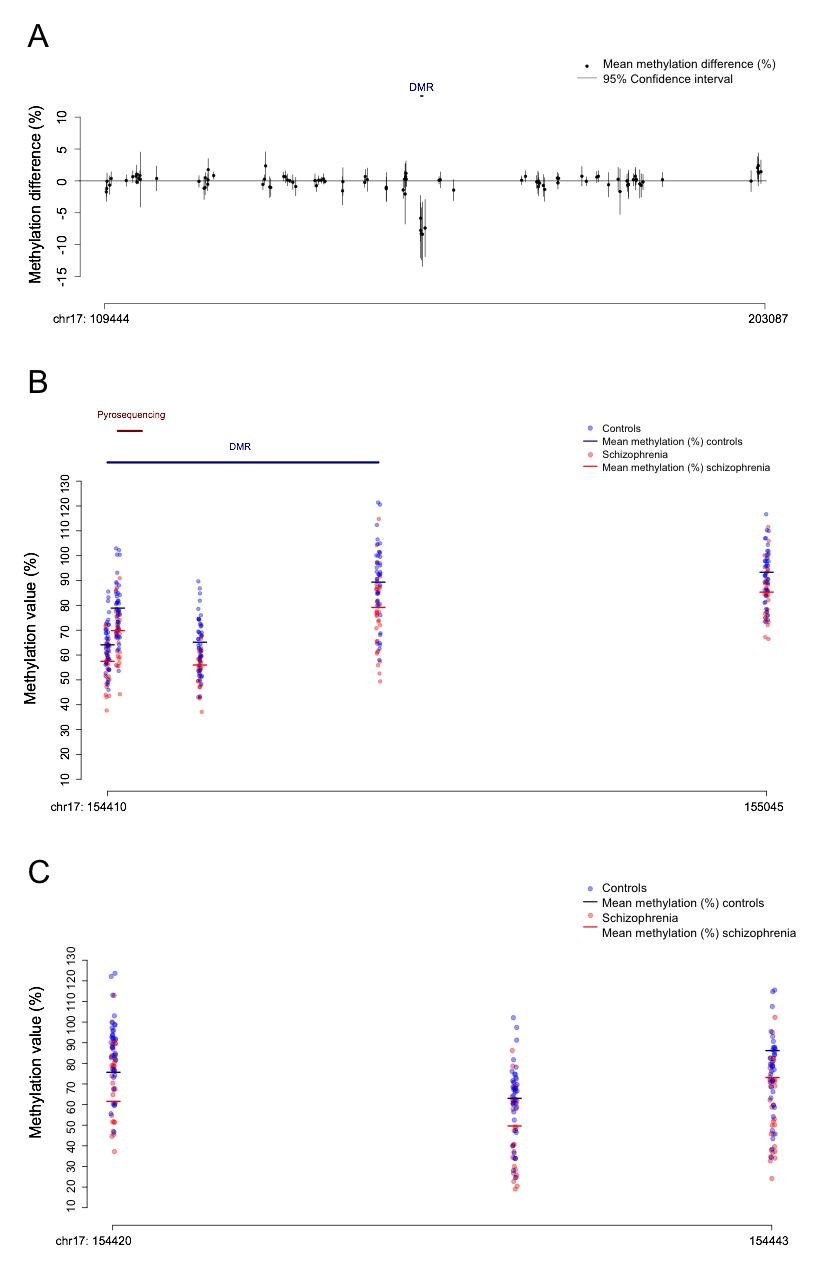
**

**Supplementary Figure 16: Validation of a highly-significant schizophrenia-associated hypomethylated region within the *RPH3AL* gene on chromosome 17 in the striatum (STR).** We validated the chr17:154410-154672 schizophrenia-associated region identified using the Illumina 450K array (**A** and **B**) in the STR samples. We used bisulfite-PCR-pyrosequencing to validate schizophrenia-associated hypomethylation at cg11940040 and two adjacent CpG sites not present in the array (**C**).

**Supplementary Figure 17: Hierarchical-clustering of the thousand most variable probes across all samples.** There are no systematic differences between samples from the two brain-banks. Cerebellum samples are clearly distinct from the other three brain regions (prefrontal cortex, striatum and hippocampus).

**Supplementary Figure 18: Heatmap showing the fifty top-ranked schizophrenia-associated differently methylated positions identified using a multi-region model incorporating prefrontal cortex (PFC), striatum (STR) and hippocampus (HC).** Shown for each probe is the DNA methylation difference between cases and controls, with the corresponding difference at the same probe for the three individual brain regions. Probes are ordered by *P-*value for hypomethylated (blue, top) and hypermethylated (red, bottom) loci from the multi-region model.

**Supplementary Figure 19: Heatmap showing differently methylated regions (DMRs) associated with schizophrenia identified using a multi-region model incorporating prefrontal cortex (PFC), striatum (STR) and hippocampus (HC).** Shown are schizophrenia-associated DNA methylation differences in each of the significant DMRs (Šidák-corrected P < 0.05, number of probes ≥ 2), with disease-associated differences in the corresponding probes shown for the three individual brain regions.

**Supplementary Figure 20: Quantile-quantile plots for schizophrenia polygenic risk score EWAS analyses.** Shown are the observed and expected quantiles (*P*-values) observed in the PRS analyses of **A**) prefrontal cortex (λ = 0.96), **B**) striatum (λ = 1.10), **C**) hippocampus (λ = 1.17), and **D**) cerebellum (λ = 1.26), and **E**) the multi-region model (λ = 1.33). Blue shading indicates 95% confidence intervals generated using permutations for each of the individual brain region analyses.

**
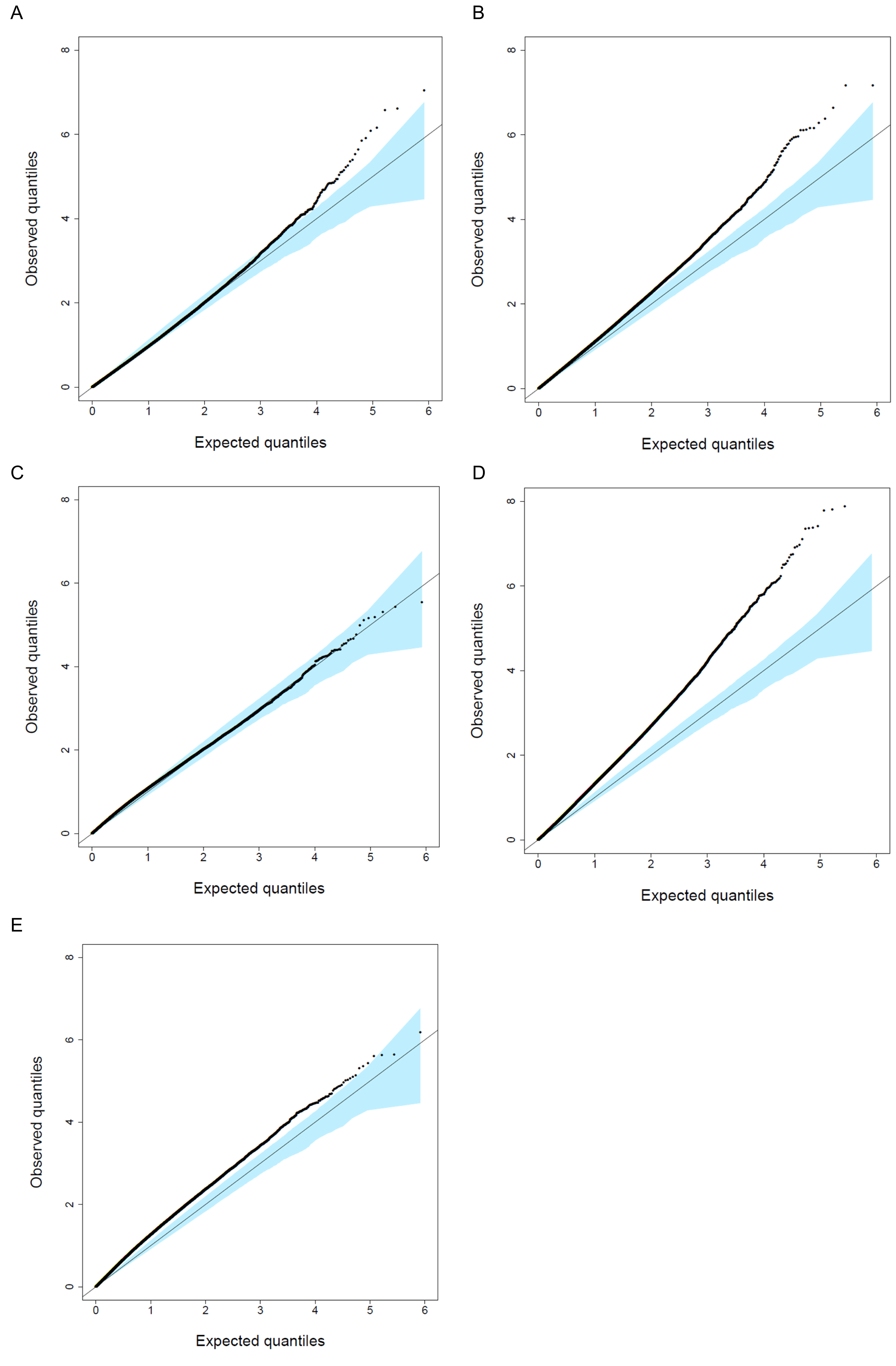
**

**Supplementary Figure 21: Heatmap showing the fifty top-ranked differently methylated positions associated with schizophrenia polygenic risk score (PRS) in the prefrontal cortex (PFC).** Shown for each probe is the DNA methylation effect size associated with PRS, with the corresponding effect at the same probe for the three other brain regions (striatum (STR), hippocampus (HC) and cerebellum (CER)) dissected from the same individuals. Probes are ordered by *P-*value for hypomethylated (blue, top) and hypermethylated (red, bottom) loci within the PFC.

**Supplementary Figure 22: Heatmap showing the fifty top-ranked differently methylated positions associated with schizophrenia polygenic risk score (PRS) in the striatum (STR).** Shown for each probe is the DNA methylation effect size associated with PRS, with the corresponding effect at the same probe for the three other brain regions (prefrontal cortex (PFC), hippocampus (HC) and cerebellum (HC)) dissected from the same individuals. Probes are ordered by *P-*value for hypomethylated (blue, top) and hypermethylated (red, bottom) loci within the STR.

**Supplementary Figure 23: Heatmap showing the fifty top-ranked differently methylated positions associated with schizophrenia polygenic risk score (PRS) in the hippocampus (HC).** Shown for each probe is the DNA methylation effect size associated with PRS, with the corresponding effect at the same probe for the three other brain regions (prefrontal cortex (PFC), striatum (STR) and cerebellum (CER)) dissected from the same individuals. Probes are ordered by *P-*value for hypomethylated (blue, top) and hypermethylated (red, bottom) loci within the HC.

**Supplementary Figure 24: Heatmap showing the fifty top-ranked differently methylated positions associated with schizophrenia polygenic risk score in the cerebellum (CER).** Shown for each probe is the DNA methylation effect size associated with (PRS), with the corresponding effect at the same probe for the three other brain regions (prefrontal cortex (PFC), striatum (STR) and hippocampus (HC)) dissected from the same individuals. Probes are ordered by *P-*value for hypomethylated (blue, top) and hypermethylated (red, bottom) loci within the CER.

**Supplementary Figure 25: DNA methylation differences associated with schizophrenia polygenic risk score (PRS) at top-ranked differently methylated positions (DMPs) identified in each individual brain region are significantly correlated with differences at the same sites in the other three brain regions.** Shown are the schizophrenia-associated differences observed across additional brain regions for PRS DMPs identified in **A**) prefrontal cortex, **B**) striatum, **C**) hippocampus, and **D**) cerebellum.

**Supplementary Figure 26: Although a non-overlapping set of top-ranked differently methylated positions (DMPs) were identified in the case-control and polygenic risk score (PRS) analyses, effect sizes are correlated across approaches.** Shown for the prefrontal cortex are **A**) PRS effect sizes for DMPs identified in the schizophrenia case-control analysis and **B**) schizophrenia-associated DNA methylation differences for DMPs identified in an analysis of schizophrenia polygenic burden.

**Supplementary Figure 27: Although a different set of top-ranked differently methylated positions (DMPs) were identified in the case-control and polygenic risk score (PRS) analyses, effect sizes are correlated across approaches.** Shown for the striatum are **A**) PRS effect sizes for DMPs identified in the schizophrenia case-control analysis and **B**) schizophrenia-associated DNA methylation differences for DMPs identified in an analysis of schizophrenia polygenic burden.

**Supplementary Figure 28: Although a different set of top-ranked differently methylated positions (DMPs) were identified in the case-control and polygenic risk score (PRS) analyses, effect sizes are correlated across approaches.** Shown for the hippocampus are **A**) PRS effect sizes for DMPs identified in the schizophrenia case-control analysis and **B**) schizophrenia-associated DNA methylation differences for DMPs identified in an analysis of schizophrenia polygenic burden.

**Supplementary Figure 29: Although a different set of top-ranked differently methylated positions (DMPs) were identified in the case-control and polygenic risk score (PRS) analyses, effect sizes are correlated across approaches.** Shown for the cerebellum are **A**) PRS effect sizes for DMPs identified in the schizophrenia case-control analysis and **B**) schizophrenia-associated DNA methylation differences for DMPs identified in an analysis of schizophrenia polygenic burden.

**Supplementary Figure 30: Heatmap showing the fifty top-ranked differently methylated positions associated with schizophrenia polygenic risk score identified using a multi-region model incorporating prefrontal cortex (PFC), striatum (STR) and hippocampus (HC).** Shown for each probe is the DNA methylation effect size, with the corresponding difference at the same probe for the three individual brain regions. Probes are ordered by *P-*value for hypomethylated (blue, top) and hypermethylated (red, bottom) loci from the multi-region model.

**Supplementary Figure 31: Heatmap showing differently methylated regions (DMRs) associated with schizophrenia polygenic risk score identified using a multi-region model incorporating prefrontal cortex (PFC), striatum (STR) and hippocampus (HC).** Shown is the DNA methylation effect size for probes in each of the significant DMRs (Šidák-corrected P < 0.05, number of probes ≥ 2), with the corresponding effect size at the same probe for the three individual brain regions.

**Supplementary Figure 32: The relationship between lowest mQTL *P*-value and polygenic risk score EWAS *P*-value for all DNA methylation probes associated (P < 1.00E-10) with genotype at a SNP incorporated in the schizophrenia PRS in A) prefrontal cortex, B) striatum and C) cerebellum.** The solid red line indicates a stringent PRS EWAS threshold of *P <* 1.66E-07. The dashed red line indicates a lenient PRS EWAS threshold of *P* < 1.00E-03.


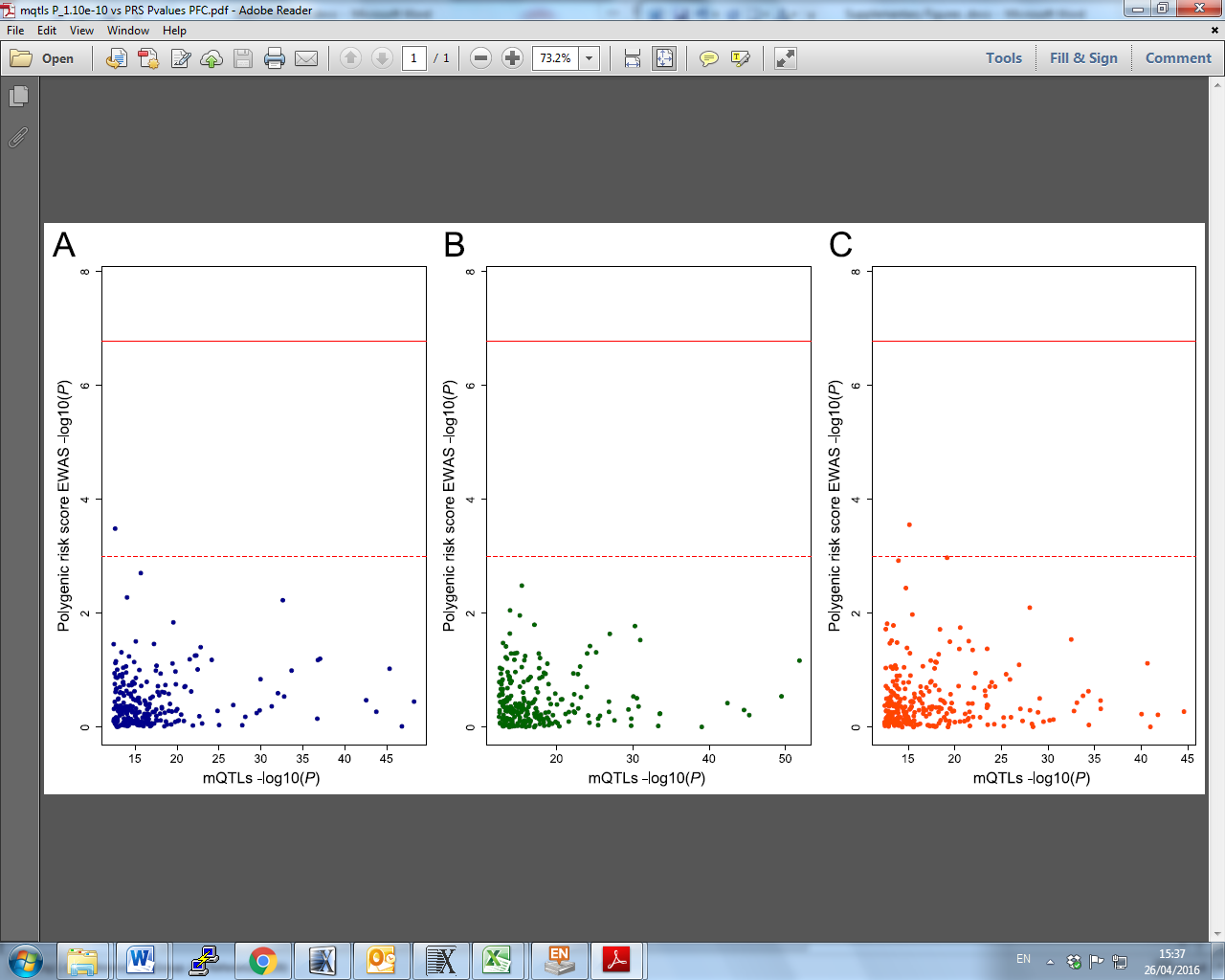


**REFERENCES**

1. Horvath S. DNA methylation age of human tissues and cell types. *Genome biology* 2013; **14**(10)**:** R115.
