# Supplementary material for: Schizophrenia-associated methylomic variation: molecular signatures of disease and polygenic risk burden across multiple brain regions
Source: Hum Mol Genet. 2016 Dec 22;26(1):210–25. doi: 10.1093/hmg/ddw373 (PMC5351932; doi:10.1093/hmg/ddw373)
Supplement: Supplementary Data [file ddw373_Supp.zip › Supplementary Tables Final.docx]

**Supplementary Tables**

Viana et al

**Schizophrenia-associated methylomic variation: molecular signatures of disease and polygenic risk burden across multiple brain regions**

**Supplementary Table 1: Overview of samples included in the polygenic risk score analysis.** LNDBB = MRC London Neurodegenerative Diseases Brain Bank, DBCBB = Douglas-Bell Canada Brain Bank.

|  |  |  | **N** | **Sex (male:female)** | **Age at death** | **Brain weight (g)** | **pH** | **Polygenic risk score** |
| --- | --- | --- | --- | --- | --- | --- | --- | --- |
| **LNDBB** | **Prefrontal cortex** | **schizophrenia** | 16 | 9:7 | 65.68 ± 14.61 | 1222.46 ± 140.74 | 6.64 ± 0.29 | -15.50 ± 0.29 |
|  |  | **controls** | 20 | 14:6 | 62.70 ± 19.76 | 1389.50 ± 186.12 | 6.46 ± 0.34 | -18.83 ± 0.34 |
|  |  | **total** | 36 | 23:13 | 64.03 ± 17.49 | 1323.70 ± 186.72 | 6.54 ± 0.33 | -17.31 ± 0.33 |
|  |  | ***P*** | - | - | 0.61 | 0.01 | 0.10 | 0.01 |
|  | **Striatum** | **schizophrenia** | 17 | 9:8 | 65.94 ± 14.19 | 1220.36 ± 135.45 | 6.61 ± 0.3 | -15.34 ± 3.81 |
|  |  | **controls** | 23 | 16:7 | 65.22 ± 19 | 1385.26 ± 190.23 | 6.44 ± 0.34 | -18.75 ± 3.20 |
|  |  | **total** | 40 | 25:15 | 65.53 ± 16.92 | 1315.30 ± 186.17 | 6.52 ± 0.33 | -17.30 ± 3.83 |
|  |  | ***P*** | - | - | 0.89 | 0.01 | 0.12 | 0.01 |
|  | **Hippocampus** | **schizophrenia** | 11 | 8:3 | 65.91 ± 13.17 | 1255.11 ± 158.25 | 6.63 ± 0.29 | -14.79 ± 2.79 |
|  |  | **controls** | 12 | 10:2 | 62.58 ± 18.43 | 1422.10 ± 181.66 | 6.43 ± 0.39 | -20.55 ± 2.28 |
|  |  | **total** | 23 | 18:5 | 64.17 ± 15.86 | 1343 ± 187 | 6.53 ± 0.35 | -17.79 ± 3.84 |
|  |  | ***P*** | - | - | 0.62 | 0.05 | 0.21 | 3.08E-05 |
|  | **Cerebellum** | **schizophrenia** | 17 | 9:8 | 65.94 ± 14.19 | 1220.36 ± 135.45 | 6.61 ± 0.3 | -15.34 ± 3.81 |
|  |  | **controls** | 19 | 13:6 | 63.11 ± 20.22 | 1386.21 ± 190.62 | 6.44 ± 0.33 | -18.69 ± 3.14 |
|  |  | **total** | 36 | 22:14 | 64.44 ± 17.44 | 1315.85 ± 186.61 | 6.52 ± 0.33 | -17.07 ± 3.83 |
|  |  | ***P*** | - | - | 0.63 | 0.01 | 0.11 | 0.01 |
| **DBCBB** | **Prefrontal cortex** | **schizophrenia** | 17 | 14:3 | 46.24 ± 16.81 | 1404.62 ± 161.9 | 6.25 ± 0.21 | -16.64 ± 2.87 |
|  |  | **controls** | 14 | 12:2 | 46.75 ± 16.72 | 1430.09 ± 183.76 | 6.06 ± 0.29 | -18.41 ± 3.11 |
|  |  | **total** | 31 | 26:5 | 46.48 ± 16.51 | 1418.20 ± 171.41 | 6.16 ± 0.27 | -17.52 ± 3.08 |
|  |  | ***P*** | - | - | 0.93 | 0.69 | 0.05 | 0.11 |
|  | **Striatum** | **schizophrenia** | 15 | 12:3 | 47.13 ± 17.32 | 1377.48 ± 157.92 | 6.23 ± 0.21 | -16.90 ± 2.78 |
|  |  | **controls** | 16 | 13:3 | 46.75 ± 16.72 | 1430.09 ± 183.76 | 6.06 ± 0.29 | -18.41 ± 3.11 |
|  |  | **total** | 31 | 25:6 | 46.94 ± 16.73 | 1407.54 ± 172.11 | 6.14 ± 0.27 | -17.68 ± 3.01 |
|  |  | ***P*** | - | - | 0.95 | 0.42 | 0.08 | 0.16 |
|  | **Cerebellum** | **schizophrenia** | 16 | 14:2 | 44.56 ± 15.84 | 1404.62 ± 161.9 | 6.25 ± 0.22 | -16.84 ± 2.86 |
|  |  | **controls** | 16 | 13:3 | 46.75 ± 16.72 | 1430.09 ± 183.76 | 6.06 ± 0.29 | -18.41 ± 3.11 |
|  |  | **total** | 32 | 27:5 | 45.66 ± 16.06 | 1418.20 ± 171.41 | 6.16 ± 0.27 | -17.65 ± 3.05 |
|  |  | ***P*** | - | - | 0.71 | 0.69 | 0.04 | 0.15 |

**Supplementary Table 2: Top ranked schizophrenia-associated differently methylated probes (DMPs) identified in the prefrontal cortex (PFC) meta-analysis.** Listed for each PFC DMP (grey) are corresponding results from the striatum (STR; *P* < 0.05 in green), hippocampus (HC; *P* < 0.05 in red) and cerebellum (CER; *P* < 0.05 in orange) meta-analyses (PFC, STR and CER) or linear regression (HC). Also shown is the association with schizophrenia polygenic burden in PFC (*P* < 0.05 in blue). Illumina and Genomic Regions Enrichment of Annotation Tool (GREAT) annotation[^1^](#_ENREF_1) is listed for each DMP.

| \| **Probe ID** \| **Genomic position (hg19)** \| **Illumina gene annotation** \| **Gene region** \| **GREAT annotation** [**^1^**](#_ENREF_1) \| **Methylation difference PFC (%)** \| ***P*  PFC** \| **Methylation difference STR (%)** \| ***P* STR** \| **Methylation difference HC (%)** \| ***P* HC** \| **Methylation difference CER (%)** \| ***P* CER** \| **Polygenic risk score PFC methylation difference (%)** \| **Polygenic risk score PFC *P*** \| \| --- \| --- \| --- \| --- \| --- \| --- \| --- \| --- \| --- \| --- \| --- \| --- \| --- \| --- \| --- \| \| cg08743050 \| chr11:113113936 \| NCAM1 \| Body \| TTC12; NCAM1 \| -3.80 \| 1.84E-08 \| -0.59 \| 0.56 \| -2.53 \| 0.18 \| -0.48 \| 0.29 \| -0.10 \| 0.55 \| \| cg05686445 \| chr7:127636396 \| C7orf54; SND1 \| TSS1500; Body \| LRRC4; SND1 \| -3.65 \| 1.63E-07 \| -0.67 \| 0.51 \| 3.17 \| 0.26 \| -0.75 \| 0.50 \| -0.49 \| 5.21E-04 \| \| cg26778001 \| chr19:55142181 \| LILRB1 \| 5'UTR \| LILRB1 \| 2.48 \| 3.64E-07 \| 1.83 \| 0.01 \| 1.09 \| 0.34 \| 3.75 \| 4.02E-04 \| 0.20 \| 0.07 \| \| cg26173173 \| chr8:144642813 \| GSDMD \| Body \| GSDMD; C8orf73 \| 3.11 \| 8.06E-07 \| 1.39 \| 0.08 \| 1.73 \| 0.30 \| 1.21 \| 0.18 \| 0.12 \| 0.40 \| \| cg03325693 \| chr11:34196316 \| ABTB2 \| Body \| NAT10; ABTB2 \| -2.36 \| 8.73E-07 \| -1.28 \| 0.05 \| 0.28 \| 0.88 \| -0.08 \| 0.88 \| -0.28 \| 0.01 \| \| cg16782339 \| chr2:109847146 \| SH3RF3 \| Body \| SH3RF3; SEPT10 \| 1.83 \| 9.12E-07 \| 0.11 \| 0.78 \| -1.86 \| 0.05 \| 0.19 \| 0.79 \| 0.11 \| 0.13 \| \| cg10071493 \| chr16:1179514 \| - \| - \| C1QTNF8; CACNA1H \| 3.69 \| 2.13E-06 \| -0.42 \| 0.54 \| 0.64 \| 0.72 \| 0.03 \| 0.97 \| 0.09 \| 0.59 \| \| cg18812956 \| chr3:19987665 \| RAB5A \| TSS1500 \| RAB5A \| 4.68 \| 2.90E-06 \| 1.70 \| 0.22 \| 1.49 \| 0.58 \| 1.83 \| 0.28 \| 0.24 \| 0.26 \| \| cg23303408 \| chr5:145718597 \| POU4F3 \| 5'UTR; 1stExon \| POU4F3 \| -1.93 \| 3.04E-06 \| -0.56 \| 0.18 \| 0.15 \| 0.88 \| -0.09 \| 0.79 \| -0.06 \| 0.53 \| \| cg24281764 \| chr12:132330505 \| MMP17 \| Body \| ULK1; MMP17 \| 0.71 \| 4.38E-06 \| -8.18E-02 \| 0.67 \| -0.10 \| 0.76 \| -0.09 \| 0.51 \| -0.02 \| 0.59 \| \| cg10654165 \| chr8:105479705 \| DPYS \| TSS1500 \| DPYS \| 2.24 \| 4.39E-06 \| -1.61 \| 0.02 \| -0.29 \| 0.82 \| 0.24 \| 0.82 \| 0.12 \| 0.22 \| \| cg16204289 \| chr2:75796759 \| FAM176A \| 5'UTR; 1stExon \| MRPL19; FAM176A \| 1.51 \| 4.86E-06 \| -3.14E-02 \| 0.95 \| 0.19 \| 0.75 \| 0.44 \| 0.37 \| 0.03 \| 0.74 \| \| cg17901382 \| chr17:73514798 \| TSEN54 \| Body \| CASKIN2; TSEN54 \| 2.69 \| 4.98E-06 \| 9.09E-04 \| 1.00 \| 0.38 \| 0.76 \| -1.99 \| 0.25 \| 0.19 \| 0.10 \| \| cg10932125 \| chr8:28929689 \| KIF13B \| Body \| HMBOX1; KIF13B \| 2.37 \| 5.38E-06 \| -0.63 \| 0.31 \| 0.87 \| 0.36 \| 0.97 \| 0.13 \| 0.11 \| 0.37 \| \| cg20098710 \| chr19:19640075 \| YJEFN3 \| Body \| YJEFN3 \| -2.88 \| 5.61E-06 \| -0.75 \| 0.46 \| 0.57 \| 0.77 \| -0.52 \| 0.51 \| -0.25 \| 0.04 \| \| cg20383948 \| chr21:46898137 \| COL18A1 \| Body \| COL18A1; SLC19A1 \| -3.70 \| 5.89E-06 \| 0.60 \| 0.55 \| -3.29 \| 0.09 \| 1.21 \| 0.02 \| -0.26 \| 0.09 \| \| cg16350225 \| chr1:167666363 \| RCSD1 \| Body \| CREG1; MPZL1 \| 4.28 \| 6.08E-06 \| -0.47 \| 0.64 \| 2.08 \| 0.28 \| 2.91 \| 0.01 \| 0.07 \| 0.71 \| \| cg19028706 \| chr3:42158765 \| TRAK1 \| Body \| TRAK1; CCK \| -3.49 \| 6.71E-06 \| -0.18 \| 0.89 \| 2.15 \| 0.32 \| -0.35 \| 0.61 \| 0.15 \| 0.35 \| \| cg07405426 \| chr16:30825646 \| - \| - \| ZNF629; BCL7C \| 0.70 \| 7.44E-06 \| -0.45 \| 0.06 \| 0.32 \| 0.41 \| -0.47 \| 0.02 \| 0.02 \| 0.52 \| \| cg20044211 \| chr6:32185995 \| NOTCH4 \| Body \| GPSM3; NOTCH4 \| 2.69 \| 7.54E-06 \| 1.30 \| 0.04 \| 2.69 \| 0.17 \| 1.68 \| 0.03 \| 0.14 \| 0.18 \| \| cg10072351 \| chr17:45000308 \| GOSR2 \| TSS200 \| GOSR2 \| -0.61 \| 8.09E-06 \| 0.06 \| 0.76 \| 0.30 \| 0.76 \| -0.56 \| 0.06 \| -0.02 \| 0.64 \| \| cg26088561 \| chr6:30619080 \| C6orf136 \| Body \| C6orf136; DHX16 \| -2.62 \| 8.26E-06 \| 0.84 \| 0.20 \| 2.61 \| 0.02 \| -0.44 \| 0.65 \| -0.04 \| 0.72 \| \| cg04314225 \| chr21:47844134 \| PCNT \| Body \| DIP2A; PCNT \| 2.09 \| 9.18E-06 \| 0.34 \| 0.64 \| -1.09 \| 0.26 \| 1.03 \| 0.23 \| 0.09 \| 0.39 \| \| cg06099244 \| chr3:112739834 \| C3orf17 \| TSS1500 \| C3orf17 \| 1.90 \| 9.47E-06 \| -0.16 \| 0.78 \| 1.10 \| 0.43 \| 0.25 \| 0.74 \| 0.20 \| 0.02 \| \| cg06777813 \| chr15:101085111 \| LASS3 \| TSS200 \| CERS3 \| 4.38 \| 1.02E-05 \| 2.07 \| 0.06 \| 1.15 \| 0.52 \| 3.22 \| 1.91E-03 \| -0.38 \| 0.07 \| \| cg02272814 \| chr6:46655782 \| TDRD6 \| 1stExon; 5'UTR \| TDRD6 \| 2.99 \| 1.06E-05 \| 0.27 \| 0.66 \| 2.78 \| 0.04 \| 1.19 \| 0.09 \| 0.19 \| 0.17 \| \| cg27059530 \| chr14:67707359 \| MPP5 \| TSS1500 \| MPP5 \| -1.02 \| 1.08E-05 \| -0.55 \| 0.04 \| -1.02 \| 0.08 \| 0.14 \| 0.52 \| -0.07 \| 0.17 \| \| cg07243041 \| chr16:1135931 \| - \| - \| C1QTNF8; SSTR5 \| 2.43 \| 1.11E-05 \| 0.55 \| 0.16 \| 1.14 \| 0.25 \| 0.64 \| 0.42 \| -0.06 \| 0.61 \| \| cg14125292 \| chr16:34407918 \| - \| - \| BC068290 \| 2.45 \| 1.17E-05 \| 0.74 \| 0.25 \| 2.60 \| 0.07 \| -0.33 \| 0.73 \| 0.08 \| 0.48 \| \| cg18705408 \| chr2:20212524 \| MATN3 \| TSS200 \| MATN3 \| -1.01 \| 1.26E-05 \| -0.25 \| 0.19 \| 0.01 \| 0.97 \| -0.14 \| 0.43 \| -0.07 \| 0.22 \| \| cg09741917 \| chr2:98702260 \| VWA3B \| TSS1500 \| CNGA3; TMEM131 \| 1.51 \| 1.40E-05 \| 0.36 \| 0.42 \| 0.35 \| 0.66 \| 0.43 \| 0.53 \| 0.03 \| 0.67 \| \| cg25529303 \| chr6:151186601 \| MTHFD1L \| TSS200 \| MTHFD1L \| -2.03 \| 1.46E-05 \| -0.70 \| 0.34 \| 1.10 \| 0.53 \| -1.15 \| 0.06 \| -0.21 \| 0.02 \| \| cg08633665 \| chr8:128972829 \| MIR1205; PVT1 \| TSS200; Body \| MYC \| 2.99 \| 1.52E-05 \| -0.14 \| 0.84 \| 0.86 \| 0.54 \| 1.75 \| 0.01 \| 0.05 \| 0.74 \| \| cg16679302 \| chr16:85622276 \| - \| - \| KIAA0182; KIAA0513 \| -2.86 \| 1.52E-05 \| 0.53 \| 0.61 \| 1.87 \| 0.30 \| -1.55 \| 0.37 \| -0.05 \| 0.76 \| \| cg25049698 \| chr6:50692605 \| TFAP2D \| Body \| TFAP2B; TFAP2D \| -2.16 \| 1.54E-05 \| 0.20 \| 0.72 \| 1.38 \| 0.23 \| 0.27 \| 0.59 \| -0.20 \| 0.05 \| \| cg02213139 \| chr1:85527754 \| WDR63 \| TSS1500 \| MCOLN3; SYDE2 \| -1.46 \| 1.54E-05 \| 0.39 \| 0.30 \| -0.74 \| 0.44 \| -0.08 \| 0.86 \| 0.10 \| 0.24 \| \| cg27645498 \| chr12:125145553 \| - \| - \| NCOR2; SCARB1 \| -3.10 \| 1.54E-05 \| -0.35 \| 0.73 \| -2.16 \| 0.26 \| -0.96 \| 0.18 \| -0.15 \| 0.34 \| \| cg02370100 \| chr21:43655256 \| ABCG1 \| Body \| ABCG1; TFF3 \| -2.04 \| 1.56E-05 \| -0.75 \| 0.25 \| -0.75 \| 0.55 \| 0.04 \| 0.93 \| -0.17 \| 0.09 \| \| cg24044052 \| chr2:231191662 \| SP140L \| TSS1500 \| SP140L \| 2.84 \| 1.75E-05 \| 0.34 \| 0.63 \| 1.30 \| 0.39 \| 0.44 \| 0.44 \| 0.06 \| 0.68 \| \| ch.3.183336F \| chr3:9516816 \| SETD5 \| Body \| LHFPL4; THUMPD3 \| -1.78 \| 2.00E-05 \| 0.15 \| 0.69 \| 0.63 \| 0.45 \| 0.27 \| 0.58 \| 0.03 \| 0.79 \| \| cg26963844 \| chr17:47929214 \| - \| - \| TAC4 \| -1.38 \| 2.09E-05 \| -0.29 \| 0.53 \| -1.47 \| 0.07 \| -0.05 \| 0.86 \| 0.09 \| 0.18 \| \| cg26548293 \| chr1:153606178 \| C1orf77; S100A13 \| TSS1500; 5'UTR \| CHTOP \| 3.76 \| 2.11E-05 \| 1.53 \| 0.19 \| -0.37 \| 0.90 \| 2.81 \| 0.02 \| 0.15 \| 0.36 \| \| cg15081722 \| chr10:1517340 \| ADARB2 \| Body \| IDI1; ADARB2 \| 4.42 \| 2.12E-05 \| 1.73 \| 0.14 \| -0.43 \| 0.85 \| 2.52 \| 0.11 \| 0.10 \| 0.68 \| \| cg06477164 \| chr2:37375682 \| EIF2AK2 \| TSS1500; 5'UTR \| EIF2AK2; CCDC75 \| 2.09 \| 2.34E-05 \| 0.09 \| 0.84 \| -0.03 \| 0.98 \| 1.04 \| 0.11 \| 0.14 \| 0.24 \| \| cg22488717 \| chr2:26785946 \| C2orf70 \| Body \| OTOF \| -2.11 \| 2.39E-05 \| -0.57 \| 0.30 \| -1.40 \| 0.31 \| 0.05 \| 0.95 \| -0.03 \| 0.80 \| \| cg02565255 \| chr5:177503040 \| - \| - \| PROP1; N4BP3 \| -1.04 \| 2.54E-05 \| 0.19 \| 0.57 \| -0.58 \| 0.34 \| -0.06 \| 0.84 \| -0.09 \| 0.05 \| \| cg11321921 \| chr18:77235850 \| NFATC1 \| Body \| CTDP1; NFATC1 \| 1.43 \| 2.54E-05 \| 0.37 \| 0.36 \| 0.22 \| 0.78 \| 0.07 \| 0.88 \| 0.09 \| 0.19 \| \| cg09789590 \| chr19:46800479 \| HIF3A \| Body; TSS1500 \| HIF3A \| -2.17 \| 2.60E-05 \| -0.52 \| 0.41 \| -1.38 \| 0.17 \| 0.74 \| 0.01 \| 0.00 \| 0.98 \| \| cg03944444 \| chr1:16785803 \| NECAP2 \| 3'UTR \| NECAP2; NBPF1 \| -3.01 \| 2.62E-05 \| 1.19 \| 0.13 \| -0.93 \| 0.58 \| 0.60 \| 0.35 \| -0.31 \| 0.02 \| \| cg26819783 \| chr2:240653447 \| - \| - \| HDAC4; NDUFA10 \| 1.35 \| 3.01E-05 \| -0.51 \| 0.24 \| 1.23 \| 0.06 \| 0.74 \| 0.08 \| 0.08 \| 0.25 \| |  |  |  |  |  |  |  |  |  |  |  |  |  |  |  |  |
| --- | --- | --- | --- | --- | --- | --- | --- | --- | --- | --- | --- | --- | --- | --- | --- | --- | --- | --- | --- | --- | --- | --- | --- | --- | --- | --- | --- | --- | --- | --- | --- | --- | --- | --- | --- | --- | --- | --- | --- | --- | --- | --- | --- | --- | --- | --- | --- | --- | --- | --- | --- | --- | --- | --- | --- | --- | --- | --- | --- | --- | --- | --- | --- | --- | --- | --- | --- | --- | --- | --- | --- | --- | --- | --- | --- | --- | --- | --- | --- | --- | --- | --- | --- | --- | --- | --- | --- | --- | --- | --- | --- | --- | --- | --- | --- | --- | --- | --- | --- | --- | --- | --- | --- | --- | --- | --- | --- | --- | --- | --- | --- | --- | --- | --- | --- | --- | --- | --- | --- | --- | --- | --- | --- | --- | --- | --- | --- | --- | --- | --- | --- | --- | --- | --- | --- | --- | --- | --- | --- | --- | --- | --- | --- | --- | --- | --- | --- | --- | --- | --- | --- | --- | --- | --- | --- | --- | --- | --- | --- | --- | --- | --- | --- | --- | --- | --- | --- | --- | --- | --- | --- | --- | --- | --- | --- | --- | --- | --- | --- | --- | --- | --- | --- | --- | --- | --- | --- | --- | --- | --- | --- | --- | --- | --- | --- | --- | --- | --- | --- | --- | --- | --- | --- | --- | --- | --- | --- | --- | --- | --- | --- | --- | --- | --- | --- | --- | --- | --- | --- | --- | --- | --- | --- | --- | --- | --- | --- | --- | --- | --- | --- | --- | --- | --- | --- | --- | --- | --- | --- | --- | --- | --- | --- | --- | --- | --- | --- | --- | --- | --- | --- | --- | --- | --- | --- | --- | --- | --- | --- | --- | --- | --- | --- | --- | --- | --- | --- | --- | --- | --- | --- | --- | --- | --- | --- | --- | --- | --- | --- | --- | --- | --- | --- | --- | --- | --- | --- | --- | --- | --- | --- | --- | --- | --- | --- | --- | --- | --- | --- | --- | --- | --- | --- | --- | --- | --- | --- | --- | --- | --- | --- | --- | --- | --- | --- | --- | --- | --- | --- | --- | --- | --- | --- | --- | --- | --- | --- | --- | --- | --- | --- | --- | --- | --- | --- | --- | --- | --- | --- | --- | --- | --- | --- | --- | --- | --- | --- | --- | --- | --- | --- | --- | --- | --- | --- | --- | --- | --- | --- | --- | --- | --- | --- | --- | --- | --- | --- | --- | --- | --- | --- | --- | --- | --- | --- | --- | --- | --- | --- | --- | --- | --- | --- | --- | --- | --- | --- | --- | --- | --- | --- | --- | --- | --- | --- | --- | --- | --- | --- | --- | --- | --- | --- | --- | --- | --- | --- | --- | --- | --- | --- | --- | --- | --- | --- | --- | --- | --- | --- | --- | --- | --- | --- | --- | --- | --- | --- | --- | --- | --- | --- | --- | --- | --- | --- | --- | --- | --- | --- | --- | --- | --- | --- | --- | --- | --- | --- | --- | --- | --- | --- | --- | --- | --- | --- | --- | --- | --- | --- | --- | --- | --- | --- | --- | --- | --- | --- | --- | --- | --- | --- | --- | --- | --- | --- | --- | --- | --- | --- | --- | --- | --- | --- | --- | --- | --- | --- | --- | --- | --- | --- | --- | --- | --- | --- | --- | --- | --- | --- | --- | --- | --- | --- | --- | --- | --- | --- | --- | --- | --- | --- | --- | --- | --- | --- | --- | --- | --- | --- | --- | --- | --- | --- | --- | --- | --- | --- | --- | --- | --- | --- | --- | --- | --- | --- | --- | --- | --- | --- | --- | --- | --- | --- | --- | --- | --- | --- | --- | --- | --- | --- | --- | --- | --- | --- | --- | --- | --- | --- | --- | --- | --- | --- | --- | --- | --- | --- | --- | --- | --- | --- | --- | --- | --- | --- | --- | --- | --- | --- | --- | --- | --- | --- | --- | --- | --- | --- | --- | --- | --- | --- | --- | --- | --- | --- | --- | --- | --- | --- | --- | --- | --- | --- | --- | --- | --- | --- | --- | --- | --- | --- | --- | --- | --- | --- | --- | --- | --- | --- | --- | --- | --- | --- | --- | --- | --- | --- | --- | --- | --- | --- | --- | --- | --- | --- | --- | --- | --- | --- | --- | --- | --- | --- | --- | --- | --- | --- | --- | --- | --- | --- | --- | --- | --- | --- | --- | --- | --- | --- | --- | --- | --- | --- | --- | --- | --- | --- | --- | --- | --- | --- | --- | --- | --- | --- | --- | --- | --- | --- | --- | --- | --- | --- | --- | --- | --- | --- | --- | --- | --- | --- | --- | --- | --- | --- | --- | --- | --- | --- | --- | --- | --- | --- | --- | --- | --- | --- | --- | --- | --- | --- | --- | --- | --- | --- | --- | --- | --- | --- | --- | --- | --- | --- | --- | --- | --- | --- | --- | --- | --- | --- | --- | --- | --- | --- | --- | --- | --- | --- | --- | --- | --- | --- | --- | --- | --- | --- | --- | --- | --- | --- | --- | --- | --- | --- | --- | --- | --- | --- | --- | --- | --- | --- | --- | --- | --- | --- | --- | --- | --- | --- | --- | --- | --- | --- | --- | --- | --- | --- | --- | --- |

**Supplementary Table 3: Top ranked schizophrenia-associated differently methylated probes (DMPs) identified in the striatum (STR) meta-analysis.** Listed for each STR DMP (grey) are corresponding results from the prefrontal cortex (PFC; *P* < 0.05 in blue), hippocampus (HC; *P* < 0.05 in red) and cerebellum (CER; *P* < 0.05 in orange) meta-analyses (PFC, STR and CER) or linear regression (HC). Also shown is the association with schizophrenia polygenic burden in STR (*P* < 0.05 in green). Illumina and Genomic Regions Enrichment of Annotation Tool (GREAT) annotation[^1^](#_ENREF_1) is listed for each DMP.

| **Probe ID** | **Genomic position (hg19)** | | **Illumina gene annotation** | **Gene region** | **GREAT annotation** [**^1^**](#_ENREF_1) | **Methylation difference (%) STR** | ***P*  STR** | **Methylation difference PFC (%)** | ***P* PFC** | **Methylation difference HC (%)** | ***P*  HC** | **Methylation difference CER (%)** | ***P*  CER** | **Polygenic risk score STR methylation difference (%)** | **Polygenic risk score STR *P*** |
| --- | --- | --- | --- | --- | --- | --- | --- | --- | --- | --- | --- | --- | --- | --- | --- |
| cg08103144 | chr5:150028986 | SYNPO | | Body | MYOZ3; SYNPO | -3.17 | 3.64E-08 | 0.68 | 0.21 | 0.24 | 0.84 | 0.83 | 0.29 | -0.12 | 0.35 |
| cg03847432 | chr7:43391524 | HECW1 | | Body | STK17A; HECW1 | 2.71 | 5.23E-08 | 0.74 | 0.31 | 3.30 | 0.01 | 1.32 | 0.18 | 0.06 | 0.60 |
| cg22182016 | chr15:57998894 | GRINL1A; GCOM1 | | TSS200; Body | POLR2M | -0.51 | 5.67E-08 | -0.05 | 0.55 | 0.17 | 0.34 | -0.13 | 0.10 | 0.01 | 0.59 |
| cg25361651 | chr20:29847402 | DEFB115 | | Body | DEFB115; DEFB116 | 4.43 | 6.52E-08 | 1.03 | 0.32 | -1.80 | 0.46 | - | - | 0.03 | 0.88 |
| cg22221320 | chr1:89664340 | GBP4 | | Body | GBP4 | 6.93 | 7.88E-08 | 3.75 | 0.02 | -0.05 | 0.99 | 2.68 | 0.12 | 0.03 | 0.88 |
| cg02049663 | chr7:99686396 | COPS6 | | TSS200 | COPS6 | -0.64 | 3.76E-07 | -0.27 | 0.03 | 0.13 | 0.74 | -0.24 | 0.10 | -0.04 | 0.13 |
| cg23245620 | chr2:45172972 | SIX3 | | 3'UTR | SIX3; SIX2 | 3.49 | 4.47E-07 | -0.78 | 0.44 | 2.48 | 0.14 | -1.09 | 0.15 | 0.17 | 0.19 |
| cg07514654 | chr7:157258062 | - | | - | DNAJB6 | -4.07 | 8.90E-07 | -2.63 | 0.04 | -0.33 | 0.89 | -1.81 | 0.13 | -0.28 | 0.13 |
| cg01663682 | chr15:93447777 | CHD2 | | Body | CHD2; RGMA | -1.26 | 1.06E-06 | -0.10 | 0.64 | -1.54 | 0.05 | -0.49 | 0.04 | -0.09 | 0.08 |
| cg27203372 | chr7:2638310 | IQCE | | Body | TTYH3; IQCE | 1.48 | 1.26E-06 | 0.26 | 0.57 | 1.09 | 0.19 | 0.08 | 0.84 | 0.10 | 0.10 |
| cg02454364 | chr1:236156917 | NID1 | | Body | LYST; NID1 | -2.86 | 1.36E-06 | 0.07 | 0.58 | -0.16 | 0.64 | -0.46 | 0.08 | -0.25 | 0.02 |
| cg24741713 | chr5:59064389 | PDE4D | | Body | PDE4D | -1.30 | 2.76E-06 | 0.18 | 0.60 | -1.64 | 0.02 | -0.26 | 0.44 | -0.08 | 0.09 |
| cg03226218 | chr12:57082170 | PTGES3 | | TSS200 | PTGES3 | -0.59 | 2.80E-06 | 0.03 | 0.81 | -0.36 | 0.37 | -0.03 | 0.88 | -0.03 | 0.18 |
| cg02443072 | chr17:37183683 | - | | - | PLXDC1; LASP1 | -1.62 | 3.32E-06 | -1.32 | 0.01 | -1.08 | 0.10 | -0.41 | 0.34 | -0.02 | 0.76 |
| cg03804621 | chr10:124638756 | FAM24B; LOC399815 | | 5'UTR; TSS1500 | FAM24B | 5.44 | 3.71E-06 | 4.36 | 0.002 | 1.46 | 0.52 | 6.02 | 6.58E-05 | 0.15 | 0.44 |
| cg21365602 | chr1:89664407 | GBP4 | | Body | GBP4 | 6.23 | 4.99E-06 | 4.46 | 8.42E-04 | 0.54 | 0.86 | 4.49 | 5.97E-04 | 0.07 | 0.76 |
| cg16685608 | chr14:52211579 | - | | - | GNG2; FRMD6 | 3.48 | 5.06E-06 | 2.20 | 0.02 | -0.94 | 0.50 | 1.24 | 0.30 | 0.12 | 0.40 |
| cg18122392 | chr2:99013409 | CNGA3 | | Body | INPP4A; CNGA3 | -1.99 | 5.37E-06 | 0.67 | 0.18 | -0.54 | 0.40 | 0.04 | 0.94 | -0.09 | 0.23 |
| cg12253200 | chr19:49123013 | RPL18; SPHK2 | | TSS1500; 5'UTR | RPL18; SPHK2 | -1.00 | 5.55E-06 | -0.29 | 0.19 | -0.99 | 0.04 | -0.35 | 0.17 | -0.09 | 0.02 |
| cg18116486 | chr14:58667316 | ACTR10 | | Body | ACTR10 | -0.84 | 5.95E-06 | -0.21 | 0.33 | -0.54 | 0.22 | 0.15 | 0.53 | -0.01 | 0.88 |
| cg05612904 | chr1:101491636 | DPH5 | | TSS1500 | DPH5 | -0.64 | 8.13E-06 | -0.04 | 0.78 | -1.14 | 0.10 | -0.04 | 0.78 | -0.08 | 3.55E-03 |
| cg05461666 | chr1:20573259 | - | | - | PLA2G2C; VWA5B1 | 1.36 | 8.45E-06 | 0.63 | 0.03 | 2.03 | 0.40 | 0.30 | 0.32 | 0.14 | 0.04 |
| cg06768993 | chr4:8443412 | ACOX3 | | TSS1500 | ACOX3; METTL19 | 3.28 | 8.57E-06 | 2.03 | 0.03 | 1.27 | 0.46 | 3.37 | 9.12E-04 | 0.19 | 0.18 |
| cg24688803 | chr12:105478590 | ALDH1L2 | | TSS1500 | ALDH1L2 | 2.66 | 1.03E-05 | 1.62 | 0.05 | 0.46 | 0.78 | -0.85 | 0.46 | 0.04 | 0.68 |
| cg15607358 | chr12:372049 | SLC6A13 | | TSS200 | SLC6A13 | 4.49 | 1.03E-05 | -0.91 | 0.41 | 0.26 | 0.87 | -0.73 | 0.53 | 0.42 | 0.01 |
| cg15559640 | chr15:89010445 | MRPS11; MRPL46 | | TSS1500; 1stExon | MRPS11; MRPL46 | -0.98 | 1.18E-05 | -0.26 | 0.22 | -0.61 | 0.17 | -0.22 | 0.31 | -0.01 | 0.76 |
| cg18803856 | chr8:1495169 | DLGAP2 | | 5'UTR | CLN8; DLGAP2 | 1.73 | 1.37E-05 | 0.83 | 0.09 | -0.80 | 0.44 | 0.36 | 0.35 | 0.01 | 0.94 |
| cg25924911 | chr3:45838094 | SLC6A20 | | TSS200 | SLC6A20 | 0.69 | 1.38E-05 | -0.11 | 0.34 | -0.04 | 0.88 | 0.05 | 0.78 | 0.07 | 0.02 |
| cg22513099 | chr8:117788299 | - | | - | UTP23; RAD21 | 3.93 | 1.39E-05 | -0.19 | 0.85 | 3.70 | 0.13 | 3.12 | 4.93E-03 | 0.46 | 5.70E-03 |
| cg13938909 | chr1:89873226 | LOC400759 | | TSS200 | LRRC8B; GBP6 | 4.35 | 1.44E-05 | 2.08 | 0.02 | 3.25 | 0.22 | - | - | 0.29 | 0.13 |
| cg26823162 | chr19:55792075 | HSPBP1 | | TSS1500 | BRSK1; HSPBP1 | 1.44 | 1.47E-05 | 0.74 | 0.05 | 0.52 | 0.54 | 0.77 | 0.14 | 0.10 | 0.08 |
| cg11225745 | chr1:22927925 | EPHA8 | | Body | C1QA; EPHA8 | 1.73 | 1.49E-05 | -0.14 | 0.73 | 0.55 | 0.50 | -0.79 | 0.03 | 8.95E-03 | 0.89 |
| cg11584284 | chr6:130690629 | - | | - | TMEM200A; L3MBTL3 | 1.98 | 1.49E-05 | -0.36 | 0.51 | 0.26 | 0.83 | 0.35 | 0.71 | 0.11 | 0.20 |
| cg07500432 | chr18:77918588 | PARD6G; LOC100130522 | | Body | ADNP2; PARD6G | 4.64 | 1.54E-05 | 6.69 | 6.25E-05 | 5.11 | 0.001 | 3.14 | 0.04 | 0.50 | 6.50E-03 |
| cg04682911 | chr8:59971099 | TOX | | Body | NSMAF; TOX | -1.35 | 1.66E-05 | -0.01 | 0.97 | 0.83 | 0.33 | 0.70 | 0.19 | -0.08 | 0.19 |
| cg10207277 | chr8:114449243 | CSMD3 | | TSS200 | CSMD3 | -0.83 | 1.80E-05 | 0.01 | 0.98 | 0.32 | 0.46 | -0.08 | 0.73 | -0.05 | 0.16 |
| cg04278794 | chr16:3406339 | OR2C1 | | 1stExon | OR2C1 | 3.11 | 1.97E-05 | 0.48 | 0.62 | 1.44 | 0.33 | 1.78 | 0.06 | 0.24 | 0.08 |
| cg11794120 | chr7:2087905 | MAD1L1 | | Body | MAD1L1; ELFN1 | -1.43 | 2.03E-05 | -0.97 | 0.04 | -0.93 | 0.42 | 1.25 | 4.09E-03 | -0.05 | 0.38 |
| cg00996764 | chr19:51382591 | KLK2 | | 3'UTR | KLK2; KLK4 | 1.82 | 2.16E-05 | -0.32 | 0.45 | -0.39 | 0.66 | 0.80 | 0.19 | 0.02 | 0.77 |
| cg07777224 | chr19:58919807 | ZNF584 | | TSS1500 | ZNF584 | -1.41 | 2.37E-05 | -0.05 | 0.82 | 1.09 | 0.35 | -0.23 | 0.46 | -0.03 | 0.59 |
| cg21008684 | chr2:157198370 | - | | - | GPD2; NR4A2 | -0.76 | 2.54E-05 | -0.13 | 0.55 | -0.38 | 0.19 | -0.05 | 0.82 | -0.08 | 0.02 |
| cg01837362 | chr12:34492938 | - | | - | ALG10 | 5.26 | 2.56E-05 | 4.78 | 5.15E-04 | 5.90 | 0.03 | 6.01 | 8.20E-05 | 0.24 | 0.30 |
| cg09063683 | chr5:37890150 | - | | - | EGFLAM; GDNF | -3.24 | 2.70E-05 | -0.99 | 0.19 | -5.56 | 8.44E-04 | -1.52 | 0.05 | -0.26 | 0.04 |
| cg10042645 | chr11:2308589 | - | | - | ASCL2; C11orf21 | 2.16 | 2.70E-05 | 0.25 | 0.66 | 2.81 | 0.01 | 0.54 | 0.36 | 0.05 | 0.65 |
| cg18743464 | chr2:131089942 | - | | - | TUBA3E; CCDC115 | 2.73 | 2.71E-05 | 1.34 | 0.05 | 0.53 | 0.68 | 1.53 | 0.18 | 0.10 | 0.42 |
| cg13897348 | chr1:1549699 | MIB2 | | TSS1500 | MIB2 | 4.57 | 2.73E-05 | -0.45 | 0.69 | -0.01 | 1.00 | -1.56 | 0.11 | 0.34 | 0.05 |
| cg15212418 | chr17:155045 | RPH3AL | | Body | DOC2B; RPH3AL | -9.74 | 2.86E-05 | -7.39 | 1.24E-03 | -9.06 | 0.07 | -9.30 | 2.12E-03 | -0.65 | 0.09 |
| cg13647960 | chr10:68582091 | CTNNA3 | | Body | LRRTM3 | 2.93 | 2.92E-05 | - | - | -0.89 | 0.58 | - | - | 0.24 | 0.07 |
| cg21196747 | chr6:27521385 | - | | - | ZNF184; HIST1H2BL | -2.93 | 2.99E-05 | -2.25 | 0.01 | -0.79 | 0.71 | -3.11 | 2.18E-04 | -0.19 | 0.12 |
| cg18444231 | chr17:55058723 | SCPEP1 | | Body | AKAP1; SCPEP1 | 3.76 | 3.02E-05 | 1.84 | 0.05 | 6.77 | 0.003 | -0.03 | 0.98 | 0.48 | 1.61E-03 |

**Supplementary Table 4: Top ranked schizophrenia-associated differently methylated probes (DMPs) identified in the hippocampus (HC) linear regression analysis.** Listed for each HC DMP (grey) are corresponding results from the prefrontal cortex (PFC; *P* < 0.05 in blue), striatum (STR; *P* < 0.05 in green) and cerebellum (CER; *P* < 0.05 in orange) meta-analyses. Also shown is the association with schizophrenia polygenic burden in HC (*P* < 0.05 in red). Illumina and Genomic Regions Enrichment of Annotation Tool (GREAT) annotation[^1^](#_ENREF_1) is listed for each DMP.

| **Probe ID** | **Genomic position (hg19)** | **Illumina gene annotation** | **Gene region** | **GREAT annotation** [**^1^**](#_ENREF_1) | **Methylation difference (%) HC** | ***P* HC** | **Methylation difference PFC (%)** | ***P* PFC** | **Methylation difference STR (%)** | ***P* STR** | **Methylation difference CER (%)** | ***P* CER** | **Polygenic risk score HC methylation difference (%)** | **Polygenic risk score HC *P*** |
| --- | --- | --- | --- | --- | --- | --- | --- | --- | --- | --- | --- | --- | --- | --- |
| cg07751266 | chr16:67515323 | ATP6V0D1 | TSS1500 | ATP6V0D1 | -1.35 | 1.27E-07 | -0.13 | 0.41 | -0.27 | 0.30 | -0.26 | 0.21 | -0.16 | 7.82E-04 |
| cg10383028 | chr17:48638097 | CACNA1G | TSS1500 | CACNA1G | -0.59 | 5.30E-06 | -0.09 | 0.29 | 0.02 | 0.84 | 0.17 | 0.12 | -0.06 | 8.15E-03 |
| cg20871346 | chr6:17021625 | - | - | RBM24; ATXN1 | -6.14 | 9.89E-06 | 0.46 | 0.41 | -0.47 | 0.53 | -0.09 | 0.91 | -0.58 | 0.01 |
| cg01422136 | chr5:132362224 | ZCCHC10 | 1stExon | ZCCHC10 | -1.56 | 1.75E-05 | -0.03 | 0.89 | 0.05 | 0.82 | 0.13 | 0.56 | -0.23 | 5.50E-05 |
| cg23082877 | chr19:49243427 | RASIP1 | Body | RASIP1 | -4.37 | 2.03E-05 | 0.01 | 0.98 | -0.55 | 0.51 | -1.42 | 0.08 | -0.48 | 6.52E-03 |
| cg16250023 | chr17:58470033 | USP32 | TSS1500 | USP32 | -0.92 | 2.71E-05 | 0.02 | 0.90 | -0.22 | 0.11 | -0.09 | 0.59 | -0.12 | 3.79E-04 |
| cg25119073 | chr19:23870151 | ZNF675 | TSS200 | ZNF675 | -2.56 | 2.87E-05 | -0.12 | 0.78 | -0.43 | 0.30 | 0.11 | 0.84 | -0.35 | 7.24E-04 |
| cg23860886 | chr6:18155593 | KDM1B; TPMT | TSS200; TSS1500 | TPMT; KDM1B | -1.05 | 2.89E-05 | -0.06 | 0.68 | 0.14 | 0.53 | -0.02 | 0.90 | -0.09 | 0.01 |
| cg23201032 | chr3:134369828 | KY | 5'UTR; 1stExon | KY | -0.91 | 2.91E-05 | 0.27 | 0.18 | -0.13 | 0.38 | 0.10 | 0.52 | -0.13 | 3.48E-04 |
| cg05092310 | chr17:43226346 | HEXIM1 | 1stExon; 5'UTR | HEXIM2; HEXIM1 | -1.01 | 3.56E-05 | -0.17 | 0.32 | -0.18 | 0.46 | 0.32 | 0.15 | -0.11 | 7.60E-03 |
| cg19115272 | chr6:30139538 | TRIM15 | Body | TRIM10; TRIM26 | -1.06 | 3.95E-05 | -0.02 | 0.91 | -0.05 | 0.67 | 0.02 | 0.90 | -0.13 | 1.58E-03 |
| cg07158797 | chr1:215740701 | KCTD3 | TSS200 | KCTD3 | 1.37 | 4.22E-05 | -0.05 | 0.66 | -0.22 | 0.18 | -0.09 | 0.56 | 0.20 | 6.92E-05 |
| cg05167468 | chr15:21905516 | - | - | OR4M2 | 2.37 | 4.39E-05 | 0.11 | 0.72 | -0.14 | 0.72 | 0.25 | 0.58 | 0.33 | 3.48E-04 |
| cg08266474 | chr6:84904847 | KIAA1009 | Body | KIAA1009; MRAP2 | 5.21 | 4.52E-05 | 0.29 | 0.59 | 0.72 | 0.25 | 1.38 | 0.02 | 0.33 | 0.13 |
| cg21106136 | chr3:194405973 | FAM43A | TSS1500 | LSG1; XXYLT1 | 7.73 | 4.65E-05 | -0.04 | 0.96 | 1.52 | 0.15 | - | - | 0.96 | 3.74E-03 |
| cg12167135 | chr17:80573887 | WDR45L | Body | WDR45L; FOXK2 | -3.96 | 5.23E-05 | 0.03 | 0.98 | -0.27 | 0.75 | 0.79 | 0.33 | -0.35 | 0.03 |
| cg13590055 | chr18:77917647 | LOC100130522; PARD6G | Body; 3'UTR | ADNP2; PARD6G | 12.85 | 5.80E-05 | 8.89 | 1.33E-04 | 7.99 | 6.91E-05 | 4.18 | 0.01 | 1.33 | 9.52E-03 |
| cg20195319 | chr7:47568297 | TNS3 | 5'UTR | TNS3 | 1.88 | 5.99E-05 | 0.00 | 1.00 | 0.49 | 0.07 | 0.03 | 0.95 | 0.14 | 0.10 |
| cg01310473 | chr7:76829168 | CCDC146; FGL2 | Body; TSS200 | FGL2 | 3.58 | 6.15E-05 | -0.44 | 0.55 | 1.37 | 0.03 | -1.95 | 0.07 | 0.39 | 0.02 |
| cg19815565 | chr18:77917615 | LOC100130522; PARD6G | Body; 3'UTR | ADNP2; PARD6G | 12.76 | 6.24E-05 | 8.06 | 6.77E-04 | 8.57 | 2.23E-04 | 4.03 | 0.06 | 1.24 | 0.02 |
| cg01657493 | chr2:64681082 | HSPC159 | TSS1500 | LGALSL | -2.05 | 7.10E-05 | 0.35 | 0.19 | -0.41 | 0.28 | -0.03 | 0.92 | -0.23 | 4.04E-03 |
| cg18938150 | chr17:42144162 | LSM12 | 5'UTR | G6PC3 | -1.12 | 7.37E-05 | 0.05 | 0.77 | 0.11 | 0.49 | -0.19 | 0.40 | -0.16 | 7.48E-04 |
| cg03075791 | chr2:120774652 | EPB41L5 | 5'UTR | EPB41L5; TMEM185B | 2.96 | 7.94E-05 | 0.39 | 0.60 | 0.11 | 0.83 | 0.68 | 0.25 | 0.48 | 2.85E-06 |
| cg02851167 | chr8:143378153 | TSNARE1 | Body | FLJ43860; TSNARE1 | 3.52 | 8.30E-05 | -0.32 | 0.61 | 0.43 | 0.45 | 0.60 | 0.42 | 0.31 | 0.04 |
| cg09851072 | chr16:4749498 | ANKS3 | Body | NUDT16L1; ZNF500 | -2.02 | 8.42E-05 | -0.26 | 0.46 | 0.28 | 0.54 | -0.37 | 0.46 | -0.17 | 0.06 |
| cg08681110 | chr6:114178501 | MARCKS | TSS200 | MARCKS | -2.11 | 8.98E-05 | 0.18 | 0.51 | -0.18 | 0.54 | 0.74 | 9.61E-04 | -0.20 | 0.03 |
| cg07152487 | chr11:14665355 | PDE3B; PSMA1 | 1stExon; TSS200; 5'UTR | PDE3B | 0.99 | 9.08E-05 | -0.09 | 0.48 | -0.03 | 0.89 | 0.17 | 0.34 | 0.09 | 0.04 |
| cg24023498 | chr2:157199345 | - | - | GPD2; NR4A2 | -2.26 | 9.08E-05 | -0.69 | 0.07 | -0.23 | 0.71 | 0.28 | 0.40 | -0.23 | 0.01 |
| cg05134775 | chr10:98273510 | TLL2 | 1stExon; 5'UTR | TLL2 | 0.81 | 9.25E-05 | 0.07 | 0.61 | -0.15 | 0.22 | -0.11 | 0.36 | 0.12 | 8.42E-04 |
| cg04044983 | chr16:67280079 | FHOD1 | Body | SLC9A5; FHOD1 | -1.51 | 1.06E-04 | -0.24 | 0.41 | -0.01 | 0.97 | -0.25 | 0.46 | -0.14 | 0.04 |
| cg18489266 | chr11:59323918 | - | - | OR4D9; OSBP | 4.56 | 1.17E-04 | -1.19 | 0.02 | -0.48 | 0.51 | 0.29 | 0.62 | 0.41 | 0.06 |
| cg12591668 | chr1:192520335 | - | - | RGS1; RGS21 | 4.52 | 1.18E-04 | 0.53 | 0.49 | 1.02 | 0.11 | -0.35 | 0.63 | 0.53 | 5.84E-03 |
| cg03532879 | chr20:37209395 | ADIG | TSS1500 | ADIG | 6.73 | 1.23E-04 | -0.22 | 0.70 | 1.41 | 0.14 | -0.48 | 0.47 | 0.65 | 0.02 |
| cg24122364 | chr13:99574736 | DOCK9 | Body | SLC15A1; DOCK9 | -10.93 | 1.27E-04 | -1.08 | 0.40 | -1.37 | 0.40 | -0.11 | 0.93 | -1.55 | 5.72E-04 |
| cg15193475 | chr15:80998711 | FAM108C1 | Body | KIAA1199; FAM108C1 | 3.65 | 1.37E-04 | 1.06 | 0.11 | 1.26 | 0.07 | 1.33 | 0.02 | 0.52 | 8.53E-04 |
| cg01896926 | chr17:685509 | GLOD4; RNMTL1 | 5'UTR; 1stExon; TSS200 | RNMTL1; GLOD4 | -0.49 | 1.41E-04 | 0.03 | 0.84 | -0.07 | 0.44 | 0.24 | 0.08 | -0.06 | 3.20E-03 |
| cg21328651 | chr7:158742190 | - | - | ESYT2; VIPR2 | 2.35 | 1.42E-04 | 1.37 | 0.02 | 0.50 | 0.13 | -0.20 | 0.66 | 0.19 | 0.06 |
| cg09414612 | chr2:85838835 | C2orf68 | Body | USP39 | -0.81 | 1.42E-04 | 0.13 | 0.49 | -0.05 | 0.57 | 0.23 | 0.03 | -0.11 | 1.97E-03 |
| cg22573675 | chr2:38762739 | - | - | ATL2; HNRPLL | -3.36 | 1.42E-04 | -0.10 | 0.81 | 0.38 | 0.39 | 1.07 | 0.35 | -0.47 | 1.14E-03 |
| cg22761176 | chr2:173539542 | - | - | RAPGEF4; PDK1 | -6.09 | 1.44E-04 | -1.14 | 0.29 | 0.41 | 0.67 | 0.37 | 0.75 | -0.65 | 0.02 |
| cg00545229 | chr4:15429531 | C1QTNF7 | TSS200; Body; 5'UTR | C1QTNF7 | -3.53 | 1.46E-04 | 0.66 | 0.36 | 0.60 | 0.35 | 1.32 | 3.08E-03 | -0.35 | 0.03 |
| cg12271317 | chr4:108972693 | LEF1 | 3'UTR; Body | HADH; LEF1 | -3.33 | 1.47E-04 | 0.41 | 0.26 | 0.14 | 0.76 | -0.22 | 0.77 | -0.40 | 4.01E-03 |
| cg23213170 | chr9:108320507 | FKTN | 5'UTR | FKTN | -1.37 | 1.47E-04 | 0.05 | 0.75 | -0.20 | 0.26 | -0.30 | 0.14 | -0.16 | 4.40E-03 |
| cg25512683 | chr17:41003399 | AOC3 | 1stExon | AOC3 | 3.59 | 1.47E-04 | -0.58 | 0.38 | -0.48 | 0.44 | 0.64 | 0.34 | 0.43 | 4.84E-03 |
| cg24799451 | chr11:134093757 | NCAPD3; VPS26B | 1stExon; TSS1500 | VPS26B; NCAPD3 | -1.07 | 1.48E-04 | -0.18 | 0.28 | -0.22 | 0.34 | 0.03 | 0.87 | -0.09 | 0.06 |
| cg09442740 | chr7:100482960 | SRRT | Body | UFSP1; SRRT | 1.60 | 1.48E-04 | 0.34 | 0.15 | 0.25 | 0.77 | -0.51 | 0.21 | 0.23 | 1.09E-04 |
| cg12859716 | chr17:6552190 | MED31 | Body | KIAA0753; MED31 | 4.41 | 1.53E-04 | 0.58 | 0.24 | 0.25 | 0.63 | 0.36 | 0.54 | 0.45 | 0.03 |
| cg08634721 | chr13:30169482 | SLC7A1 | 5'UTR | SLC7A1 | -1.77 | 1.54E-04 | - | - | -0.11 | 0.64 | -0.13 | 0.64 | -0.21 | 4.07E-03 |
| cg25201910 | chr3:138327818 | FAIM | 5'UTR; TSS200 | FAIM | -2.77 | 1.59E-04 | -0.39 | 0.40 | -0.05 | 0.90 | -0.44 | 0.37 | -0.36 | 1.42E-03 |
| cg12799790 | chr10:54071134 | - | - | DKK1 | 6.39 | 1.61E-04 | - | - | 1.86 | 0.03 | 1.22 | 0.35 | 0.74 | 9.36E-03 |

**Supplementary Table 5: Top ranked schizophrenia-associated differently methylated probes (DMPs) identified in the cerebellum (CER) meta-analysis.** Listed for each CER DMP (grey) are corresponding results from the prefrontal cortex (PFC; *P* < 0.05 in blue), striatum (STR; *P* < 0.05 in green) and hippocampus (HC; *P* < 0.05 in red) meta-analyses (PFC, STR and CER) or linear regression (HC). Also shown is the association with schizophrenia polygenic burden in CER (*P* < 0.05 in orange). Illumina and Genomic Regions Enrichment of Annotation Tool (GREAT) annotation[^1^](#_ENREF_1) is listed for each DMP.

| **Probe ID** | **Genomic position (hg19)** | **Illumina gene annotation** | **Gene region** | **GREAT annotation** [**^1^**](#_ENREF_1) | **Methylation difference (%) CER** | ***P* CER** | **Methylation difference PFC (%)** | ***P* PFC** | **Methylation difference STR (%)** | ***P* STR** | **Methylation difference HC (%)** | ***P* HC** | **Polygenic risk score CER methylation difference (%)** | **Polygenic risk score CER *P*** |
| --- | --- | --- | --- | --- | --- | --- | --- | --- | --- | --- | --- | --- | --- | --- |
| cg14609448 | chr21:34896882 | GART | Body; 3'UTR | DNAJC28; GART | 2.29 | 2.48E-08 | 0.15 | 0.81 | -0.02 | 0.95 | -0.57 | 0.70 | 0.19 | 0.02 |
| cg01757160 | chr12:96588951 | ELK3 | 5'UTR | ELK3 | 0.56 | 5.74E-08 | 0.15 | 0.31 | 0.03 | 0.88 | -0.30 | 0.44 | 0.04 | 0.04 |
| cg09757430 | chr13:28397122 | - | - | PDX1; GSX1 | -3.13 | 1.37E-07 | 1.21 | 0.23 | -0.31 | 0.77 | -2.05 | 0.38 | -0.18 | 0.13 |
| cg20751795 | chr19:58281019 | ZNF586 | TSS200 | ZNF586 | 0.57 | 1.64E-07 | 0.18 | 0.10 | -0.02 | 0.89 | -0.44 | 0.03 | 0.05 | 0.01 |
| cg01692482 | chr20:52198378 | ZNF217 | 1stExon | ZNF217; TSHZ2 | 2.51 | 2.30E-07 | 0.12 | 0.87 | -1.05 | 0.09 | -0.69 | 0.47 | 0.19 | 0.05 |
| cg02688226 | chr5:68665548 | RAD17; TAF9 | TSS200; 1stExon; 5'UTR; TSS1500 | RAD17; TAF9 | -0.51 | 2.84E-07 | -0.17 | 0.10 | -4.30E-03 | 0.97 | -0.20 | 0.38 | -0.02 | 0.43 |
| cg02966813 | chr19:52495464 | ZNF615 | 3'UTR | ZNF350; ZNF615 | 3.42 | 3.47E-07 | 0.72 | 0.41 | 0.02 | 0.98 | 1.17 | 0.51 | 0.17 | 0.23 |
| cg23055921 | chr7:26112215 | - | - | NPVF; NFE2L3 | 4.18 | 3.49E-07 | 1.58 | 0.06 | 0.71 | 0.40 | 2.36 | 0.22 | 0.40 | 0.01 |
| cg22230538 | chr8:28258841 | - | - | FZD3; ZNF395 | 0.45 | 4.59E-07 | 0.18 | 0.06 | 0.13 | 0.25 | -0.24 | 0.42 | 0.03 | 0.10 |
| cg10432626 | chr10:72033567 | - | - | PPA1; NPFFR1 | 2.09 | 5.09E-07 | 0.53 | 0.26 | 0.15 | 0.77 | 1.80 | 0.19 | 0.16 | 0.09 |
| cg18618431 | chr8:134531916 | ST3GAL1 | 5'UTR | NDRG1; ST3GAL1 | 2.36 | 5.65E-07 | -0.90 | 0.10 | -0.29 | 0.51 | 1.34 | 0.20 | 0.27 | 3.20E-03 |
| cg10589310 | chr5:23507030 | PRDM9 | TSS1500 | PRDM9 | 5.08 | 8.37E-07 | 1.88 | 0.02 | 2.12 | 0.01 | 3.53 | 0.03 | 0.51 | 1.14E-03 |
| cg12830694 | chr19:38747796 | PPP1R14A | TSS1500 | PPP1R14A | 1.68 | 1.13E-06 | 0.44 | 0.20 | 0.42 | 0.20 | -0.36 | 0.53 | 0.12 | 0.14 |
| cg22530668 | chr1:5919081 | - | - | NPHP4 | 2.00 | 1.13E-06 | 0.59 | 0.18 | 0.40 | 0.45 | -0.29 | 0.76 | 0.08 | 0.35 |
| cg13043509 | chr7:150434452 | GIMAP5 | 1stExon; 5'UTR | GIMAP1-GIMAP5; TMEM176B | 4.36 | 1.44E-06 | 1.94 | 0.05 | 0.79 | 0.36 | -0.15 | 0.91 | 0.07 | 0.69 |
| cg10303653 | chr6:32049516 | TNXB | Body | TNXB; CYP21A2 | -5.91 | 1.66E-06 | 0.49 | 0.55 | -0.15 | 0.84 | -1.78 | 0.06 | 0.00 | 1.00 |
| cg20220242 | chr21:30392188 | RWDD2B | TSS1500 | USP16; RWDD2B | 5.92 | 1.82E-06 | 3.15 | 0.02 | 3.28 | 4.13E-03 | 1.22 | 0.63 | 0.47 | 0.04 |
| cg22401939 | chr1:206731166 | RASSF5 | Body | RASSF5; EIF2D | -3.73 | 2.06E-06 | -0.18 | 0.81 | 0.78 | 0.38 | 0.60 | 0.74 | -0.35 | 0.02 |
| cg22135102 | chr6:32036897 | TNXB | Body | CYP21A2; TNXB | -2.99 | 2.20E-06 | 0.16 | 0.76 | 0.11 | 0.83 | -0.21 | 0.86 | -0.09 | 0.42 |
| cg00208830 | chr1:183154778 | LAMC2 | TSS1500 | LAMC2 | -3.54 | 2.24E-06 | -0.70 | 0.19 | -0.16 | 0.78 | -1.65 | 0.16 | -0.15 | 0.26 |
| cg13259118 | chr14:62161958 | HIF1A | TSS200 | HIF1A | -0.77 | 2.31E-06 | -0.33 | 0.03 | -0.16 | 0.32 | -0.60 | 0.16 | -0.05 | 0.11 |
| cg02397497 | chr6:10412073 | TFAP2A | Body; 5'UTR; 1stExon | OFCC1; TFAP2A | 1.23 | 2.73E-06 | -0.52 | 0.45 | -0.16 | 0.72 | 0.66 | 0.51 | 0.06 | 0.21 |
| cg08917060 | chr1:197872294 | C1orf53 | Body | DENND1B; LHX9 | -1.09 | 3.15E-06 | -0.19 | 0.47 | 0.01 | 0.98 | 0.35 | 0.71 | -0.05 | 0.18 |
| cg26125384 | chr2:70314274 | PCBP1 | TSS1500 | PCBP1 | -2.02 | 3.19E-06 | -0.35 | 0.30 | -0.64 | 0.22 | 1.35 | 0.26 | -0.11 | 0.19 |
| cg00846140 | chr7:1892569 | MAD1L1 | Body | ELFN1; MAD1L1 | 2.29 | 3.25E-06 | 0.55 | 0.25 | 0.26 | 0.67 | 1.52 | 0.06 | 0.23 | 0.02 |
| cg05695876 | chr1:201665422 | NAV1 | Body | IPO9; NAV1 | -2.89 | 3.75E-06 | -1.17 | 0.32 | -0.67 | 0.52 | -1.13 | 0.61 | -0.16 | 0.19 |
| cg01664124 | chr1:42928162 | - | - | PPIH; PPCS | 2.77 | 3.86E-06 | 0.46 | 0.41 | 2.00 | 1.03E-03 | 2.11 | 0.05 | 0.20 | 0.10 |
| cg24919344 | chr5:24208918 | - | - | CDH10; PRDM9 | -3.85 | 4.27E-06 | -2.44 | 0.01 | 1.44 | 0.05 | -1.52 | 0.40 | -0.11 | 0.53 |
| cg19467738 | chr11:94823267 | ENDOD1 | 1stExon | ENDOD1 | 0.66 | 4.34E-06 | 0.06 | 0.68 | 0.14 | 0.21 | 0.06 | 0.86 | 0.07 | 1.11E-03 |
| ch.7.124218544F | chr7:124431308 | - | - | GPR37; POT1 | -3.11 | 4.35E-06 | -0.09 | 0.90 | -0.34 | 0.60 | -1.20 | 0.27 | -0.27 | 0.03 |
| cg14629665 | chr9:136819477 | VAV2 | Body | SARDH; VAV2 | -3.17 | 4.66E-06 | -0.59 | 0.47 | 0.26 | 0.81 | 1.52 | 0.48 | -0.29 | 0.03 |
| cg23461926 | chr5:147691991 | SPINK7 | 5'UTR; 1stExon | SPINK7 | 3.48 | 4.83E-06 | 0.30 | 0.60 | 0.35 | 0.60 | 2.86 | 0.05 | 0.28 | 0.02 |
| cg03919836 | chr16:58426331 | GINS3 | 5'UTR; 1stExon | GINS3 | 0.88 | 4.83E-06 | -0.13 | 0.44 | -0.42 | 0.04 | -0.49 | 0.31 | 0.05 | 0.14 |
| cg18710945 | chr14:24887496 | NYNRIN | 3'UTR | CBLN3; NYNRIN | 2.17 | 4.89E-06 | 0.56 | 0.25 | -0.27 | 0.59 | 1.30 | 0.28 | 0.20 | 0.02 |
| cg13169491 | chr7:128368940 | FAM71F1 | Body | CALU; METTL2B | -2.64 | 4.96E-06 | -0.76 | 0.04 | -0.13 | 0.65 | -2.67 | 0.02 | -0.19 | 0.05 |
| cg20975713 | chr15:102264772 | TARSL2 | TSS200 | TARSL2 | -0.99 | 5.13E-06 | 0.07 | 0.70 | 0.14 | 0.60 | -0.02 | 0.97 | -0.05 | 0.23 |
| cg18100830 | chr7:123903317 | - | - | TMEM229A; GPR37 | -5.07 | 5.27E-06 | -0.37 | 0.48 | 0.66 | 0.39 | -0.69 | 0.47 | -0.41 | 0.05 |
| cg25830605 | chr6:79758654 | PHIP | Body | PHIP; IRAK1BP1 | 2.32 | 5.29E-06 | -0.76 | 0.46 | -0.16 | 0.78 | 0.41 | 0.85 | 0.10 | 0.35 |
| cg19267163 | chr6:125004984 | NKAIN2 | Body | RNF217; NKAIN2 | -5.12 | 5.46E-06 | -1.39 | 0.12 | 0.23 | 0.79 | 2.61 | 0.13 | -0.39 | 0.05 |
| cg17753475 | chr2:180477963 | ZNF385B | Body | SESTD1; ZNF385B | 3.05 | 5.54E-06 | 0.63 | 0.15 | -0.63 | 0.49 | 1.27 | 0.40 | 0.06 | 0.65 |
| cg04609245 | chr4:41646293 | LIMCH1 | Body | PHOX2B; LIMCH1 | -2.99 | 5.91E-06 | -2.55 | 1.95E-03 | -0.91 | 0.26 | 2.09 | 0.10 | 0.03 | 0.82 |
| cg01758993 | chr20:44993436 | SLC35C2 | TSS1500 | SLC35C2 | -3.27 | 6.19E-06 | -0.90 | 0.03 | 0.13 | 0.82 | 1.54 | 0.29 | -0.27 | 0.04 |
| cg07393322 | chr22:43117318 | A4GALT | TSS1500 | A4GALT | -2.25 | 6.79E-06 | -0.67 | 0.22 | 0.17 | 0.81 | -0.93 | 0.47 | -0.11 | 0.32 |
| cg05388307 | chr4:166248715 | SC4MOL | TSS200 | MSMO1 | 0.51 | 6.89E-06 | -0.09 | 0.45 | 0.05 | 0.72 | -0.56 | 0.03 | 0.04 | 0.09 |
| cg00954536 | chr5:110427846 | WDR36 | TSS200 | WDR36 | -1.28 | 7.02E-06 | -0.23 | 0.39 | -0.07 | 0.88 | -0.72 | 0.37 | -0.08 | 0.10 |
| cg21349849 | chr8:99916884 | - | - | STK3; OSR2 | 2.96 | 7.41E-06 | 0.65 | 0.17 | -0.62 | 0.20 | 1.61 | 0.09 | 0.03 | 0.79 |
| cg04484842 | chr15:72410733 | MYO9A; SENP8 | TSS1500; 1stExon; 5'UTR | MYO9A; SENP8 | 0.56 | 7.87E-06 | 0.08 | 0.57 | -0.05 | 0.76 | -2.33E-03 | 0.99 | 0.02 | 0.32 |
| cg04362002 | chr5:23506738 | PRDM9 | TSS1500 | PRDM9 | 4.16 | 8.53E-06 | 1.42 | 0.10 | 1.56 | 0.06 | 2.22 | 0.19 | 0.24 | 0.16 |
| cg27316811 | chr16:1576146 | IFT140 | Body | TMEM204; TELO2 | 1.39 | 8.60E-06 | 0.56 | 0.04 | 0.54 | 0.12 | -1.10 | 0.12 | 0.13 | 0.02 |
| cg08722395 | chr2:144692640 | - | - | GTDC1; ARHGAP15 | 2.71 | 8.85E-06 | -0.87 | 0.27 | 1.36 | 0.04 | -0.20 | 0.86 | 0.27 | 0.03 |

**Supplementary Table 6: Bisulfite-pyrosequencing confirmed significant schizophrenia-associated hypomethylation across the *RPH3AL* DMR in both PFC and STR.**

| **CpG** | **450K probe** | **Genomic coordinates (hg19)** | **Prefrontal cortex** | | | | **Striatum** | | | |
| --- | --- | --- | --- | --- | --- | --- | --- | --- | --- | --- |
|  |  |  | **450K** | | **Pyrosequencing** | | **450K** | | **Pyrosequencing** | |
|  |  |  | **Methylation difference (%)** | ***P*** | **Methylation difference (%)** | ***P*** | **Methylation difference (%)** | ***P*** | **Methylation difference (%)** | ***P*** |
| 1 | - | chr17:154444 | - | - | -8.86 | 1.61E-03 | - | - | -5.09 | 1.82E-02 |
| 2 | - | chr17:154429 | - | - | -8.16 | 1.83E-03 | - | - | -5.10 | 1.50E-02 |
| 3 | cg11940040 | chr17:154420 | -7.76 | 4.36E-04 | -9.03 | 1.97E-03 | -5.04 | 7.89E-03 | -5.84 | 1.24E-02 |
| Average | - | - | - | - | -8.68 | 1.72E-03 | - | - | -5.33 | 1.41E-02 |

**Supplementary Table 7: Top ranked schizophrenia-associated differently methylated probes (DMPs) identified in the multiregion model incorporating the prefrontal cortex (PFC), striatum (STR) and hippocampus (HC) data.** Listed for each DMP are results from the multilevel model (grey) and corresponding results from the prefrontal cortex (PFC; *P* < 0.05 in blue), striatum (STR; *P* < 0.05 in green) and hippocampus (HC; *P* < 0.05 in red) meta-analyses (PFC and STR) or linear regression (HC). Also shown is the association with schizophrenia polygenic burden (multiregion model, *P*<0.05 in purple). Illumina and Genomic Regions Enrichment of Annotation Tool (GREAT) annotation[^1^](#_ENREF_1) is listed for each DMP.

| **Probe ID** | **Genomic position (hg19)** | **Illumina gene annotation** | **Gene region** | **GREAT annotation** [**^1^**](#_ENREF_1) | **Methylation difference (%) multilevel model** | ***P* multilevel model** | **Methylation difference PFC (%)** | ***P* PFC** | **Methylation difference STR (%)** | ***P* STR** | **Methylation difference HC (%)** | ***P* HC** | **Polygenic risk score methylation difference (%) multiregion model** | **Polygenic risk score *P* multiregion model** |
| --- | --- | --- | --- | --- | --- | --- | --- | --- | --- | --- | --- | --- | --- | --- |
| cg05966228 | chr16:67666442 | CTCF | Body | ACD; CTCF | -3.07 | 6.14E-07 | -1.73 | 0.02 | -1.99 | 6.13E-03 | -3.97 | 0.02 | -0.27 | 6.70E-03 |
| cg19091779 | chr12:13200089 | KIAA1467 | Body | KIAA1467; GSG1 | 1.90 | 6.63E-07 | 1.51 | 3.35E-03 | 1.49 | 1.77E-03 | 2.51 | 0.01 | 0.15 | 0.01 |
| cg08575268 | chr4:57411000 | - | - | ARL9; HOPX | -1.53 | 8.68E-07 | -0.69 | 0.03 | -1.62 | 1.37E-04 | -0.97 | 0.16 | -0.17 | 4.86E-04 |
| cg01837362 | chr12:34492938 | - | - | ALG10 | 6.12 | 9.91E-07 | 4.78 | 5.15E-04 | 5.26 | 2.56E-05 | 5.90 | 0.03 | 0.31 | 0.12 |
| cg01663682 | chr15:93447777 | CHD2 | Body | CHD2; RGMA | -0.96 | 2.01E-06 | -0.10 | 0.64 | -1.26 | 1.06E-06 | -1.54 | 0.05 | -9.72E-02 | 4.40E-03 |
| cg16669395 | chr16:10208417 | GRIN2A | Body | GRIN2A | -3.58 | 2.31E-06 | -2.79 | 2.94E-03 | -2.75 | 3.25E-03 | -2.22 | 0.27 | -0.19 | 0.12 |
| cg23749029 | chr7:148923218 | ZNF282 | 3'UTR | ZNF212; ZNF282 | -1.79 | 2.40E-06 | -1.34 | 7.80E-03 | -1.01 | 0.03 | -2.81 | 0.02 | -0.13 | 0.03 |
| cg07500432 | chr18:77918588 | PARD6G; LOC100130522 | Body | ADNP2; PARD6G | 6.09 | 3.03E-06 | 6.69 | 6.25E-05 | 4.64 | 1.54E-05 | 5.11 | 1.32E-03 | 0.64 | 2.01E-03 |
| cg00067720 | chr5:67521141 | PIK3R1 | TSS1500 | SLC30A5; PIK3R1 | -3.30 | 3.04E-06 | -1.24 | 0.07 | -2.42 | 2.53E-03 | -1.99 | 0.25 | -0.35 | 2.02E-03 |
| cg21299345 | chr4:1597692 | - | - | FAM53A; CRIPAK | 1.39 | 3.23E-06 | 1.13 | 3.56E-03 | 0.87 | 0.01 | 1.76 | 0.05 | 0.10 | 0.05 |
| cg13441156 | chr3:10335288 | GHRL | TSS1500 | GHRL | 2.98 | 4.05E-06 | 2.75 | 1.38E-03 | 2.24 | 0.01 | 2.47 | 0.13 | 0.19 | 0.08 |
| cg26228577 | chr2:121624862 | GLI2 | Body | GLI2; TFCP2L1 | -2.58 | 5.74E-06 | -0.82 | 0.20 | -2.33 | 2.36E-03 | -2.00 | 0.10 | -0.15 | 0.12 |
| cg01302436 | chr12:132865713 | GALNT9 | Body | GALNT9; NOC4L | -4.38 | 5.99E-06 | -2.32 | 0.01 | -0.48 | 0.65 | -5.61 | 0.03 | -0.47 | 1.65E-03 |
| cg07228402 | chr5:77917917 | LHFPL2 | 5'UTR | LHFPL2; SCAMP1 | 3.35 | 6.17E-06 | 2.94 | 2.90E-03 | 3.22 | 7.60E-04 | 2.77 | 0.23 | 0.14 | 0.27 |
| cg18806980 | chr16:84438123 | ATP2C2 | Body | ATP2C2; KIAA1609 | 2.03 | 6.61E-06 | 1.36 | 9.10E-03 | 1.94 | 6.38E-03 | 2.96 | 0.01 | 0.17 | 0.02 |
| cg16040341 | chr15:83544284 | HOMER2 | Body | WHAMM; HOMER2 | -2.04 | 8.23E-06 | -1.15 | 0.06 | -1.64 | 1.79E-04 | -1.51 | 0.15 | -0.22 | 1.56E-03 |
| cg16757441 | chr2:241419011 | ANKMY1 | 3'UTR | GPC1; ANKMY1 | 1.96 | 8.51E-06 | 1.42 | 0.02 | 1.32 | 0.03 | 1.55 | 0.09 | 0.18 | 0.01 |
| cg04230050 | chr6:166144874 | - | - | PDE10A; T | 1.69 | 8.66E-06 | 1.27 | 0.02 | 1.91 | 1.57E-04 | 0.93 | 0.27 | 0.06 | 0.38 |
| cg19353294 | chr6:164200967 | - | - | QKI | 1.97 | 8.66E-06 | 0.95 | 0.07 | 1.59 | 4.42E-03 | 4.22 | 8.92E-03 | 0.08 | 0.28 |
| cg23754665 | chr16:3313947 | - | - | ZNF263; MEFV | 1.68 | 8.76E-06 | 0.92 | 6.11E-03 | 1.80 | 9.37E-04 | 2.25 | 2.47E-03 | 0.06 | 0.32 |
| cg24538947 | chr2:26785301 | C2orf70 | TSS200 | OTOF | -2.33 | 9.13E-06 | -0.72 | 0.20 | -2.28 | 2.37E-03 | -2.69 | 0.05 | -0.32 | 7.76E-05 |
| cg11484348 | chr20:29896479 | DEFB116 | TSS200 | DEFB116 | 3.71 | 9.97E-06 | 2.71 | 1.13E-03 | 3.37 | 2.06E-04 | 5.41 | 0.02 | 0.25 | 0.06 |
| cg00254608 | chr12:124813321 | NCOR2 | Body | NCOR2; ZNF664 | -3.50 | 1.00E-05 | -1.75 | 0.05 | -3.02 | 3.92E-03 | -1.19 | 0.45 | -0.25 | 0.06 |
| cg00183888 | chr7:155276880 | - | - | RBM33; EN2 | -2.45 | 1.11E-05 | -1.42 | 0.06 | -1.40 | 0.05 | -1.94 | 0.19 | -0.28 | 2.86E-03 |
| cg10091053 | chr16:89603535 | SPG7 | Body; 3'UTR | RPL13; SPG7 | -2.89 | 1.13E-05 | -1.91 | 8.02E-03 | -1.56 | 0.03 | -3.88 | 0.07 | -0.27 | 0.01 |
| cg19988490 | chr6:30167065 | TRIM26 | 5'UTR | TRIM10; TRIM26 | 1.82 | 1.16E-05 | 1.19 | 0.04 | 1.72 | 2.16E-03 | 1.83 | 0.10 | 0.08 | 0.23 |
| cg14835517 | chr12:113917417 | - | - | LHX5; RBM19 | -1.52 | 1.24E-05 | -1.33 | 0.01 | -1.23 | 7.42E-03 | -1.28 | 0.09 | -0.14 | 7.37E-03 |
| cg22488717 | chr2:26785946 | C2orf70 | Body | OTOF | -1.83 | 1.25E-05 | -2.11 | 2.39E-05 | -0.57 | 0.30 | -1.40 | 0.31 | -0.11 | 0.11 |
| cg03608093 | chr11:691457 | DEAF1 | Body | TMEM80 | -1.94 | 1.29E-05 | -1.60 | 9.76E-03 | -1.28 | 0.04 | -1.90 | 0.07 | -6.42E-02 | 0.40 |
| cg19567740 | chr7:128527122 | KCP | Body | KCP; ATP6V1F | 1.93 | 1.31E-05 | 1.38 | 0.03 | 1.64 | 3.16E-03 | 2.78 | 9.08E-03 | 0.03 | 0.64 |
| cg27059530 | chr14:67707359 | MPP5 | TSS1500 | MPP5 | -0.80 | 1.31E-05 | -1.02 | 1.08E-05 | -0.55 | 0.04 | -1.02 | 0.08 | -5.21E-02 | 0.06 |
| cg24439505 | chr11:2172098 | INS-IGF2; IGF2 | Body; TSS1500 | IGF2; INS-IGF2 | -2.58 | 1.33E-05 | -2.39 | 2.14E-04 | -1.13 | 0.13 | -5.51 | 4.85E-03 | -0.15 | 0.14 |
| cg09941712 | chr15:81068966 | - | - | KIAA1199 | -1.37 | 1.41E-05 | -1.03 | 0.03 | -1.21 | 5.53E-04 | -1.17 | 0.11 | -7.84E-02 | 0.10 |
| cg20413415 | chr7:43351469 | HECW1 | Body | STK17A; HECW1 | 1.20 | 1.42E-05 | 1.12 | 7.51E-04 | 1.02 | 0.01 | 1.43 | 0.10 | 0.05 | 0.21 |
| cg03496533 | chr17:46035183 | PRR15L | TSS200 | CDK5RAP3; PNPO | 2.75 | 1.47E-05 | 1.68 | 0.01 | 1.67 | 0.08 | 2.33 | 0.15 | 0.07 | 0.49 |
| cg24652994 | chr7:122011261 | CADPS2 | Body | FEZF1; RNF133 | 1.33 | 1.50E-05 | 1.01 | 8.53E-03 | 0.61 | 0.09 | 1.95 | 0.11 | 0.06 | 0.22 |
| cg23092072 | chr4:87927706 | AFF1 | Body; TSS1500 | AFF1; HSD17B13 | -2.31 | 1.51E-05 | -0.56 | 0.03 | -2.30 | 8.19E-03 | -0.98 | 0.18 | -0.16 | 0.08 |
| cg11029358 | chr21:46875433 | COL18A1 | 5'UTR; 1stExon; Body | COL18A1 | -2.57 | 1.60E-05 | -1.65 | 0.03 | -2.19 | 9.00E-04 | -2.44 | 0.06 | -7.41E-02 | 0.44 |
| cg08775629 | chr2:107457756 | ST6GAL2 | Body | RGPD3; ST6GAL2 | -2.33 | 1.61E-05 | -1.45 | 0.06 | -1.64 | 0.02 | -2.78 | 0.09 | -2.59E-01 | 3.99E-03 |
| cg00369811 | chr6:37673597 | - | - | ZFAND3; MDGA1 | -1.25 | 1.72E-05 | -0.69 | 0.04 | -1.67 | 3.94E-05 | -0.53 | 0.58 | -3.72E-02 | 0.42 |
| cg11348994 | chr5:95769993 | PCSK1 | TSS1500 | PCSK1 | -2.48 | 1.73E-05 | -1.34 | 0.01 | -2.22 | 0.01 | -0.73 | 0.40 | -0.34 | 2.22E-04 |
| cg14843030 | chr1:32716051 | LCK | TSS1500 | LCK; EIF3I | 1.68 | 1.73E-05 | 1.09 | 0.04 | 1.62 | 4.97E-04 | 2.66 | 0.02 | 0.14 | 0.03 |
| cg14418226 | chr6:40996092 | UNC5CL | 3'UTR | LRFN2; UNC5CL | -1.98 | 1.74E-05 | -1.06 | 0.08 | -1.44 | 3.97E-03 | -1.78 | 0.17 | -1.72E-01 | 0.02 |
| cg18864882 | chr20:60371478 | CDH4 | Body | TAF4; CDH4 | 2.83 | 1.77E-05 | 2.18 | 0.02 | 2.68 | 4.38E-04 | 2.20 | 0.15 | 0.16 | 0.14 |
| cg25476129 | chr2:220341206 | SPEG | Body | GMPPA; SPEG | -2.22 | 1.80E-05 | -0.22 | 0.72 | -2.82 | 1.02E-04 | -2.80 | 0.01 | -0.26 | 1.19E-03 |
| cg04245131 | chr1:235133338 | - | - | IRF2BP2; TOMM20 | -2.37 | 1.90E-05 | -1.68 | 9.98E-03 | -1.24 | 0.08 | -1.54 | 0.14 | -0.27 | 2.47E-03 |
| cg00632364 | chr6:170401305 | - | - | DLL1; C6orf70 | -3.41 | 1.95E-05 | -1.46 | 0.14 | -2.80 | 3.95E-03 | -1.36 | 0.59 | -0.28 | 0.02 |
| cg21637761 | chr17:20107320 | CYTSB | Body | SPECC1; LGALS9B | -2.87 | 2.04E-05 | -2.17 | 7.54E-03 | -1.82 | 0.01 | -3.23 | 0.03 | -0.27 | 6.98E-03 |
| cg27597505 | chr12:54148217 | - | - | HOXC13; CALCOCO1 | -2.36 | 2.07E-05 | -2.44 | 1.30E-03 | -1.42 | 0.04 | -0.95 | 0.43 | -0.14 | 0.10 |
| cg19459454 | chr3:32509030 | - | - | CMTM6; CMTM7 | -3.00 | 2.10E-05 | -3.44 | 6.15E-05 | -1.20 | 0.13 | -2.65 | 0.20 | -0.25 | 0.03 |

**Supplementary Table 8: Significant schizophrenia-associated differently methylated regions (DMRs) identified in the multilevel model incorporating the prefrontal cortex, striatum and hippocampus data.** Shown in chromosomal order is the location of significant (Šidák-corrected *P* < 0.05) DMRs identified in the multilevel model. The column “Gene” represents the combined Illumina and Genomic Regions Enrichment of Annotation Tool (GREAT) annotation [^1^](#_ENREF_1).

| **Region** | **Gene** | **Probes** | **N probes** | **Median *P*** | **Šidák *P*** |
| --- | --- | --- | --- | --- | --- |
| chr1:89664260-89664546 | GBP4 | cg23978657; cg02482460; cg22221320; cg21365602; cg20410995 | 5 | 3.67E-03 | 0.01 |
| chr3:32509018-32509332 | CMTM6; CMTM7 | cg00718400; cg19459454; cg13050802; cg05625284; cg16621749; cg05651657 | 6 | 4.36E-03 | 3.32E-03 |
| chr3:49170496-49170850 | LAMB2 | cg01919208; cg02954987; cg08234664; cg05654765; cg14099457; cg11566975 | 6 | 9.57E-03 | 8.60E-03 |
| chr3:55517496-55518442 | WNT5A; LRTM1 | cg20746482; cg08852968; cg27364162; cg18562578; cg18010752; cg17679453; cg04941246; cg24216596; cg08666668; cg02867696; cg23456221 | 11 | 1.06E-03 | 8.90E-11 |
| chr5:23507450-23507657 | PRDM9 | cg22054885; cg19837938; cg02444433; cg25472530; cg22079902; cg01667892 | 6 | 7.27E-03 | 0.04 |
| chr6:30043049-30043419 | RNF39 | cg23500724; cg10865856; cg24016627; cg23939808; cg12967914; cg00853042; cg23027574; cg05853632; cg22105332; cg19006429; cg27532187; cg01631162 | 12 | 1.36E-02 | 5.82E-04 |
| chr6:31648650-31649094 | LY6G5C | cg08226747; cg18581937; cg19387310; cg25188387; cg07845406; cg17990278; cg03588325; cg23642766; cg06667222; cg14102811; cg14463529; cg07151644; cg09295695 | 13 | 2.37E-02 | 3.38E-03 |
| chr6:33739406-33739654 | LEMD2; IP6K3 | cg07979401; cg18005901; cg16010596; cg13859433 | 4 | 1.43E-03 | 0.04 |
| chr6:40995889-40996214 | UNC5CL; LRFN2 | cg21774121; cg24419528; cg00592058; cg14418226; cg21128951 | 5 | 2.03E-03 | 2.87E-05 |
| chr10:63809073-63809171 | ARID5B; RTKN2 | cg14789659; cg00928816; cg20746552; cg16389209; cg07520810; cg16401465 | 6 | 3.78E-03 | 4.51E-03 |
| chr10:124638756-124639013 | FAM24B; LOC399815 | cg03804621; cg16299003; cg11218091; cg14708218; cg18195080 | 5 | 1.39E-03 | 4.44E-04 |
| chr10:134994186-134994456 | KNDC1; UTF1 | cg13609319; cg18398637; cg06517181; cg08815970; cg09274040 | 5 | 2.72E-04 | 4.10E-03 |
| chr11:6341717-6341909 | PRKCDBP | cg16459349; cg20938665; cg02273041; cg27132391; cg15202102; cg26678920; cg05628549; cg10064871 | 8 | 1.12E-02 | 0.03 |
| chr12:124864528-124864682 | NCOR2;ZNF664 | cg04930596; cg07241090; cg17387577; cg17825194 | 4 | 1.79E-04 | 2.64E-04 |
| chr14:36983129-36983695 | SFTA3; MBIP; NKX2-1 | cg23542968; cg10385303; cg16478719; cg22945387; cg27294268; cg05372242; cg04330513 | 5 | 1.04E-02 | 6.79E-04 |
| chr16:67184918-67185195 | B3GNT9 | cg08659394; cg05527491; cg06212637; cg20984188; cg02771381; cg02724047 | 6 | 5.84E-03 | 9.98E-03 |
| chr16:89603535-89603818 | SPG7; RPL13 | cg10091053; cg02207944; cg03740221; cg00855299 | 4 | 4.37E-03 | 5.41E-03 |
| chr17:154410-154672 | RPH3AL; DOC2B | cg08770870; cg11940040; cg10440639; cg23246911 | 4 | 1.77E-04 | 1.23E-05 |
| chr17:76220608-76220956 | BIRC5; EPR1; TMEM235 | cg11912239; cg10140240; cg00017271; cg19272238; cg07366188; cg10070788 | 6 | 6.10E-03 | 1.42E-03 |
| chr19:57149436-57149631 | ZNF835; ZNF71 | cg07962143; cg15091407; cg02940165; cg14627089 | 4 | 3.06E-03 | 0.03 |
| chr20:37230326-37230613 | C20orf95; ARHGAP40 | cg01025836; cg00557360; cg04608177; cg03356734; cg06301550; cg08438366 | 6 | 5.14E-03 | 0.03 |
| chr21:46875142-46875434 | COL18A1 | cg02124724; cg16121744; cg14903689; cg07279557; cg11029358 | 5 | 8.81E-03 | 5.86E-03 |

**Supplementary Table 9: Results for top-ranked schizophrenia-associated probes identified in a previous study of cortical tissue**[**^2^**](#_ENREF_2)**.** Shown are data for each brain region for probes previously associated with schizophrenia (FDR < 0.1) in the cortex by Pidsley et al (2014)[^2^](#_ENREF_2).

| Probe | Gene | Pidsley et al. Methylation difference (%) | Pidsley et al. *P* | PFC methylation difference (%) | PFC *P* | STR methylation difference (%) | STR *P* | HC methylation difference (%) | HC *P* | CER methylation difference (%) | CER *P* | Multilevel model methylation difference (%) | Multilevel model *P* |
| --- | --- | --- | --- | --- | --- | --- | --- | --- | --- | --- | --- | --- | --- |
| cg26173173 | GSDMD | 4 | 1.16E-07 | 3.11 | 8.06E-07 | 1.39 | 7.59E-02 | 1.73 | 2.99E-01 | 1.21 | 1.81E-01 | 2.47 | 4.28E-05 |
| cg24803255 | RASA3 | -9 | 1.25E-07 | -3.23 | 8.98E-05 | -0.99 | 5.26E-02 | 0.58 | 7.98E-01 | -1.35 | 3.03E-01 | -2.61 | 1.51E-04 |
| cg00903099 | HTR5A | -2 | 2.40E-07 | -1.25 | 2.42E-04 | -0.09 | 8.79E-01 | -0.07 | 9.62E-01 | 0.06 | 8.75E-01 | -0.67 | 1.36E-01 |
| cg08171022 | PPFIA1 | -5 | 2.85E-07 | -2.49 | 6.26E-04 | 0.14 | 8.76E-01 | -1.03 | 5.84E-01 | -1.01 | 1.38E-01 | -1.82 | 1.19E-02 |
| cg02857643 | CACNA1G | -5 | 1.63E-06 | -0.04 | 9.44E-01 | -0.65 | 6.40E-02 | 0.06 | 8.60E-01 | 0.25 | 3.17E-01 | -0.82 | 2.09E-02 |
| cg00236305 | MYT1L | -6 | 1.75E-06 | -2.40 | 3.68E-04 | 0.04 | 9.13E-01 | -0.82 | 4.53E-01 | -2.66 | 1.12E-01 | -1.56 | 4.62E-04 |
| cg14966346 | KLC1 | -5 | 2.51E-06 | -1.63 | 1.46E-02 | 0.06 | 8.93E-01 | -0.80 | 4.92E-01 | -0.38 | 7.05E-01 | -1.22 | 3.53E-03 |
| cg13079528 | SDK1 | -8 | 2.75E-06 | -2.24 | 1.44E-02 | 0.08 | 9.18E-01 | -2.02 | 2.35E-01 | 0.32 | 1.74E-01 | -1.28 | 3.52E-02 |
| cg14429765 | MCPH1 | 3 | 2.79E-06 | - | - | - | - | - | - | - | - | - | - |
| cg08602214 | RHOBTB2 | -7 | 2.87E-06 | -1.93 | 2.41E-03 | -0.28 | 6.41E-01 | -1.82 | 1.61E-01 | -0.27 | 4.95E-01 | -1.08 | 3.45E-02 |
| cg19735533 | - | -6 | 2.94E-06 | -1.45 | 6.34E-02 | 0.51 | 1.54E-01 | -0.50 | 6.58E-01 | 0.13 | 8.21E-01 | -0.44 | 3.52E-01 |
| cg09507608 | - | -6 | 2.95E-06 | -2.64 | 1.85E-03 | -0.71 | 4.26E-01 | -1.39 | 5.89E-01 | -0.54 | 4.82E-01 | -1.52 | 3.78E-02 |
| cg23844013 | C8A | 3 | 3.20E-06 | 1.82 | 1.04E-04 | -0.28 | 5.79E-01 | -0.41 | 7.84E-01 | 0.70 | 3.39E-01 | 0.44 | 3.28E-01 |
| cg26578910 | PRKD2 | -4 | 3.47E-06 | -2.01 | 7.61E-05 | -0.35 | 7.04E-01 | -0.03 | 9.81E-01 | 0.40 | 2.91E-01 | -0.02 | 9.81E-01 |
| cg21847368 | MCF2L | -3 | 3.70E-06 | -1.29 | 8.90E-03 | 0.60 | 2.71E-01 | 1.71 | 1.05E-01 | 1.42 | 8.20E-02 | -0.55 | 1.72E-01 |
| cg03607729 | LCMT1 | -4 | 4.19E-06 | -0.44 | 3.49E-01 | -0.25 | 5.66E-01 | -0.77 | 4.06E-01 | 0.57 | 1.97E-01 | -0.90 | 2.53E-02 |
| cg10248981 | LHPP | -7 | 4.32E-06 | -2.85 | 7.43E-04 | 0.43 | 7.63E-01 | -4.24 | 1.43E-01 | -0.36 | 8.47E-01 | -2.12 | 4.93E-02 |
| cg03445663 | HDLBP | -8 | 4.59E-06 | -2.50 | 1.67E-03 | 0.13 | 5.28E-01 | -0.76 | 6.24E-01 | -0.93 | 2.54E-01 | -1.92 | 1.30E-03 |
| cg15079231 | VIPR1 | -4 | 4.93E-06 | -2.08 | 5.59E-04 | 0.18 | 7.74E-01 | -0.29 | 8.43E-01 | 0.32 | 6.27E-01 | -0.91 | 6.35E-02 |
| cg21341878 | ZFYVE28 | -5 | 5.14E-06 | -1.63 | 1.47E-02 | 0.18 | 6.06E-01 | -0.59 | 5.55E-01 | -1.24 | 4.28E-01 | -1.10 | 9.27E-03 |
| cg04922803 | GLT8D2 | 6 | 5.19E-06 | 2.75 | 1.11E-02 | -0.36 | 8.09E-01 | 1.70 | 4.73E-01 | 1.01 | 5.24E-01 | 0.24 | 8.46E-01 |
| cg18857062 | CRIP3 | -4 | 5.29E-06 | -2.63 | 9.65E-05 | 1.25 | 1.97E-01 | -0.90 | 6.35E-01 | -0.70 | 1.51E-01 | -1.23 | 1.04E-01 |

**Supplementary Table 10: Schizophrenia-associated differently methylated probes (DMPs) located within robustly-associated regions identified in a recent large GWAS analysis**[**^3^**](#_ENREF_3)**.** Listed are all DMPs significant at *P* < 1.00E-3 within the 108 genomic regions associated with schizophrenia in the recent GWAS analysis.

| **Probe** | **Brain region** | **Methylation difference (%)** | ***P*** | ***P* Rank** | **Gene** | **GWAS region** | **Best SNP** | **Min *P*** |
| --- | --- | --- | --- | --- | --- | --- | --- | --- |
| cg20098710 | Prefrontal cortex | -2.88 | 5.61E-06 | 15 | YJEFN3 | chr19:19374022-19658022 | rs2905426 | 3.63E-10 |
| cg11794120 | Striatum | -1.43 | 2.03E-05 | 38 | MAD1L1 | chr7:1896096-2190096 | chr7_2025096_I | 8.20E-15 |
| cg17170741 | Cerebellum | -0.93 | 3.22E-05 | 112 | ATPAF2; C17orf39 | chr17:17722402-18030202 | rs8082590 | 1.77E-08 |
| cg19899008 | Striatum | -3.24 | 5.69E-05 | 74 | SLC12A4 | chr16:67709340-68311340 | rs8044995 | 1.51E-08 |
| cg17436506 | Multilevel model | 0.82 | 1.12E-04 | 243 | ARL6IP4; OGFOD2 | chr12:123448113-123909113 | rs2851447 | 1.86E-14 |
| cg06002516 | Multilevel model | -2.37 | 1.50E-04 | 324 | R3HDM2 | chr12:57428314-57682971 | rs12826178 | 2.02E-12 |
| cg24533149 | Multilevel model | -0.81 | 1.77E-04 | 389 | MOBKL3 | chr2:198148577-198835577 | rs6434928 | 2.06E-11 |
| cg22376739 | Cerebellum | -1.23 | 2.00E-04 | 488 | TOM1L2; LRRC48 | chr17:17722402-18030202 | rs8082590 | 1.77E-08 |
| cg04113450 | Multilevel model | 2.04 | 2.16E-04 | 455 | SCAND3 | chr6:28303247-28712247 | rs115329265 | 3.48E-31 |
| cg10881128 | Cerebellum | -1.23 | 2.17E-04 | 519 | PTPRF | chr1:44029384-44128084 | rs11210892 | 3.39E-10 |
| cg00657460 | Multilevel model | -1.96 | 2.65E-04 | 536 | TCF4 | chr18:52747686-53200117 | rs9636107 | 3.34E-12 |
| cg04502620 | Multilevel model | -2.08 | 2.73E-04 | 549 | DOC2A | chr16:29924377-30144877 | rs12691307 | 4.55E-11 |
| cg01687878 | Cerebellum | 3.00 | 2.84E-04 | 643 | - | chr12:123448113-123909113 | rs2851447 | 1.86E-14 |
| cg27007524 | Cerebellum | -3.20 | 3.26E-04 | 728 | - | chr6:28303247-28712247 | rs115329265 | 3.48E-31 |
| cg25966682 | Cerebellum | 2.06 | 3.65E-04 | 788 | LRP1 | chr12:57428314-57682971 | rs12826178 | 2.02E-12 |
| cg03498304 | Cerebellum | 0.51 | 3.88E-04 | 828 | CENPT; THAP11 | chr16:67709340-68311340 | rs8044995 | 1.51E-08 |
| cg13247935 | Multilevel model | -2.47 | 4.41E-04 | 824 | KLC1 | chr14:103996234-104184834 | rs12887734 | 1.36E-13 |
| cg15471197 | Cerebellum | 2.55 | 5.13E-04 | 1013 | LRP1 | chr12:57428314-57682971 | rs12826178 | 2.02E-12 |
| cg01385430 | Cerebellum | 0.51 | 5.37E-04 | 1055 | VPS45 | chr1:149998890-150242490 | rs140505938 | 4.49E-10 |
| cg10124201 | Cerebellum | 2.64 | 5.63E-04 | 1090 | BOLL | chr2:198148577-198835577 | rs6434928 | 2.06E-11 |
| cg11244672 | Prefrontal cortex | -2.73 | 5.95E-04 | 664 | YJEFN3 | chr19:19374022-19658022 | rs2905426 | 3.63E-10 |
| cg08365687 | Striatum | 1.95 | 6.08E-04 | 430 | NT5DC2; LOC440957 | chr3:52541105-52903405 | rs2535627 | 4.26E-11 |
| cg03885098 | Striatum | -1.79 | 6.27E-04 | 442 | LOC148696 | chr1:207912183-208024083 | rs7523273 | 4.47E-08 |
| cg00022076 | Multilevel model | 1.11 | 6.44E-04 | 1120 | PLA2G15 | chr16:67709340-68311340 | rs8044995 | 1.51E-08 |
| cg01805920 | Cerebellum | -0.44 | 6.64E-04 | 1235 | HIRIP3; INO80E | chr16:29924377-30144877 | rs12691307 | 4.55E-11 |
| cg18711734 | Cerebellum | 2.17 | 6.72E-04 | 1243 | - | chr5:137598121-137948092 | rs3849046 | 4.67E-09 |
| cg12758687 | Prefrontal cortex | -1.63 | 6.97E-04 | 777 | DRD2 | chr11:113317794-113423994 | rs2514218 | 2.75E-11 |
| cg17815546 | Multilevel model | 0.91 | 7.01E-04 | 1207 | MAD1L1 | chr7:1896096-2190096 | chr7_2025096_I | 8.20E-15 |
| cg19076659 | Cerebellum | 2.83 | 7.06E-04 | 1285 | KDM3B | chr5:137598121-137948092 | rs3849046 | 4.67E-09 |
| cg01230250 | Cerebellum | 0.99 | 7.08E-04 | 1288 | MDK | chr11:46342943-46751213 | chr11_46350213_D | 1.26E-11 |
| cg19911185 | Cerebellum | 3.03 | 7.19E-04 | 1306 | FAM53C | chr5:137598121-137948092 | rs3849046 | 4.67E-09 |
| cg20091215 | Prefrontal cortex | 1.63 | 7.27E-04 | 803 | DGKZ | chr11:46342943-46751213 | chr11_46350213_D | 1.26E-11 |
| cg08020660 | Prefrontal cortex | 0.29 | 7.27E-04 | 805 | ALDOA | chr16:29924377-30144877 | rs12691307 | 4.55E-11 |
| cg08450501 | Cerebellum | 0.81 | 7.27E-04 | 1317 | NCK1 | chr3:135807405-136615405 | rs7432375 | 7.26E-11 |
| cg09276072 | Multilevel model | 1.25 | 7.32E-04 | 1244 | TSNAXIP1 | chr16:67709340-68311340 | rs8044995 | 1.51E-08 |
| cg20803211 | Prefrontal cortex | 2.37 | 7.61E-04 | 835 | ZSCAN23 | chr6:28303247-28712247 | rs115329265 | 3.48E-31 |
| cg13494126 | Cerebellum | 0.36 | 7.72E-04 | 1382 | MAD1L1 | chr7:1896096-2190096 | chr7_2025096_I | 8.20E-15 |
| cg02969798 | Cerebellum | 2.29 | 7.80E-04 | 1395 | ARL6IP4 | chr12:123448113-123909113 | rs2851447 | 1.86E-14 |
| cg05566397 | Prefrontal cortex | -0.59 | 8.18E-04 | 888 | MLL5; LOC100216545 | chr7:104598064-105063064 | rs6466055 | 1.13E-09 |
| cg04676715 | Cerebellum | -0.79 | 8.30E-04 | 1453 | TSNAXIP1; RANBP10 | chr16:67709340-68311340 | rs8044995 | 1.51E-08 |
| cg14651446 | Cerebellum | -0.73 | 8.81E-04 | 1536 | - | chr6:28303247-28712247 | rs115329265 | 3.48E-31 |
| cg27305769 | Hippocampus | 2.01 | 8.85E-04 | 261 | MAD1L1 | chr7:1896096-2190096 | chr7_2025096_I | 8.20E-15 |
| cg19643441 | Prefrontal cortex | -1.56 | 8.96E-04 | 958 | SF4 | chr19:19374022-19658022 | rs2905426 | 3.63E-10 |
| cg03240800 | Multilevel model | 2.80 | 9.55E-04 | 1576 | PCDHA6; PCDHA2; PCDHA1; PCDHA7; PCDHA8; PCDHA5; PCDHA3; PCDHA4 | chr5:140023664-140222664 | chr5_140143664_I | 4.85E-08 |
| cg17861150 | Hippocampus | -1.92 | 9.84E-04 | 291 | RERE | chr1:8411184-8638984 | chr1_8424984_D | 1.17E-09 |

**Supplementary Table 11: Top ranked polygenic risk score-associated DMPs identified in the prefrontal cortex (PFC) meta-analysis.** Listed for each PFC DMP (grey) are corresponding results from the striatum (STR; *P* < 0.05 in green), hippocampus (HC; *P* < 0.05 in red) and cerebellum (CER; *P* < 0.05 in orange) meta-analyses (PFC, STR and CER) or linear regression (HC). Also shown is the association with schizophrenia diagnosis in PFC (*P* < 0.05 in blue). The methylation difference is measured per PRS unit. Illumina and Genomic Regions Enrichment of Annotation Tool (GREAT) annotation[^1^](#_ENREF_1) is listed for each DMP.

| **Probe ID** | **Genomic position (hg19)** | **Illumina gene annotation** | **Gene region** | **GREAT annotation** [**^1^**](#_ENREF_1) | **Methylation difference (%) PFC** | ***P* PFC** | **Methylation difference STR (%)** | ***P* STR** | **Methylation difference HC (%)** | ***P* HC** | **Methylation difference CER (%)** | ***P* CER** | **Disease PFC methylation difference (%)** | **Disease PFC *P*** |
| --- | --- | --- | --- | --- | --- | --- | --- | --- | --- | --- | --- | --- | --- | --- |
| cg18847009 | chr2:70175826 | - | - | ASPRV1; MXD1 | -0.51 | 8.98E-08 | -0.32 | 0.02 | -0.79 | 1.07E-03 | -0.20 | 0.22 | -1.12 | 0.11 |
| cg14595786 | chr19:51626986 | SIGLEC9 | TSS1500 | SIGLEC9 | 0.54 | 2.45E-07 | 0.12 | 0.44 | 0.63 | 0.04 | 0.17 | 0.20 | 2.72 | 1.96E-03 |
| cg05090695 | chr11:2907670 | CDKN1C | TSS1500 | CDKN1C | -0.53 | 2.65E-07 | 0.16 | 0.21 | -0.30 | 0.06 | -0.05 | 0.12 | -0.56 | 0.43 |
| cg01948217 | chr20:36932385 | BPI | TSS200 | BPI | 0.66 | 7.02E-07 | 0.24 | 0.20 | 0.00 | 0.99 | -0.14 | 0.34 | -0.03 | 0.97 |
| cg01986619 | chr7:2613963 | IQCE | Body | TTYH3; IQCE | 0.48 | 8.15E-07 | -0.11 | 0.41 | 0.37 | 0.04 | -0.13 | 0.30 | 1.04 | 0.13 |
| cg20678082 | chr1:168356591 | - | - | TBX19; XCL2 | -0.66 | 1.23E-06 | -0.08 | 0.65 | -0.32 | 0.27 | -0.21 | 0.34 | -0.91 | 0.34 |
| cg17460228 | chr15:41052250 | - | - | FAM82A2; GCHFR | 0.38 | 1.42E-06 | 0.28 | 0.11 | 0.60 | 0.02 | -0.05 | 0.68 | 0.93 | 0.11 |
| cg04221388 | chr5:167689716 | ODZ2 | Body | WWC1; ODZ2 | 0.23 | 2.28E-06 | 0.02 | 0.70 | -0.13 | 0.33 | -0.05 | 0.58 | 0.58 | 0.16 |
| cg26283550 | chr11:62623419 | SNORD27; SLC3A2; SNORD28; SNORD25; SNORD26; SNHG1 | TSS1500; TSS200 | SLC3A2 | -0.08 | 2.96E-06 | 0.00 | 0.97 | 0.03 | 0.48 | -0.02 | 0.50 | -0.07 | 0.59 |
| cg03000593 | chr6:33283162 | ZBTB22; TAPBP | Body; TSS1500 | TAPBP | -0.58 | 4.06E-06 | 0.02 | 0.84 | 0.19 | 0.37 | -0.04 | 0.80 | -0.28 | 0.72 |
| cg17926234 | chr2:61404587 | AHSA2 | 1stExon; 5'UTR | AHSA2 | 0.10 | 4.41E-06 | -0.02 | 0.83 | 0.00 | 0.96 | 0.00 | 0.96 | 0.10 | 0.57 |
| cg23822732 | chr2:2692854 | - | - | TRAPPC12; MYT1L | 0.23 | 5.53E-06 | -0.09 | 0.12 | 0.16 | 0.14 | -0.04 | 0.56 | 0.82 | 0.03 |
| cg04293307 | chr17:63553581 | AXIN2 | Body | AXIN2; RGS9 | 0.54 | 6.07E-06 | 0.02 | 0.91 | 0.44 | 0.15 | 0.41 | 0.03 | 0.49 | 0.63 |
| cg25489169 | chr17:46689639 | HOXB7 | TSS1500 | HOXB7 | -0.17 | 7.07E-06 | 0.11 | 0.10 | -0.04 | 0.73 | -0.06 | 0.26 | -0.48 | 0.06 |
| cg12463346 | chr4:102268854 | PPP3CA | TSS1500 | PPP3CA | 0.08 | 7.72E-06 | 0.02 | 0.43 | 0.09 | 0.21 | 0.03 | 0.22 | 0.11 | 0.31 |
| cg04862340 | chr16:6534204 | A2BP1 | 5'UTR | RBFOX1 | 0.86 | 8.04E-06 | - | - | - | - | - | - | 1.23 | 0.32 |
| cg27454589 | chr15:71509548 | THSD4 | Body | NR2E3; THSD4 | 0.28 | 9.12E-06 | 0.06 | 0.63 | 0.06 | 0.80 | -0.03 | 0.77 | 0.58 | 0.26 |
| cg02489245 | chr15:53495286 | - | - | UNC13C; ONECUT1 | 0.29 | 1.15E-05 | 0.08 | 0.35 | 0.11 | 0.41 | 0.02 | 0.88 | 1.03 | 0.02 |
| cg05987933 | chr5:176513799 | FGFR4 | TSS200 | FGFR4 | -0.17 | 1.15E-05 | -0.13 | 0.22 | -0.33 | 0.08 | -0.13 | 0.11 | -0.25 | 0.35 |
| cg13548543 | chr9:34460044 | DNAI1; C9orf25 | Body; TSS1500 | DNAI1; ENHO | 0.40 | 1.30E-05 | 0.06 | 0.70 | 0.32 | 0.32 | -0.10 | 0.42 | 0.25 | 0.72 |
| cg08506743 | chr11:131779750 | NTM | TSS1500; Body | NTM | -0.32 | 1.39E-05 | 0.07 | 0.40 | -0.01 | 0.96 | 0.20 | 0.09 | -0.72 | 0.15 |
| cg16037569 | chr1:9710867 | PIK3CD | TSS1500 | PIK3CD | 0.48 | 1.40E-05 | 0.29 | 0.14 | 0.14 | 0.77 | 0.05 | 0.76 | 0.42 | 0.58 |
| cg26894839 | chr5:1511271 | LPCAT1 | Body | SLC6A3; LPCAT1 | 0.26 | 1.45E-05 | -0.08 | 0.35 | 0.13 | 0.29 | 0.20 | 0.04 | 1.35 | 1.18E-03 |
| cg24202468 | chr13:99195339 | STK24 | Body | RNF113B; STK24 | 0.57 | 1.45E-05 | 0.21 | 0.20 | 0.54 | 0.02 | 0.07 | 0.64 | 0.91 | 0.26 |
| cg09055236 | chr7:2673197 | TTYH3 | Body | AMZ1; TTYH3 | 0.27 | 1.46E-05 | -0.05 | 0.58 | 0.24 | 0.14 | -0.06 | 0.24 | 0.86 | 0.04 |
| cg22930808 | chr3:122281881 | PARP9; DTX3L | 5'UTR; TSS1500 | DTX3L | 0.56 | 1.47E-05 | 0.39 | 9.87E-04 | 0.04 | 0.82 | 0.18 | 0.06 | 0.71 | 0.37 |
| cg17552088 | chr6:38087605 | ZFAND3 | Body | ZFAND3; BTBD9 | 0.68 | 1.59E-05 | 0.43 | 0.01 | -0.10 | 0.83 | 0.30 | 0.11 | -0.06 | 0.95 |
| cg10119001 | chr1:153114766 | SPRR2C | TSS1500 | SPRR2F; SPRR2G | 0.44 | 1.73E-05 | 0.08 | 0.64 | 0.58 | 0.14 | 0.32 | 0.03 | 1.10 | 0.11 |
| cg23630131 | chr7:65973040 | - | - | KCTD7; TPST1 | 0.64 | 1.89E-05 | 0.14 | 0.54 | 0.09 | 0.71 | 0.13 | 0.61 | 1.36 | 0.19 |
| cg15928016 | chr10:94064371 | MAR05 | Body | MARCH5; IDE | 0.58 | 2.01E-05 | -0.07 | 0.58 | 0.45 | 0.22 | 0.40 | 3.78E-04 | 1.48 | 0.08 |
| cg00735454 | chr13:44595496 | LOC121838 | TSS1500 | ENOX1; SERP2 | 0.83 | 2.06E-05 | 0.40 | 0.01 | 0.26 | 0.31 | 0.22 | 0.15 | 0.83 | 0.47 |
| cg23696248 | chr19:45260501 | BCL3 | Body | CBLC; BCL3 | -0.27 | 2.08E-05 | 0.14 | 0.19 | -0.32 | 0.18 | -0.28 | 0.04 | -0.35 | 0.39 |
| cg19851574 | chr6:167178233 | RPS6KA2 | Body | BRP44L; RPS6KA2 | 0.23 | 2.10E-05 | 0.03 | 0.52 | -0.20 | 0.17 | 0.12 | 0.26 | -0.15 | 0.66 |
| cg06951750 | chr16:614645 | C16orf11 | Body | PIGQ; SOLH | 0.61 | 2.31E-05 | 0.24 | 0.09 | 0.43 | 0.03 | 0.12 | 0.20 | 1.97 | 0.01 |
| cg07410044 | chr2:71222186 | TEX261 | TSS200 | TEX261 | 0.14 | 2.42E-05 | 0.00 | 0.98 | 0.06 | 0.47 | 0.05 | 0.30 | 0.56 | 0.01 |
| cg21136371 | chr11:1991065 | - | - | MRPL23; IGF2 | -0.35 | 2.49E-05 | -0.24 | 0.02 | -0.05 | 0.83 | -0.05 | 0.75 | -0.82 | 0.17 |
| cg06105987 | chr7:151483394 | PRKAG2 | Body | RHEB; PRKAG2 | -0.05 | 2.77E-05 | 0.01 | 0.54 | -0.03 | 0.29 | 0.00 | 0.95 | -0.05 | 0.59 |
| cg24368167 | chr3:38179936 | ACAA1; MYD88 | TSS1500; TSS200 | ACAA1; MYD88 | -0.27 | 2.97E-05 | -0.06 | 0.32 | -0.22 | 0.02 | -0.12 | 0.06 | -0.32 | 0.42 |
| cg15353031 | chr10:50887632 | C10orf53 | TSS200 | CHAT; OGDHL | -0.14 | 3.12E-05 | -0.07 | 0.12 | 0.05 | 0.52 | 0.07 | 0.02 | -0.11 | 0.61 |
| cg19852211 | chr5:140187240 | PCDHA2; PCDHA1; PCDHA4; PCDHA3 | Body; 1stExon | PCDHAC2; ZMAT2 | 0.33 | 3.25E-05 | -0.14 | 0.04 | 0.12 | 0.29 | 0.04 | 0.64 | 0.54 | 0.24 |
| cg10998122 | chr11:64008466 | FKBP2 | TSS1500; 1stExon; 5'UTR; TSS200 | FKBP2 | 0.11 | 3.65E-05 | 0.05 | 0.60 | 0.04 | 0.63 | 0.02 | 0.61 | -0.09 | 0.63 |
| cg01868896 | chr15:52581521 | MYO5C | Body | GNB5; MYO5C | 0.43 | 3.89E-05 | -0.02 | 0.90 | 0.24 | 0.53 | 0.22 | 0.16 | 1.75 | 9.40E-03 |
| cg08616269 | chr10:70480892 | CCAR1 | TSS200 | CCAR1 | 0.19 | 4.18E-05 | -0.04 | 0.43 | -0.02 | 0.76 | 0.05 | 0.34 | 0.46 | 0.14 |
| cg19526685 | chr8:126963507 | - | - | TRIB1; FAM84B | 0.47 | 4.30E-05 | -0.01 | 0.94 | -0.11 | 0.63 | -0.23 | 0.05 | -0.01 | 0.99 |
| cg11832804 | chr5:1279449 | TERT | Body | TERT; SLC6A18 | 0.15 | 4.35E-05 | 0.05 | 0.20 | -0.03 | 0.51 | 0.03 | 0.41 | 0.37 | 0.06 |
| cg25730577 | chr1:110453002 | CSF1 | TSS1500 | CSF1 | -0.25 | 4.51E-05 | -0.08 | 0.37 | -0.35 | 2.09E-03 | -0.08 | 0.22 | -0.55 | 0.15 |
| cg04245568 | chr16:88453579 | - | - | ZNF469; BANP | 0.11 | 5.88E-05 | -0.01 | 0.68 | -0.08 | 0.15 | 0.04 | 0.15 | 0.80 | 0.35 |
| cg04410989 | chr20:35578437 | SAMHD1 | N_Shore | DSN1; SAMHD1 | 0.29 | 6.34E-05 | 0.20 | 0.07 | 0.15 | 0.45 | 0.10 | 0.20 | -0.45 | 0.36 |
| cg00379630 | chr6:159736614 | - | - | FNDC1; SOD2 | 0.50 | 7.87E-05 | -0.14 | 0.14 | -0.04 | 0.83 | 0.01 | 0.95 | -1.04 | 0.14 |
| cg04021074 | chr10:14816947 | FAM107B | TSS200 | FRMD4A; CDNF | 0.30 | 8.08E-05 | 0.18 | 0.02 | 0.13 | 0.52 | 0.09 | 0.48 | -0.08 | 0.46 |

**Supplementary Table 12: Top ranked polygenic risk score-associated differently methylated probes (DMPs) identified in the striatum (STR) meta-analysis.** Listed for each STR DMP (grey) are corresponding results from the prefrontal cortex (PFC; *P* < 0.05 in blue), hippocampus (HC; *P* < 0.05 in red) and cerebellum (CER; *P* < 0.05 in orange) meta-analyses (PFC, STR and CER) or linear regression (HC). Also shown is the association with schizophrenia diagnosis in STR (*P* < 0.05 in green). The methylation difference is measured per PRS unit. Illumina and Genomic Regions Enrichment of Annotation Tool (GREAT) annotation[^1^](#_ENREF_1) is listed for each DMP.

| **Probe ID** | **Genomic position (hg19)** | **Illumina gene annotation** | **Gene region** | **GREAT annotation** [**^1^**](#_ENREF_1) | **Methylation difference (%) STR** | ***P* STR** | **Methylation difference PFC (%)** | ***P* PFC** | **Methylation difference HC (%)** | ***P* HC** | **Methylation difference CER (%)** | ***P* CER** | **Disease STR *P*** | **Disease STR methylation difference (%)** |
| --- | --- | --- | --- | --- | --- | --- | --- | --- | --- | --- | --- | --- | --- | --- |
| cg26893445 | chr15:85924187 | AKAP13 | 5'UTR | AKAP13 | 0.15 | 6.73E-08 | -0.01 | 0.71 | 0.13 | 0.13 | -0.03 | 0.17 | 0.81 | 0.05 |
| cg12595281 | chr15:93633172 | RGMA | TSS1500 | RGMA | 0.68 | 6.85E-08 | 0.16 | 0.33 | 0.28 | 0.36 | 0.26 | 0.19 | 0.97 | -0.04 |
| cg13567870 | chr22:24985852 | C22orf36; GGT1 | Body; 5'UTR | GGT1; PIWIL3 | 0.24 | 2.29E-07 | 0.13 | 0.31 | 0.37 | 0.27 | 0.03 | 0.61 | 0.11 | 0.54 |
| cg25188724 | chr15:58674907 | - | - | LIPC; AQP9 | 0.50 | 4.16E-07 | -0.01 | 0.89 | 0.19 | 0.43 | 0.07 | 0.46 | 0.14 | 0.95 |
| cg18651578 | chr20:5844315 | C20orf196 | 3'UTR | GPCPD1; CHGB | 0.46 | 5.30E-07 | 0.05 | 0.77 | 0.05 | 0.88 | 0.09 | 0.54 | 0.72 | 0.28 |
| cg13954067 | chr9:136679658 | VAV2 | Body | SARDH; VAV2 | -0.65 | 6.87E-07 | -0.29 | 0.05 | 0.20 | 0.46 | -0.27 | 0.14 | 1.17E-03 | -2.78 |
| cg25327452 | chr9:100000464 | KIAA1529 | TSS1500 | C9orf174; ZNF322 | -0.19 | 7.01E-07 | 0.03 | 0.33 | -0.03 | 0.66 | -0.02 | 0.62 | 6.06E-03 | -0.73 |
| cg25789405 | chr7:156263914 | - | - | SHH; C7orf13 | -0.16 | 7.65E-07 | -0.02 | 0.76 | 0.07 | 0.39 | -1.69E-03 | 0.97 | 0.76 | -0.09 |
| cg06854438 | chr1:220263111 | BPNT1 | 1stExon; 5'UTR | IARS2; BPNT1 | -0.15 | 7.82E-07 | 0.05 | 0.50 | -0.05 | 0.76 | -0.03 | 0.49 | 0.28 | -0.26 |
| cg26064870 | chr8:102944342 | NCALD | 5'UTR | NCALD; GRHL2 | 0.36 | 7.84E-07 | 0.05 | 0.47 | 0.03 | 0.83 | 2.45E-03 | 0.98 | 0.02 | 1.06 |
| cg03293330 | chr3:187385312 | - | - | SST; RTP4 | 0.63 | 1.09E-06 | - | - | -0.05 | 0.85 | 0.28 | 0.11 | 0.04 | 1.76 |
| cg14372324 | chr20:30347798 | TPX2 | Body | MYLK2; TPX2 | 1.32 | 1.11E-06 | 0.60 | 0.08 | 1.66 | 0.07 | 1.00 | 1.31E-03 | 0.03 | 3.54 |
| cg15674825 | chr5:150052349 | MYOZ3 | Body | MYOZ3; RBM22 | 0.42 | 1.16E-06 | -0.01 | 0.88 | -0.36 | 0.20 | -0.10 | 0.62 | 0.83 | 0.15 |
| cg05415496 | chr17:79519191 | C17orf70 | 1stExon; 5'UTR | C17orf70 | -0.07 | 1.25E-06 | -0.01 | 0.65 | 0.03 | 0.08 | -3.09E-04 | 0.98 | 0.01 | -0.22 |
| cg24646359 | chr10:102288877 | NDUFB8 | Body | NDUFB8 | -0.18 | 1.38E-06 | 0.01 | 0.77 | 0.04 | 0.82 | 3.40E-03 | 0.92 | 0.02 | -0.60 |
| cg23664774 | chr22:29446636 | ZNRF3 | Body | KREMEN1; ZNRF3 | 0.47 | 1.62E-06 | 0.02 | 0.86 | 0.10 | 0.71 | 0.05 | 0.83 | 0.89 | 0.10 |
| cg07986469 | chr10:129795003 | PTPRE | 5'UTR | PTPRE; MKI67 | 0.57 | 1.71E-06 | 0.20 | 0.06 | 0.09 | 0.68 | -0.01 | 0.85 | 0.39 | 0.68 |
| cg09493966 | chr16:51165636 | - | - | SALL1; CYLD | 0.50 | 2.04E-06 | 0.01 | 0.95 | -0.35 | 0.03 | -0.18 | 0.21 | 0.38 | 0.60 |
| cg19376461 | chr7:110358708 | IMMP2L | Body | LRRN3 | 0.43 | 2.11E-06 | -0.17 | 0.18 | 0.32 | 0.07 | -0.14 | 0.54 | 0.40 | 0.51 |
| cg06452647 | chr12:49961750 | MCRS1 | 5'UTR | MCRS1 | -0.11 | 2.40E-06 | 0.03 | 0.42 | -0.01 | 0.92 | -0.10 | 2.93E-03 | 0.07 | -0.32 |
| cg11427534 | chr5:132155233 | - | - | SEPT8; SHROOM1 | 0.47 | 2.48E-06 | 0.04 | 0.78 | 0.02 | 0.93 | -0.39 | 0.08 | 0.86 | -0.19 |
| cg01020037 | chr6:31047822 | - | - | C6orf15; MUC22 | -0.26 | 3.09E-06 | 0.09 | 0.27 | -0.36 | 0.03 | -0.07 | 0.43 | 0.10 | -0.72 |
| cg22244135 | chr7:7226343 | C1GALT1 | 5'UTR | C1GALT1; COL28A1 | 0.22 | 3.33E-06 | -0.06 | 0.27 | 0.17 | 0.07 | 0.13 | 0.09 | 6.54E-03 | 0.93 |
| cg15022015 | chr17:78869527 | RPTOR | Body | CHMP6; RPTOR | 0.28 | 3.82E-06 | 0.10 | 0.17 | 0.13 | 0.12 | 0.07 | 0.42 | 0.20 | 0.58 |
| cg17689581 | chr2:82508075 | - | - | NONE | 0.36 | 4.35E-06 | -0.05 | 0.69 | -0.20 | 0.34 | 0.23 | 3.72E-03 | 0.28 | 0.56 |
| cg26570279 | chr16:58324876 | KLKBL4 | Body | PRSS54; CCDC113 | 0.22 | 4.60E-06 | -0.06 | 0.36 | 0.08 | 0.16 | 0.05 | 0.58 | 0.60 | 0.18 |
| cg12988813 | chr7:27946471 | JAZF1 | Body | TAX1BP1; JAZF1 | 0.53 | 5.20E-06 | 0.21 | 0.20 | 0.16 | 0.49 | 0.16 | 0.05 | 0.13 | 1.18 |
| . | chr3:71027020 | FOXP1 | Body | FOXP1 | 0.30 | 5.39E-06 | 0.09 | 0.48 | -0.01 | 0.97 | 0.34 | 0.02 | 0.14 | 0.74 |
| cg17095753 | chr7:140623924 | BRAF | Body | BRAF | -0.19 | 6.72E-06 | 0.01 | 0.78 | -0.13 | 0.13 | -0.02 | 0.66 | 0.25 | -0.34 |
| cg22129639 | chr15:43785364 | TP53BP1 | TSS200; 5'UTR | TP53BP1 | -0.43 | 7.13E-06 | -0.04 | 0.44 | -0.37 | 0.19 | -0.04 | 0.61 | 0.25 | -0.81 |
| cg20548182 | chr12:59426481 | - | - | SLC16A7; LRIG3 | 0.26 | 7.68E-06 | -0.04 | 0.65 | -0.07 | 0.54 | 0.15 | 0.11 | 0.63 | 0.21 |
| cg03292206 | chr2:111424914 | BUB1 | Body | RGPD6; BUB1 | 0.52 | 7.79E-06 | 0.14 | 0.45 | -0.07 | 0.81 | 0.03 | 0.81 | 0.06 | 1.44 |
| cg26509022 | chr15:101419296 | ALDH1A3 | TSS1500 | ALDH1A3 | 0.61 | 8.77E-06 | -0.02 | 0.90 | -0.06 | 0.83 | 0.53 | 0.15 | 0.12 | 1.55 |
| cg22708290 | chr12:56368226 | RAB5B | 5'UTR | RAB5B | -0.52 | 8.99E-06 | -0.01 | 0.94 | -0.02 | 0.92 | -0.21 | 0.18 | 0.61 | -0.41 |
| cg02549170 | chr4:186436021 | PDLIM3 | Body | CCDC110; PDLIM3 | 0.19 | 9.08E-06 | -0.01 | 0.91 | -0.03 | 0.74 | 0.01 | 0.89 | 0.11 | 0.51 |
| cg12449325 | chr11:34814081 | - | - | PDHX; EHF | 0.40 | 9.42E-06 | -0.05 | 0.44 | 0.03 | 0.90 | 0.06 | 0.43 | 0.33 | 0.56 |
| cg08135379 | chr12:47474763 | AMIGO2 | TSS1500 | AMIGO2 | 0.42 | 1.00E-05 | 0.04 | 0.80 | 0.02 | 0.91 | 0.13 | 0.43 | 0.45 | 0.51 |
| cg26008841 | chr1:55450605 | TMEM61 | Body | BSND; TMEM61 | 0.33 | 1.14E-05 | 0.07 | 0.35 | 0.11 | 0.52 | 0.10 | 0.39 | 0.19 | 0.68 |
| cg08600218 | chr7:139412996 | HIPK2 | Body | HIPK2; CLEC2L | 0.44 | 1.20E-05 | 0.01 | 0.95 | 0.30 | 0.12 | 0.06 | 0.48 | 0.60 | 0.37 |
| cg21940313 | chr17:41620911 | ETV4 | Body | ETV4; DHX8 | 0.44 | 1.27E-05 | 0.14 | 0.18 | -0.05 | 0.84 | 0.46 | 3.82E-03 | 0.18 | 0.89 |
| cg26075039 | chr2:121684535 | GLI2 | Body | GLI2; TFCP2L1 | 1.23 | 1.32E-05 | 0.89 | 0.02 | -0.75 | 0.19 | 0.42 | 0.18 | 0.60 | 0.91 |
| cg17066531 | chr6:36922415 | PI16 | 5'UTR; 1stExon | PI16 | 0.46 | 1.34E-05 | -0.03 | 0.78 | -0.16 | 0.52 | 0.20 | 0.43 | 0.26 | 0.78 |
| cg27263448 | chr7:127637871 | C7orf54; SND1 | Body | LRRC4; SND1 | 0.15 | 1.42E-05 | 0.02 | 0.78 | 0.05 | 0.66 | -0.04 | 0.85 | 0.32 | 0.28 |
| cg05544885 | chr16:88807707 | FAM38A | Body | CTU2; PIEZO1 | 0.33 | 1.46E-05 | 0.14 | 0.08 | -0.03 | 0.86 | 0.15 | 0.05 | 0.19 | 0.71 |
| cg01379237 | chr3:189983744 | - | - | LEPREL1; CLDN1 | 0.57 | 1.63E-05 | 0.16 | 0.42 | 0.13 | 0.60 | 0.03 | 0.87 | 0.48 | -0.16 |
| cg05494483 | chr5:17001489 | - | - | BASP1; MYO10 | -0.32 | 1.67E-05 | 0.25 | 0.03 | 0.07 | 0.60 | 0.11 | 0.24 | 6.99E-03 | 1.48 |
| cg02141675 | chr5:153569408 | GALNT10 | TSS1500 | GALNT10 | -0.19 | 1.74E-05 | -0.01 | 0.83 | -0.14 | 0.20 | 0.04 | 0.59 | 0.07 | 1.71 |
| cg10939579 | chr13:20768309 | GJB2 | TSS1500 | GJB2 | -0.68 | 1.76E-05 | -0.09 | 0.65 | 0.34 | 0.40 | - | - | 0.32 | -0.53 |
| cg11593949 | chr7:45927735 | IGFBP1 | TSS1500 | IGFBP1 | 0.38 | 1.79E-05 | 0.20 | 0.17 | 0.40 | 0.12 | -0.04 | 0.84 | 0.04 | -0.65 |
| cg08820821 | chr19:49588236 | SNRNP70 | TSS1500 | SNRNP70 | -0.23 | 1.80E-05 | 0.03 | 0.62 | 0.02 | 0.77 | 0.00 | 0.97 | 0.53 | 0.79 |

**Supplementary Table 13: Top ranked polygenic risk score-associated differently methylated probes (DMPs) identified in the hippocampus (HC) linear regression.** Listed for each HC DMP (grey) are corresponding results from the prefrontal cortex (PFC; *P* < 0.05 in blue), striatum (STR; *P* < 0.05 in green) and cerebellum (CER; *P* < 0.05 in orange) meta-analyses. Also shown is the association with schizophrenia diagnosis in HC (*P* < 0.05 in red). The methylation difference is measured per PRS unit. Illumina and Genomic Regions Enrichment of Annotation Tool (GREAT) annotation[^1^](#_ENREF_1) is listed for each DMP.

| **Probe ID** | **Genomic position (hg19)** | **Illumina gene annotation** | **Gene region** | **GREAT annotation** [**^1^**](#_ENREF_1) | **Methylation difference (%) HC** | ***P* HC** | **Methylation difference PFC (%)** | ***P* PFC** | **Methylation difference STR (%)** | ***P* STR** | **Methylation difference CER (%)** | ***P* CER** | **Disease HC methylation difference (%)** | **Disease HC *P*** |
| --- | --- | --- | --- | --- | --- | --- | --- | --- | --- | --- | --- | --- | --- | --- |
| cg03075791 | chr2:120774652 | EPB41L5 | 5'UTR | EPB41L5; TMEM185B | 0.48 | 2.85E-06 | 0.16 | 0.27 | -0.01 | 0.90 | 0.15 | 0.07 | 2.96 | 7.94E-05 |
| cg01305596 | chr16:16228299 | ABCC1 | Body | ABCC6; ABCC1 | 0.12 | 3.67E-06 | 0.01 | 0.71 | 0.03 | 0.11 | 2.30E-03 | 0.91 | 0.57 | 6.14E-03 |
| cg16365352 | chr1:236954819 | - | - | MTR | 0.63 | 4.88E-06 | -0.01 | 0.87 | 0.02 | 0.81 | 0.11 | 0.26 | 3.21 | 2.04E-03 |
| cg04074321 | chr10:88296423 | - | - | OPN4; WAPAL | -0.92 | 6.59E-06 | 0.04 | 0.74 | 0.17 | 0.19 | 0.05 | 0.79 | -5.04 | 1.45E-03 |
| cg00555456 | chr7:96745696 | ACN9 | TSS1500 | ACN9 | -0.85 | 6.96E-06 | -0.11 | 0.57 | 0.11 | 0.33 | 0.05 | 0.72 | -4.49 | 1.77E-03 |
| cg05239158 | chr15:35842016 | - | - | ZNF770 | -0.97 | 7.69E-06 | -0.08 | 0.62 | 0.23 | 0.14 | 0.21 | 0.33 | -5.20 | 8.73E-04 |
| cg02315597 | chr19:52598999 | ZNF841 | 5'UTR; 1stExon | ZNF841 | -0.20 | 1.02E-05 | 0.02 | 0.54 | -0.04 | 0.28 | -0.03 | 0.52 | -0.80 | 0.02 |
| cg23811289 | chr14:101440409 | SNORD114-17 | TSS1500 | DIO3; RTL1 | 0.53 | 1.73E-05 | -0.14 | 0.41 | 0.11 | 0.36 | 0.23 | 0.21 | 2.72 | 4.38E-03 |
| cg22502856 | chr1:209825678 | LAMB3 | TSS1500; 5'UTR; TSS200 | LAMB3 | 0.37 | 2.14E-05 | 0.05 | 0.29 | -0.06 | 0.31 | 6.86E-04 | 0.99 | 2.06 | 1.40E-03 |
| cg04487827 | chr1:44434389 | DPH2 | TSS1500 | DPH2 | -0.60 | 2.18E-05 | -0.23 | 2.26E-03 | 0.09 | 0.41 | 0.07 | 0.45 | -3.64 | 1.98E-03 |
| cg27105205 | chr1:154934396 | PYGO2 | TSS200 | PYGO2 | 0.11 | 2.33E-05 | -0.01 | 0.61 | -0.01 | 0.41 | 1.92E-04 | 0.99 | 0.53 | 0.02 |
| cg01200150 | chr18:76322683 | - | - | SALL3 | -1.37 | 2.75E-05 | -0.11 | 0.46 | 0.13 | 0.49 | 0.31 | 0.05 | -6.31 | 0.01 |
| cg19832184 | chr20:33578069 | MYH7B; MIR499 | Body; TSS200 | MYH7B; TRPC4AP | 0.53 | 2.82E-05 | 0.06 | 0.52 | 0.01 | 0.92 | 0.09 | 0.38 | 2.75 | 3.02E-03 |
| cg08370082 | chr3:196616876 | SENP5 | Body | SENP5; NCBP2 | 1.16 | 3.14E-05 | 0.18 | 0.40 | -0.15 | 0.46 | - | - | 4.83 | 0.02 |
| cg25246281 | chr17:27188748 | MIR451; MIR144 | TSS1500; TSS200 | ERAL1; FLOT2 | 0.76 | 3.84E-05 | 0.06 | 0.58 | 0.02 | 0.80 | -0.09 | 0.50 | 4.17 | 1.40E-03 |
| cg14833040 | chr6:47197781 | - | - | GPR110; TNFRSF21 | 0.24 | 3.93E-05 | 0.05 | 0.32 | 0.10 | 0.04 | 0.04 | 0.50 | 1.03 | 0.02 |
| cg26045524 | chr7:150035522 | RARRES2 | 3'UTR | ACTR3C; RARRES2 | 0.55 | 3.95E-05 | 0.04 | 0.72 | 0.31 | 0.02 | 0.20 | 0.23 | 2.48 | 0.02 |
| cg11956108 | chr1:1895061 | KIAA1751 | Body | GABRD; TMEM52 | 0.31 | 3.96E-05 | 0.07 | 0.45 | -0.11 | 0.21 | 0.03 | 0.73 | 1.65 | 6.54E-03 |
| cg14183864 | chr2:74699903 | MRPL53 | 5'UTR; 1stExon | MRPL53 | -0.15 | 4.03E-05 | 0.01 | 0.70 | -0.01 | 0.77 | 0.03 | 0.41 | -0.77 | 5.82E-03 |
| cg18961589 | chr10:133598669 | - | - | PPP2R2D | -0.38 | 4.21E-05 | -0.02 | 0.82 | 0.07 | 0.39 | 0.09 | 0.24 | -2.34 | 7.37E-04 |
| cg20810675 | chr4:171604188 | - | - | AADAT | 0.94 | 4.29E-05 | 0.12 | 0.53 | -0.03 | 0.87 | -0.04 | 0.82 | 4.91 | 5.79E-03 |
| cg09936645 | chr1:207627581 | CR2 | TSS200 | CR2 | -0.06 | 4.75E-05 | 0.03 | 0.05 | 0.02 | 0.37 | -3.50E-03 | 0.82 | -0.22 | 0.05 |
| cg14069049 | chr4:11430698 | HS3ST1 | TSS200 | HS3ST1 | -0.19 | 5.01E-05 | 0.09 | 0.02 | 2.38E-03 | 0.93 | -0.01 | 0.64 | -1.12 | 1.45E-03 |
| cg18290739 | chr6:10389490 | - | - | OFCC1; TFAP2A | 0.95 | 5.28E-05 | -0.10 | 0.58 | 0.03 | 0.85 | 0.14 | 0.38 | 3.57 | 0.05 |
| cg01422136 | chr5:132362224 | ZCCHC10 | 1stExon | ZCCHC10 | -0.23 | 5.50E-05 | 0.02 | 0.57 | -0.07 | 0.02 | -0.01 | 0.78 | -1.56 | 1.75E-05 |
| cg19159842 | chr17:73727435 | ITGB4 | Body | ITGB4; GALK1 | 0.58 | 5.65E-05 | 0.08 | 0.55 | 0.02 | 0.83 | -1.05E-03 | 0.99 | 1.97 | 0.12 |
| cg15043711 | chr6:31797954 | HSPA1B | 3'UTR; 1stExon | HSPA1B; NEU1 | -0.39 | 5.68E-05 | -0.04 | 0.71 | -0.16 | 0.02 | -0.18 | 0.04 | -2.20 | 3.39E-03 |
| cg07057342 | chr14:31915923 | C14orf126 | 3'UTR | HEATR5A; C14orf126 | 0.83 | 5.75E-05 | -0.05 | 0.75 | -0.17 | 0.29 | 0.36 | 5.90E-03 | 3.90 | 7.32E-03 |
| cg18959411 | chr6:160182805 | ACAT2 | TSS200 | ACAT2 | -0.13 | 5.76E-05 | -0.04 | 0.44 | -0.04 | 0.23 | -0.04 | 0.16 | -0.92 | 8.64E-04 |
| cg02441618 | chr17:40936570 | WNK4 | Body | WNK4; CCDC56 | -0.89 | 5.91E-05 | 0.11 | 0.32 | -0.03 | 0.84 | -0.14 | 0.44 | -3.69 | 0.06 |
| cg14228238 | chr3:168864123 | MECOM | TSS200; Body; 5'UTR | MECOM | -0.60 | 5.94E-05 | 0.16 | 0.16 | 0.15 | 0.13 | 0.01 | 0.81 | -2.56 | 0.02 |
| cg01204911 | chr5:142187317 | ARHGAP26 | Body | ARHGAP26; NR3C1 | -0.69 | 6.27E-05 | 0.05 | 0.64 | -0.09 | 0.39 | 0.01 | 0.79 | -2.60 | 0.03 |
| cg23433530 | chr15:93014435 | C15orf32 | TSS1500 | ST8SIA2; FAM174B | 0.69 | 6.37E-05 | 0.04 | 0.64 | 0.10 | 0.23 | 0.12 | 0.19 | 3.87 | 1.03E-03 |
| cg06135282 | chr13:86370102 | SLITRK6 | Body | SLITRK6 | 0.43 | 6.45E-05 | 0.08 | 0.45 | -0.06 | 0.38 | 0.10 | 0.16 | 2.23 | 2.99E-03 |
| cg21985690 | chr5:79330669 | THBS4 | TSS1500 | THBS4 | 1.45 | 6.52E-05 | 0.04 | 0.79 | 0.08 | 0.57 | 0.10 | 0.56 | 7.31 | 3.37E-03 |
| cg07158797 | chr1:215740701 | KCTD3 | TSS200 | KCTD3 | 0.20 | 6.92E-05 | 2.35E-03 | 0.92 | 0.00 | 0.97 | -0.02 | 0.51 | 1.37 | 4.22E-05 |
| cg15815726 | chr6:7129714 | RREB1 | 5'UTR | RREB1; SSR1 | 0.95 | 6.98E-05 | 0.29 | 0.13 | 0.25 | 0.27 | -0.04 | 0.84 | 5.35 | 3.06E-03 |
| cg12100385 | chr14:88602662 | - | - | GPR65; KCNK10 | 0.35 | 7.29E-05 | -0.01 | 0.88 | -0.06 | 0.41 | 0.08 | 0.11 | 0.93 | 0.18 |
| cg03196189 | chr12:51488916 | TFCP2 | 3'UTR | CSRNP2; TFCP2 | 0.35 | 7.36E-05 | -0.01 | 0.94 | 0.07 | 0.22 | -0.04 | 0.74 | 2.01 | 1.95E-03 |
| cg10374499 | chr11:44601397 | CD82 | 5'UTR | TSPAN18; CD82 | 0.84 | 7.39E-05 | 0.18 | 0.13 | 0.24 | 0.14 | 0.01 | 0.92 | 2.65 | 0.13 |
| cg05529754 | chr16:85045486 | ZDHHC7 | TSS1500 | ZDHHC7 | -0.25 | 7.44E-05 | -0.01 | 0.82 | -0.06 | 0.22 | 0.03 | 0.39 | -1.27 | 4.49E-03 |
| cg18187593 | chr6:33290972 | DAXX | TSS200 | DAXX | -0.18 | 9.01E-05 | 0.00 | 0.91 | -0.01 | 0.79 | 0.02 | 0.50 | -0.81 | 0.01 |
| cg12930930 | chr11:5372503 | OR51B6 | TSS1500 | OR51B6 | 0.86 | 9.49E-05 | 0.25 | 0.22 | 0.33 | 0.01 | 0.22 | 0.20 | 4.25 | 6.28E-03 |
| cg00060320 | chr3:134369974 | KY | TSS200 | KY | -0.10 | 9.58E-05 | 0.03 | 0.32 | -0.05 | 0.09 | -0.01 | 0.69 | -0.67 | 3.99E-04 |
| cg02863947 | chr3:119499190 | NR1I2 | TSS200 | NR1I2 | 0.32 | 9.73E-05 | -0.01 | 0.90 | 0.04 | 0.50 | 0.05 | 0.53 | 2.01 | 2.90E-04 |
| cg21759953 | chr2:121748257 | GLI2 | 3'UTR | GLI2; TFCP2L1 | 0.30 | 9.83E-05 | -0.03 | 0.68 | 0.03 | 0.56 | -0.03 | 0.63 | 1.56 | 3.39E-03 |
| cg01962750 | chr2:8298983 | - | - | ID2 | -0.61 | 9.90E-05 | 0.08 | 0.52 | -0.03 | 0.75 | -0.12 | 0.37 | -2.72 | 0.02 |
| cg04944537 | chr10:125428817 | GPR26 | Body | GPR26; CPXM2 | 0.49 | 1.01E-04 | 0.06 | 0.62 | 0.08 | 0.29 | -4.56E-03 | 0.95 | 2.61 | 2.22E-03 |
| cg16216907 | chr7:42267430 | GLI3 | 5'UTR | INHBA; GLI3 | -1.13 | 1.08E-04 | 0.13 | 0.44 | 0.03 | 0.89 | -0.06 | 0.64 | -4.99 | 0.01 |
| cg09442740 | chr7:100482960 | SRRT | Body | UFSP1; SRRT | 0.23 | 1.09E-04 | 0.03 | 0.56 | -0.06 | 0.69 | 0.03 | 0.72 | 1.60 | 1.48E-04 |

**Supplementary Table 14: Top ranked polygenic risk score-associated differently methylated probes (DMPs) identified in the cerebellum (CER) meta-analysis.** Listed for each CER DMP (grey) are corresponding results from the prefrontal cortex (PFC; *P* < 0.05 in blue), striatum (STR; *P* < 0.05 in green) and hippocampus (HC; *P* < 0.05 in red) meta-analyses (PFC, STR and CER) or linear regression (HC). Also shown is the association with schizophrenia diagnosis in CER (*P* < 0.05 in orange). The methylation difference is measured per PRS unit. Illumina and Genomic Regions Enrichment of Annotation Tool (GREAT) annotation[^1^](#_ENREF_1) is listed for each DMP.

| **Probe ID** | **Genomic position (hg19)** | **Illumina gene annotation** | **Gene region** | **GREAT annotation** [**^1^**](#_ENREF_1) | **Methylation difference (%) CER** | ***P* CER** | **Methylation difference PFC (%)** | ***P* PFC** | **Methylation difference STR (%)** | ***P* STR** | **Methylation difference HC (%)** | ***P* HC** | **Disease CER methylation difference (%)** | **Disease CER *P*** |
| --- | --- | --- | --- | --- | --- | --- | --- | --- | --- | --- | --- | --- | --- | --- |
| cg20640266 | chr9:116811789 | ZNF618 | Body | AMBP; ZNF618 | 0.60 | 1.62E-09 | -0.02 | 0.93 | 0.12 | 0.10 | 0.02 | 0.88 | 1.20 | 0.11 |
| cg27150552 | chr7:48026856 | SUNC1 | 3'UTR | HUS1; SUN3 | 0.34 | 1.30E-08 | -0.13 | 0.30 | -0.02 | 0.89 | 0.47 | 0.06 | 0.84 | 0.18 |
| cg05209768 | chr2:164573665 | FIGN | Body | KCNH7; FIGN | 0.70 | 1.55E-08 | -0.04 | 0.82 | 0.01 | 0.96 | 0.47 | 0.16 | 2.00 | 0.04 |
| cg07793808 | chr12:122019006 | KDM2B | TSS200; TSS1500 | KDM2B | -0.19 | 1.66E-08 | 0.05 | 0.62 | -0.02 | 0.91 | -0.39 | 0.23 | -0.23 | 0.36 |
| cg10218777 | chr3:133180261 | BFSP2 | Body | CDV3; BFSP2 | 0.67 | 3.86E-08 | 0.06 | 0.69 | 0.05 | 0.79 | 0.45 | 0.20 | 2.91 | 6.54E-03 |
| cg01682070 | chr16:29996774 | TAOK2 | Body | HIRIP3; TAOK2 | 0.32 | 4.20E-08 | 0.03 | 0.64 | 0.04 | 0.50 | 0.02 | 0.83 | 0.87 | 0.04 |
| cg11786558 | chr17:2266589 | SGSM2 | Body | SGSM2; MNT | 0.69 | 4.35E-08 | 0.04 | 0.80 | 0.02 | 0.87 | -0.10 | 0.69 | 1.28 | 0.14 |
| cg26053083 | chr11:14995770 | - | - | CALCA | -0.15 | 4.41E-08 | -0.02 | 0.70 | -0.06 | 0.10 | -0.08 | 0.19 | -0.29 | 0.14 |
| cg01022840 | chr14:71250264 | MAP3K9 | Body | MAP3K9; TTC9 | 0.63 | 7.86E-08 | -0.01 | 0.91 | 0.13 | 0.20 | 0.34 | 0.25 | 1.94 | 0.03 |
| cg08478539 | chr15:68640339 | ITGA11 | Body | FEM1B; ITGA11 | 0.69 | 1.06E-07 | 1.24E-03 | 0.99 | 0.11 | 0.41 | 0.02 | 0.94 | 1.41 | 0.14 |
| cg23788334 | chr2:137181176 | - | - | THSD7B; CXCR4 | -0.13 | 1.15E-07 | -0.04 | 0.37 | 0.01 | 0.75 | -0.05 | 0.53 | -0.15 | 0.38 |
| cg16904520 | chr2:230590962 | - | - | DNER; TRIP12 | 0.26 | 1.25E-07 | -0.04 | 0.79 | 0.10 | 0.60 | 0.08 | 0.78 | 1.27 | 9.37E-04 |
| cg09987651 | chr6:27463667 | - | - | ZNF184; HIST1H2BL | -0.50 | 1.76E-07 | 0.01 | 0.96 | -0.08 | 0.42 | -0.31 | 0.06 | -0.86 | 0.24 |
| cg03314644 | chr14:31890001 | - | - | HEATR5A | -0.34 | 1.82E-07 | -0.01 | 0.93 | -0.04 | 0.74 | -0.36 | 0.14 | -0.48 | 0.29 |
| cg03812240 | chr9:125107355 | - | - | RBM18; PTGS1 | 0.28 | 2.13E-07 | -4.47E-03 | 0.95 | 0.04 | 0.59 | 0.03 | 0.92 | 0.23 | 0.60 |
| cg13846270 | chr13:51417929 | DLEU7 | TSS200 | RNASEH2B; ST13P4 | -0.84 | 2.55E-07 | -0.11 | 0.50 | -0.13 | 0.38 | -0.44 | 0.14 | -3.95 | 2.06E-04 |
| cg14595617 | chr2:3105137 | - | - | MYT1L; TRAPPC12 | 0.48 | 2.96E-07 | 0.02 | 0.71 | 0.14 | 0.01 | -0.04 | 0.75 | 0.13 | 0.84 |
| cg07984684 | chr16:89724635 | C16orf55; CHMP1A | Body; TSS1500 | CHMP1A | -0.19 | 3.12E-07 | 0.04 | 0.52 | 0.01 | 0.89 | -0.06 | 0.57 | -0.44 | 0.11 |
| cg17788761 | chr1:78340579 | FAM73A | Body | NEXN; FAM73A | 0.62 | 3.15E-07 | -0.04 | 0.85 | 0.07 | 0.55 | 0.58 | 0.02 | 2.34 | 8.32E-03 |
| cg06343355 | chr6:29521023 | - | - | MAS1L; UBD | -0.56 | 3.72E-07 | 0.13 | 0.25 | -0.09 | 0.37 | 2.23E-03 | 0.99 | -0.99 | 0.30 |
| cg08853103 | chr17:79514978 | C17orf70 | Body | C17orf70; FSCN2 | 0.40 | 5.87E-07 | -0.02 | 0.83 | 0.01 | 0.93 | 0.17 | 0.22 | 1.51 | 0.02 |
| cg01580578 | chr7:156889041 | - | - | MNX1; UBE3C | -0.72 | 6.52E-07 | -0.28 | 0.06 | -0.19 | 0.16 | -0.53 | 0.07 | -1.68 | 0.10 |
| cg02583247 | chr12:4555311 | FGF6 | TSS1500 | FGF6 | 0.37 | 6.96E-07 | -0.07 | 0.35 | 5.17E-04 | 0.99 | 0.32 | 0.01 | 0.64 | 0.22 |
| cg12374123 | chr15:45406327 | DUOX2; DUOXA2 | 5'UTR; TSS200; 1stExon | DUOXA2; DUOX2 | -0.59 | 7.45E-07 | -0.17 | 0.17 | 0.08 | 0.48 | -0.27 | 0.15 | -2.12 | 0.01 |
| cg19321126 | chr2:242211919 | HDLBP | 5'UTR | HDLBP | 0.09 | 7.81E-07 | 0.07 | 0.02 | -4.24E-03 | 0.87 | 0.09 | 0.14 | 0.41 | 3.40E-03 |
| cg20755721 | chr21:38070524 | SIM2 | TSS1500 | SIM2 | -0.49 | 8.17E-07 | 0.03 | 0.74 | 0.04 | 0.70 | -0.32 | 0.14 | -1.19 | 0.09 |
| cg05919625 | chr18:32870178 | ZNF271; ZNF397OS | TSS200; 5'UTR; 1stExon | ZNF271; ZSCAN30 | -0.10 | 8.17E-07 | 0.02 | 0.37 | -3.25E-03 | 0.87 | -0.05 | 0.16 | -0.18 | 0.21 |
| cg19575244 | chr6:157094085 | - | - | ARID1B | 0.46 | 8.49E-07 | 0.03 | 0.81 | 0.14 | 0.30 | 0.37 | 0.16 | 1.31 | 0.09 |
| cg15034300 | chr2:85134468 | - | - | KCMF1; TMSB10 | -0.80 | 8.61E-07 | -0.08 | 0.44 | 0.16 | 0.09 | -0.38 | 0.16 | 0.34 | 0.79 |
| cg11468000 | chr6:139694674 | CITED2 | Body | CITED2 | -0.15 | 8.83E-07 | -0.01 | 0.90 | -0.06 | 0.12 | -0.12 | 0.13 | 0.15 | 0.51 |
| cg15506890 | chr2:3487001 | - | - | ADI1; TRAPPC12 | 1.27 | 9.40E-07 | 0.75 | 4.62E-03 | 0.62 | 8.69E-04 | -0.04 | 0.91 | 1.89 | 0.33 |
| cg05447008 | chr6:73331114 | KCNQ5 | TSS1500 | KCNQ5 | -0.09 | 1.01E-06 | -0.02 | 0.54 | -8.27E-04 | 0.98 | 0.03 | 0.53 | -0.22 | 0.09 |
| cg14308867 | chr6:31603476 | BAT2 | Body | PRRC2A; BAG6 | 0.30 | 1.05E-06 | 0.00 | 0.99 | -0.01 | 0.89 | -0.06 | 0.65 | 0.65 | 0.12 |
| cg26656452 | chr10:115313165 | HABP2 | Body | HABP2 | 0.41 | 1.08E-06 | -0.04 | 0.48 | 0.03 | 0.55 | -0.03 | 0.78 | 1.14 | 0.04 |
| cg13159946 | chr20:30073521 | NCRNA00028 | TSS200 | HM13; REM1 | -0.75 | 1.11E-06 | -0.24 | 0.17 | -0.44 | 0.02 | -0.89 | 0.03 | -0.35 | 0.78 |
| cg14597361 | chr12:22778766 | ETNK1 | Body | ETNK1 | -0.16 | 1.14E-06 | -0.02 | 0.75 | -0.07 | 0.07 | 0.02 | 0.76 | -0.29 | 0.22 |
| cg08041245 | chr2:179516579 | TTN; MIR548N | Body | TTN; PLEKHA3 | 0.56 | 1.20E-06 | 0.11 | 0.31 | -0.02 | 0.82 | 0.28 | 0.02 | 2.22 | 0.01 |
| cg24523948 | chr8:72916620 | - | - | MSC; TRPA1 | -0.66 | 1.27E-06 | -0.11 | 0.53 | 0.02 | 0.89 | 0.33 | 0.26 | -2.60 | 6.54E-03 |
| cg10569616 | chr7:124536676 | POT1 | Body; 5'UTR | GPR37; POT1 | 0.45 | 1.37E-06 | -0.21 | 0.26 | 0.07 | 0.51 | 0.22 | 0.37 | 1.07 | 0.11 |
| cg11630392 | chr3:150920964 | GPR171; MED12L | 1stExon; Body; 5'UTR | GPR171 | 0.63 | 1.44E-06 | -0.07 | 0.72 | 0.04 | 0.82 | - | - | 2.23 | 0.02 |
| cg15094071 | chr3:183525728 | YEATS2 | Body | MAP6D1; YEATS2 | 0.29 | 1.58E-06 | -0.05 | 0.50 | -0.02 | 0.80 | -0.25 | 0.20 | 0.63 | 0.13 |
| cg20981127 | chr19:17357587 | NR2F6 | TSS1500 | NR2F6 | -0.60 | 1.59E-06 | -0.02 | 0.87 | -0.05 | 0.45 | -0.32 | 0.15 | -0.84 | 0.40 |
| cg05266784 | chr1:160312524 | COPA; NCSTN | Body; TSS1500 | NCSTN; COPA | -0.19 | 1.60E-06 | -0.03 | 0.58 | -0.02 | 0.74 | -0.16 | 0.30 | -0.23 | 0.42 |
| cg02783970 | chr17:78121126 | EIF4A3 | TSS200 | EIF4A3 | -0.07 | 1.63E-06 | -0.03 | 0.13 | -0.02 | 0.39 | -0.11 | 0.12 | -0.14 | 0.28 |
| cg17152101 | chr1:32509371 | KHDRBS1 | 3'UTR | TMEM39B; KHDRBS1 | 0.47 | 1.63E-06 | -0.03 | 0.85 | 0.13 | 0.22 | 0.05 | 0.84 | 0.91 | 0.30 |
| cg18559785 | chr19:42915372 | LIPE | Body | CNFN; LIPE | 0.37 | 1.65E-06 | -0.23 | 0.06 | -0.04 | 0.71 | 0.07 | 0.69 | 1.07 | 0.09 |
| cg11629408 | chr8:67415435 | C8orf46 | Body | ADHFE1; MYBL1 | 0.28 | 1.65E-06 | 0.01 | 0.90 | 0.27 | 1.14E-03 | 0.18 | 0.29 | 0.18 | 0.65 |
| cg17561452 | chr16:84224707 | ADAD2 | TSS200 | TAF1C; ADAD2 | 0.48 | 1.66E-06 | 0.02 | 0.89 | 0.02 | 0.89 | -0.08 | 0.69 | 1.92 | 0.01 |
| cg02400308 | chr5:160048139 | ATP10B | Body | PTTG1; ATP10B | 0.24 | 1.71E-06 | 0.08 | 0.22 | 0.10 | 0.11 | -0.15 | 0.41 | 0.31 | 0.42 |
| cg06845571 | chr6:108279703 | SEC63 | TSS1500 | SEC63 | -0.18 | 1.72E-06 | 0.01 | 0.84 | -0.05 | 0.44 | -0.22 | 0.12 | -0.28 | 0.26 |

**Supplementary Table 15: For DMPs identified in each of the four individual brain regions, PRS-associated DNA methylation differences are significantly correlated with those at the same probes in the other three brain regions.** Shown is the correlation in PRS-associated DNA methylation differences for the 50 top-ranked DMPs in each of the four brain regions. See also **Supplementary Figure 19**.

|  |  | **Prefrontal cortex** | | **Striatum** | | **Hippocampus** | | **Cerebellum** | |
| --- | --- | --- | --- | --- | --- | --- | --- | --- | --- |
|  |  | **ρ** | ***P*** | **ρ** | ***P*** | **ρ** | ***P*** | **ρ** | ***P*** |
| **50 top ranked probes** | **Prefrontal cortex** | - | - | 0.50 | 2.90E-04 | 0.64 | 7.35E-07 | 0.52 | 1.21E-04 |
|  | **Striatum** | 0.60 | 6.40E-06 | - | - | 0.21 | 0.15 | 0.61 | 4.03E-06 |
|  | **Hippocampus** | 0.33 | 0.02 | 0.01 | 0.92 | - | - | 0.17 | 0.24 |
|  | **Cerebellum** | 0.42 | 0.00 | 0.59 | 5.75E-06 | 0.65 | 3.70E-07 | - | - |

**Supplementary Table 16: Top ranked polygenic risk score-associated differently methylated probes (DMPs) identified in the multiregion model incorporating the prefrontal cortex (PFC), striatum (STR) and hippocampus (HC) data.** Listed for each DMP are corresponding results from the multilevel model (grey) and corresponding results from the prefrontal cortex (PFC; *P* < 0.05 in blue), striatum (STR; *P* < 0.05 in green) and hippocampus (HC; *P* < 0.05 in red) meta-analyses (PFC and STR) or linear regression (HC). Also shown is the association with schizophrenia diagnosis (multi-region model, *P*<0.05 in purple). The methylation difference is measured per PRS unit. Illumina and Genomic Regions Enrichment of Annotation Tool (GREAT) annotation[^1^](#_ENREF_1) is listed for each DMP.

| **Probe ID** | **Genomic position (hg19)** | **Illumina gene annotation** | **Gene region** | **GREAT annotation** [**^1^**](#_ENREF_1) | **Methylation difference (%) multilevel model** | ***P* multilevel model** | **Methylation difference PFC (%)** | ***P* PFC** | **Methylation difference STR (%)** | ***P* STR** | **Methylation difference HC (%)** | ***P* HC** | **Disease methylation difference (%)** | **Disease *P*** | |
| --- | --- | --- | --- | --- | --- | --- | --- | --- | --- | --- | --- | --- | --- | --- | --- |
| cg04910228 | chr1:231739450 | TSNAX-DISC1 | Body | DISC1; TSNAX | -0.38 | 6.50E-07 | -0.23 | 0.03 | -0.43 | 8.49E-05 | -0.54 | 4.56E-03 | -0.97 | 0.09 |  |
| cg00735454 | chr13:44595496 | LOC121838 | TSS1500 | ENOX1; SERP2 | 0.58 | 2.28E-06 | 0.83 | 2.06E-05 | 0.40 | 0.01 | 0.26 | 0.31 | 0.53 | 0.52 |  |
| cg22930808 | chr3:122281881 | PARP9; DTX3L | 5'UTR; TSS1500 | DTX3L | 0.41 | 2.33E-06 | 0.56 | 1.47E-05 | 0.39 | 9.87E-04 | 0.04 | 0.82 | 0.92 | 0.12 |  |
| cg21062760 | chr19:36205574 | ZBTB32 | Body | MLL4 | -0.36 | 2.50E-06 | -0.32 | 5.87E-03 | -0.25 | 0.01 | -0.59 | 0.05 | -1.57 | 3.78E-03 |  |
| cg01495332 | chr7:50798564 | GRB10 | 5'UTR; Body | DDC; GRB10 | -0.52 | 3.67E-06 | -0.28 | 0.10 | -0.59 | 5.72E-04 | -0.61 | 0.03 | -1.93 | 0.02 |  |
| cg09735146 | chr16:85845936 | - | - | IRF8; COX4I1 | -0.49 | 4.39E-06 | -0.40 | 9.68E-03 | -0.27 | 0.06 | -0.82 | 0.01 | -1.99 | 6.11E-03 |  |
| cg12988813 | chr7:27946471 | JAZF1 | Body | TAX1BP1; JAZF1 | 0.42 | 4.98E-06 | 0.21 | 0.20 | 0.53 | 5.20E-06 | 0.16 | 0.49 | 1.09 | 0.08 |  |
| cg14054620 | chr9:38622748 | C9orf122 | Body | IGFBPL1; CNTNAP3 | 0.64 | 7.24E-06 | 0.14 | 0.40 | 0.70 | 3.82E-04 | 0.80 | 0.04 | 2.04 | 0.04 |  |
| cg23539745 | chr12:106142108 | - | - | APPL2; NUAK1 | 0.63 | 8.04E-06 | 0.28 | 0.04 | 0.29 | 0.02 | 0.21 | 0.09 | -0.95 | 0.30 |  |
| cg07535191 | chr11:27250166 | - | - | BBOX1; LGR4 | 0.51 | 8.67E-06 | 0.26 | 0.12 | 0.48 | 1.34E-03 | 1.10 | 4.26E-03 | 1.96 | 7.97E-03 |  |
| cg26064870 | chr8:102944342 | NCALD | 5'UTR | NCALD; GRHL2 | 0.22 | 9.56E-06 | 0.05 | 0.47 | 0.36 | 7.84E-07 | 0.03 | 0.83 | 0.98 | 2.36E-03 |  |
| cg09357276 | chr4:188917856 | ZFP42 | 5'UTR | ZFP42 | -0.49 | 9.74E-06 | -0.30 | 0.02 | -0.44 | 2.70E-03 | -0.75 | 0.07 | -2.24 | 2.76E-03 |  |
| cg26937267 | chr11:1315171 | TOLLIP | Body | TOLLIP; MUC5B | -0.42 | 1.09E-05 | -0.26 | 0.04 | -0.38 | 1.13E-03 | -0.41 | 0.20 | -1.44 | 0.03 |  |
| cg24640510 | chr15:80988694 | FAM108C1 | Body | KIAA1199; FAM108C1 | 0.27 | 1.24E-05 | 0.12 | 0.10 | 0.28 | 5.52E-03 | 0.42 | 0.01 | 0.48 | 0.25 |  |
| cg26840462 | chr11:125220543 | PKNOX2 | 5'UTR | FEZ1; PKNOX2 | -0.25 | 1.31E-05 | -0.25 | 9.52E-03 | -0.11 | 8.15E-03 | -0.07 | 0.52 | -0.56 | 0.16 |  |
| cg11717194 | chr1:11990078 | - | - | PLOD1; KIAA2013 | -0.34 | 1.37E-05 | -0.34 | 2.07E-04 | -0.30 | 0.01 | -0.25 | 0.02 | -1.57 | 2.28E-03 |  |
| cg00324562 | chr21:27106440 | ATP5J; GABPA | 5'UTR; Body; TSS1500 | GABPA | -0.11 | 1.43E-05 | -0.07 | 0.08 | -0.09 | 0.01 | -0.11 | 0.05 | -0.47 | 2.50E-03 |  |
| cg21245653 | chr22:50426164 | - | - | PIM3; MLC1 | 0.67 | 1.53E-05 | 0.66 | 1.09E-04 | 0.23 | 0.07 | 0.05 | 0.60 | -0.90 | 0.37 |  |
| cg12382398 | chr15:90358537 | ANPEP | TSS1500 | ANPEP | -0.30 | 1.55E-05 | -0.09 | 0.31 | -0.36 | 3.55E-04 | -0.57 | 0.03 | -0.83 | 0.09 |  |
| cg08619378 | chr7:45616358 | ADCY1 | Body | IGFBP1; ADCY1 | -0.47 | 1.73E-05 | -0.51 | 3.05E-03 | -0.48 | 1.73E-04 | -0.19 | 0.50 | -1.30 | 0.07 |  |
| cg27247731 | chr10:100994441 | HPSE2 | Body | HPS1; HPSE2 | -0.20 | 2.04E-05 | -0.07 | 0.28 | -0.22 | 4.81E-03 | -0.46 | 3.18E-03 | -0.59 | 0.07 |  |
| cg13379208 | chr6:142564220 | - | - | GPR126; VTA1 | 0.40 | 2.06E-05 | 0.34 | 4.64E-03 | 0.35 | 2.10E-03 | 0.46 | 0.03 | 1.90 | 4.03E-03 |  |
| cg18851960 | chr19:43979739 | PHLDB3 | Body | LYPD3; ETHE1 | -0.38 | 2.07E-05 | -0.35 | 5.42E-03 | -0.31 | 6.27E-03 | -0.29 | 0.34 | -1.20 | 0.05 |  |
| cg00224508 | chr1:151031323 | CDC42SE1; MLLT11 | 5'UTR; TSS1500 | MLLT11; CDC42SE1 | -0.14 | 2.37E-05 | -0.07 | 0.15 | -0.20 | 5.04E-04 | -0.14 | 0.04 | -0.86 | 7.84E-05 |  |
| cg24149904 | chr8:6691719 | XKR5 | Body | XKR5; AGPAT5 | -0.36 | 2.38E-05 | -0.22 | 0.10 | -0.33 | 0.01 | -0.58 | 0.03 | -1.39 | 0.02 |  |
| cg24388251 | chr8:55618220 | - | - | XKR4; RP1 | 0.40 | 2.41E-05 | 0.39 | 9.41E-04 | 0.16 | 0.16 | 0.39 | 0.11 | 1.84 | 2.41E-03 |  |
| cg01275038 | chr4:7287492 | SORCS2 | Body | SORCS2; PSAPL1 | 0.52 | 2.42E-05 | 0.33 | 0.03 | 0.48 | 5.68E-03 | 1.06 | 1.87E-04 | 2.93 | 4.02E-04 |  |
| cg07304068 | chr3:47823820 | SMARCC1 | TSS1500 | SMARCC1 | -0.09 | 2.53E-05 | -0.03 | 0.18 | -0.11 | 9.63E-04 | -0.13 | 0.07 | -0.44 | 2.89E-03 |  |
| cg26574247 | chr17:77712786 | ENPP7 | 3'UTR | CBX2; ENPP7 | 0.39 | 2.59E-05 | 0.42 | 5.88E-03 | 0.24 | 0.03 | 0.37 | 0.17 | 1.31 | 0.03 |  |
| cg16429927 | chr2:172430723 | - | - | DYNC1I2; CYBRD1 | -0.31 | 2.61E-05 | -0.18 | 0.13 | -0.37 | 2.20E-04 | -0.45 | 0.02 | -1.46 | 2.95E-03 |  |
| cg22133973 | chr6:170789640 | - | - | PSMB1; FAM120B | -0.39 | 2.79E-05 | -0.22 | 0.07 | -0.39 | 2.20E-03 | -0.72 | 0.03 | -1.42 | 0.05 |  |
| cg20645973 | chr11:102402091 | MMP7 | TSS1500 | MMP7 | 0.31 | 2.80E-05 | 0.26 | 0.03 | 0.18 | 0.06 | 0.30 | 0.15 | 1.24 | 7.97E-03 |  |
| cg26205771 | chr8:53851156 | NPBWR1 | TSS1500 | NPBWR1 | -0.36 | 2.81E-05 | -0.22 | 0.04 | -0.27 | 0.02 | -0.25 | 0.10 | -1.50 | 0.02 |  |
| cg26019600 | chr17:43978704 | MAPT | 5'UTR | STH; MAPT | -0.57 | 2.99E-05 | -0.41 | 0.02 | -0.42 | 0.03 | -0.80 | 0.04 | -2.81 | 1.59E-03 |  |
| cg27662639 | chr16:22312063 | POLR3E | 5'UTR | POLR3E; CDR2 | -0.34 | 2.99E-05 | -0.24 | 0.08 | -0.38 | 5.83E-04 | -0.42 | 0.04 | -1.56 | 5.79E-03 |  |
| cg25448355 | chr8:121224799 | COL14A1 | Body | COL14A1; MRPL13 | 0.33 | 2.99E-05 | 0.10 | 0.41 | 0.30 | 4.86E-03 | 0.36 | 0.09 | 0.16 | 0.77 |  |
| cg26960370 | chr10:73576410 | PSAP | 3'UTR | C10orf54; PSAP | -0.23 | 3.34E-05 | -0.21 | 0.03 | -0.15 | 0.04 | -0.19 | 0.19 | -1.01 | 7.03E-03 |  |
| cg19746982 | chr18:77552568 | - | - | KCNG2; CTDP1 | 0.32 | 3.37E-05 | 0.28 | 0.02 | 0.30 | 2.77E-03 | 0.14 | 0.44 | 0.73 | 0.15 |  |
| cg02289038 | chr4:24915774 | CCDC149 | TSS1500; 5'UTR | LGI2; SOD3 | 0.21 | 3.40E-05 | 0.06 | 0.40 | 0.19 | 2.02E-03 | 0.47 | 5.61E-03 | 0.90 | 0.01 |  |
| cg18268547 | chr17:79615552 | TSPAN10 | 3'UTR | TSPAN10; PDE6G | -0.45 | 3.42E-05 | -0.52 | 6.02E-03 | -0.53 | 2.18E-04 | -0.19 | 0.61 | -1.45 | 0.04 |  |
| cg15022015 | chr17:78869527 | RPTOR | Body | CHMP6; RPTOR | 0.18 | 3.44E-05 | 0.10 | 0.17 | 0.28 | 3.82E-06 | 0.13 | 0.12 | 0.46 | 0.10 |  |
| cg02683714 | chr8:142500299 | FLJ43860 | Body | FLJ43860; PTP4A3 | 0.76 | 3.49E-05 | 0.32 | 0.11 | 0.34 | 9.76E-04 | 0.17 | 0.24 | -0.87 | 0.46 |  |
| cg21886364 | chr5:39398185 | DAB2 | 5'UTR | C9; DAB2 | 0.88 | 3.52E-05 | 0.68 | 6.07E-03 | 0.81 | 3.11E-04 | 0.55 | 0.18 | 0.39 | 0.78 |  |
| cg23504719 | chr10:99735010 | CRTAC1 | Body | CRTAC1; GOLGA7B | -0.65 | 3.56E-05 | -0.49 | 0.02 | -0.71 | 2.16E-04 | -0.87 | 0.01 | -2.71 | 0.01 |  |
| cg13652372 | chr19:46177461 | MIR642; GIPR | TSS1500; Body | GIPR; SNRPD2 | 0.18 | 3.60E-05 | 0.04 | 0.61 | 0.22 | 1.16E-04 | 0.22 | 0.09 | 0.72 | 8.89E-03 |  |
| cg27326876 | chr5:172098275 | NEURL1B | Body | NEURL1B; DUSP1 | 0.14 | 3.61E-05 | 0.04 | 0.44 | 0.08 | 0.06 | 0.30 | 8.96E-03 | 0.66 | 7.08E-03 |  |
| cg27292417 | chr3:53850724 | CHDH | 3'UTR | CHDH; CACNA1D | 0.64 | 3.70E-05 | 0.32 | 0.06 | 0.57 | 0.01 | 1.41 | 2.44E-03 | 1.55 | 0.13 |  |
| cg09504568 | chr15:93127028 | - | - | FAM174B; ST8SIA2 | 0.72 | 3.70E-05 | 0.52 | 3.67E-03 | 0.30 | 0.05 | 0.03 | 0.75 | -1.04 | 0.36 |  |
| cg20276630 | chr10:97055439 | - | - | PDLIM1 | -0.28 | 3.75E-05 | -0.23 | 0.01 | -0.21 | 0.08 | -0.31 | 0.08 | -1.05 | 0.02 |  |
| cg17865653 | chr18:22005767 | IMPACT | TSS1500 | IMPACT | 0.21 | 3.79E-05 | 0.18 | 0.03 | 0.14 | 0.02 | 0.32 | 0.11 | 0.91 | 8.87E-03 |  |

**Supplementary Table 17: Significant polygenic risk score-associated differently methylated regions (DMRs) identified in the multilevel model incorporating the prefrontal cortex, striatum and hippocampus data.** Shown in chromosomal order is the location of significant (Šidák-corrected *P* < 0.05) DMRs identified in the multilevel model. The column “Gene” represents the combined Illumina and Genomic Regions Enrichment of Annotation Tool (GREAT) annotation [^1^](#_ENREF_1).

| **Region** | **Gene** | **Probes** | **N probes** | **Median *P*** | **Šidák *P*** |
| --- | --- | --- | --- | --- | --- |
| chr1:156358116-156358613 | RHBG; C1orf61 | cg24361162; cg09989644; cg12062819; cg23842796; cg20433275; cg15575683 | 6 | 0.002 | 1.05E-04 |
| chr3:55517806-55518143 | WNT5A; LRTM1 | cg27364162; cg18562578; cg18010752; cg17679453; cg04941246; cg24216596 | 6 | 0.005 | 0.019 |
| chr4:74847646-74847830 | PF4 | cg15158783; cg21043213; cg16072462; cg15398841; cg02530824; cg06834998; cg05509609 | 7 | 0.007 | 0.009 |
| chr5:37834672-37835169 | GDNF; NUP155 | cg07423205; cg21590264; cg26473844; cg18725867; cg08204023; cg18182111; cg20683765 | 7 | 0.002 | 1.73E-06 |
| chr5:92908771-92909070 | FLJ42709; NR2F1 | cg02020829; cg22217860; cg06668065 | 3 | 2.71E-04 | 0.027 |
| chr5:102898223-102898730 | NUDT12 | cg07666882; cg02976617; cg13665998; cg09166085; cg07655627 | 5 | 0.001 | 1.15E-04 |
| chr6:28583971-28584289 | SCAND3; TRIM27 | cg01400884; cg02246609; cg01309870; cg04626491; cg09470274; cg20450471; cg09897374; cg00919411; cg15967709; cg03682719; cg20839206; cg22121557; cg03858673; cg24114014; cg04645150; cg11111740 | 16 | 0.018 | 0.005 |
| chr6:29974715-29975081 | HLA-J ;NCRNA00171 | cg04566848; cg24725574; cg01814945; cg23313031; cg19448822; cg13207333; cg11867546; cg23483840; cg08163199; cg25318809; cg14781281; cg08325845; cg15726260; cg12976581; cg14432143; cg21330423; cg09659004; cg15364169 | 18 | 0.033 | 0.023 |
| chr6:30042919-30043419 | RNF39 | cg12704854; cg11562284; cg02552311; cg03219282; cg24766429; cg23500724; cg10865856; cg24016627; cg23939808; cg12967914; cg00853042; cg23027574; cg05853632; cg22105332; cg19006429; cg27532187; cg01631162 | 17 | 0.022 | 7.36E-04 |
| chr6:30853948-30854234 | DDR1 | cg16215084; cg25251478; cg26321999; cg00934322; cg07187855; cg24566261; cg09965419; cg17091577 | 8 | 0.009 | 0.018 |
| chr6:42927993-42928346 | GNMT | cg25671484; cg16682276; cg23093754; cg24153763; cg27588902; cg09436375; cg17345569; cg07941301; cg04013093; cg11409096; cg23696834 | 11 | 0.020 | 0.029 |
| chr7:29519388-29519657 | CHN2; PRR15 | cg02844647; cg04635849; cg03100044; cg21251563; cg16920620; cg00615485 | 6 | 0.008 | 0.033 |
| chr7:95025736-95025956 | PON3; PON1; PON2 | cg07121856; cg23230584; cg08520743; cg08828819; cg04685170; cg15927196; cg15500865; cg25572105 | 8 | 0.016 | 0.014 |
| chr7:156870822-156871089 | MNX1; UBE3C | cg24880736; cg02456451; cg00173659 | 3 | 8.86E-04 | 0.030 |
| chr8:37824306-37824766 | ADRB3 | cg02174634; cg23460057; cg10357888; cg01386493; cg19806221 | 5 | 5.97E-04 | 2.50E-04 |
| chr10:99734912-99735203 | CRTAC1; GOLGA7B | cg27110886; cg23504719; cg23245905; cg08930881 | 4 | 0.001 | 0.011 |
| chr11:1891872-1892393 | LSP1; TNNT3 | cg27261733; cg08276062; cg24552015; cg09989681; cg20331155; cg21529477; cg22043296; cg26897904; cg26868156; cg15079934; cg08756594 | 11 | 0.006 | 8.89E-05 |
| chr12:54070294-54070592 | ATP5G2 | cg17561241; cg02541613; cg22546318; cg02056144; cg22997177; cg27479634; cg19842134 | 7 | 0.002 | 3.57E-04 |
| chr16:85845626-85845937 | IRF8; COX4I1 | cg03889236; cg05312293; cg06521653; cg09735146 | 4 | 0.003 | 0.009 |
| chr20:32308081-32308481 | PXMP4 | cg27194921; cg25092328; cg20588982; cg06231372; cg12297619; cg24270031; cg04730850 | 7 | 0.008 | 0.006 |
| chr20:37230326-37230613 | C20orf95; ARHGAP40 | cg01025836; cg00557360; cg04608177; cg03356734; cg06301550; cg08438366 | 6 | 9.35E-04 | 3.18E-06 |

**Supplementary Table 18: Methylation QTLs (mQTLs) identified in the prefrontal cortex (PFC) for SNPs included in the schizophrenia polygenic risk score.** Shown are associations between DNA methylation at specific Illumina 450K probes and genetic variants used to derive polygenic risk score (PRS) in our study, in addition to the corresponding *P*-values for PRS-associated DNA methylation variation at the same probe.

| **SNP** | **SNP genomic location (hg19)** | **mQTL *P*** | **CpG** | **CpG genomic location (hg19)** | **EWAS *P*** |
| --- | --- | --- | --- | --- | --- |
| rs9726753 | chr1:153591652 | 5.88E-49 | cg08477332 | chr1:153590243 | 0.36 |
| rs117194038 | chr17:43927290 | 1.65E-47 | cg17117718 | chr17:43663208 | 0.97 |
| rs12635522 | chr3:125708174 | 4.79E-46 | cg15145296 | chr3:125709740 | 0.09 |
| rs35059736 | chr7:158224692 | 1.83E-44 | cg01191920 | chr7:158217561 | 0.54 |
| rs76344840 | chr9:33120204 | 2.97E-43 | cg20290983 | chr6:43655470 | 0.34 |
| rs12635522 | chr3:125708174 | 9.23E-38 | cg04553112 | chr3:125709451 | 0.06 |
| rs12635522 | chr3:125708174 | 1.68E-37 | cg02807482 | chr3:125708958 | 0.07 |
| rs4792919 | chr17:41873309 | 1.98E-37 | cg26893861 | chr17:41843967 | 0.71 |
| rs12635522 | chr3:125708174 | 2.29E-34 | cg06494592 | chr3:125709126 | 0.10 |
| rs10857676 | chr10:134970598 | 1.74E-33 | cg00753039 | chr10:134969141 | 0.29 |
| rs6680259 | chr1:7122308 | 2.50E-33 | cg20409752 | chr1:7122726 | 0.01 |
| rs10920265 | chr1:201822955 | 9.59E-33 | cg11586189 | chr1:201857591 | 0.25 |
| rs117194038 | chr17:43927290 | 5.30E-32 | cg22968622 | chr17:43663579 | 0.43 |
| rs907033 | chr16:83987856 | 1.14E-30 | cg27171569 | chr16:83987465 | 0.14 |
| rs11734838 | chr4:7980737 | 1.43E-30 | cg22688802 | chr4:7980661 | 0.51 |
| rs9933817 | chr16:83972660 | 3.25E-30 | cg16457916 | chr16:83968360 | 0.56 |
| rs4880509 | chr10:1511150 | 7.38E-29 | cg13684379 | chr10:1511173 | 0.66 |
| rs4880509 | chr10:1511150 | 1.71E-28 | cg09316607 | chr10:1511024 | 0.93 |
| rs2800973 | chr22:19168616 | 2.02E-27 | cg02655711 | chr22:19163373 | 0.41 |
| rs60668498 | chr19:44642672 | 9.76E-26 | cg23489630 | chr19:44645078 | 0.92 |
| rs4880509 | chr10:1511150 | 1.48E-25 | cg04012681 | chr10:1511277 | 0.52 |
| rs13282161 | chr8:1705041 | 7.43E-25 | cg17694851 | chr8:1707553 | 0.07 |
| rs13065 | chr14:100996312 | 1.04E-23 | cg18516195 | chr14:101012996 | 0.86 |
| rs10271372 | chr7:157793023 | 1.56E-23 | cg12440927 | chr7:157791721 | 0.04 |
| rs4807546 | chr19:4182060 | 2.24E-23 | cg23999422 | chr19:4173466 | 0.64 |
| rs9348260 | chr6:170516123 | 3.49E-23 | cg22739554 | chr6:170500876 | 0.10 |
| rs7833924 | chr8:144996029 | 5.06E-23 | cg10276948 | chr8:144986694 | 0.06 |
| rs7833924 | chr8:144996029 | 7.35E-23 | cg09374673 | chr8:144986488 | 0.06 |
| rs9933817 | chr16:83972660 | 1.30E-22 | cg16528738 | chr16:83968260 | 0.94 |
| rs907033 | chr16:83987856 | 2.05E-22 | cg07978099 | chr16:83986941 | 0.24 |
| rs2412322 | chr17:48578726 | 3.11E-22 | cg00901687 | chr17:48585270 | 0.06 |
| rs4689604 | chr4:7129556 | 1.06E-21 | cg16307866 | chr4:7129517 | 0.19 |
| rs7496866 | chr15:27102200 | 1.31E-21 | cg10318222 | chr15:27111940 | 0.20 |
| rs6435711 | chr2:213410065 | 1.54E-21 | cg16329650 | chr2:213403929 | 0.78 |
| rs13147452 | chr4:1078124 | 3.25E-21 | cg27284194 | chr4:1044797 | 0.60 |
| rs1156782 | chr6:115995564 | 6.21E-21 | cg04193905 | chr6:115989126 | 0.76 |
| rs2568198 | chr2:85403546 | 8.25E-21 | cg22128724 | chr2:85402928 | 0.78 |
| rs4928043 | chr3:54085900 | 9.48E-21 | cg15798837 | chr3:54122146 | 0.48 |
| rs10058772 | chr5:180033292 | 1.31E-20 | cg06967124 | chr5:180045597 | 0.52 |
| rs5412 | chr17:7184046 | 1.37E-20 | cg01757206 | chr17:7183913 | 0.18 |
| rs30927 | chr16:55317598 | 1.54E-20 | cg01064265 | chr16:55363058 | 0.11 |
| rs13065 | chr14:100996312 | 1.67E-20 | cg19590140 | chr14:101012492 | 0.81 |
| rs7833924 | chr8:144996029 | 2.76E-20 | cg06045337 | chr8:145013910 | 0.01 |
| rs2412322 | chr17:48578726 | 3.49E-20 | cg11440486 | chr17:48585216 | 0.08 |
| rs212781 | chr6:133771248 | 4.13E-20 | cg25075347 | chr6:133731801 | 0.53 |
| rs1735173 | chr8:146071261 | 4.84E-20 | cg20672363 | chr8:146075022 | 0.90 |
| rs10079713 | chr5:28692468 | 6.95E-20 | cg07881623 | chr6:80731107 | 0.39 |
| rs13147452 | chr4:1078124 | 9.41E-20 | cg04106633 | chr4:1044584 | 0.52 |
| rs16961809 | chr19:29226299 | 9.75E-20 | cg12756686 | chr19:29218302 | 0.26 |
| rs7516453 | chr1:15972558 | 1.52E-19 | cg17385448 | chr1:15911702 | 0.90 |
| rs1350543 | chr4:56014389 | 2.10E-19 | cg01777861 | chr4:56023843 | 0.79 |
| rs454759 | chr12:125799159 | 2.43E-19 | cg03923277 | chr12:104359732 | 0.18 |
| rs11650633 | chr17:80083807 | 2.92E-19 | cg16920238 | chr17:80076378 | 0.56 |
| rs57550038 | chr17:14078445 | 3.24E-19 | cg00902417 | chr17:15492168 | 0.25 |
| rs4807546 | chr19:4182060 | 3.25E-19 | cg01287132 | chr19:4173254 | 0.97 |
| rs2978902 | chr8:6690173 | 6.20E-19 | cg11878365 | chr8:6692387 | 0.24 |
| rs7201047 | chr16:86335556 | 6.38E-19 | cg04352168 | chr16:86334058 | 0.47 |
| rs1341741 | chr10:888073 | 6.63E-19 | cg26597838 | chr10:835615 | 0.45 |
| rs1341741 | chr10:888073 | 8.95E-19 | cg20503657 | chr10:835505 | 0.76 |
| rs16961809 | chr19:29226299 | 9.06E-19 | cg14983838 | chr19:29218262 | 0.11 |
| rs11636395 | chr15:45535684 | 1.04E-18 | cg25801113 | chr15:45476975 | 0.58 |
| rs9876131 | chr3:136726790 | 1.34E-18 | cg21827317 | chr3:136751795 | 0.27 |
| rs16961809 | chr19:29226299 | 1.90E-18 | cg03161606 | chr19:29218774 | 0.19 |
| rs11984421 | chr7:100266633 | 2.09E-18 | cg02938413 | chr7:100330587 | 0.24 |
| rs1350543 | chr4:56014389 | 2.39E-18 | cg09978860 | chr4:56023921 | 0.76 |
| rs12924275 | chr16:9191790 | 2.77E-18 | cg08831531 | chr16:9218945 | 0.08 |
| rs9936140 | chr16:10193417 | 2.88E-18 | cg08242859 | chr7:128032651 | 0.63 |
| rs56340588 | chr7:127799341 | 3.00E-18 | cg02301128 | chr7:127792165 | 0.74 |
| rs2062480 | chr1:197905400 | 3.12E-18 | cg00114966 | chr1:197893920 | 0.49 |
| rs74002504 | chr2:241828338 | 3.34E-18 | cg04034577 | chr2:241836375 | 0.10 |
| rs9643305 | chr8:134610055 | 4.90E-18 | cg22582999 | chr8:134594669 | 0.54 |
| rs8082590 | chr17:17958402 | 5.31E-18 | cg04398451 | chr17:18023971 | 0.86 |
| rs12097673 | chr1:2843180 | 5.61E-18 | cg21402748 | chr1:2838849 | 0.03 |
| rs72732566 | chr5:36744234 | 7.70E-18 | cg03995615 | chr5:36744219 | 0.54 |
| rs7803698 | chr7:64427895 | 8.13E-18 | cg12143784 | chr7:64541923 | 0.29 |
| rs9769809 | chr7:64956889 | 1.06E-17 | cg12143784 | chr7:64541923 | 0.29 |
| rs3128778 | chr4:2402474 | 1.07E-17 | cg01601518 | chr4:2404284 | 0.86 |
| rs7512217 | chr1:15586878 | 1.13E-17 | cg08815479 | chr1:15573596 | 0.93 |
| rs79175383 | chr12:96336533 | 1.49E-17 | cg25229172 | chr12:96336121 | 0.31 |
| rs7206985 | chr17:9159202 | 1.57E-17 | cg12361772 | chr17:9160821 | 0.88 |
| rs2800973 | chr22:19168616 | 1.61E-17 | cg24911827 | chr22:19170109 | 0.96 |
| rs2342082 | chr12:123090380 | 1.85E-17 | cg23029597 | chr12:123009494 | 0.39 |
| rs6565516 | chr17:78965146 | 1.89E-17 | cg10070101 | chr17:78963290 | 0.60 |
| rs6685767 | chr1:46823929 | 2.25E-17 | cg15580309 | chr1:46814106 | 0.45 |
| rs7257916 | chr19:45482884 | 2.38E-17 | cg13119609 | chr19:45449297 | 0.72 |
| rs2505949 | chr6:80804414 | 2.48E-17 | cg08355045 | chr6:80787529 | 0.87 |
| rs11675057 | chr2:26399481 | 2.89E-17 | cg22920501 | chr2:26401640 | 0.78 |
| rs11688491 | chr2:98167020 | 3.06E-17 | cg26665480 | chr2:98280029 | 0.58 |
| rs4555948 | chr6:160094492 | 3.17E-17 | cg13221458 | chr6:160112632 | 0.64 |
| rs7257916 | chr19:45482884 | 3.24E-17 | cg09555818 | chr19:45449301 | 0.81 |
| rs7470675 | chr9:132588337 | 3.47E-17 | cg13529314 | chr9:132598258 | 0.88 |
| rs74002504 | chr2:241828338 | 3.63E-17 | cg07537917 | chr2:241836409 | 0.49 |
| rs6676743 | chr1:110296338 | 4.07E-17 | cg10807101 | chr1:110282274 | 0.16 |
| rs56378923 | chr10:72316604 | 4.21E-17 | cg22643110 | chr10:72319345 | 0.74 |
| rs908951 | chr16:89697625 | 4.97E-17 | cg08949735 | chr16:89699720 | 0.74 |
| rs2246207 | chr17:61987576 | 5.00E-17 | cg06873352 | chr17:61820015 | 0.61 |
| rs72635991 | chr10:788658 | 5.00E-17 | cg24280607 | chr15:41709584 | 0.40 |
| rs62014776 | chr16:1339750 | 5.51E-17 | cg03705235 | chr16:1371463 | 0.63 |
| rs7496866 | chr15:27102200 | 5.62E-17 | cg03325535 | chr15:27111949 | 0.37 |
| rs13147452 | chr4:1078124 | 7.37E-17 | cg21130718 | chr4:1044621 | 0.34 |
| rs930526 | chr17:6473353 | 7.39E-17 | cg23551722 | chr17:6546898 | 0.66 |
| rs60061503 | chr1:185364328 | 7.89E-17 | cg11066601 | chr1:185373486 | 0.58 |
| rs113935737 | chr15:65123276 | 8.14E-17 | cg25489524 | chr15:65127973 | 0.84 |
| rs9812936 | chr3:50043654 | 9.76E-17 | cg12257692 | chr3:49977190 | 0.96 |
| rs59462516 | chr12:1165012 | 1.32E-16 | cg07813377 | chr12:1192425 | 0.68 |
| rs12467950 | chr2:220275736 | 1.38E-16 | cg15015639 | chr2:220282977 | 0.80 |
| rs9348260 | chr6:170516123 | 1.85E-16 | cg21597487 | chr6:170500638 | 0.19 |
| rs1350543 | chr4:56014389 | 1.93E-16 | cg16572876 | chr4:56024045 | 0.93 |
| rs4929922 | chr11:8975776 | 2.09E-16 | cg21881798 | chr11:8931708 | 0.00 |
| rs12595938 | chr16:8958081 | 2.14E-16 | cg08308162 | chr16:8889244 | 0.80 |
| rs28839814 | chr4:13555899 | 2.63E-16 | cg20477448 | chr4:13656704 | 0.38 |
| rs1730794 | chr4:3451960 | 3.04E-16 | cg25120210 | chr4:3464653 | 0.54 |
| rs9726753 | chr1:153591652 | 3.13E-16 | cg05659314 | chr1:153590020 | 0.10 |
| rs3021270 | chr22:40396409 | 3.35E-16 | cg21771250 | chr22:40406049 | 0.93 |
| rs6968990 | chr7:32552159 | 3.51E-16 | cg06627557 | chr7:32535165 | 0.31 |
| rs62185165 | chr2:241251453 | 3.62E-16 | cg21947394 | chr2:241260110 | 0.35 |
| rs1375813 | chr3:142809577 | 4.50E-16 | cg01520402 | chr3:142790398 | 0.31 |
| rs9812936 | chr3:50043654 | 4.87E-16 | cg05623727 | chr3:50126028 | 0.56 |
| rs12653391 | chr5:439796 | 4.91E-16 | cg00049323 | chr5:472564 | 0.34 |
| rs58635565 | chr8:41561181 | 5.08E-16 | cg07533533 | chr8:41559593 | 0.92 |
| rs12691433 | chr7:154989404 | 5.13E-16 | cg07944445 | chr7:155006275 | 0.85 |
| rs13283037 | chr9:97018520 | 5.44E-16 | cg13980266 | chr9:97022269 | 0.78 |
| rs117194038 | chr17:43927290 | 5.52E-16 | cg01934064 | chr17:44064242 | 0.73 |
| rs10793287 | chr11:77846706 | 6.02E-16 | cg09721595 | chr11:77773924 | 0.88 |
| rs1812214 | chr8:105344070 | 7.22E-16 | cg04554929 | chr8:105342491 | 0.73 |
| rs9769809 | chr7:64956889 | 7.61E-16 | cg01136167 | chr7:65037704 | 0.55 |
| rs2370234 | chr1:25304464 | 7.89E-16 | cg23273869 | chr1:25296894 | 0.47 |
| rs7662812 | chr4:24983413 | 8.17E-16 | cg19676182 | chr4:24981695 | 0.14 |
| rs7402982 | chr15:99193269 | 8.24E-16 | cg03437748 | chr15:99193247 | 0.03 |
| rs12022839 | chr1:58089133 | 8.59E-16 | cg00026909 | chr1:58089001 | 0.63 |
| rs11149799 | chr16:75174049 | 8.71E-16 | cg00897404 | chr16:75182368 | 0.77 |
| rs7514450 | chr1:220991171 | 8.91E-16 | cg08655206 | chr1:221058198 | 0.34 |
| rs4782336 | chr16:89151518 | 1.01E-15 | cg00697672 | chr16:89151343 | 0.63 |
| rs45537633 | chr6:41117824 | 1.18E-15 | cg03644281 | chr6:41068752 | 0.23 |
| rs11060115 | chr12:129554644 | 1.19E-15 | cg01290755 | chr12:129554587 | 0.17 |
| rs4789846 | chr17:80225545 | 1.29E-15 | cg22805688 | chr17:80197841 | 0.84 |
| rs7574691 | chr2:240044687 | 1.30E-15 | cg15934368 | chr2:240044021 | 0.07 |
| rs11712066 | chr3:151830309 | 1.48E-15 | cg27098685 | chr3:151867537 | 0.85 |
| rs30927 | chr16:55317598 | 1.65E-15 | cg07592723 | chr16:55365146 | 0.32 |
| rs6597862 | chr10:126649723 | 1.69E-15 | cg06432487 | chr10:126623651 | 0.54 |
| rs9817966 | chr3:46650540 | 1.78E-15 | cg24524379 | chr3:46600244 | 0.16 |
| rs12674529 | chr8:127885599 | 1.82E-15 | cg21238284 | chr8:127889295 | 0.78 |
| rs455104 | chr17:10618227 | 1.89E-15 | cg00549475 | chr17:10632715 | 0.44 |
| rs30927 | chr16:55317598 | 2.12E-15 | cg06722193 | chr16:55359355 | 0.17 |
| rs2276968 | chr4:7070118 | 2.26E-15 | cg06697600 | chr4:7070879 | 0.55 |
| rs4571937 | chr1:20897033 | 2.30E-15 | cg00750606 | chr1:20899121 | 0.85 |
| rs4770476 | chr13:24269820 | 2.35E-15 | cg07031408 | chr13:24269867 | 0.51 |
| rs57094537 | chr4:40261798 | 2.39E-15 | cg25243082 | chr4:40267141 | 0.83 |
| rs7016869 | chr8:2474928 | 2.58E-15 | cg02472801 | chr8:2480483 | 0.18 |
| rs2637657 | chr10:133993806 | 2.60E-15 | cg18037376 | chr10:134004552 | 0.52 |
| rs11675057 | chr2:26399481 | 2.70E-15 | cg25036284 | chr2:26402008 | 0.81 |
| rs1919784 | chr7:33105268 | 2.89E-15 | cg22798885 | chr7:33102694 | 0.74 |
| rs7663448 | chr4:10054787 | 2.91E-15 | cg26043149 | chr18:55253948 | 0.29 |
| rs10987908 | chr9:130936530 | 3.07E-15 | cg10071929 | chr9:130955135 | 0.72 |
| rs392124 | chr17:9669921 | 3.17E-15 | cg13468767 | chr17:9672024 | 0.26 |
| rs13147452 | chr4:1078124 | 3.59E-15 | cg04016957 | chr4:1044486 | 0.42 |
| rs3793202 | chr7:6207142 | 4.84E-15 | cg22849526 | chr7:6199437 | 0.82 |
| rs6711715 | chr2:130330516 | 5.25E-15 | cg05903289 | chr2:130345205 | 0.06 |
| rs11179581 | chr12:37892432 | 5.32E-15 | cg10856724 | chr12:34555212 | 0.18 |
| rs117194038 | chr17:43927290 | 5.58E-15 | cg01341218 | chr17:43662625 | 0.46 |
| rs7104785 | chr11:804212 | 5.81E-15 | cg01741372 | chr11:783889 | 0.93 |
| rs9284725 | chr2:102744854 | 5.95E-15 | cg22835712 | chr2:102737379 | 0.71 |
| rs7833924 | chr8:144996029 | 5.96E-15 | cg27082292 | chr8:145001361 | 0.26 |
| rs11675057 | chr2:26399481 | 6.22E-15 | cg27170947 | chr2:26402098 | 0.50 |
| rs4314559 | chr7:2365892 | 6.93E-15 | cg16553052 | chr7:2349605 | 0.67 |
| rs12028536 | chr1:228743706 | 7.56E-15 | cg06261630 | chr1:228741351 | 0.74 |
| rs4910458 | chr11:9379402 | 8.41E-15 | cg19695805 | chr11:9385645 | 0.96 |
| rs7496866 | chr15:27102200 | 8.56E-15 | cg01378667 | chr15:27111911 | 0.44 |
| rs257701 | chr5:123737922 | 8.80E-15 | cg01806427 | chr5:123737813 | 0.76 |
| rs13044479 | chr20:62245686 | 8.94E-15 | cg09650180 | chr20:62225654 | 0.74 |
| rs7833924 | chr8:144996029 | 9.08E-15 | cg05696706 | chr8:145013932 | 0.01 |
| rs10987908 | chr9:130936530 | 9.61E-15 | cg09976142 | chr9:130955436 | 0.58 |
| rs4770476 | chr13:24269820 | 9.62E-15 | cg10885151 | chr13:24270087 | 0.57 |
| rs12129745 | chr1:28572317 | 1.07E-14 | cg04993605 | chr1:28573052 | 0.23 |
| rs34228916 | chr12:115942842 | 1.14E-14 | cg18639984 | chr12:115943877 | 0.11 |
| rs8016689 | chr14:103319981 | 1.21E-14 | cg23020514 | chr14:103360112 | 0.09 |
| rs8008761 | chr14:90739754 | 1.29E-14 | cg10090757 | chr14:90744615 | 0.91 |
| rs4782336 | chr16:89151518 | 1.30E-14 | cg07845093 | chr16:89151447 | 0.88 |
| rs2269906 | chr17:42294337 | 1.34E-14 | cg13607699 | chr17:42295918 | 0.43 |
| rs10175462 | chr2:113988492 | 1.41E-14 | cg21550016 | chr2:113992930 | 0.51 |
| rs72781258 | chr2:20274213 | 1.73E-14 | cg24657347 | chr2:20261756 | 0.94 |
| rs8057544 | chr16:537650 | 1.96E-14 | cg27494100 | chr16:537705 | 0.82 |
| rs72755098 | chr15:64427587 | 2.09E-14 | cg02848875 | chr15:64387786 | 0.28 |
| rs9998888 | chr4:824575 | 2.10E-14 | cg24793722 | chr4:824416 | 0.70 |
| rs193930 | chr2:8107520 | 2.21E-14 | cg03155496 | chr2:8117019 | 0.46 |
| rs8053397 | chr16:87573468 | 2.32E-14 | cg08031982 | chr16:87577539 | 0.18 |
| rs4984688 | chr16:785717 | 2.32E-14 | cg18653534 | chr16:772142 | 0.37 |
| rs117194038 | chr17:43927290 | 2.36E-14 | cg22433210 | chr17:43662623 | 0.82 |
| rs7143780 | chr14:96154609 | 2.44E-14 | cg03043804 | chr14:96152706 | 0.49 |
| rs9782 | chr12:103351826 | 2.56E-14 | cg27569040 | chr12:103351855 | 0.86 |
| rs9915323 | chr17:37770481 | 2.59E-14 | cg00129232 | chr17:37814104 | 0.92 |
| rs17333520 | chr11:31058834 | 2.60E-14 | cg26647111 | chr11:31128758 | 0.09 |
| rs111543213 | chr19:37642385 | 2.61E-14 | cg08835041 | chr19:37461278 | 0.16 |
| rs9912302 | chr17:44916982 | 2.75E-14 | cg25836567 | chr17:44929689 | 0.16 |
| rs10920265 | chr1:201822955 | 2.87E-14 | cg06775570 | chr1:201857621 | 0.25 |
| rs2376584 | chr17:76402116 | 3.21E-14 | cg05887092 | chr17:76393375 | 0.46 |
| rs16961809 | chr19:29226299 | 3.22E-14 | cg25267487 | chr19:29217858 | 0.12 |
| rs3128778 | chr4:2402474 | 3.27E-14 | cg13053151 | chr4:2403559 | 0.59 |
| rs3808524 | chr8:23161983 | 3.42E-14 | cg24531534 | chr8:23162162 | 0.53 |
| rs10773762 | chr12:130765366 | 3.60E-14 | cg14604444 | chr12:130766091 | 0.48 |
| rs12977777 | chr19:35323365 | 3.64E-14 | cg15695738 | chr19:35329860 | 0.13 |
| rs11230570 | chr11:60782634 | 3.68E-14 | cg27098804 | chr11:60776124 | 0.45 |
| rs4866663 | chr5:2727471 | 4.15E-14 | cg27345924 | chr5:2727758 | 0.91 |
| rs6560691 | chr10:134140245 | 4.24E-14 | cg23771949 | chr10:134165390 | 0.05 |
| rs7015233 | chr8:144381511 | 5.46E-14 | cg12888521 | chr8:144346762 | 0.42 |
| rs10151225 | chr14:52589352 | 5.59E-14 | cg12071775 | chr14:52591786 | 0.25 |
| rs28520336 | chr6:17555977 | 5.81E-14 | cg09879382 | chr6:17581248 | 0.93 |
| rs10492997 | chr1:19769371 | 6.19E-14 | cg17081867 | chr1:19768096 | 0.25 |
| rs7016869 | chr8:2474928 | 6.30E-14 | cg01414268 | chr8:2480911 | 0.20 |
| rs10987908 | chr9:130936530 | 6.90E-14 | cg13642260 | chr9:130955380 | 0.63 |
| rs10773762 | chr12:130765366 | 7.12E-14 | cg27633287 | chr12:130766243 | 0.44 |
| rs2205661 | chr22:45731759 | 7.17E-14 | cg00733150 | chr22:45705707 | 0.12 |
| rs12698058 | chr7:157225536 | 7.24E-14 | cg03453431 | chr7:157225567 | 0.44 |
| rs10175462 | chr2:113988492 | 7.76E-14 | cg11763394 | chr2:113992921 | 0.47 |
| rs4143866 | chr16:85716366 | 7.96E-14 | cg26571870 | chr16:85723150 | 0.54 |
| rs7455225 | chr7:73241386 | 9.64E-14 | cg02874145 | chr7:73246406 | 0.32 |
| rs10888514 | chr1:152707929 | 9.78E-14 | cg07796016 | chr1:152779584 | 0.98 |
| rs4475020 | chr3:14599121 | 1.10E-13 | cg21529591 | chr3:14596904 | 0.32 |
| rs6421977 | chr11:407708 | 1.12E-13 | cg18351999 | chr11:406901 | 0.52 |
| rs4965672 | chr15:101089241 | 1.20E-13 | cg02597199 | chr15:101098829 | 0.77 |
| rs6711715 | chr2:130330516 | 1.21E-13 | cg05962382 | chr2:130345044 | 0.71 |
| rs2021560 | chr19:22373470 | 1.23E-13 | cg22620746 | chr19:22234992 | 0.44 |
| rs4789885 | chr17:77310495 | 1.30E-13 | cg08223357 | chr17:77302162 | 0.79 |
| rs16965349 | chr17:36614524 | 1.32E-13 | cg12050358 | chr17:36612909 | 0.78 |
| rs12208321 | chr6:56132243 | 1.35E-13 | cg07143470 | chr6:56111812 | 0.10 |
| rs12097673 | chr1:2843180 | 1.35E-13 | cg00996827 | chr1:2838805 | 0.14 |
| rs3793969 | chr11:1318884 | 1.36E-13 | cg14329644 | chr11:1253904 | 0.73 |
| rs4900242 | chr14:95151985 | 1.45E-13 | cg16462006 | chr14:95155784 | 0.42 |
| rs58069944 | chr8:2294105 | 1.46E-13 | cg11900328 | chr8:2263331 | 0.99 |
| rs2297475 | chr10:90984623 | 1.55E-13 | cg17741809 | chr10:90985055 | 0.44 |
| rs29655 | chr5:180267436 | 1.61E-13 | cg19091830 | chr5:180286094 | 0.21 |
| rs6946060 | chr7:157644761 | 1.67E-13 | cg22216157 | chr7:157643037 | 0.82 |
| rs6946060 | chr7:157644761 | 1.74E-13 | cg25449441 | chr7:157644471 | 0.94 |
| rs4928043 | chr3:54085900 | 1.84E-13 | cg01296889 | chr3:54122032 | 0.22 |
| rs45537633 | chr6:41117824 | 1.87E-13 | cg04346459 | chr6:41068666 | 0.74 |
| rs28400431 | chr8:143698404 | 1.87E-13 | cg10104451 | chr8:143696006 | 0.68 |
| rs72704639 | chr1:150798335 | 1.91E-13 | cg04414720 | chr1:150670196 | 0.07 |
| rs2453606 | chr17:19387091 | 1.92E-13 | cg19949948 | chr17:19361230 | 0.68 |
| rs28452050 | chr16:29317318 | 1.94E-13 | cg05645661 | chr16:29329490 | 0.77 |
| rs668338 | chr11:60713358 | 1.97E-13 | cg06257669 | chr11:60702218 | 0.43 |
| rs2870479 | chr19:57449293 | 2.03E-13 | cg12414181 | chr15:75287860 | 0.86 |
| rs7794450 | chr7:6625847 | 2.05E-13 | cg19149522 | chr7:6616423 | 0.13 |
| rs143349430 | chr10:32183019 | 2.18E-13 | cg04359828 | chr10:32216031 | 0.24 |
| rs12653230 | chr5:159735611 | 2.21E-13 | cg17267804 | chr5:159735392 | 0.07 |
| rs1055150 | chr19:18499784 | 2.34E-13 | cg21088460 | chr19:18499786 | 0.87 |
| rs9525735 | chr13:43661306 | 2.36E-13 | cg14729962 | chr13:43597565 | 0.00 |
| rs2145848 | chr6:3035894 | 2.56E-13 | cg05728019 | chr6:3024023 | 0.86 |
| rs890393 | chr18:74102435 | 2.60E-13 | cg24786174 | chr18:74118243 | 0.18 |
| rs11650633 | chr17:80083807 | 2.65E-13 | cg00755572 | chr17:80077754 | 0.36 |
| rs7018316 | chr8:144630169 | 2.85E-13 | cg20458811 | chr8:144631810 | 0.11 |
| rs11675057 | chr2:26399481 | 3.21E-13 | cg04944784 | chr2:26401820 | 0.76 |
| rs10058772 | chr5:180033292 | 3.26E-13 | cg00744924 | chr5:180042471 | 0.72 |
| rs75934331 | chr1:183182029 | 3.30E-13 | cg01417625 | chr1:183187433 | 0.48 |
| rs5023799 | chr13:47095293 | 3.55E-13 | cg11342437 | chr13:47126328 | 0.03 |
| rs12761857 | chr10:7105868 | 3.56E-13 | cg00998146 | chr19:18284560 | 0.79 |

**Supplementary Table 19: Methylation QTLs (mQTLs) identified in the striatum (STR) for SNPs included in the schizophrenia polygenic risk score.** Shown are associations between DNA methylation at specific Illumina 450K probes and genetic variants used to derive polygenic risk score (PRS) in our study, in addition to the corresponding *P*-values for PRS-associated DNA methylation variation at the same probe.

| **SNP** | **SNP genomic location (hg19)** | **mQTL *P*** | **CpG** | **CpG genomic location (hg19)** | **EWAS *P*** |
| --- | --- | --- | --- | --- | --- |
| rs6680259 | chr1:7122308 | 1.55E-52 | cg20409752 | chr1:7122726 | 0.07 |
| rs9726753 | chr1:153591652 | 3.54E-50 | cg08477332 | chr1:153590243 | 0.29 |
| rs12635522 | chr3:125708174 | 5.95E-46 | cg15145296 | chr3:125709740 | 0.61 |
| rs117194038 | chr17:43927290 | 2.87E-45 | cg17117718 | chr17:43663208 | 0.50 |
| rs76344840 | chr9:33120204 | 4.29E-43 | cg20290983 | chr6:43655470 | 0.38 |
| rs4792919 | chr17:41873309 | 1.00E-39 | cg26893861 | chr17:41843967 | 0.99 |
| rs35059736 | chr7:158224692 | 2.93E-34 | cg01191920 | chr7:158217561 | 0.58 |
| rs12635522 | chr3:125708174 | 3.18E-34 | cg02807482 | chr3:125708958 | 0.59 |
| rs12635522 | chr3:125708174 | 5.04E-34 | cg06494592 | chr3:125709126 | 0.95 |
| rs9348260 | chr6:170516123 | 1.16E-31 | cg22739554 | chr6:170500876 | 0.03 |
| rs12635522 | chr3:125708174 | 1.92E-31 | cg04553112 | chr3:125709451 | 0.43 |
| rs117194038 | chr17:43927290 | 3.19E-31 | cg22968622 | chr17:43663579 | 0.31 |
| rs7833924 | chr8:144996029 | 5.67E-31 | cg10276948 | chr8:144986694 | 0.02 |
| rs907033 | chr16:83987856 | 9.19E-31 | cg27171569 | chr16:83987465 | 0.29 |
| rs4880509 | chr10:1511150 | 1.74E-30 | cg09316607 | chr10:1511024 | 0.71 |
| rs72732566 | chr5:36744234 | 1.80E-30 | cg03995615 | chr5:36744219 | 0.95 |
| rs4880509 | chr10:1511150 | 4.08E-30 | cg13684379 | chr10:1511173 | 0.49 |
| rs4807546 | chr19:4182060 | 2.74E-28 | cg23999422 | chr19:4173466 | 0.76 |
| rs12653230 | chr5:159735611 | 1.13E-27 | cg17267804 | chr5:159735392 | 0.02 |
| rs10857676 | chr10:134970598 | 1.43E-27 | cg00753039 | chr10:134969141 | 0.54 |
| rs9933817 | chr16:83972660 | 1.46E-27 | cg16528738 | chr16:83968260 | 0.36 |
| rs11650633 | chr17:80083807 | 2.21E-26 | cg16920238 | chr17:80076378 | 0.63 |
| rs6597862 | chr10:126649723 | 3.79E-26 | cg06432487 | chr10:126623651 | 0.92 |
| rs4880509 | chr10:1511150 | 3.79E-26 | cg04012681 | chr10:1511277 | 0.71 |
| rs7833924 | chr8:144996029 | 6.96E-26 | cg09374673 | chr8:144986488 | 0.05 |
| rs199503 | chr17:44862162 | 2.97E-25 | cg22968622 | chr17:43663579 | 0.31 |
| rs11636395 | chr15:45535684 | 4.47E-25 | cg25801113 | chr15:45476975 | 0.04 |
| rs117194038 | chr17:43927290 | 5.23E-25 | cg01934064 | chr17:44064242 | 0.84 |
| rs4807546 | chr19:4182060 | 7.48E-25 | cg01287132 | chr19:4173254 | 0.62 |
| rs9348260 | chr6:170516123 | 1.05E-24 | cg21597487 | chr6:170500638 | 0.05 |
| rs9348260 | chr6:170516123 | 1.14E-24 | cg21235075 | chr6:170500610 | 0.20 |
| rs11060115 | chr12:129554644 | 6.89E-24 | cg01290755 | chr12:129554587 | 0.30 |
| rs199503 | chr17:44862162 | 8.32E-24 | cg17117718 | chr17:43663208 | 0.50 |
| rs2412322 | chr17:48578726 | 8.83E-24 | cg00901687 | chr17:48585270 | 0.09 |
| rs2505949 | chr6:80804414 | 1.55E-23 | cg08355045 | chr6:80787529 | 0.47 |
| rs13065 | chr14:100996312 | 1.68E-23 | cg18516195 | chr14:101012996 | 0.12 |
| rs2800973 | chr22:19168616 | 4.34E-23 | cg02655711 | chr22:19163373 | 0.56 |
| rs4555948 | chr6:160094492 | 5.15E-23 | cg13221458 | chr6:160112632 | 0.26 |
| rs5412 | chr17:7184046 | 5.65E-23 | cg01757206 | chr17:7183913 | 0.53 |
| rs6455887 | chr6:163674175 | 6.75E-23 | cg07343445 | chr6:163673130 | 0.11 |
| rs73752005 | chr6:88092421 | 9.34E-23 | cg06087457 | chr6:88040249 | 0.41 |
| rs56340588 | chr7:127799341 | 2.61E-22 | cg02301128 | chr7:127792165 | 0.54 |
| rs9933817 | chr16:83972660 | 2.70E-22 | cg16457916 | chr16:83968360 | 0.80 |
| rs13147452 | chr4:1078124 | 7.13E-22 | cg27284194 | chr4:1044797 | 0.59 |
| rs72755098 | chr15:64427587 | 1.99E-21 | cg02848875 | chr15:64387786 | 0.32 |
| rs74002504 | chr2:241828338 | 2.82E-21 | cg04034577 | chr2:241836375 | 0.33 |
| rs1350543 | chr4:56014389 | 4.64E-21 | cg09978860 | chr4:56023921 | 0.97 |
| rs60061503 | chr1:185364328 | 7.87E-21 | cg11066601 | chr1:185373486 | 0.85 |
| rs454759 | chr12:125799159 | 9.47E-21 | cg03923277 | chr12:104359732 | 0.40 |
| rs1350543 | chr4:56014389 | 1.43E-20 | cg01777861 | chr4:56023843 | 0.81 |
| rs257701 | chr5:123737922 | 1.59E-20 | cg01806427 | chr5:123737813 | 0.26 |
| rs7131362 | chr11:1689325 | 3.18E-20 | cg04938738 | chr11:1689429 | 0.62 |
| rs60668498 | chr19:44642672 | 3.93E-20 | cg23489630 | chr19:44645078 | 0.87 |
| rs12595938 | chr16:8958081 | 6.21E-20 | cg08308162 | chr16:8889244 | 0.39 |
| rs28839814 | chr4:13555899 | 8.95E-20 | cg20477448 | chr4:13656704 | 0.82 |
| rs28452050 | chr16:29317318 | 1.09E-19 | cg05645661 | chr16:29329490 | 0.32 |
| rs35037013 | chr10:54656418 | 1.12E-19 | cg05984115 | chr10:54631212 | 0.92 |
| rs2291393 | chr17:80585094 | 1.13E-19 | cg27136344 | chr17:80606911 | 0.54 |
| rs16961809 | chr19:29226299 | 1.20E-19 | cg14983838 | chr19:29218262 | 0.18 |
| rs11230233 | chr11:55349858 | 1.27E-19 | cg20623702 | chr11:55431584 | 0.73 |
| rs650241 | chr11:75277757 | 1.71E-19 | cg26104986 | chr11:75275303 | 0.08 |
| rs4143866 | chr16:85716366 | 1.92E-19 | cg26571870 | chr16:85723150 | 0.68 |
| rs6685767 | chr1:46823929 | 2.97E-19 | cg15580309 | chr1:46814106 | 0.12 |
| rs8082590 | chr17:17958402 | 3.07E-19 | cg04398451 | chr17:18023971 | 0.56 |
| rs1341741 | chr10:888073 | 4.50E-19 | cg26597838 | chr10:835615 | 0.94 |
| rs2412322 | chr17:48578726 | 4.74E-19 | cg11440486 | chr17:48585216 | 0.11 |
| rs13147452 | chr4:1078124 | 5.33E-19 | cg21130718 | chr4:1044621 | 0.74 |
| rs12097673 | chr1:2843180 | 5.33E-19 | cg21402748 | chr1:2838849 | 0.84 |
| rs10793287 | chr11:77846706 | 5.40E-19 | cg09721595 | chr11:77773924 | 0.73 |
| rs1294417 | chr6:6741932 | 5.59E-19 | cg06612196 | chr6:6737390 | 0.81 |
| rs7803698 | chr7:64427895 | 7.87E-19 | cg12143784 | chr7:64541923 | 0.13 |
| rs212781 | chr6:133771248 | 1.12E-18 | cg25075347 | chr6:133731801 | 0.69 |
| rs62014776 | chr16:1339750 | 1.21E-18 | cg03705235 | chr16:1371463 | 0.60 |
| rs9726753 | chr1:153591652 | 1.23E-18 | cg05659314 | chr1:153590020 | 0.69 |
| rs6565516 | chr17:78965146 | 1.41E-18 | cg10070101 | chr17:78963290 | 0.63 |
| rs2066700 | chr13:50887725 | 1.58E-18 | cg17976839 | chr13:50923823 | 0.06 |
| rs11675057 | chr2:26399481 | 1.61E-18 | cg22920501 | chr2:26401640 | 0.74 |
| rs4689604 | chr4:7129556 | 1.69E-18 | cg16307866 | chr4:7129517 | 0.88 |
| rs16961809 | chr19:29226299 | 1.88E-18 | cg03161606 | chr19:29218774 | 0.14 |
| rs2342082 | chr12:123090380 | 2.10E-18 | cg23029597 | chr12:123009494 | 0.09 |
| rs13282161 | chr8:1705041 | 2.29E-18 | cg17694851 | chr8:1707553 | 0.05 |
| rs684337 | chr17:727349 | 2.30E-18 | cg05176970 | chr17:724273 | 0.72 |
| rs199503 | chr17:44862162 | 2.40E-18 | cg01934064 | chr17:44064242 | 0.84 |
| rs62185165 | chr2:241251453 | 2.58E-18 | cg21947394 | chr2:241260110 | 0.64 |
| rs3793202 | chr7:6207142 | 3.00E-18 | cg22849526 | chr7:6199437 | 0.24 |
| rs3891052 | chr14:105672895 | 3.86E-18 | cg27037305 | chr14:105689766 | 0.54 |
| rs16961809 | chr19:29226299 | 4.54E-18 | cg12756686 | chr19:29218302 | 0.21 |
| rs45537633 | chr6:41117824 | 4.90E-18 | cg03644281 | chr6:41068752 | 0.68 |
| rs4475020 | chr3:14599121 | 5.36E-18 | cg21529591 | chr3:14596904 | 0.61 |
| rs10058772 | chr5:180033292 | 5.48E-18 | cg06967124 | chr5:180045597 | 0.47 |
| rs13065 | chr14:100996312 | 5.57E-18 | cg19590140 | chr14:101012492 | 0.47 |
| rs9390343 | chr6:146010527 | 6.69E-18 | cg01476807 | chr6:146125657 | 0.94 |
| rs57550038 | chr17:14078445 | 7.18E-18 | cg00902417 | chr17:15492168 | 0.33 |
| rs7257916 | chr19:45482884 | 7.23E-18 | cg09555818 | chr19:45449301 | 0.65 |
| rs6801145 | chr3:167312146 | 8.54E-18 | cg18801567 | chr3:167450363 | 0.02 |
| rs7662812 | chr4:24983413 | 8.97E-18 | cg19676182 | chr4:24981695 | 1.00 |
| rs57094537 | chr4:40261798 | 9.01E-18 | cg25243082 | chr4:40267141 | 0.58 |
| rs392124 | chr17:9669921 | 1.16E-17 | cg13468767 | chr17:9672024 | 0.36 |
| rs4928043 | chr3:54085900 | 1.34E-17 | cg15798837 | chr3:54122146 | 0.37 |
| rs6435711 | chr2:213410065 | 2.36E-17 | cg16329650 | chr2:213403929 | 0.38 |
| rs2978902 | chr8:6690173 | 2.70E-17 | cg11878365 | chr8:6692387 | 0.77 |
| rs10897269 | chr11:62162088 | 2.70E-17 | cg23876832 | chr11:62092739 | 0.13 |
| rs9876131 | chr3:136726790 | 3.09E-17 | cg21827317 | chr3:136751795 | 0.82 |
| rs7015233 | chr8:144381511 | 3.83E-17 | cg20232550 | chr8:144344793 | 0.95 |
| rs890393 | chr18:74102435 | 4.51E-17 | cg24786174 | chr18:74118243 | 0.14 |
| rs66712530 | chr12:132664285 | 4.93E-17 | cg26320244 | chr12:132663455 | 0.44 |
| rs2568198 | chr2:85403546 | 5.14E-17 | cg22128724 | chr2:85402928 | 0.72 |
| rs144716806 | chr4:38854783 | 5.17E-17 | cg26681822 | chr4:38858561 | 0.38 |
| rs72704639 | chr1:150798335 | 5.97E-17 | cg09365446 | chr1:150670422 | 0.15 |
| rs2014453 | chr7:44142792 | 6.41E-17 | cg12399411 | chr7:44134432 | 0.59 |
| rs12214357 | chr6:77577369 | 7.43E-17 | cg17141972 | chr6:77547510 | 0.53 |
| rs13147452 | chr4:1078124 | 8.06E-17 | cg04106633 | chr4:1044584 | 0.58 |
| rs12698058 | chr7:157225536 | 9.93E-17 | cg03453431 | chr7:157225567 | 0.49 |
| rs9769809 | chr7:64956889 | 1.03E-16 | cg12143784 | chr7:64541923 | 0.13 |
| rs13283037 | chr9:97018520 | 1.09E-16 | cg13980266 | chr9:97022269 | 0.81 |
| rs10175462 | chr2:113988492 | 1.12E-16 | cg21550016 | chr2:113992930 | 0.99 |
| rs4910458 | chr11:9379402 | 1.16E-16 | cg19695805 | chr11:9385645 | 0.63 |
| rs9917823 | chr3:183673780 | 1.39E-16 | cg01324343 | chr3:183735012 | 0.06 |
| rs12129745 | chr1:28572317 | 1.48E-16 | cg04993605 | chr1:28573052 | 0.85 |
| rs4802745 | chr19:51320111 | 1.71E-16 | cg02725269 | chr19:51327177 | 0.35 |
| rs3128778 | chr4:2402474 | 1.78E-16 | cg13053151 | chr4:2403559 | 0.77 |
| rs2475509 | chr6:39890217 | 1.79E-16 | cg10871120 | chr6:39891273 | 0.07 |
| rs2062480 | chr1:197905400 | 1.85E-16 | cg00114966 | chr1:197893920 | 0.34 |
| rs12450494 | chr17:7207887 | 2.22E-16 | cg04514024 | chr17:7222668 | 0.31 |
| rs7833924 | chr8:144996029 | 2.22E-16 | cg06045337 | chr8:145013910 | 0.16 |
| rs10175462 | chr2:113988492 | 2.53E-16 | cg11763394 | chr2:113992921 | 0.88 |
| rs28400431 | chr8:143698404 | 2.54E-16 | cg10104451 | chr8:143696006 | 0.25 |
| rs4984688 | chr16:785717 | 2.65E-16 | cg18653534 | chr16:772142 | 0.46 |
| rs4571937 | chr1:20897033 | 2.83E-16 | cg00750606 | chr1:20899121 | 0.91 |
| rs9936140 | chr16:10193417 | 2.84E-16 | cg08242859 | chr7:128032651 | 0.41 |
| rs13147452 | chr4:1078124 | 3.33E-16 | cg04016957 | chr4:1044486 | 0.50 |
| rs4789846 | chr17:80225545 | 3.71E-16 | cg22805688 | chr17:80197841 | 0.96 |
| rs1058167 | chr22:42538029 | 3.83E-16 | cg09322432 | chr22:42527611 | 0.00 |
| rs2553022 | chr7:100401862 | 3.90E-16 | cg12616177 | chr7:100434510 | 0.09 |
| rs12028536 | chr1:228743706 | 4.04E-16 | cg06261630 | chr1:228741351 | 0.51 |
| rs4965672 | chr15:101089241 | 4.41E-16 | cg02597199 | chr15:101098829 | 0.54 |
| rs72704639 | chr1:150798335 | 5.04E-16 | cg04414720 | chr1:150670196 | 0.44 |
| rs11650633 | chr17:80083807 | 5.82E-16 | cg00755572 | chr17:80077754 | 0.78 |
| rs10987908 | chr9:130936530 | 6.99E-16 | cg10071929 | chr9:130955135 | 0.67 |
| rs62120467 | chr2:3382624 | 7.07E-16 | cg12447832 | chr2:3383257 | 0.19 |
| rs9549822 | chr13:112691814 | 7.07E-16 | cg16875032 | chr8:121823916 | 0.01 |
| rs7104785 | chr11:804212 | 7.10E-16 | cg01741372 | chr11:783889 | 0.76 |
| rs274692 | chr5:6734625 | 7.52E-16 | cg10857441 | chr5:6722123 | 0.43 |
| rs9817966 | chr3:46650540 | 7.80E-16 | cg24524379 | chr3:46600244 | 0.38 |
| rs1341741 | chr10:888073 | 1.02E-15 | cg20503657 | chr10:835505 | 0.59 |
| rs111543213 | chr19:37642385 | 1.05E-15 | cg08835041 | chr19:37461278 | 0.45 |
| rs34228916 | chr12:115942842 | 1.16E-15 | cg18639984 | chr12:115943877 | 0.93 |
| rs4782336 | chr16:89151518 | 1.28E-15 | cg07845093 | chr16:89151447 | 0.60 |
| rs72781258 | chr2:20274213 | 1.33E-15 | cg24657347 | chr2:20261756 | 0.86 |
| rs7018316 | chr8:144630169 | 1.35E-15 | cg20458811 | chr8:144631810 | 0.95 |
| rs883138 | chr7:150042793 | 1.54E-15 | cg12556325 | chr7:150026731 | 0.54 |
| rs193572 | chr7:116835734 | 1.67E-15 | cg16444922 | chr7:116842338 | 0.42 |
| rs7514450 | chr1:220991171 | 1.68E-15 | cg15450098 | chr1:221057561 | 0.05 |
| rs10753541 | chr1:23061992 | 1.92E-15 | cg05266663 | chr1:23061564 | 0.54 |
| rs28701975 | chr1:38272307 | 2.06E-15 | cg00095214 | chr1:38272200 | 0.44 |
| rs907033 | chr16:83987856 | 2.70E-15 | cg07978099 | chr16:83986941 | 0.39 |
| rs7854075 | chr9:96571211 | 2.70E-15 | cg14396892 | chr9:96623032 | 0.11 |
| rs10175462 | chr2:113988492 | 3.31E-15 | cg07772999 | chr2:113993052 | 0.96 |
| rs2370234 | chr1:25304464 | 3.33E-15 | cg23273869 | chr1:25296894 | 0.05 |
| rs6517397 | chr21:38352125 | 3.52E-15 | cg21871091 | chr21:38349937 | 0.66 |
| rs2205661 | chr22:45731759 | 3.64E-15 | cg00733150 | chr22:45705707 | 0.07 |
| rs12022839 | chr1:58089133 | 4.85E-15 | cg00026909 | chr1:58089001 | 0.50 |
| rs11675057 | chr2:26399481 | 4.93E-15 | cg04944784 | chr2:26401820 | 0.54 |
| rs10079713 | chr5:28692468 | 5.93E-15 | cg07881623 | chr6:80731107 | 0.58 |
| rs1350543 | chr4:56014389 | 6.00E-15 | cg16572876 | chr4:56024045 | 0.94 |
| rs1571624 | chr13:112164791 | 6.50E-15 | cg25943066 | chr13:112164965 | 0.93 |
| rs1183079 | chr7:2904672 | 6.75E-15 | cg14668632 | chr7:2872130 | 0.97 |
| rs930526 | chr17:6473353 | 6.79E-15 | cg23551722 | chr17:6546898 | 0.87 |
| rs4648729 | chr1:1808769 | 6.96E-15 | cg03396347 | chr1:1875803 | 0.80 |
| rs8044982 | chr16:58521090 | 7.09E-15 | cg02036364 | chr16:58521443 | 0.75 |
| rs10987908 | chr9:130936530 | 7.42E-15 | cg13642260 | chr9:130955380 | 0.47 |
| rs4793213 | chr17:41307101 | 8.37E-15 | cg23758822 | chr17:41437982 | 0.16 |
| rs7663448 | chr4:10054787 | 8.99E-15 | cg15882809 | chr6:26285828 | 0.91 |
| rs11251278 | chr10:2544043 | 9.11E-15 | cg18171855 | chr10:2543474 | 0.47 |
| rs12097673 | chr1:2843180 | 9.33E-15 | cg00996827 | chr1:2838805 | 0.55 |
| rs62120467 | chr2:3382624 | 9.82E-15 | cg01472464 | chr2:3383078 | 0.05 |
| rs7663448 | chr4:10054787 | 9.89E-15 | cg26043149 | chr18:55253948 | 0.28 |
| rs8140485 | chr22:50529146 | 1.05E-14 | cg24864161 | chr22:50528282 | 0.43 |
| rs9812936 | chr3:50043654 | 1.11E-14 | cg12257692 | chr3:49977190 | 0.97 |
| rs1055150 | chr19:18499784 | 1.15E-14 | cg21088460 | chr19:18499786 | 0.17 |
| rs1316524 | chr1:31972540 | 1.25E-14 | cg07096763 | chr1:31971752 | 0.48 |
| rs4708675 | chr6:168555071 | 1.34E-14 | cg04463397 | chr6:168556793 | 0.01 |
| rs4808736 | chr19:18117488 | 1.39E-14 | cg21649277 | chr19:18117794 | 0.75 |
| rs7606595 | chr2:15900274 | 1.43E-14 | cg26669897 | chr2:15909070 | 0.02 |
| rs7574691 | chr2:240044687 | 1.48E-14 | cg15635302 | chr2:240043224 | 0.72 |
| rs16961809 | chr19:29226299 | 1.51E-14 | cg25267487 | chr19:29217858 | 0.15 |
| rs28647894 | chr17:80340483 | 1.55E-14 | cg07797397 | chr2:177052527 | 0.13 |
| rs2665971 | chr17:74010038 | 1.58E-14 | cg10138630 | chr17:74024966 | 0.15 |
| rs6711715 | chr2:130330516 | 1.62E-14 | cg05962382 | chr2:130345044 | 0.72 |
| rs2003181 | chr14:105642016 | 1.63E-14 | cg15868425 | chr14:105643522 | 0.32 |
| rs12445614 | chr16:89166832 | 1.70E-14 | cg09427016 | chr16:89167018 | 0.91 |
| rs4145082 | chr6:86352016 | 1.75E-14 | cg03285617 | chr6:86179345 | 1.00 |
| rs12924275 | chr16:9191790 | 2.20E-14 | cg08831531 | chr16:9218945 | 0.12 |
| rs2872542 | chr20:61664872 | 2.81E-14 | cg08045932 | chr20:61659980 | 0.71 |
| rs7734278 | chr5:178761358 | 2.82E-14 | cg26694831 | chr5:178763419 | 0.11 |
| rs12517252 | chr5:149910986 | 2.83E-14 | cg02633363 | chr5:149906179 | 0.30 |
| rs7206985 | chr17:9159202 | 2.94E-14 | cg12361772 | chr17:9160821 | 0.21 |
| rs7545884 | chr1:67665785 | 3.00E-14 | cg23726106 | chr1:67600229 | 0.53 |
| rs1557026 | chr1:228383367 | 3.10E-14 | cg01200585 | chr1:228362443 | 0.45 |
| rs30927 | chr16:55317598 | 3.32E-14 | cg02198701 | chr16:55364614 | 0.06 |
| rs6711715 | chr2:130330516 | 3.50E-14 | cg05903289 | chr2:130345205 | 0.35 |
| rs7305397 | chr12:42850058 | 3.95E-14 | cg19980929 | chr12:42632907 | 0.70 |
| rs7710436 | chr5:54056541 | 3.99E-14 | cg06536806 | chr5:54081633 | 0.50 |
| rs58069944 | chr8:2294105 | 4.37E-14 | cg11900328 | chr8:2263331 | 0.91 |
| rs13224850 | chr7:40168950 | 4.41E-14 | cg13264672 | chr7:39993440 | 0.74 |
| rs7016869 | chr8:2474928 | 4.45E-14 | cg02472801 | chr8:2480483 | 0.49 |
| rs732215 | chr7:50544063 | 4.58E-14 | cg00647317 | chr7:50633725 | 0.04 |
| rs6500602 | chr16:4497451 | 4.86E-14 | cg01793945 | chr16:4420291 | 0.06 |
| rs12904722 | chr15:42304003 | 4.90E-14 | cg03080639 | chr15:42302379 | 0.80 |
| rs17333520 | chr11:31058834 | 4.92E-14 | cg06552810 | chr11:31128660 | 0.52 |
| rs2246207 | chr17:61987576 | 5.22E-14 | cg06873352 | chr17:61820015 | 0.33 |
| rs7470605 | chr9:136890107 | 5.38E-14 | cg13789015 | chr9:136890014 | 0.93 |
| rs9630726 | chr17:43068628 | 5.49E-14 | cg20215112 | chr17:43065055 | 0.45 |
| rs2295232 | chr6:153365384 | 6.81E-14 | cg17707550 | chr6:153380415 | 0.37 |
| rs10987908 | chr9:130936530 | 8.00E-14 | cg09976142 | chr9:130955436 | 0.85 |
| rs45537633 | chr6:41117824 | 8.16E-14 | cg09580153 | chr6:41068724 | 0.93 |
| rs6421977 | chr11:407708 | 8.33E-14 | cg18351999 | chr11:406901 | 0.69 |
| rs9782 | chr12:103351826 | 8.83E-14 | cg27569040 | chr12:103351855 | 0.44 |
| rs13269498 | chr8:54578332 | 9.49E-14 | cg10225865 | chr8:54605566 | 0.96 |
| rs6946060 | chr7:157644761 | 9.51E-14 | cg24524099 | chr7:157643007 | 0.74 |
| rs6702840 | chr1:246626857 | 9.63E-14 | cg04798314 | chr1:246668601 | 0.61 |
| rs7560311 | chr2:1803605 | 9.89E-14 | cg21862353 | chr2:1801628 | 0.35 |
| rs113935737 | chr15:65123276 | 1.09E-13 | cg07915896 | chr15:65129816 | 0.43 |
| rs9525735 | chr13:43661306 | 1.11E-13 | cg05035143 | chr13:43597297 | 0.03 |
| rs6946060 | chr7:157644761 | 1.14E-13 | cg22216157 | chr7:157643037 | 0.96 |
| rs6968990 | chr7:32552159 | 1.19E-13 | cg06627557 | chr7:32535165 | 0.21 |
| rs2872542 | chr20:61664872 | 1.25E-13 | cg23505145 | chr19:12996616 | 0.15 |
| rs74002504 | chr2:241828338 | 1.27E-13 | cg07537917 | chr2:241836409 | 0.41 |
| rs909832 | chr1:25754025 | 1.33E-13 | cg24991732 | chr1:25594486 | 0.27 |
| rs2637647 | chr10:133954267 | 1.35E-13 | cg02162534 | chr10:133956875 | 0.71 |
| rs10271372 | chr7:157793023 | 1.35E-13 | cg12440927 | chr7:157791721 | 0.27 |
| rs7765960 | chr6:13958385 | 1.43E-13 | cg24233211 | chr6:14002749 | 0.23 |
| rs143349430 | chr10:32183019 | 1.58E-13 | cg04359828 | chr10:32216031 | 0.10 |
| rs6946377 | chr7:64324352 | 1.62E-13 | cg16681239 | chr7:64349915 | 0.16 |
| rs13084718 | chr3:13610039 | 1.81E-13 | cg02146340 | chr3:13610059 | 0.18 |
| rs2376584 | chr17:76402116 | 2.10E-13 | cg02836325 | chr17:76403955 | 0.12 |
| rs4557742 | chr8:145508113 | 2.15E-13 | cg15151778 | chr8:145502134 | 0.21 |
| rs571780 | chr9:136001763 | 2.60E-13 | cg13753488 | chr9:136001623 | 0.83 |
| rs699664 | chr2:85780536 | 2.61E-13 | cg02493740 | chr2:85810744 | 0.36 |
| rs9998888 | chr4:824575 | 2.70E-13 | cg24793722 | chr4:824416 | 0.86 |
| rs4807546 | chr19:4182060 | 2.74E-13 | cg18542377 | chr19:4172961 | 0.27 |
| rs4334037 | chr11:69256482 | 2.91E-13 | cg23478547 | chr11:69259265 | 0.09 |
| rs2800973 | chr22:19168616 | 3.10E-13 | cg24911827 | chr22:19170109 | 0.51 |
| rs9861843 | chr3:54159358 | 3.11E-13 | cg12173409 | chr3:54154746 | 0.55 |
| rs6027929 | chr20:59535741 | 3.48E-13 | cg19181528 | chr20:59542589 | 0.16 |
| rs11251278 | chr10:2544043 | 3.58E-13 | cg05625103 | chr10:2543513 | 0.28 |
| rs2993317 | chr13:113687143 | 3.64E-13 | cg07204236 | chr13:113705910 | 0.66 |

**Supplementary Table 20: Methylation QTLs (mQTLs) identified in the cerebellum (CER) for SNPs included in the schizophrenia polygenic risk score.** Shown are associations between DNA methylation at specific Illumina 450K probes and genetic variants used to derive polygenic risk score (PRS) in our study, in addition to the corresponding *P*-values for PRS-associated DNA methylation variation at the same probe.

| **SNP** | **SNP genomic location (hg19)** | **mQTL *P*** | **CpG** | **CpG genomic location (hg19)** | **EWAS *P*** |
| --- | --- | --- | --- | --- | --- |
| rs12635522 | chr3:125708174 | 2.67E-45 | cg02807482 | chr3:125708958 | 0.53 |
| rs12635522 | chr3:125708174 | 1.67E-42 | cg15145296 | chr3:125709740 | 0.61 |
| rs2141182 | chr12:131401591 | 1.06E-41 | cg07816006 | chr12:131401305 | 0.99 |
| rs9726753 | chr1:153591652 | 2.20E-41 | cg08477332 | chr1:153590243 | 0.08 |
| rs12635522 | chr3:125708174 | 9.50E-41 | cg06494592 | chr3:125709126 | 0.59 |
| rs10205909 | chr2:3526603 | 2.28E-36 | cg21240684 | chr2:3526841 | 0.48 |
| rs557873 | chr9:135336912 | 2.36E-36 | cg21190742 | chr9:135336993 | 0.34 |
| rs2141182 | chr12:131401591 | 4.21E-35 | cg10021924 | chr12:131401275 | 0.92 |
| rs7803698 | chr7:64427895 | 4.72E-35 | cg24247132 | chr7:64458642 | 0.23 |
| rs12635522 | chr3:125708174 | 1.80E-34 | cg04553112 | chr3:125709451 | 0.28 |
| rs4789846 | chr17:80225545 | 8.17E-34 | cg02744699 | chr17:80197898 | 0.37 |
| rs117194038 | chr17:43927290 | 1.67E-33 | cg22968622 | chr17:43663579 | 0.52 |
| rs6680259 | chr1:7122308 | 3.31E-33 | cg20409752 | chr1:7122726 | 0.03 |
| rs4807546 | chr19:4182060 | 2.72E-31 | cg01287132 | chr19:4173254 | 0.74 |
| rs35059736 | chr7:158224692 | 6.83E-31 | cg01191920 | chr7:158217561 | 0.76 |
| rs1726866 | chr7:141672705 | 3.11E-30 | cg19476643 | chr7:141672455 | 0.80 |
| rs11712066 | chr3:151830309 | 8.01E-30 | cg27098685 | chr3:151867537 | 0.31 |
| rs10857676 | chr10:134970598 | 1.21E-29 | cg00753039 | chr10:134969141 | 0.55 |
| rs4792919 | chr17:41873309 | 4.15E-29 | cg26893861 | chr17:41843967 | 0.99 |
| rs3214023 | chr12:53682986 | 5.41E-29 | cg04065151 | chr12:53682969 | 0.88 |
| rs2800973 | chr22:19168616 | 8.83E-29 | cg02655711 | chr22:19163373 | 0.51 |
| rs2412322 | chr17:48578726 | 9.29E-29 | cg00901687 | chr17:48585270 | 0.01 |
| rs117194038 | chr17:43927290 | 5.51E-28 | cg10094238 | chr17:43483251 | 0.78 |
| rs4807546 | chr19:4182060 | 9.43E-28 | cg23999422 | chr19:4173466 | 0.48 |
| rs76344840 | chr9:33120204 | 1.29E-27 | cg20290983 | chr6:43655470 | 0.08 |
| rs10793287 | chr11:77846706 | 9.62E-27 | cg09721595 | chr11:77773924 | 0.67 |
| rs13065 | chr14:100996312 | 1.23E-26 | cg18516195 | chr14:101012996 | 0.15 |
| rs6968990 | chr7:32552159 | 3.17E-26 | cg06133097 | chr7:32552212 | 0.12 |
| rs4807546 | chr19:4182060 | 5.63E-26 | cg07492962 | chr19:4173315 | 0.96 |
| rs117194038 | chr17:43927290 | 5.91E-26 | cg27244773 | chr17:43483116 | 0.68 |
| rs4789846 | chr17:80225545 | 4.83E-25 | cg22805688 | chr17:80197841 | 0.19 |
| rs12507178 | chr4:86920281 | 5.94E-25 | cg13324779 | chr4:86923558 | 0.89 |
| rs650241 | chr11:75277757 | 1.13E-24 | cg26104986 | chr11:75275303 | 0.16 |
| rs62185165 | chr2:241251453 | 1.81E-24 | cg21947394 | chr2:241260110 | 0.19 |
| rs13226756 | chr7:149015681 | 2.87E-24 | cg07762347 | chr7:149016554 | 0.42 |
| rs2553022 | chr7:100401862 | 3.03E-24 | cg12616177 | chr7:100434510 | 0.91 |
| rs1341741 | chr10:888073 | 3.40E-24 | cg26597838 | chr10:835615 | 0.40 |
| rs16961809 | chr19:29226299 | 3.48E-24 | cg14983838 | chr19:29218262 | 0.04 |
| rs2468300 | chr12:84901620 | 3.71E-24 | cg09278098 | chr12:84902512 | 0.46 |
| rs4984688 | chr16:785717 | 5.25E-24 | cg18653534 | chr16:772142 | 0.28 |
| rs2800973 | chr22:19168616 | 5.97E-24 | cg24911827 | chr22:19170109 | 0.23 |
| rs12099513 | chr12:5267322 | 1.98E-23 | cg01414572 | chr12:5248588 | 0.67 |
| rs16961809 | chr19:29226299 | 6.17E-23 | cg12756686 | chr19:29218302 | 0.11 |
| rs2568198 | chr2:85403546 | 8.69E-23 | cg22128724 | chr2:85402928 | 0.20 |
| rs2342082 | chr12:123090380 | 1.11E-22 | cg23029597 | chr12:123009494 | 0.66 |
| rs164080 | chr5:141391532 | 1.24E-22 | cg25940447 | chr5:141391533 | 0.04 |
| rs28452050 | chr16:29317318 | 1.31E-22 | cg05645661 | chr16:29329490 | 0.38 |
| rs400132 | chr1:2141866 | 2.46E-22 | cg24578937 | chr1:2090814 | 0.95 |
| rs9812936 | chr3:50043654 | 2.71E-22 | cg05623727 | chr3:50126028 | 0.54 |
| rs2412322 | chr17:48578726 | 3.26E-22 | cg11440486 | chr17:48585216 | 0.03 |
| rs930526 | chr17:6473353 | 3.49E-22 | cg23551722 | chr17:6546898 | 0.70 |
| rs60668498 | chr19:44642672 | 3.60E-22 | cg23489630 | chr19:44645078 | 0.38 |
| rs62154034 | chr2:101953138 | 4.28E-22 | cg23685994 | chr2:101959022 | 0.75 |
| rs9921300 | chr16:5641315 | 7.95E-22 | cg03979510 | chr16:5641033 | 0.59 |
| rs8053397 | chr16:87573468 | 9.76E-22 | cg08031982 | chr16:87577539 | 0.79 |
| rs1345145 | chr8:102144280 | 2.66E-21 | cg13263591 | chr8:102142199 | 0.02 |
| rs16965349 | chr17:36614524 | 2.88E-21 | cg12050358 | chr17:36612909 | 0.80 |
| rs7496866 | chr15:27102200 | 3.36E-21 | cg10318222 | chr15:27111940 | 0.04 |
| rs1285820 | chr14:91840017 | 4.02E-21 | cg10511902 | chr14:91842949 | 0.19 |
| rs3760312 | chr17:13970282 | 1.02E-20 | cg27005118 | chr17:13972210 | 0.82 |
| rs2620032 | chr17:71745091 | 1.25E-20 | cg24457076 | chr17:71744550 | 0.52 |
| rs4807546 | chr19:4182060 | 1.89E-20 | cg09617135 | chr19:4173482 | 0.53 |
| rs8053397 | chr16:87573468 | 2.18E-20 | cg03020503 | chr16:87577655 | 0.38 |
| rs12672284 | chr7:32825576 | 2.92E-20 | cg11105292 | chr7:32802564 | 0.26 |
| rs2269481 | chr4:2386139 | 2.94E-20 | cg11208915 | chr4:2401758 | 0.47 |
| rs4729915 | chr7:103081125 | 3.42E-20 | cg21537297 | chr8:144298583 | 0.03 |
| rs7833924 | chr8:144996029 | 3.44E-20 | cg06045337 | chr8:145013910 | 0.47 |
| rs11738251 | chr5:177819313 | 3.72E-20 | cg00986130 | chr5:177821799 | 0.79 |
| rs4789846 | chr17:80225545 | 4.40E-20 | cg21034531 | chr17:80197757 | 0.89 |
| rs1780033 | chr1:118339119 | 5.46E-20 | cg01778345 | chr1:118427435 | 0.99 |
| rs4264326 | chr14:105411700 | 6.84E-20 | cg21017887 | chr14:105400489 | 0.00 |
| rs11146990 | chr12:133027749 | 7.52E-20 | cg15402627 | chr12:133021489 | 0.58 |
| rs75934331 | chr1:183182029 | 9.41E-20 | cg01417625 | chr1:183187433 | 0.75 |
| rs9769809 | chr7:64956889 | 1.20E-19 | cg24247132 | chr7:64458642 | 0.23 |
| rs4737 | chr16:75238103 | 1.23E-19 | cg06389950 | chr16:75240536 | 0.37 |
| rs1888502 | chr21:41545747 | 1.41E-19 | cg10828127 | chr21:41550814 | 0.67 |
| rs74002504 | chr2:241828338 | 1.85E-19 | cg07537917 | chr2:241836409 | 0.35 |
| rs1881191 | chr2:236607924 | 2.76E-19 | cg24888581 | chr2:236616026 | 0.47 |
| rs117194038 | chr17:43927290 | 3.41E-19 | cg17117718 | chr17:43663208 | 0.76 |
| rs6676743 | chr1:110296338 | 3.47E-19 | cg10807101 | chr1:110282274 | 0.33 |
| rs16961809 | chr19:29226299 | 4.01E-19 | cg03161606 | chr19:29218774 | 0.02 |
| rs6982268 | chr8:12994642 | 4.62E-19 | cg03231596 | chr8:12987546 | 0.45 |
| rs2447027 | chr10:14003363 | 4.69E-19 | cg27572370 | chr10:14002394 | 0.45 |
| rs2645673 | chr4:77816229 | 4.95E-19 | cg21917090 | chr4:77816250 | 0.22 |
| rs9933817 | chr16:83972660 | 5.08E-19 | cg16528738 | chr16:83968260 | 0.85 |
| rs17385407 | chr1:160473286 | 5.46E-19 | cg22696814 | chr1:160398070 | 0.05 |
| rs117194038 | chr17:43927290 | 5.58E-19 | cg14260695 | chr17:43506184 | 0.54 |
| rs7496866 | chr15:27102200 | 9.61E-19 | cg01378667 | chr15:27111911 | 0.07 |
| rs77841981 | chr7:5231658 | 9.82E-19 | cg07508942 | chr7:5267994 | 0.96 |
| rs3861297 | chr18:75380032 | 1.05E-18 | cg11874321 | chr18:75380364 | 0.45 |
| rs11248093 | chr4:2276047 | 1.20E-18 | cg19771469 | chr4:2275994 | 0.07 |
| rs144716806 | chr4:38854783 | 1.31E-18 | cg26681822 | chr4:38858561 | 0.98 |
| rs4314559 | chr7:2365892 | 1.32E-18 | cg16553052 | chr7:2349605 | 0.19 |
| rs7470605 | chr9:136890107 | 1.51E-18 | cg13789015 | chr9:136890014 | 0.09 |
| rs11146990 | chr12:133027749 | 1.92E-18 | cg11090202 | chr12:133021713 | 0.96 |
| rs4689604 | chr4:7129556 | 2.21E-18 | cg16307866 | chr4:7129517 | 0.94 |
| rs881347 | chr10:134000130 | 2.66E-18 | cg10144198 | chr10:134001728 | 0.43 |
| rs12595938 | chr16:8958081 | 3.12E-18 | cg08308162 | chr16:8889244 | 0.45 |
| rs1341741 | chr10:888073 | 3.31E-18 | cg20503657 | chr10:835505 | 0.49 |
| rs274692 | chr5:6734625 | 3.32E-18 | cg10857441 | chr5:6722123 | 0.16 |
| rs75231006 | chr14:102987539 | 3.89E-18 | cg23712530 | chr14:102964522 | 0.07 |
| rs7807840 | chr7:35834025 | 4.20E-18 | cg11531232 | chr7:35808197 | 0.16 |
| rs77841981 | chr7:5231658 | 4.76E-18 | cg12631105 | chr7:5267896 | 0.96 |
| rs77617940 | chr20:61814968 | 5.27E-18 | cg17237881 | chr20:61867533 | 0.59 |
| rs2276947 | chr4:8233943 | 5.40E-18 | cg25777912 | chr4:8233394 | 0.45 |
| rs11248093 | chr4:2276047 | 5.63E-18 | cg22029856 | chr4:2276003 | 0.24 |
| rs13147452 | chr4:1078124 | 6.30E-18 | cg13468214 | chr4:1046988 | 0.93 |
| rs11688491 | chr2:98167020 | 8.16E-18 | cg26665480 | chr2:98280029 | 0.92 |
| rs7710436 | chr5:54056541 | 1.12E-17 | cg06536806 | chr5:54081633 | 0.20 |
| rs73494059 | chr7:154468109 | 1.21E-17 | cg15618646 | chr7:154473202 | 0.64 |
| rs9390343 | chr6:146010527 | 1.21E-17 | cg25629118 | chr6:146113270 | 0.50 |
| rs117194038 | chr17:43927290 | 2.21E-17 | cg12609785 | chr17:43660871 | 0.51 |
| rs35037013 | chr10:54656418 | 2.36E-17 | cg05984115 | chr10:54631212 | 0.47 |
| rs11633474 | chr15:22939192 | 2.46E-17 | cg26344513 | chr15:22930613 | 0.58 |
| rs8082590 | chr17:17958402 | 2.56E-17 | cg04398451 | chr17:18023971 | 0.27 |
| rs11995562 | chr8:1427444 | 2.72E-17 | cg16582891 | chr8:1430303 | 0.09 |
| rs964757 | chr2:183114225 | 3.16E-17 | cg17054006 | chr2:183107046 | 0.38 |
| rs56340588 | chr7:127799341 | 4.36E-17 | cg02301128 | chr7:127792165 | 0.25 |
| rs62285061 | chr4:1688981 | 4.82E-17 | cg05026014 | chr4:1749153 | 0.19 |
| rs6946060 | chr7:157644761 | 5.48E-17 | cg24524099 | chr7:157643007 | 0.87 |
| rs74002504 | chr2:241828338 | 5.49E-17 | cg01588581 | chr2:241832900 | 0.34 |
| rs887687 | chr7:44185805 | 6.62E-17 | cg18628255 | chr7:44152333 | 0.32 |
| rs2666873 | chr8:55090128 | 6.67E-17 | cg20636351 | chr8:55087400 | 0.31 |
| rs6503422 | chr17:43163851 | 7.09E-17 | cg25538415 | chr17:43129957 | 0.50 |
| rs77841981 | chr7:5231658 | 9.84E-17 | cg16035714 | chr7:5267749 | 0.91 |
| rs73494059 | chr7:154468109 | 1.68E-16 | cg23463533 | chr7:154473289 | 0.90 |
| rs55674909 | chr15:31525516 | 1.97E-16 | cg03330558 | chr15:31516127 | 0.30 |
| rs11123564 | chr2:3683615 | 2.10E-16 | cg14926093 | chr2:3680421 | 0.55 |
| rs113661747 | chr14:75894945 | 2.26E-16 | cg22143352 | chr14:75897841 | 0.26 |
| rs3861297 | chr18:75380032 | 2.34E-16 | cg27582240 | chr18:75380514 | 0.76 |
| rs10271372 | chr7:157793023 | 2.57E-16 | cg12440927 | chr7:157791721 | 0.64 |
| rs2978902 | chr8:6690173 | 2.71E-16 | cg11878365 | chr8:6692387 | 1.00 |
| rs7455225 | chr7:73241386 | 2.93E-16 | cg02874145 | chr7:73246406 | 0.36 |
| rs6968990 | chr7:32552159 | 3.63E-16 | cg06627557 | chr7:32535165 | 0.01 |
| rs6514834 | chr20:18010631 | 3.71E-16 | cg02912291 | chr20:17944845 | 0.80 |
| rs4959982 | chr6:4464320 | 3.75E-16 | cg12916580 | chr6:4403020 | 0.35 |
| rs454759 | chr12:125799159 | 4.45E-16 | cg03923277 | chr12:104359732 | 0.43 |
| rs55674909 | chr15:31525516 | 4.89E-16 | cg12689679 | chr15:31516316 | 0.90 |
| rs890393 | chr18:74102435 | 5.18E-16 | cg24786174 | chr18:74118243 | 0.35 |
| rs34228916 | chr12:115942842 | 5.22E-16 | cg18639984 | chr12:115943877 | 0.46 |
| rs1476835 | chr4:17665456 | 5.29E-16 | cg04450456 | chr4:17643702 | 0.36 |
| rs2244746 | chr15:43695083 | 5.85E-16 | cg05490132 | chr15:43661835 | 0.12 |
| rs4689604 | chr4:7129556 | 6.23E-16 | cg13998369 | chr4:7129440 | 0.89 |
| rs12342201 | chr9:95894964 | 6.61E-16 | cg13713821 | chr9:95899302 | 0.05 |
| rs13147452 | chr4:1078124 | 6.95E-16 | cg09755784 | chr4:1047097 | 0.49 |
| rs6946060 | chr7:157644761 | 7.11E-16 | cg22216157 | chr7:157643037 | 0.81 |
| rs11675057 | chr2:26399481 | 7.13E-16 | cg22920501 | chr2:26401640 | 0.85 |
| rs1058167 | chr22:42538029 | 7.55E-16 | cg11915388 | chr22:42470451 | 0.00 |
| rs77841981 | chr7:5231658 | 8.64E-16 | cg01000248 | chr7:5267360 | 0.77 |
| rs13008444 | chr2:36909816 | 9.00E-16 | cg21931986 | chr2:36922916 | 0.16 |
| rs7018316 | chr8:144630169 | 9.41E-16 | cg16976870 | chr8:144631524 | 0.73 |
| rs13147452 | chr4:1078124 | 1.02E-15 | cg01815783 | chr4:1047043 | 0.84 |
| rs6711715 | chr2:130330516 | 1.03E-15 | cg05903289 | chr2:130345205 | 0.67 |
| rs7018316 | chr8:144630169 | 1.14E-15 | cg18649319 | chr8:144631768 | 0.68 |
| rs12129745 | chr1:28572317 | 1.26E-15 | cg04993605 | chr1:28573052 | 0.73 |
| rs7514450 | chr1:220991171 | 1.38E-15 | cg15450098 | chr1:221057561 | 0.04 |
| rs34474195 | chr1:3178582 | 1.41E-15 | cg26520908 | chr1:3191876 | 0.64 |
| rs2968475 | chr16:88976663 | 1.45E-15 | cg08484992 | chr16:88977278 | 0.88 |
| rs74002504 | chr2:241828338 | 1.53E-15 | cg04034577 | chr2:241836375 | 0.84 |
| rs74002504 | chr2:241828338 | 1.53E-15 | cg21187597 | chr2:241846305 | 0.71 |
| rs9912302 | chr17:44916982 | 1.62E-15 | cg25836567 | chr17:44929689 | 0.54 |
| rs13147452 | chr4:1078124 | 1.71E-15 | cg10407489 | chr4:1043616 | 0.83 |
| rs8116218 | chr20:44496330 | 1.80E-15 | cg04807470 | chr20:44452801 | 0.00 |
| rs1919784 | chr7:33105268 | 2.04E-15 | cg22798885 | chr7:33102694 | 0.62 |
| rs7730045 | chr5:56077993 | 2.17E-15 | cg20203395 | chr5:56204925 | 0.44 |
| rs7376288 | chr4:741742 | 2.18E-15 | cg14024328 | chr4:719362 | 0.21 |
| rs3793202 | chr7:6207142 | 2.40E-15 | cg22849526 | chr7:6199437 | 0.62 |
| rs7833924 | chr8:144996029 | 2.52E-15 | cg27082292 | chr8:145001361 | 0.31 |
| rs72713299 | chr15:34034124 | 2.85E-15 | cg16888559 | chr15:34031029 | 0.09 |
| rs56303414 | chr16:67466435 | 3.04E-15 | cg25341653 | chr16:67233277 | 0.54 |
| rs3021270 | chr22:40396409 | 3.14E-15 | cg21771250 | chr22:40406049 | 0.83 |
| rs2270115 | chr17:9804724 | 3.29E-15 | cg26853458 | chr17:9805074 | 0.17 |
| rs3093182 | chr19:15994924 | 3.49E-15 | cg26851661 | chr19:16045708 | 0.73 |
| rs7730045 | chr5:56077993 | 3.53E-15 | cg18230493 | chr5:56204884 | 0.48 |
| rs2857851 | chr4:3043512 | 4.44E-15 | cg14003022 | chr4:3043019 | 1.00 |
| rs62285061 | chr4:1688981 | 5.82E-15 | cg08488569 | chr4:1749241 | 0.44 |
| rs137934836 | chr17:16312563 | 6.28E-15 | cg08466034 | chr17:16318930 | 0.92 |
| rs72755098 | chr15:64427587 | 6.84E-15 | cg02848875 | chr15:64387786 | 0.32 |
| rs7549293 | chr1:205312280 | 7.42E-15 | cg00407231 | chr1:205312199 | 0.67 |
| rs732215 | chr7:50544063 | 8.49E-15 | cg00647317 | chr7:50633725 | 0.12 |
| rs9902733 | chr17:7271219 | 8.50E-15 | cg25737411 | chr17:7286288 | 0.36 |
| rs1350543 | chr4:56014389 | 8.63E-15 | cg09978860 | chr4:56023921 | 0.86 |
| rs72634702 | chr1:3661182 | 8.84E-15 | cg19903298 | chr1:3659644 | 0.84 |
| rs557888 | chr11:94258480 | 9.91E-15 | cg20289045 | chr11:94270260 | 0.93 |
| rs557934 | chr1:182548607 | 1.01E-14 | cg27563952 | chr1:182557982 | 0.22 |
| rs10079713 | chr5:28692468 | 1.09E-14 | cg07881623 | chr6:80731107 | 0.38 |
| rs8116218 | chr20:44496330 | 1.14E-14 | cg12112556 | chr20:44455373 | 0.00 |
| rs2221903 | chr4:123538912 | 1.17E-14 | cg10583651 | chr4:123538969 | 0.71 |
| rs117194038 | chr17:43927290 | 1.18E-14 | cg05485769 | chr17:44820573 | 0.93 |
| rs256881 | chr5:16572798 | 1.20E-14 | cg24531590 | chr5:16559541 | 0.57 |
| rs117194038 | chr17:43927290 | 1.28E-14 | cg10780632 | chr17:43973522 | 0.67 |
| rs55674909 | chr15:31525516 | 1.32E-14 | cg19666541 | chr15:31516111 | 0.50 |
| rs6518257 | chr21:47210107 | 1.37E-14 | cg18931629 | chr21:47287357 | 0.45 |
| rs4915215 | chr1:201077212 | 1.44E-14 | cg22815214 | chr1:201083145 | 0.29 |
| rs9615062 | chr22:45602573 | 1.46E-14 | cg20078807 | chr22:45608713 | 0.30 |
| rs9881242 | chr3:134032225 | 1.46E-14 | cg26387619 | chr3:134032421 | 0.21 |
| rs13147452 | chr4:1078124 | 1.52E-14 | cg27284194 | chr4:1044797 | 0.75 |
| rs16961809 | chr19:29226299 | 1.61E-14 | cg25267487 | chr19:29217858 | 0.03 |
| rs7455225 | chr7:73241386 | 1.62E-14 | cg17787108 | chr7:73246044 | 0.46 |
| rs6504120 | chr17:60830725 | 1.67E-14 | cg23831897 | chr17:60827363 | 0.15 |
| rs62444320 | chr7:5111830 | 1.70E-14 | cg02215787 | chr7:5107766 | 0.45 |
| rs4557742 | chr8:145508113 | 1.71E-14 | cg15151778 | chr8:145502134 | 0.13 |
| rs571780 | chr9:136001763 | 1.91E-14 | cg13753488 | chr9:136001623 | 0.85 |
| rs9826313 | chr3:57943818 | 1.93E-14 | cg07735586 | chr3:57945651 | 0.18 |
| rs2062480 | chr1:197905400 | 1.96E-14 | cg00114966 | chr1:197893920 | 0.09 |
| rs2872542 | chr20:61664872 | 2.22E-14 | cg16240275 | chr20:61666158 | 0.86 |
| rs7539178 | chr1:65383002 | 2.27E-14 | cg09765463 | chr1:65393430 | 0.43 |
| rs28676999 | chr15:40569884 | 2.33E-14 | cg19335742 | chr15:40566880 | 0.43 |
| rs2637657 | chr10:133993806 | 2.35E-14 | cg18037376 | chr10:134004552 | 0.82 |
| rs111543213 | chr19:37642385 | 2.43E-14 | cg08835041 | chr19:37461278 | 0.55 |
| rs11230570 | chr11:60782634 | 2.49E-14 | cg27098804 | chr11:60776124 | 0.28 |
| rs117194038 | chr17:43927290 | 2.52E-14 | cg01341218 | chr17:43662625 | 0.08 |
| rs13724 | chr8:142221032 | 2.64E-14 | cg17525220 | chr8:142204116 | 0.81 |
| rs1554948 | chr17:7286326 | 2.72E-14 | cg18632631 | chr17:7284049 | 0.18 |
| rs903759 | chr2:241078945 | 2.81E-14 | cg03314473 | chr2:241083794 | 0.39 |
| rs3907645 | chr4:7338501 | 2.97E-14 | cg19931925 | chr4:7338730 | 0.72 |
| rs62014776 | chr16:1339750 | 3.05E-14 | cg03705235 | chr16:1371463 | 0.94 |
| rs8053397 | chr16:87573468 | 3.13E-14 | cg16596957 | chr16:87575150 | 0.91 |
| rs1554948 | chr17:7286326 | 3.19E-14 | cg25737411 | chr17:7286288 | 0.36 |
| rs9627788 | chr22:50300438 | 3.58E-14 | cg15880211 | chr22:50250494 | 0.55 |
| rs12761857 | chr10:7105868 | 3.74E-14 | cg00998146 | chr19:18284560 | 0.43 |
| rs2159397 | chr17:14486205 | 3.93E-14 | cg17334453 | chr17:14479244 | 0.02 |
| rs1055150 | chr19:18499784 | 4.00E-14 | cg21088460 | chr19:18499786 | 0.25 |
| rs67327962 | chr16:89366932 | 4.06E-14 | cg27251473 | chr16:89359053 | 0.40 |
| rs11136381 | chr8:1273538 | 4.41E-14 | cg24513387 | chr8:1273604 | 0.16 |
| rs7252903 | chr19:16075619 | 4.61E-14 | cg26851661 | chr19:16045708 | 0.73 |
| rs9817966 | chr3:46650540 | 4.72E-14 | cg24524379 | chr3:46600244 | 0.09 |
| rs1996370 | chr11:35546199 | 4.84E-14 | cg14642338 | chr11:35547903 | 0.81 |
| rs2297776 | chr9:34372931 | 5.03E-14 | cg14096074 | chr9:34255149 | 0.82 |
| rs7702622 | chr5:122548721 | 5.23E-14 | cg04547002 | chr5:122551801 | 0.63 |
| rs2367209 | chr3:160398885 | 6.09E-14 | cg03789276 | chr3:160170225 | 0.03 |
| rs73494059 | chr7:154468109 | 6.11E-14 | cg11537355 | chr7:154473324 | 0.38 |
| rs7920264 | chr10:134046083 | 6.53E-14 | cg05225883 | chr10:134043755 | 0.55 |
| rs12608939 | chr19:3652680 | 6.99E-14 | cg10996109 | chr19:3637309 | 0.24 |
| rs340111 | chr5:178773203 | 7.06E-14 | cg27054655 | chr5:178772969 | 0.35 |
| rs10773762 | chr12:130765366 | 7.10E-14 | cg14604444 | chr12:130766091 | 0.29 |
| rs117194038 | chr17:43927290 | 7.28E-14 | cg08318660 | chr17:44122580 | 0.64 |
| rs2665971 | chr17:74010038 | 7.35E-14 | cg00498401 | chr17:74024829 | 0.23 |
| rs9615062 | chr22:45602573 | 7.41E-14 | cg02541592 | chr22:45608686 | 0.09 |
| rs6959895 | chr7:142434960 | 7.66E-14 | cg02329916 | chr7:142457299 | 0.71 |
| rs2290769 | chr17:73826406 | 9.20E-14 | cg06407111 | chr17:73872650 | 0.63 |
| rs71559409 | chr7:101006835 | 9.39E-14 | cg15127702 | chr7:101079617 | 0.41 |
| rs2475509 | chr6:39890217 | 9.54E-14 | cg10871120 | chr6:39891273 | 0.03 |
| rs11738251 | chr5:177819313 | 1.02E-13 | cg06730250 | chr5:177821870 | 0.57 |
| rs1557026 | chr1:228383367 | 1.12E-13 | cg24846680 | chr1:228362309 | 0.43 |
| rs56303414 | chr16:67466435 | 1.15E-13 | cg19514469 | chr16:67233432 | 0.36 |
| rs1072231 | chr2:118634348 | 1.17E-13 | cg24461052 | chr2:118607738 | 0.89 |
| rs12473344 | chr2:106959895 | 1.18E-13 | cg24419520 | chr2:106959257 | 0.55 |
| rs7730045 | chr5:56077993 | 1.19E-13 | cg24531977 | chr5:56204891 | 0.84 |
| rs7305397 | chr12:42850058 | 1.20E-13 | cg19980929 | chr12:42632907 | 0.89 |
| rs9365604 | chr6:164171914 | 1.20E-13 | cg18405330 | chr6:164171960 | 0.53 |
| rs2317947 | chr1:55431003 | 1.25E-13 | cg15129052 | chr1:55416755 | 0.62 |
| rs342778 | chr13:53185730 | 1.25E-13 | cg05335186 | chr13:53173507 | 0.31 |
| rs13268456 | chr8:28473911 | 1.29E-13 | cg23665710 | chr8:28476677 | 0.97 |
| rs66964681 | chr2:242915902 | 1.42E-13 | cg23069297 | chr2:242833648 | 0.81 |
| rs7803698 | chr7:64427895 | 1.43E-13 | cg12143784 | chr7:64541923 | 0.63 |
| rs36062268 | chr9:27585697 | 1.45E-13 | cg14297867 | chr9:27526172 | 0.39 |
| rs11149799 | chr16:75174049 | 1.48E-13 | cg00897404 | chr16:75182368 | 0.53 |
| rs1957841 | chr14:89588834 | 1.61E-13 | cg09271279 | chr14:89588740 | 0.38 |
| rs135572 | chr22:46527242 | 1.64E-13 | cg00004775 | chr22:46516503 | 0.32 |
| rs7514450 | chr1:220991171 | 1.67E-13 | cg26440142 | chr1:221057573 | 0.21 |
| rs12976309 | chr19:3864966 | 1.75E-13 | cg22553301 | chr19:3839231 | 0.66 |
| rs7018316 | chr8:144630169 | 1.76E-13 | cg10438391 | chr8:144631915 | 0.79 |
| rs61821477 | chr1:159383337 | 1.77E-13 | cg25076881 | chr1:159409836 | 0.41 |
| rs73210894 | chr8:20019512 | 1.84E-13 | cg09628359 | chr8:20039283 | 0.75 |
| rs12433009 | chr14:104196405 | 1.86E-13 | cg01849466 | chr14:104193079 | 0.02 |
| rs6754331 | chr2:236441590 | 1.98E-13 | cg00059854 | chr2:236443857 | 0.76 |
| rs2816607 | chr14:105718385 | 2.25E-13 | cg10792982 | chr14:105748885 | 0.32 |
| rs2246207 | chr17:61987576 | 2.30E-13 | cg06873352 | chr17:61820015 | 0.76 |
| rs12291981 | chr11:681502 | 2.34E-13 | cg00115288 | chr11:705892 | 0.51 |
| rs6711715 | chr2:130330516 | 2.38E-13 | cg05962382 | chr2:130345044 | 0.91 |
| rs1474256 | chr15:79463847 | 2.39E-13 | cg17916960 | chr15:79447300 | 0.52 |
| rs11230570 | chr11:60782634 | 2.43E-13 | cg04046629 | chr11:60775831 | 0.70 |
| rs4793213 | chr17:41307101 | 2.48E-13 | cg23758822 | chr17:41437982 | 0.19 |
| rs1939686 | chr11:115785356 | 2.57E-13 | cg26145504 | chr11:115801162 | 0.09 |
| rs73036509 | chr12:6165814 | 2.58E-13 | cg04053108 | chr12:6166028 | 0.02 |
| rs939421 | chr3:46576081 | 2.91E-13 | cg24524379 | chr3:46600244 | 0.09 |
| rs1267813 | chr11:133981075 | 2.94E-13 | cg20138604 | chr11:134023651 | 0.90 |
| rs11871657 | chr17:49714763 | 3.15E-13 | cg05229989 | chr17:49724713 | 0.95 |
| rs11610602 | chr12:8070815 | 3.31E-13 | cg01627669 | chr12:8068706 | 0.42 |
| rs8044407 | chr16:29154849 | 3.33E-13 | cg07505478 | chr16:29193318 | 0.87 |
| rs58474699 | chr17:79257728 | 3.53E-13 | cg03823431 | chr17:79229385 | 0.82 |

**Supplementary Table 21: Total number of probe included/excluded in the analyses after data normalization and quality control.** LNDBB = MRC London Neurodegenerative Diseases Brain Bank, DBCBB = Douglas-Bell Canada Brain Bank.

|  | **Prefrontal cortex** | **Striatum** | **Hippocampus** | **Cerebellum** |
| --- | --- | --- | --- | --- |
| **LNDBB** | 415,426 /  70,151 | 419,489 /  66,088 | 417,213 /  68,364 | 410,756 /  74,821 |
| **DBCBB** | 417,033 /  68,544 | 417,470 /  68,107 | - | 417,039 /  68,538 |
| **Meta-analysis** | 413,201 /  72,376 | 417,046 /  68,531 | - | 409,311 /  76,266 |

**Supplementary Table 22:** **Primers and assay conditions for the bisulfite-pyrosequencing assay targeting the chr17:154410-154672 schizophrenia-associated differentially methylated region in *RPH3AL*.**

| **CpG** | **450K probe** | **Genomic coordinates (hg19)** | **Annealing PCR Temperature (ºC)** | **PCR primer reverse** | **PCR primer forward** | **Sequencing primer** |
| --- | --- | --- | --- | --- | --- | --- |
| 1 | - | chr17:154444 | 60 | 5'-ACAAAAATCCAA CCAAACTCATTAA-3' | 5'-Biotin-ATAATATAATTAGA GGGGAAGGAAGTT-3' | 5'-CCAAACTCATT AATTCTCCTA-3' |
| 2 | - | chr17:154429 |  |  |  |  |
| 3 | cg11940040 | chr17:154420 |  |  |  |  |

**REFERENCES**

1. McLean CY, Bristor D, Hiller M, Clarke SL, Schaar BT, Lowe CB *et al.* GREAT improves functional interpretation of cis-regulatory regions. *Nature biotechnology* 2010; **28**(5)**:** 495-501.

2. Pidsley R, Viana J, Hannon E, Spiers H, Troakes C, Al-Saraj S *et al.* Methylomic profiling of human brain tissue supports a neurodevelopmental origin for schizophrenia. *Genome Biol* 2014; **15**(10)**:** 483.

3. Schizophrenia Working Group of the Psychiatric Genomics C. Biological insights from 108 schizophrenia-associated genetic loci. *Nature* 2014; **511**(7510)**:** 421-427.
